# Comparative effectiveness of generic and brand-name medication use: A database study of US health insurance claims

**DOI:** 10.1371/journal.pmed.1002763

**Published:** 2019-03-13

**Authors:** Rishi J. Desai, Ameet Sarpatwari, Sara Dejene, Nazleen F. Khan, Joyce Lii, James R. Rogers, Sarah K. Dutcher, Saeid Raofi, Justin Bohn, John G. Connolly, Michael A. Fischer, Aaron S. Kesselheim, Joshua J. Gagne

**Affiliations:** 1 Division of Pharmacoepidemiology and Pharmacoeconomics, Department of Medicine, Brigham and Women’s Hospital and Harvard Medical School, Boston, Massachusetts, United States of America; 2 Office of Surveillance and Epidemiology, Center for Drug Evaluation and Research, Food and Drug Administration, Silver Spring, Maryland, United States of America; 3 Center for Drug Evaluation and Research, Food and Drug Administration, Silver Spring, Maryland, United States of America; 4 Harvard School of Public Health, Boston, Massachusetts, United States of America; Imperial College London, UNITED KINGDOM

## Abstract

**Background:**

To the extent that outcomes are mediated through negative perceptions of generics (the nocebo effect), observational studies comparing brand-name and generic drugs are susceptible to bias favoring the brand-name drugs. We used authorized generic (AG) products, which are identical in composition and appearance to brand-name products but are marketed as generics, as a control group to address this bias in an evaluation aiming to compare the effectiveness of generic versus brand medications.

**Methods and findings:**

For commercial health insurance enrollees from the US, administrative claims data were derived from 2 databases: (1) Optum Clinformatics Data Mart (years: 2004–2013) and (2) Truven MarketScan (years: 2003–2015). For a total of 8 drug products, the following groups were compared using a cohort study design: (1) patients switching from brand-name products to AGs versus generics, and patients initiating treatment with AGs versus generics, where AG use proxied brand-name use, addressing negative perception bias, and (2) patients initiating generic versus brand-name products (bias-prone direct comparison) and patients initiating AG versus brand-name products (negative control). Using Cox proportional hazards regression after 1:1 propensity-score matching, we compared a composite cardiovascular endpoint (for amlodipine, amlodipine-benazepril, and quinapril), non-vertebral fracture (for alendronate and calcitonin), psychiatric hospitalization rate (for sertraline and escitalopram), and insulin initiation (for glipizide) between the groups. Inverse variance meta-analytic methods were used to pool adjusted hazard ratios (HRs) for each comparison between the 2 databases. Across 8 products, 2,264,774 matched pairs of patients were included in the comparisons of AGs versus generics. A majority (12 out of 16) of the clinical endpoint estimates showed similar outcomes between AGs and generics. Among the other 4 estimates that did have significantly different outcomes, 3 suggested improved outcomes with generics and 1 favored AGs (patients switching from amlodipine brand-name: HR [95% CI] 0.92 [0.88–0.97]). The comparison between generic and brand-name initiators involved 1,313,161 matched pairs, and no differences in outcomes were noted for alendronate, calcitonin, glipizide, or quinapril. We observed a lower risk of the composite cardiovascular endpoint with generics versus brand-name products for amlodipine and amlodipine-benazepril (HR [95% CI]: 0.91 [0.84–0.99] and 0.84 [0.76–0.94], respectively). For escitalopram and sertraline, we observed higher rates of psychiatric hospitalizations with generics (HR [95% CI]: 1.05 [1.01–1.10] and 1.07 [1.01–1.14], respectively). The negative control comparisons also indicated potentially higher rates of similar magnitude with AG compared to brand-name initiation for escitalopram and sertraline (HR [95% CI]: 1.06 [0.98–1.13] and 1.11 [1.05–1.18], respectively), suggesting that the differences observed between brand and generic users in these outcomes are likely explained by either residual confounding or generic perception bias. Limitations of this study include potential residual confounding due to the unavailability of certain clinical parameters in administrative claims data and the inability to evaluate surrogate outcomes, such as immediate changes in blood pressure, upon switching from brand products to generics.

**Conclusions:**

In this study, we observed that use of generics was associated with comparable clinical outcomes to use of brand-name products. These results could help in promoting educational interventions aimed at increasing patient and provider confidence in the ability of generic medicines to manage chronic diseases.

## Abstract 2

Rishi Desai & colleagues use data from two large US commercial insurance databases to compare brand-name versus authorised generic drugs and negative perception bias.

## Author summary

**Why was this study done?:**

- Empirical research investigating comparative outcomes of generic versus brand-name drug use in routine-care patient populations is limited. Many patients and providers perceive generics to be less effective and less safe than their brand-name counterparts.
- To the extent that outcomes are mediated through negative perceptions (the nocebo effect), observational studies comparing brand-name and generic drugs are susceptible to bias favoring the brand-name drugs. This study was done to assess the comparative effectiveness of generic versus brand-name drugs, while addressing the potential for perception bias.

**What did the researchers do and find?:**

- The authors used data from 2 large US commercial insurance databases to assess authorized generics, which are chemically identical to and share the same appearance as brand-name products, as proxies for brand-name drug use to account for potential bias due to negative perceptions towards generics in comparing the effectiveness of 8 brand-name versus generic products.
- After analyzing data from more than 3.5 million patients, the authors observed that use of generics provided comparable clinical outcomes to those of the brand-name products for chronic conditions including diabetes (glipizide), hypertension (amlodipine, amlodipine-benazepril, and quinapril), osteoporosis (alendronate and calcitonin salmon), and depression and anxiety (escitalopram and sertraline).

**What do these findings mean?:**

- These results may guide the development of educational interventions to address physicians’ and patients’ negative perceptions of generics and to increase awareness regarding the equivalence of generic and brand-name drugs.

## Introduction

Generic drugs are a critical component of the healthcare system, accounting for approximately 90% of all US prescriptions dispensed [1]. Generic drugs contain equivalent amounts of the same active ingredient(s) as their brand-name counterparts, but usually cost far less [2]. Some prior studies have demonstrated improved adherence with generic drugs compared to brand-name drugs, likely due to price [3,4]. Generics are approved by regulators based on evidence of pharmaceutical equivalence and bioequivalence with the brand-name product, even though they may contain different inactive ingredients. Still, many patients and providers perceive generics to be less effective and less safe than their brand-name counterparts [5–11]. Some patients explicitly express concerns about the effectiveness of generic drugs to treat their serious illnesses [12]. Negative expectations with generic products may lead patients to experience negative clinical outcomes due to a complex neurobiological phenomenon often described as the nocebo effect [13,14].

Randomized controlled trials comparing clinical outcomes between generic and brand-name products are rarely conducted as they are not required by regulators for generic drug approval. In certain cases—for example, when bioequivalence is disputed—rigorous evaluation of the comparative effectiveness of generic drug products in post-approval observational studies can be useful. However, in contrast to interventional studies, these observational studies do not have the advantage of blinding participants to the treatment they receive. As a result, if outcomes are mediated through negative perceptions of generic products, a theoretical concern is that comparative investigations in which participants are aware of their treatment assignment may be biased in favor of the brand-name product [13]. In a recent randomized study of patients taking a brand-name β-blocker placebo tablet, a switch to a “generic” different colored placebo tablet resulted in lower reductions in blood pressure and more adverse events compared with a group that continued receiving the brand-name placebo tablet [15].

**Fig 1:**
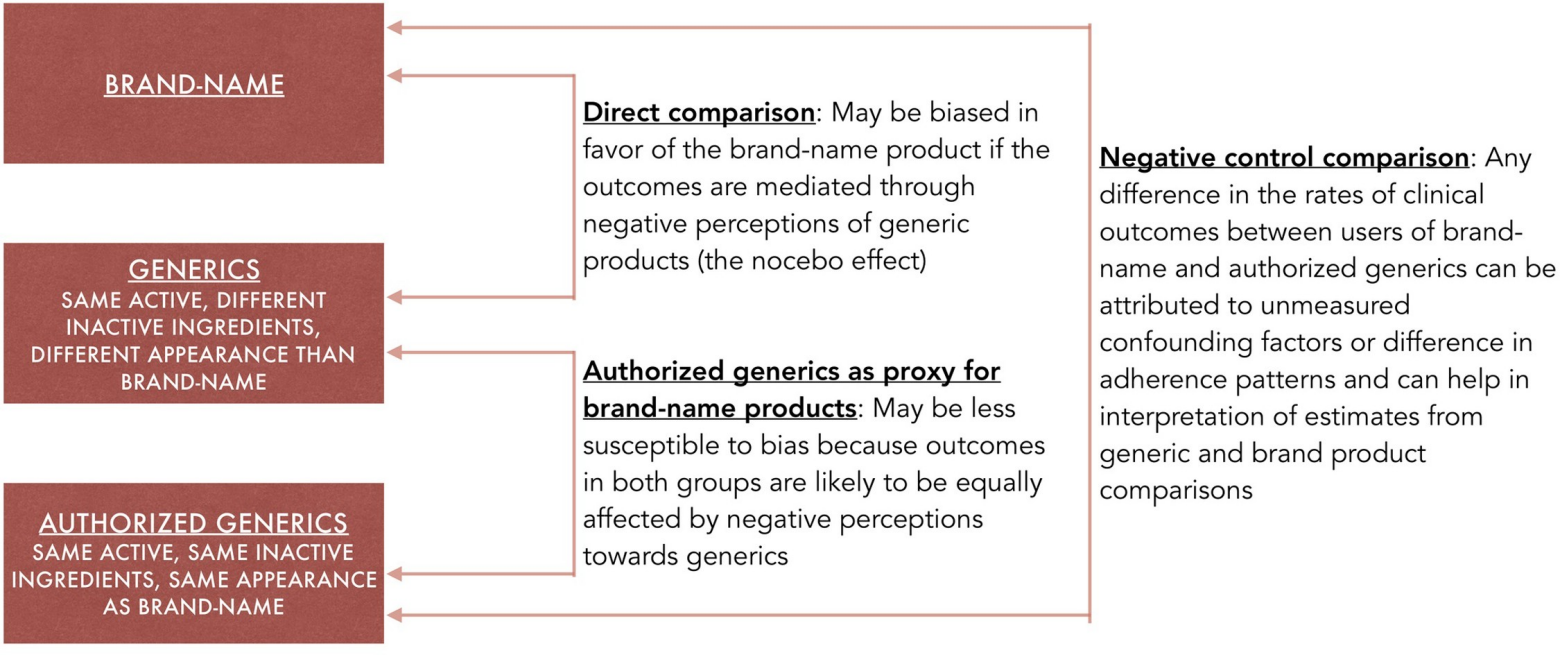
Study concept.

In this study comparing outcomes between generic and brand-name users across 8 drug products, we aimed to address potential bias due to negative perceptions by incorporating authorized generics (AGs) in the study design (Fig 1). AGs are a special type of generic; they are identical in composition and pill appearance to the brand-name product—but are marketed by brand manufacturers (or their licensees) as generics, usually after regulatory approval of other generic versions [16]. Considering AG users as a distinct exposure group, we designed 2 sets of analyses to compare outcomes between users of generics and brand-name products. In the first set of analyses, we compared outcomes between generic and AG users, with AG use as a proxy for brand-name use. These analyses were designed to address possible negative perception bias against generics under the assumption that, if such bias existed, it would impact outcomes equally in both the generic and the AG groups, since patients would not be aware of the distinction between generic and AG products and patients in both groups would believe they were taking generics. In the second set of analyses, we compared outcomes between generic users and brand-name users and between AG users and brand-name users, the latter representing a negative control. Since AGs and their corresponding brand-name products have identical active and inactive ingredients, any observed differences in the rates of clinical outcomes between users of AGs and brand products can be attributed to unmeasured confounding factors or differences in adherence patterns, which can help in interpretation of generic and brand product comparisons.

Two previous studies have used AGs to account for generic perception bias in comparative evaluations of brand-name versus generic medications [17,18]. The first study had a small sample size of approximately 5,000 patients, which precluded analysis by specific drug product [17]. The second study used the US Food and Drug Administration (FDA) Adverse Event Reporting System to compare adverse event reporting patterns between generics and AGs [18], but this data source has important limitations including substantial under-reporting and limited ability to assess whether patients used brand or generic versions of drugs [19]. The objective of our study was to overcome these limitations by comparing the effectiveness of brand, generic, and AG versions of 8 different drug products in 2 population-based data sources, allowing for control of potential bias due to negative perceptions.

## Methods

This study was undertaken as a part of a Cooperative Agreement (U01) with the FDA Office of Generic Drugs. As part of the funding proposal (S1 Text), we specified the study hypotheses, the pool of drugs of interest, the clinical outcomes of interest, and the analytic techniques to be used. Changes in the study methods to strengthen the study design, and for practical purposes, are detailed below. This study is reported as per the Strengthening the Reporting of Observational Studies in Epidemiology (STROBE) guidelines (S1 STROBE checklist).

### Data sources

Data were derived from 2 large US-based health insurance claims databases: (1) Truven MarketScan (2003 to 2015) and (2) Optum Clinformatics Data Mart (2004 to 2013). Both sources contain de-identified data that are captured during billing of routine healthcare encounters. Individuals with employer-sponsored commercial and Medicare Advantage health insurance plans from all 50 US states and the District of Columbia are represented in these data sources. In the included study years, Truven MarketScan captured the healthcare experiences of 173 million unique individuals, and Optum Clinformatics captured 55 million unique individuals. Comprehensive longitudinal information on demographics, coded inpatient and outpatient diagnoses and procedures, and outpatient prescription dispensing is recorded for all enrollees in these databases during the time of their health insurance enrollment. We selected these data sources for their size, representativeness of the employed US population, and ability to reliably identify generic-level prescription drug dispensing through National Drug Codes (NDCs). The study was approved by the Brigham and Women’s Hospital Institutional Review Board and the FDA’s Research in Human Subjects Committee.

### Drug products

We had initially proposed a list of 25 drugs with marketed AGs to be included in this analysis (S1 Text). This list was modified to include 20 products based on the following criteria: (1) restriction to products for which generics entered the market after 2003, which marks the start of availability of data in our sources; (2) restriction to products for which generics and AGs entered the market concurrently (i.e., within 30 days of each other), because non-concurrently marketed generics and AGs were thought to have fundamentally different use and switching patterns, which we believed could introduce bias; and (3) inclusion of a combination product (amlodipine-benazepril capsules) to expand the scope of the study to include generics of combination products. Of these 20 products, we differentiated generics from AGs using NDCs derived from the FDA NDC directory and excluded 12 products for which >5% of filled prescriptions in our data sources had NDCs that were not found in the NDC directory. The most likely reason for the absence of these NDCs is that the NDC directory does not maintain details on discontinued NDCs. Furthermore, the quality of recording in the NDC directory is dependent upon manufacturer submissions to the FDA, which could also introduce error or missingness. The following 8 products met our inclusion criteria: alendronate tablets, amlodipine tablets, amlodipine-benazepril capsules, calcitonin salmon nasal spray, escitalopram tablets, glipizide extended release (ER) tablets, quinapril tablets, and sertraline tablets.

### Study design

#### Evaluation of comparative outcomes between generic and AG users

For all included drug products, we compared outcomes in 2 separate patient cohorts of AG and generic users: (1) patients who had previously been dispensed the brand-name version of the drug and then switched to either an AG or generic and (2) patients who had not been dispensed the brand-name version in the prior 6 months and initiated either an AG or generic. To identify these groups, we identified patients’ first prescription for the AG or generic version of a drug after a period of 6 months of continuous health plan enrollment. This prescription date was defined as the index date, and the 6-month pre-index period was defined as the baseline period. If patients had a record of a brand-name prescription for the same drug product in the baseline period, we defined them as having switched to AG or generic from the brand-name product. If they had no record of a brand-name prescription for the same drug product in the baseline period, we defined them as AG or generic initiators. Since the AGs are identical in composition and appearance to their brand-name counterparts, the comparison of AG and generic initiators addressed the clinical question of the comparative effectiveness of the brand versus generic product among patients initiating a new treatment episode with that particular treatment, while the comparison of patients switching to AG or generic from a brand-name product addressed the clinical question of the comparative effectiveness for patients who continued on the brand-name product versus patients who switched to a generic version.

#### Evaluation of comparative outcomes between generic and brand users

Next, we compared outcomes directly between initiators of generics and initiators of brand-name products. As a negative control comparison, we also compared outcomes between initiators of AGs and initiators of the brand products. For both comparisons, patients were required to have no recorded use of any version of the medication of interest in the 6-month continuous health plan enrollment period (baseline period) before commencing any type or formulation of the drug of interest.

### Outcomes of interest and follow-up

Clinical outcomes of interest were defined based on the approved indications for the specific drug products. For the drugs treating cardiovascular disease—amlodipine tablets, amlodipine-benazepril capsules, and quinapril tablets—the outcome of interest was a composite cardiovascular endpoint comprising hospitalization with myocardial infarction or ischemic stroke as the primary discharge diagnosis or having a coronary revascularization procedure [20,21]. For alendronate tablets and calcitonin salmon nasal spray, the outcome of interest was a composite non-vertebral fracture endpoint of humerus, wrist, hip, or pelvis fracture identified using discharge diagnosis codes from hospital admission claims or a combination of diagnosis codes and surgical procedures from outpatient claims [22]. As escitalopram tablets and sertraline tablets are used for a variety of psychiatric conditions, including but not limited to major depressive disorder and generalized anxiety disorder, we reasoned that an endpoint that captured deterioration of patients’ mental health would be suitable for comparing the effectiveness for these drugs. While specific mental health scales are not available in administrative claims, healthcare services utilization for those with mental health diagnoses is well captured. Therefore, we selected hospitalization with a psychiatric condition as the principal discharge diagnosis code as the outcome of interest for escitalopram and sertraline. For glipizide ER tablets, the outcome was initiation of insulin during the follow-up period, with the rationale that a need for treatment intensification could serve as a proxy for poor glycemic control with current treatment [23]. Table A in S1 Appendix provides specific codes used to identify these outcomes.

Follow-up for the clinical outcome of interest began on the day following the index date. Follow-up continued until the occurrence of an outcome of interest, health plan disenrollment, drug discontinuation (defined as no dispensing of the index agent for at least 1 month after the end of the most recent prescription days’ supply), a switch to a different version of the drug, or the end of the study period.

### Covariates

In each comparison, we identified and accounted for a core set of variables that included patient demographics (age, sex, and geographic region), a combined comorbidity score [24], calendar year, and healthcare utilization factors as markers of contact with the healthcare system and general patient health, including the number of distinct prescription medications, outpatient visits, emergency department visits, and hospitalizations during the 6-month baseline period. Additionally, for each comparison, we identified and included risk factors for the outcome of interest. For example, we adjusted for hypertension, hyperlipidemia, and diabetes diagnoses, and use of other drugs treating cardiovascular disease (e.g., angiotensin converting enzyme inhibitors and angiotensin receptor blockers) for analyses of amlodipine tablets, amlodipine-benazepril capsules, and quinapril tablets. Calendar year was not included in the brand-name versus generic or brand-name versus AG comparisons because it had an extremely strong association with the exposure to brand and only weak associations with outcomes. Including such variables in statistical models is known to increase variance and can introduce additional bias [25]. To minimize the impact of confounding by secular trends in brand-name versus generic and brand-name versus AG comparisons, we restricted these 2 comparisons to 2 years before and 2 years after the loss of brand-name market exclusivity for each product. All covariates were measured in the 6-month pre-index period. For a full list of covariates used in each analysis, please refer to Tables B–I and R–Y in S1 Appendix.

### Statistical analysis

Propensity score (PS)–based methods were used for confounding adjustment. PSs were calculated separately within each database and for each comparison as the predicted probability of being in the exposure group of interest (AGs in the AG versus generic and AG versus brand comparisons, and generics in the generic versus brand comparison) in logistic regression models conditional on the covariates described in Tables B–I and R–Y in S1 Appendix for each individual product [26]. One-to-one nearest-neighbor matching within a caliper of 0.025 of the PS was implemented for each comparison [27]. In the matched cohorts, incidence rates for the outcomes and incidence rate differences were calculated. Cox proportional hazards models were used to estimate outcome hazard ratios (HRs) and 95% confidence intervals (CIs). All analyses were conducted for each study drug in the 2 databases separately. Database-specific estimates were pooled using an inverse variance fixed-effects approach to provide a summary effect estimate for each comparison of interest. To address the possibility of population overlap between the 2 databases, we corrected the variance of our pooled HRs assuming 5% overlap between databases. Statistical analyses were conducted with the Aetion platform, version 2.1.2.

## Results

### Evaluation of comparative outcomes between generic and AG users

There were a total of 1,694,878 1:1 PS-matched pairs in the comparisons of generic and AG initiators and 569,896 1:1 PS-matched pairs in the comparisons of patients switching from brand-name to generic versus AG across the 8 drug products and 2 databases, with cohort entry dates ranging between 2003 and 2015. Table 1 summarizes the patient demographics and baseline diagnoses of the primary indications for all matched pairs for each drug product. The average age of patients included in the analysis varied substantially across drug products, with older age ranges observed for osteoporosis drugs (alendronate, 60–67 years; calcitonin salmon, 59–70 years) and cardiovascular drugs (amlodipine, 55–63 years; amlodipine-benazepril, 53–58 years; quinapril, 53–60 years) and younger age ranges seen for users of antidepressants (escitalopram, 39–50 years; sertraline 38–50 years). All patient characteristics were balanced between 1:1 PS-matched samples in each database. The distributions of patient characteristics for generic and AG users, including co-morbid conditions and co-medications, prior to PS matching are provided in Tables B–I in S1 Appendix, and after PS matching are provided in Tables J–Q in S1 Appendix.

**Table 1:** Characteristics of patients included in the evaluation of comparative outcomes for patients initiating authorized generics (AGs) versus generics and patients switching from brand-name to AGs versus generics after 1:1 propensity score matching in each database.

Characteristic	Optum				MarketScan			
	Patients initiating AGs	Patients initiating generics	Patients switching from brand-name to AGs	Patients switching from brand-name to generics	Patients initiating AGs	Patients initiating generics	Patients switching from brand-name to AGs	Patients switching from brand-name to generics
**Alendronate**								
Number of patients	2,433	2,433	6,332	6,332	11,963	11,963	29,985	29,985
Age: mean (SD)	60 (10)	61 (10)	62 (10)	62 (9)	63 (12)	63 (12)	67 (12)	67 (12)
Male sex: *n* (%)	217 (8.9%)	190 (7.8%)	508 (8.0%)	530 (8.4%)	1,233 (10.3%)	1,216 (10.2%)	2,540 (8.5%)	2,390 (8.0%)
Osteoporosis: *n* (%)	976 (40.1%)	959 (39.4%)	1,638 (25.9%)	1,640 (25.9%)	3,469 (29.0%)	3,347 (28.0%)	4,264 (14.2%)	4,202 (14.0%)
**Amlodipine**								
Number of patients	73,853	73,853	35,004	35,004	461,045	461,045	116,521	116,521
Age: mean (SD)	55 (12)	55 (12)	58 (11)	58 (11)	60 (14)	60 (14)	63 (13)	63 (13)
Male sex: *n* (%)	41,374 (56.0%)	41,591 (56.3%)	18,954 (54.1%)	18,987 (54.2%)	231,251 (50.2%)	231,779 (50.3%)	56,127 (48.2%)	56,315 (48.3%)
Hypertension: *n* (%)	54,318 (73.5%)	53,875 (72.9%)	25,023 (71.5%)	25,083 (71.7%)	294,395 (63.9%)	293,231 (63.6%)	61,749 (53.0%)	61,963 (53.2%)
**Amlodipine-benazepril**								
Number of patients	10,941	10,941	6,034	6,034	47,375	47,375	29,652	29,652
Age: mean (SD)	53 (11)	53 (12)	55 (10)	55 (10)	55 (12)	54 (12)	58 (12)	58 (12)
Male sex: *n* (%)	6,537 (59.7%)	6,626 (60.6%)	3,931 (65.1%)	3,926 (65.1%)	26,949 (56.9%)	27,314 (57.7%)	17,765 (59.9%)	17,878 (60.3%)
Hypertension: *n* (%)	7,648 (69.9%)	7,442 (68.0%)	4,205 (69.7%)	4,133 (68.5%)	28,929 (61.1%)	28,519 (60.2%)	16,882 (56.9%)	16,649 (56.1%)
**Calcitonin salmon**								
Number of patients	1,054	1,054	458	458	7,420	7,420	2,892	2,892
Age: mean (SD)	59 (13)	59 (13)	65 (11)	65 (11)	67 (15)	67 (15)	70 (13)	70 (12)
Male sex: *n* (%)	181 (17.2%)	191 (18.1%)	42 (9.2%)	44 (9.6%)	1,243 (16.8%)	1,265 (17.0%)	244 (8.4%)	252 (8.7%)
Osteoporosis: *n* (%)	438 (41.6%)	443 (42.0%)	133 (29.0%)	128 (27.9%)	2,506 (33.8%)	2,493 (33.6%)	633 (21.9%)	634 (21.9%)
**Escitalopram**								
Number of patients	24,445	24,445	12,693	12,693	127,803	127,803	134,311	134,311
Age: mean (SD)	39 (15)	39 (15)	46 (15)	46 (15)	43 (18)	43 (18)	50 (16)	50 (16)
Male sex: *n* (%)	8,340 (34.1%)	8,313 (34.0%)	3,785 (29.8%)	3,830 (30.2%)	41,048 (32.1%)	40,679 (31.8%)	40,019 (29.8%)	40,095 (29.9%)
Depression: *n* (%)	2,455 (10.0%)	2,451 (10.0%)	1,136 (8.9%)	1,185 (9.3%)	12,928 (10.1%)	12,406 (9.7%)	10,025 (7.5%)	10,222 (7.6%)
Anxiety: *n* (%)	2,541 (10.4%)	2,544 (10.4%)	1,258 (9.9%)	1,297 (10.2%)	11,363 (8.9%)	10,827 (8.5%)	9,566 (7.1%)	9,572 (7.1%)
**Glipizide**								
Number of patients	2,193	2,193	723	723	66,713	66,713	2,840	2,840
Age: mean (SD)	58 (11)	58 (12)	60 (10)	60 (11)	59 (13)	59 (13)	65 (11)	65 (12)
Male sex: *n* (%)	1,242 (56.6%)	1,237 (56.4%)	429 (59.3%)	438 (60.6%)	37,126 (55.7%)	37,120 (55.6%)	1440 (50.7%)	1413 (49.8%)
Diabetes mellitus: *n* (%)	1,414 (64.5%)	1,424 (64.9%)	642 (88.8%)	637 (88.1%)	52,524 (78.7%)	52,218 (78.3%)	2118 (74.6%)	2164 (76.2%)
**Quinapril**								
Number of patients	8,335	8,335	14,369	14,369	32,074	32,074	25,766	25,766
Age: mean (SD)	53 (12)	53 (12)	57 (10)	57 (10)	57 (13)	57 (13)	60 (12)	60 (12)
Male sex: *n* (%)	4,784 (57.4%)	4,787 (57.4%)	8,659 (60.3%)	8,625 (60.0%)	17,551 (54.7%)	17,642 (55.0%)	13,992 (54.3%)	14,004 (54.4%)
Hypertension: *n* (%)	5,085 (61.0%)	5,091 (61.1%)	9,467 (65.9%)	9,452 (65.8%)	16,398 (51.1%)	16,483 (51.4%)	11,250 (43.7%)	11,303 (43.9%)
**Sertraline**								
Number of patients	177,959	177,959	48,019	48,019	639,270	639,270	107,155	107,155
Age: mean (SD)	38 (15)	38 (15)	44 (14)	44 (14)	41 (17)	41 (18)	50 (17)	50 (17)
Male sex: *n* (%)	58,874 (33.1%)	58,888 (33.1%)	13,879 (28.9%)	13,789 (28.7%)	207,094 (32.4%)	206,597 (32.3%)	30,583 (28.5%)	30,548 (28.5%)
Depression: *n* (%)	16,977 (9.5%)	16,764 (9.4%)	3,762 (7.8%)	3,764 (7.8%)	59,015 (9.2%)	56,989 (8.9%)	5,536 (5.2%)	5,496 (5.1%)
Anxiety: *n* (%)	15,636 (8.8%)	15,353 (8.6%)	3,280 (6.8%)	3,293 (6.9%)	47,887 (7.5%)	46,455 (7.3%)	4,134 (3.9%)	4,114 (3.8%)

Incidence rates for individual endpoints were generally consistent in all comparisons across the 2 databases (Table 2). The rates of psychiatric hospitalization were lower among patients switching from brand-name products to generics or AGs (range 8.7 to 15.5/1,000 person-years) compared to patients initiating generics or AGs (range 21.2 to 49.8/1,000 person-years) in both databases for escitalopram and sertraline. The initiator group had high use of other antidepressants at baseline compared to patients switching from brand-name products to generics or AGs (Tables F and I in S1 Appendix), suggesting that the initiator group likely represented patients with treatment-resistant depression while the patients switching from brand-name products to generics or AGs represented patients continued on a single antidepressant.

**Table 2:** Outcome incidence rates for patients initiating authorized generics (AGs) versus generics, and patients switching from brand-name products to AGs versus generics, after 1:1 propensity score matching in each database.

Characteristic	Optum				MarketScan			
	Patients initiating AGs	Patients initiating generics	Patients switching from brand-name to AGs	Patients switching from brand-name to generics	Patients initiating AGs	Patients initiating generics	Patients switching from brand-name to AGs	Patients switching from brand-name to generics
**Alendronate**								
Sample size	2,433	2,433	6,332	6,332	11,963	11,963	29,985	29,985
Total person-years	626	1,602	2,378	6,212	3,069	8,540	11,406	29,819
*n* fracture events	11	13	33	65	49	125	164	412
Incidence rate/1,000 py	17.57	8.11	13.88	10.46	15.97	14.64	14.38	13.82
Incidence rate difference (95% CI)	9.46 (−1.82, 20.74)	Ref	3.42 (−1.96, 8.79)	Ref	1.33 (−3.83, 6.48)	Ref	0.56 (−2.01, 3.14)	Ref
**Amlodipine**								
Sample size	73,853	73,853	35,004	35,004	461,045	461,045	116,521	116,521
Total person-years	46,443	47,776	35,451	41,324	328,747	306,866	133,303	134,765
*n* composite cardiovascular endpoint events	925	939	601	668	6,531	6,125	2,370	2,616
Incidence rate/1,000 py	19.92	19.65	16.95	16.17	19.87	19.96	17.78	19.41
Incidence rate difference (95% CI)	0.26 (−1.53, 2.06)	Ref	0.79 (−1.04, 2.62)	Ref	−0.09 (−0.79, 0.60)	Ref	−1.63 (−2.66, −0.60)	Ref
**Amlodipine-benazepril**								
Sample size	10,941	10,941	6,034	6,034	47,375	47,375	29,652	29,652
Total person-years	5,866	8,213	4,446	5,790	25,821	42,414	24,656	34,162
*n* composite cardiovascular endpoint events	104	99	49	50	316	427	263	398
Incidence rate/1,000 py	17.73	12.05	11.02	8.64	12.24	10.07	10.67	11.65
Incidence rate difference (95% CI)	5.67 (1.52, 9.83)	Ref	2.39 (−1.52, 6.29)	Ref	2.17 (0.52, 3.82)	Ref	−0.98 (−2.71, 0.74)	Ref
**Calcitonin salmon**								
Sample size	1,054	1,054	458	458	7,420	7,420	2,892	2,892
Total person-years	251	303	223	237	1,737	2,300	1,335	1,511
*n* fracture events	16	15	4	2	129	116	43	41
Incidence rate/1,000 py	63.71	49.46	17.92	8.44	74.26	50.44	32.22	27.13
Incidence rate difference (95% CI)	14.25 (−25.76, 54.27)	Ref	9.48 (−11.62, 30.58)	Ref	23.82 (8.06, 39.59)	Ref	5.09 (−7.62, 17.81)	Ref
**Escitalopram**								
Sample size	24,445	24,445	12,693	12,693	127,803	127,803	134,298	134,298
Total person-years	6,492	7,698	6,187	6,453	39,229	57,741	60,221	107,489
*n* psychiatric hospitalizations	323	356	85	100	1,429	1,766	595	936
Incidence rate/1,000 py	49.75	46.25	13.74	15.50	36.43	30.58	9.88	8.71
Incidence rate difference (95% CI)	3.50 (−3.74, 10.75)	Ref	−1.76 (−5.97, 2.46)	Ref	5.84 (3.48, 8.21)	Ref	1.17 (0.20, 2.14)	Ref
**Glipizide**								
Sample size	2,193	2,193	723	723	66,713	66,713		
Total person-years	1,745	1,719	553	576	44,940	42,077	2,470	2,473
*n* insulin initiations	122	94	34	29	3,869	3,088	159	119
Incidence rate/1,000 py	69.92	54.70	61.44	50.36	86.09	73.39	64.40	48.10
Incidence rate difference (95% CI)	15.23 (−1.39, 31.85)	Ref	11.08 (−16.53, 38.69)	Ref	12.70 (8.95, 16.45)	Ref	16.30 (3.00, 29.50)	Ref
**Quinapril**								
Sample size	8,335	8,335	14,369	14,369	32,074	32,074	25,766	25,766
Total person-years	5,476	4,352	14,797	11,893	22,580	19,778	35,060	22,057
*n* composite cardiovascular endpoint events	121	121	286	216	491	389	582	356
Incidence rate/1,000 py	22.10	27.80	19.33	18.16	21.74	19.67	16.60	16.14
Incidence rate difference (95% CI)	−5.70 (−12.03, 0.62)	Ref	1.17 (−2.13, 4.46)	Ref	2.08 (−0.67, 4.82)	Ref	0.46 (−1.69, 2.61)	Ref
**Sertraline**								
Sample size	177,959	177,959	48,019	48,019	639,272	639,272	107,150	107,150
Total person-years	71,333	77,623	32,544	33,825	260,109	314,073	72,249	88,924
*n* psychiatric hospitalizations	2,554	2,546	308	274	8,437	8,637	576	624
Incidence rate/1,000 py	35.80	32.80	9.46	8.10	32.44	27.50	7.97	7.02
Incidence rate difference (95% CI)	3.00 (1.12, 4.89)	Ref	1.36 (−0.06, 2.79)	Ref	4.94 (4.03, 5.84)	Ref	0.96 (0.10, 1.81)	Ref

CI, confidence interval; py, person-years.

**Fig 2:**
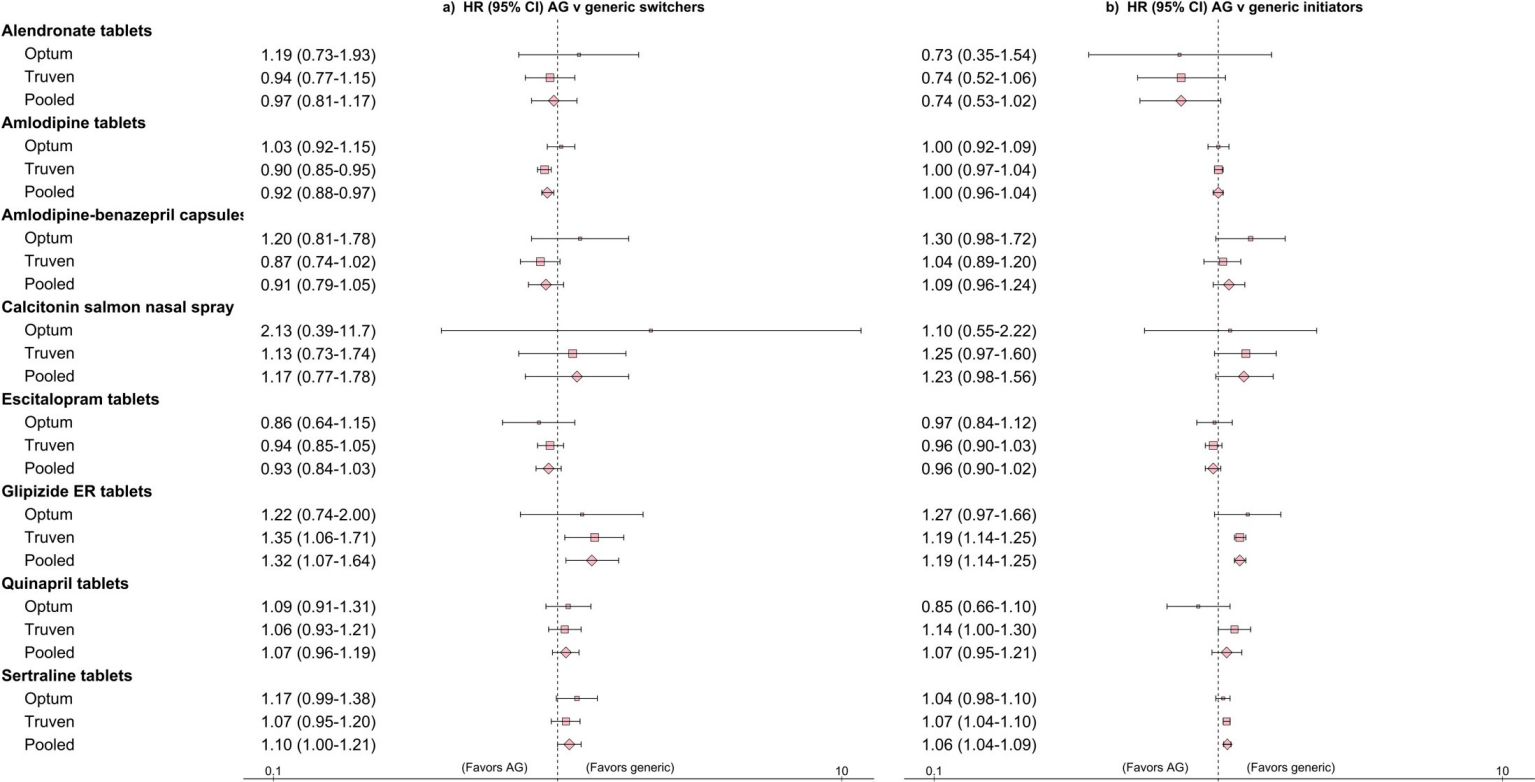
Hazard ratios (HRs) and 95% confidence intervals (CIs) comparing outcomes for patients initiating authorized generics (AGs) versus generics, and patients switching from brand-name products to AGs versus generics, after 1:1 propensity score matching in each database. The outcome for amlodipine tablets, amlodipine-benazepril capsules, and quinapril tablets was a composite endpoint comprising hospitalization for myocardial infarction, ischemic stroke, or coronary revascularization procedures. The outcome for alendronate tablets and calcitonin salmon nasal spray was a composite non-vertebral fracture endpoint comprising humerus, wrist, hip, or pelvis fractures. The outcome for escitalopram tablets and sertraline tablets was hospitalization with a psychiatric condition as the principal discharge diagnosis code. The outcome for glipizide extended release (ER) tablets was initiation of insulin during the follow-up period.

S1 Fig summarizes treatment effect estimates for each comparison before PS matching, and Fig 2 summarizes treatment effect estimates for each comparison after PS matching. After pooling the PS-matched results from the 2 databases, a majority of the estimates indicated no differences in the rates of outcomes between the AG and generic versions of drugs. Of the 4 estimates that reached statistical significance, 3 suggested better outcomes among users of generic versus AG products (HR [95% CI] for patients switching from brand-name glipizide to AG versus generic: 1.32 [1.07–1.64]; for glipizide AG versus generic initiators: 1.19 [1.14–1.25]; for sertraline AG versus generic initiators: 1.06 [1.04–1.09]). By contrast, the comparison of patients switching from brand-name amlodipine to generic versus AG favored the AG (HR [95% CI]: 0.92 [0.88–0.97]).

### Evaluation of comparative outcomes between generic and brand-name users

Across the 8 drug products and 2 databases, a total of 875,304 1:1 PS-matched pairs were included in the comparison of generic and brand-name initiators, and 437,857 1:1 PS-matched pairs were included in the comparison of AG and brand-name initiators. Table 3 summarizes the patient demographics and baseline diagnoses for primary indications for all matched pairs for each drug product. Distributions of baseline characteristics were similar for generic, AG, and brand-name initiators after 1:1 PS pair matching, for each drug product within each database (See Tables R–Y in S1 Appendix for distribution before PS matching and Tables Z–AG in S1 Appendix for distribution after PS matching). Table 4 provides incidence rates for individual outcomes for patients included in this analysis.

**Table 3:** Characteristics of patients included in the evaluation of comparative outcomes for generic or authorized generic (AG) versus brand initiators after 1:1 propensity score matching in each database.

Characteristic	Optum				MarketScan			
	Generic versus brand-name		AG versus brand-name		Generic versus brand-name		AG versus brand-name	
	Generic initiators	Brand-name initiators	AG initiators	Brand-name initiators	Generic initiators	Brand-name initiators	AG initiators	Brand-name initiators
**Alendronate**								
Number of patients	26,421	26,421	2,428	2,428	112,820	112,820	11,958	11,958
Age: mean (SD)	59 (10)	59 (10)	60 (10)	60 (10)	64.01 (12)	64.25 (12)	63.48 (12)	63.22 (12)
Male sex: *n* (%)	2,525 (9.6%)	2,535 (9.6%)	217 (8.9%)	223 (9.2%)	12,369 (11.0%)	12,545 (11.1%)	1,234 (10.3%)	1,253 (10.5%)
Osteoporosis: *n* (%)	10,342 (39.1%)	10,229 (38.7%)	972 (40.0%)	951 (39.2%)	30,753 (27.3%)	30,929 (27.4%)	3,465 (29.0%)	3,417 (28.6%)
**Amlodipine**								
Number of patients	69,478	69,478	25,259	25,259	32,740	32,740	32,740	32,740
Age: mean (SD)	54 (13)	54 (13)	56 (13)	56 (12)	60.60 (15)	60.64 (15)	60.37 (15)	60.64 (15)
Male sex: *n* (%)	36,359 (52.3%)	36,178 (52.1%)	13,732 (54.4%)	13,754 (54.5%)	14,510 (44.3%)	14,896 (45.5%)	14,816 (45.3%)	14,896 (45.5%)
Hypertension: *n* (%)	51,437 (74.0%)	51,891 (74.7%)	19,303 (76.4%)	19,223 (76.1%)	19,678 (60.1%)	19,530 (59.7%)	19,477 (59.5%)	19,530 (59.7%)
**Amlodipine-benazepril**								
Number of patients	14,704	14,704	5,992	5,992	53,495	53,495	23,158	23,158
Age: mean (SD)	52 (12)	52 (11)	54 (12)	53 (11)	54.65 (13)	54.65 (13)	54.88 (12)	54.65 (13)
Male sex: *n* (%)	8,670 (59.0%)	8,758 (59.6%)	3,408 (56.9%)	3,491 (58.3%)	29,835 (55.8%)	30,014 (56.1%)	12,988 (56.1%)	13,250 (57.2%)
Hypertension: *n* (%)	10,543 (71.7%)	10,453 (71.1%)	4,280 (71.4%)	4,131 (68.9%)	31,951 (59.7%)	31,660 (59.2%)	13,840 (59.8%)	13,584 (58.7%)
**Calcitonin salmon**								
Number of patients	944	944	636	636	6,306	6,306	3,422	3,422
Age: mean (SD)	62 (12)	62 (12)	59 (13)	60 (13)	68.37 (14)	68.42 (14)	66.23 (15)	65.77 (15)
Male sex: *n* (%)	142 (15.0%)	145 (15.4%)	102 (16.0%)	96 (15.1%)	848 (13.4%)	847 (13.4%)	518 (15.1%)	537 (15.7%)
Osteoporosis: *n* (%)	437 (46.3%)	440 (46.6%)	272 (42.8%)	262 (41.2%)	2,227 (35.3%)	2,165 (34.3%)	1,189 (34.7%)	1,196 (35.0%)
**Escitalopram**								
Number of patients	53,711	53,711	25,540	25,540	297,843	297,843	103,016	103,016
Age: mean (SD)	39 (15)	39 (15)	39 (15)	39 (15)	42.58 (17)	42.46 (17)	42.61 (18)	42.20 (17)
Male sex: *n* (%)	17,870 (33.3%)	17,982 (33.5%)	8,790 (34.4%)	8,725 (34.2%)	94,821 (31.8%)	94,674 (31.8%)	32,963 (32.0%)	32,891 (31.9%)
Depression: *n* (%)	5,349 (10.0%)	5,160 (9.6%)	2,593 (10.2%)	2,556 (10.0%)	31,146 (10.5%)	30,921 (10.4%)	10,401 (10.1%)	9,875 (9.6%)
Anxiety: *n* (%)	5,657 (10.5%)	5,383 (10.0%)	2,666 (10.4%)	2,436 (9.5%)	26,017 (8.7%)	26,425 (8.9%)	8,883 (8.6%)	8,420 (8.2%)
**Glipizide**								
Number of patients	997	997	825	825	2,388	2,388	2,246	2,246
Age: mean (SD)	58 (13)	58 (13)	58 (11)	58 (12)	60.98 (14)	61.67 (13)	61.66 (13)	61.79 (13)
Male sex: *n* (%)	539 (54.1%)	506 (50.8%)	452 (54.8%)	440 (53.3%)	1,223 (51.2%)	1,203 (50.4%)	1,124 (50.0%)	1,134 (50.5%)
Diabetes mellitus: *n* (%)	695 (69.7%)	722 (72.4%)	596 (72.2%)	605 (73.3%)	1,539 (64.4%)	1,546 (64.7%)	1,488 (66.3%)	1,495 (66.6%)
**Quinapril**								
Number of patients	4,480	4,480	3,684	3,684	8,049	8,049	9,262	9,262
Age: mean (SD)	53 (12)	53 (13)	54 (12)	54 (12)	56.58 (14)	56.55 (14)	58.48 (14)	58.47 (14)
Male sex: *n* (%)	2,579 (57.6%)	2,599 (58.0%)	2,121 (57.6%)	2,138 (58.0%)	4,163 (51.7%)	4,189 (52.0%)	5,006 (54.0%)	5,039 (54.4%)
Hypertension: *n* (%)	2,694 (60.1%)	2,653 (59.2%)	2,256 (61.2%)	2,242 (60.9%)	3,577 (44.4%)	3,481 (43.2%)	4,218 (45.5%)	4,160 (44.9%)
**Sertraline**								
Number of patients	54,493	54,493	55,674	55,674	132,986	132,986	132,056	132,056
Age: mean (SD)	40 (15)	39 (15)	39 (15)	38 (15)	43.82 (18)	43.71 (18)	42.45 (17)	42.43 (18)
Male sex: *n* (%)	17,110 (31.4%)	16,884 (31.0%)	17,472 (31.4%)	17,303 (31.1%)	41,625 (31.3%)	41,141 (30.9%)	41,584 (31.5%)	41,392 (31.3%)
Depression: *n* (%)	4,985 (9.1%)	4,685 (8.6%)	5,278 (9.5%)	4,887 (8.8%)	9,312 (7.0%)	8,656 (6.5%)	9,919 (7.5%)	9,364 (7.1%)
Anxiety: *n* (%)	4,025 (7.4%)	3,833 (7.0%)	4,323 (7.8%)	3,896 (7.0%)	6,101 (4.6%)	5,761 (4.3%)	6,660 (5.0%)	6,160 (4.7%)

**Table 4:** Outcome incidence rates for generic or authorized generic (AG) versus brand initiators after 1:1 propensity score matching in each database.

Characteristic	Optum				MarketScan			
	Generic versus brand-name		AG versus brand-name		Generic versus brand-name		AG versus brand-name	
	Generic initiators	Brand initiators	AG initiators	Brand initiators	Generic initiators	Brand initiators	AG initiators	Brand initiators
**Alendronate**								
Sample size	26,421	26,421	2,428	2,428	112,820	112,820	11,958	11,958
Total person-years	17,641	11,832	627	1,063	84,427	62,862	3,518	8,417
*n* fracture events	189	148	11	20	1,094	951	55	134
Incidence rate/1,000 py	10.71	12.51	17.53	18.81	12.96	15.13	15.63	15.92
Incidence rate difference (95% CI)	−1.79 (−4.32, 0.73)	Ref	−1.28 (−14.52, 11.96)	Ref	−2.17 (−3.40, −0.94)	Ref	−0.29 (−5.22, 4.65)	Ref
**Amlodipine**								
Sample size	69,478	69,478	25,259	25,259	32,740	32,740	32,740	32,740
Total person-years	51,871	31,604	18,331	11,762	22,040	9,560	23,720	9,565
*n* composite cardiovascular endpoint events	1,163	1,001	420	357	506	310	530	310
Incidence rate/1,000 py	22.42	31.67	22.91	30.35	22.96	32.43	22.34	32.41
Incidence rate difference (95% CI)	−9.25 (−11.60, −6.90)	Ref	−7.44 (−11.28, −3.60)	Ref	−9.47 (−13.59, −5.34)	Ref	−10.07 (−14.14, −5.99)	Ref
**Amlodipine-benazepril**								
Sample size	14,704	14,704	5,992	5,992	53,495	53,495	23,158	23,158
Total person-years	11,735	8,074	3,963	3,168	48,520	35,431	15,222	15,385
*n* composite cardiovascular endpoint events	144	140	63	55	517	486	180	208
Incidence rate/1,000 py	12.27	17.34	15.90	17.36	10.66	13.72	11.83	13.52
Incidence rate difference (95% CI)	−5.07 (−8.57, −1.57)	Ref	−1.46 (−7.50, 4.58)	Ref	−3.06 (−4.59, −1.53)	Ref	−1.69 (−4.22, 0.83)	Ref
**Calcitonin salmon**								
Sample size	944	944	636	636	6,306	6,306	3,422	3,422
Total person-years	308	307	177	183	2,159	2,336	907	1,191
*n* fracture events	17	16	8	10	105	101	56	57
Incidence rate/1,000 py	55.21	52.08	45.18	54.74	48.64	43.23	61.74	47.86
Incidence rate difference (95% CI)	3.12 (−33.48, 39.73)	Ref	−9.56 (−55.72, 36.60)	Ref	5.41 (−7.15, 17.96)	Ref	13.89 (−6.51, 34.28)	Ref
**Escitalopram**								
Sample size	53,711	53,711	25,540	25,540	301,337	301,337	103,010	103,010
Total person-years	14,007	19,281	6,821	9,215	149,419	126,038	31,309	42,636
*n* psych hosp events	674	834	343	396	4,635	4,126	1,178	1,319
Incidence rate/1,000 py	48.12	43.26	50.29	42.97	31.02	32.74	37.63	30.94
Incidence rate difference (95% CI)	4.86 (0.19, 9.54)	Ref	7.31 (0.51, 14.11)	Ref	−1.72 (−3.06, −0.38)	Ref	6.69 (3.97, 9.41)	Ref
**Glipizide**								
Sample size	997	997	825	825	2,334	2,334	2,202	2,202
Total person-years	601	422	525	360	1,474	999	1,808	950
*n* insulin events	29	25	35	18	84	62	91	58
Incidence rate/1,000 py	48.27	59.30	66.62	49.96	56.98	62.07	50.33	61.08
Incidence rate difference (95% CI)	−11.03 (−40.17, 18.10)	Ref	16.65 (−15.28, 48.59)	Ref	−5.08 (−24.76, 14.59)	Ref	−10.75 (−29.57, 8.06)	Ref
**Quinapril**								
Sample size	4,480	4,480	3,684	3,684	8,049	8,049	9,262	9,262
Total person-years	2,113	1,662	2,360	1,424	4,003	3,295	7,325	3,855
*n* composite cardiovascular endpoint events	73	60	55	52	99	97	163	118
Incidence rate/1,000 py	34.54	36.10	23.31	36.53	24.73	29.44	22.25	30.61
Incidence rate difference (95% CI)	−1.56 (−13.65, 10.54)	Ref	−13.22 (−24.90, −1.54)	Ref	−4.71 (−12.32, 2.91)	Ref	−8.36 (−14.85, −1.86)	Ref
**Sertraline**								
Sample size	54,493	54,493	55,674	55,674	132,995	132,995	132,067	132,067
Total person-years	25,322	20,168	23,435	20,465	68,982	55,602	61,082	54,877
*n* psych hosp events	670	569	739	659	1,413	1,245	1,521	1,355
Incidence rate/1,000 py	26.46	28.21	31.53	32.20	20.48	22.39	24.90	24.69
Incidence rate difference (95% CI)	−1.75 (−4.82, 1.31)	Ref	−0.67 (−4.02, 2.68)	Ref	−1.91 (−3.55, −0.27)	Ref	0.21 (−1.61, 2.02)	Ref

py, person-years.

**Fig 3:**
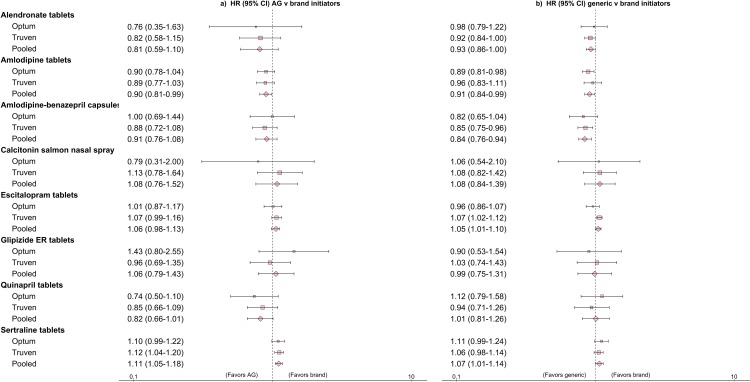
Hazard ratios (HRs) and 95% confidence intervals (CIs) comparing outcomes for authorized generic (AG) versus brand initiators and generic versus brand initiators after 1:1 propensity score matching in each database. The outcome for amlodipine tablets, amlodipine-benazepril capsules, and quinapril tablets was a composite endpoint comprising hospitalizations for myocardial infarction, ischemic stroke, or coronary revascularization procedures. The outcome for alendronate tablets and calcitonin salmon nasal spray was a composite non-vertebral fracture endpoint comprising humerus, wrist, hip, or pelvis fractures. The outcome for escitalopram tablets and sertraline tablets was hospitalization with a psychiatric condition as the principal discharge diagnosis code. The outcome for glipizide extended release (ER) tablets was initiation of insulin during the follow-up period.

S2 Fig summarizes treatment effect estimates for each product before PS matching, and Fig 3 summarizes treatment effect estimates for each product after PS matching. After PS matching, the comparison between generic and brand-name initiators (Fig 3) suggested lower rates of the composite cardiovascular endpoint with the generic version for amlodipine and amlodipine-benazepril (HR [95% CI]: 0.91 [0.84–0.99] and 0.84 [0.76–0.94], respectively). For escitalopram and sertraline, a higher rate of hospitalization with a psychiatric diagnosis was observed among patients initiating generic versions compared to those initiating brand-name versions (HR [95% CI]: 1.05 [1.01–1.10] and 1.07 [1.01–1.14], respectively). Results from the negative control analysis also showed a numerically higher rate of hospitalization with a psychiatric condition among AG initiators compared to brand-name initiators for escitalopram (HR [95% CI]: 1.06 [0.98–1.13]) and a statistically significantly higher rate for sertraline (HR [95% CI]: 1.11 [1.05–1.18]). For alendronate, calcitonin, glipizide, and quinapril, no differences were observed in the outcomes of interest between generic and brand-name initiators.

## Discussion

In this study conducted using 2 large US commercial insurance databases, we used AGs—generic versions of brand-name products that are chemically identical and identical in appearance to the branded product—to account for potential bias due to negative perceptions towards generics in comparing the effectiveness of brand-name versus generic products. We observed equivalent or better clinical outcomes among patients who used generic versus AG products in 15 out of 16 comparisons across 8 drug products. When directly comparing individuals who initiated treatment with generic versus brand-name products, we used AGs versus brand-name products as a negative control comparison. For the 2 drugs for which we observed higher rates of hospitalization with a psychiatric condition with the generic version compared to the brand-name version—escitalopram and sertraline—the negative control comparisons also tended towards higher rates with AG use compared to brand-name use, suggesting that the differences observed between brand-name and generic users in these outcomes were likely explained by residual confounding or generic perception bias.

Our results add to a growing body of literature supporting the clinical equivalence of brand-name products and FDA-approved bioequivalent generic versions [2,3,17,28,29]. With 2 large national databases and a PS-matched design, we aimed to address the possibility of perception bias affecting comparative effectiveness estimates in observational studies of generic drugs by considering AGs as a control group. We found largely consistent results across 8 drug products and 4 therapeutic classes. In the comparison involving patients switching from amlodipine brand-name to AG or generic, in which we found a lower rate of the composite cardiovascular endpoint with AG use compared to generic use, the effect size was modest (HR of 0.92 with upper 95% confidence limit of 0.97) and should be interpreted cautiously given the multiple comparisons that were undertaken. We did not correct for multiple testing to control type I error because this would increase type II error and potentially result in a missed signal [30]. Therefore, type I error might explain the statistically significant findings in the current study.

Although use of generic medications has increased rapidly in the past 2 decades, in recent national surveys about one-third of patients, physicians, and pharmacists were still classified as skeptics about the safety, effectiveness, and quality of generic medications [9,11]. Negative perceptions may lead patients to switch back to the brand-name product after generic substitution. Indeed, switching back to the brand-name product after a brand-name to generic switch is highly prevalent. In a previous study of the 8 drug products included in this study, we found that switching back to the brand-name product occurred at a rate of 8.9 per 100 person-years after a switch to a generic version, and a rate of 7.4 per 100 person-years after a switch to AG [31]. Switching back to the brand-name product when less expensive generic alternatives are available can result in unnecessary costs to patients and the healthcare system. Use of low-cost generic alternatives in place of brand-name drugs saved the US approximately $1 trillion over the last decade [32]. Results from our comparative effectiveness investigation suggest that clinical outcomes with use of generics and brand products for serious chronic conditions such as diabetes, hypertension, osteoporosis, and depression and anxiety are broadly equivalent. These results may inform clinical practice by guiding the development of educational interventions to address physicians’ and patients’ negative perceptions of generics and to increase awareness regarding the equivalence of generic and brand-name drugs. For instance, results from the current and previous studies [2,3,17,28,29] could be used to develop educational materials for clinical outreach initiatives such as academic detailing, where trained clinical educators including physicians, nurses, and pharmacists present concise summaries of the evidence demonstrating brand and generic equivalence to practitioners in their offices [33]. Similar educational interventions targeted at patients delivered by trained pharmacy personnel could increase perseverance on generic medications and reduce the rate of patients switching back to the brand-name products.

The most important strength of this study is the use of AGs as a control exposure to indirectly account for perception biases that are not recorded in any data sources. Another major strength is inclusion of patients from 2 large data sources, which provided large sample sizes for most analyses. Further, rigorous confounding control was made possible by the availability of comprehensive longitudinal insurance claims data in both sources. Finally, inclusion of 8 different drugs from 4 therapeutic classes ensures generalizability of our results across multiple therapeutic classes.

However, our study also has several limitations. First, the data were sourced from 2 commercial health insurance databases from the US that do not collect information about clinical parameters such as cholesterol or bone mineral density, introducing the possibility of residual confounding. For instance, if the brand version of alendronate is preferentially used in patients with low bone mineral density due to a perception of higher effectiveness, then residual confounding by this factor could bias the results in favor of the generic version. We sought to address this limitation by including a large number of measured confounding variables in our PS models, many of which are likely to be correlated with unmeasured factors. Second, no narrow therapeutic index drugs—which have been the subject of particular concern regarding generic safety and effectiveness—met the criteria for inclusion in this study [10]. Therefore our results may not generalize to these types of drugs, and future research comparing the effectiveness and safety of generic versus brand versions of narrow therapeutic index drugs is recommended. Third, we were not able to examine surrogate outcome measures, such as blood pressure changes or changes in lipid levels, as outcomes since the data sources do not contain this information. It is possible that slight differences in the bioavailability of different versions of the same drug could lead to small changes in laboratory values. Changes in laboratory results or other surrogate measures after switching to generics from brand-name drugs may be important to identify and characterize in future research. For instance, immediate changes in blood pressure control after switching from brand to generic amlodipine, perhaps due to the nocebo effect, could lead to compromised patient compliance. Therefore, products for which such changes are common should be identified and prioritized for patient counseling by pharmacists or physicians.

## Conclusion

In this study of 8 drug products conducted using 2 large US commercial insurance databases, we observed that use of generics provided comparable clinical outcomes as the brand products. These results could be used in educational interventions aimed at increasing patient and physician confidence in the ability of generic medicines to manage chronic diseases.

## Supporting information

S1 Appendix Supplementary tables. (PDF)

S1 Fig Hazard ratios (HRs) and 95% confidence intervals (CIs) comparing clinical outcomes for patients initiating authorized generics (AGs) versus generics and patients switching from brand-name to AGs versus generics before 1:1 propensity score matching in each database. The clinical outcome for amlodipine tablets, amlodipine-benazepril capsules, and quinapril tablets was a composite endpoint comprising hospitalization for myocardial infarction, ischemic stroke, or coronary revascularization procedures. The outcome for alendronate tablets and calcitonin salmon nasal spray was a composite non-vertebral fracture endpoint comprising humerus, wrist, hip, or pelvis fractures. The outcome for escitalopram tablets and sertraline tablets was hospitalization with a psychiatric condition as the principal discharge diagnosis code. The outcome for glipizide extended release (ER) tablets was initiation of insulin during the follow-up period.
(PDF)

S2 Fig Hazard ratios (HRs) and 95% confidence intervals (CIs) comparing clinical outcomes for authorized generic (AG) versus brand initiators and generic versus brand initiators before 1:1 propensity score matching in each database. The outcome for amlodipine tablets, amlodipine-benazepril capsules, and quinapril tablets was a composite endpoint comprising hospitalization for myocardial infarction, ischemic stroke, or coronary revascularization procedures. The outcome for alendronate tablets and calcitonin salmon nasal spray was a composite non-vertebral fracture endpoint comprising humerus, wrist, hip, or pelvis fractures. The outcome for escitalopram tablets and sertraline tablets was hospitalization with a psychiatric condition as the principal discharge diagnosis code. The outcome for glipizide extended release (ER) tablets was initiation of insulin during the follow-up period.
(PDF)

S1 STROBE checklist Strengthening the reporting of observational studies in epidemiology (STROBE) checklist. (PDF)

S1 Text Prospective analysis plan. Funding proposal submitted to the US Food and Drug Administration.
(PDF)

## Abbreviations

AG: authorized generic
CI: confidence interval
ER: extended release
FDA: US Food and Drug Administration
HR: hazard ratio
NDC: National Drug Code
PS: propensity score
