# Supplementary material for: Comparative effectiveness of generic and brand-name medication use: A database study of US health insurance claims
Source: PLoS Med. 2019 Mar 13;16(3):e1002763. doi: 10.1371/journal.pmed.1002763 (PMC6415809; doi:10.1371/journal.pmed.1002763)
Supplement: S1 Appendix — (PDF) [file pmed.1002763.s001.pdf]

Table of contents

Table A: Outcome identification algorithms.

Table B- Characteristics of patients included in the evaluation of comparative outcomes for *alendronate* between authorized generic (AG) and generic initiators and switchers before propensity score matching in each database

Table C- Characteristics of patients included in the evaluation of comparative outcomes for *amlodipine* between authorized generic (AG) and generic initiators and switchers before propensity score matching in each database

Table D- Characteristics of patients included in the evaluation of comparative outcomes for *amlodipine-benazepril* between authorized generic (AG) and generic initiators and switchers before propensity score matching in each database

Table E- Characteristics of patients included in the evaluation of comparative outcomes for *calcitonin salmon* between authorized generic (AG) and generic initiators and switchers before propensity score matching in each database

Table F- Characteristics of patients included in the evaluation of comparative outcomes for *escitalopram* between authorized generic (AG) and generic initiators and switchers before propensity score matching in each database

Table G- Characteristics of patients included in the evaluation of comparative outcomes for *glipizide* between authorized generic (AG) and generic initiators and switchers before propensity score matching in each database

Table H- Characteristics of patients included in the evaluation of comparative outcomes for *quinapril* between authorized generic (AG) and generic initiators and switchers before propensity score matching in each database

Table I- Characteristics of patients included in the evaluation of comparative outcomes for *sertraline* between authorized generic (AG) and generic initiators and switchers before propensity score matching in each database

Table J- Characteristics of patients included in the evaluation of comparative outcomes for *alendronate* between authorized generic (AG) and generic initiators and switchers after 1:1 propensity score matching in each database

Table K- Characteristics of patients included in the evaluation of comparative outcomes for *amlodipine* between authorized generic (AG) and generic initiators and switchers after 1:1 propensity score matching in each database

Table L- Characteristics of patients included in the evaluation of comparative outcomes for *amlodipine-benazepril* between authorized generic (AG) and generic initiators and switchers after 1:1 propensity score matching in each database

Table M- Characteristics of patients included in the evaluation of comparative outcomes for *calcitonin salmon* between authorized generic (AG) and generic initiators and switchers after 1:1 propensity score matching in each database

Table N- Characteristics of patients included in the evaluation of comparative outcomes for *escitalopram* between authorized generic (AG) and generic initiators and switchers after 1:1 propensity score matching in each database

Table O- Characteristics of patients included in the evaluation of comparative outcomes for *glipizide* between authorized generic (AG) and generic initiators and switchers after 1:1 propensity score matching in each database

Table P- Characteristics of patients included in the evaluation of comparative outcomes for *quinapril* between authorized generic (AG) and generic initiators and switchers after 1:1 propensity score matching in each database

Table Q- Characteristics of patients included in the evaluation of comparative outcomes for *sertraline* between authorized generic (AG) and generic initiators and switchers after 1:1 propensity score matching in each database

Table R- Characteristics of patients included in the evaluation of comparative outcomes for *alendronate* between generic or authorized generics (AG) and brand initiators before propensity score matching in each database

Table S- Characteristics of patients included in the evaluation of comparative outcomes for *amlodipine* between generic or authorized generics (AG) and brand initiators before propensity score matching in each database

Table T- Characteristics of patients included in the evaluation of comparative outcomes for *amlodipine-benazepril* between generic or authorized generics (AG) and brand initiators before propensity score matching in each database

Table U- Characteristics of patients included in the evaluation of comparative outcomes for *calcitonin* between generic or authorized generics (AG) and brand initiators before propensity score matching in each database

Table V- Characteristics of patients included in the evaluation of comparative outcomes for *escitalopram* between generic or authorized generics (AG) and brand initiators before propensity score matching in each database

Table W- Characteristics of patients included in the evaluation of comparative outcomes for *glipizide* between generic or authorized generics (AG) and brand initiators before propensity score matching in each database

Table X- Characteristics of patients included in the evaluation of comparative outcomes for *quinapril* between generic or authorized generics (AG) and brand initiators before propensity score matching in each database

Table Y- Characteristics of patients included in the evaluation of comparative outcomes for *sertraline* between generic or authorized generics (AG) and brand initiators before propensity score matching in each database

Table Z- Characteristics of patients included in the evaluation of comparative outcomes for *alendronate* between generic or authorized generics (AG) and brand initiators after 1:1 propensity score matching in each database

Table AA- Characteristics of patients included in the evaluation of comparative outcomes for *amlodipine* between generic or authorized generics (AG) and brand initiators after 1:1 propensity score matching in each database

Table AB- Characteristics of patients included in the evaluation of comparative outcomes for *amlodipine-benazepril* between generic or authorized generics (AG) and brand initiators after 1:1 propensity score matching in each database

Table AC- Characteristics of patients included in the evaluation of comparative outcomes for *calcitonin* between generic or authorized generics (AG) and brand initiators after 1:1 propensity score matching in each database

Table AD- Characteristics of patients included in the evaluation of comparative outcomes for *escitalopram* between generic or authorized generics (AG) and brand initiators after 1:1 propensity score matching in each database

Table AE- Characteristics of patients included in the evaluation of comparative outcomes for *glipizide* between generic or authorized generics (AG) and brand initiators after 1:1 propensity score matching in each database

Table AF- Characteristics of patients included in the evaluation of comparative outcomes for *quinapril* between generic or authorized generics (AG) and brand initiators after 1:1 propensity score matching in each database

Table AG- Characteristics of patients included in the evaluation of comparative outcomes for *sertraline* between generic or authorized generics (AG) and brand initiators after 1:1 propensity score matching in each database

Table A: Outcome identification algorithms

Outcome	Definition
Composite non-vertebral facture (for alendronate and calcitonin)	Humerus fracture diagnosis (ICD-9: 812.xx, 733.11) AND procedure within 30 days of fracture date (ICD-9: 78.52, 79.01, 79.11, 79.21, 79.31, 79.61; CPT-4: 23600, 23605, 23610, 23615, 23620, 23625, 23630, 23665, 23670, 23680, 24500, 24505, 24506, 24510, 24515, 24530, 24531, 24535, 24536, 24538, 24540, 24542, 24545, 24560, 24565, 24570, 24575, 24581, 24583, 24585-8, 24516) OR Radius/ulna fracture diagnosis (ICD-9: 813.xx, 733.12) AND procedure within 30 days of fracture date (ICD-9: 78.53, 79.02, 79.12, 79.22, 79.32, 79.62; CPT-4: 24620, 24625, 24635, 24650, 24655, 24660, 24665-6, 24670, 24680, 24685, 25500, 25505, 25510, 25515, 25530, 25535, 25540, 25545, 25560, 25565, 25570, 25575, 25600, 25605, 25610-1, 25615, 25620, 25650) OR Pelvis fracture diagnosis (ICD-9: 808.xx) OR Hip fracture diagnosis (ICD-9 code: 820.xx,733.14) during hospitalization AND procedure code during hospitalization (ICD-9: 78.55, 79.05, 79.15, 79.25, 79.35, 79.65; CPT-4: 27230-27248)
Composite cardiovascular outcome (for amlodipine, amlodipine-benazepril, quinapril)	Myocardial infraction defined as a hospitalization episode with ICD 410.01, 410.11, 410.21, 410.31, 410.41, 410.51, 410.61, 410.71, 410.81, or 410.91 listed in principal or secondary position OR stroke defined as ICD-9-CMs in the principal position in a hospital claim: 433.X1, 434.x1, 435.x, 436, 437.1x, 437.9x OR Coronary revascularization defined as any of the following procedure codes: CPT 92974, 92980, 92981, 92982, 92984, 92995, 92996, 33510-33519, 33521, 33522, 33523, 33530, 33533-33536, ICD procedure codes; 36.1, 36.2
Psychiatric hospitalization endpoint (for sertraline and escitalopram)	An inpatient stay with ICD-9-CM codes 290.xx-316.xx as primary diagnoses
Treatment intensification (for glipizide)	Dispending of a new insulin prescription

Table B- Characteristics of patients included in the evaluation of comparative outcomes for *alendronate* between authorized generic (AG) and generic initiators and switchers before propensity score matching in each database

	Optum				Truven			
	AG initiators	Generic initiators	AG switchers	Generic switchers	AG initiators	Generic initiators	AG switchers	Generic switchers
Cohort selection steps								
Total number of patients filling prescriptions for the version of interest after 6-month continuous enrollment	476,857		668,221		2,055,563		3,425,397	
Patients meeting new drug use restriction	379,901		192,321		2,022,728		862,070	
Patients meeting prior brand-name use requirement (to define switchers, applicable for switchers comparison only)	NA		87,990		N/A		324,079	
Total eligible by exposure group	73,973	305,928	41,217	46,773	474,066	1,548,662	192,720	131,359
Baseline characteristics								
Age: mean (sd)	55.32 (12.37)	53.20 (11.94)	59.50 (11.20)	55.71 (11.19)	60.50 (14.33)	57.68 (14.31)	67.10 (12.64)	61.55 (13.85)
Gender								
MALE: n (%)	41,444 (56.0%)	167,735 (54.8%)	22,326 (54.2%)	24,824 (53.1%)	237,280 (50.1%)	781,087 (50.4%)	91,202 (47.3%)	63,184 (48.1%)
FEMALE: n (%)	32,524 (44.0%)	138,177 (45.2%)	18,887 (45.8%)	21,945 (46.9%)	236,786 (49.9%)	767,575 (49.6%)	101,518 (52.7%)	68,175 (51.9%)
UNKNOWN: n (%)	5 (0.0%)	16 (0.0%)	4 (0.0%)	4 (0.0%)	0 (0.0%)	0 (0.0%)	0 (0.0%)	0 (0.0%)
Region								
Northeast: n (%)	5,649 (7.6%)	26,295 (8.6%)	4,492 (10.9%)	5,822 (12.4%)	58,444 (12.3%)	274,518 (17.7%)	20,808 (10.8%)	16,036 (12.2%)
Midwest: n (%)	12,032 (16.3%)	75,751 (24.8%)	9,853 (23.9%)	15,191 (32.5%)	86,982 (18.3%)	404,515 (26.1%)	75,186 (39.0%)	38,204 (29.1%)
South: n (%)	43,869 (59.3%)	168,589 (55.1%)	19,129 (46.4%)	21,667 (46.3%)	189,519 (40.0%)	658,259 (42.5%)	69,916 (36.3%)	62,494 (47.6%)
West: n (%)	12,380 (16.7%)	35,112 (11.5%)	7,721 (18.7%)	4,051 (8.7%)	134,745 (28.4%)	175,643 (11.3%)	26,211 (13.6%)	14,065 (10.7%)
Unknown/other: n (%)	43 (0.1%)	181 (0.1%)	22 (0.1%)	42 (0.1%)	4,376 (0.9%)	35,727 (2.3%)	599 (0.3%)	560 (0.4%)
Hyperlipidemia: n (%)	33,015 (44.6%)	124,770 (40.8%)	19,307 (46.8%)	20,051 (42.9%)	149,235 (31.5%)	504,309 (32.6%)	40,109 (20.8%)	31,922 (24.3%)
Diabetes mellitus: n (%)	15,101 (20.4%)	60,122 (19.7%)	8,887 (21.6%)	10,031 (21.4%)	98,037 (20.7%)	310,530 (20.1%)	39,632 (20.6%)	26,174 (19.9%)
Hypertension: n (%)	54,407 (73.5%)	224,354 (73.3%)	29,185 (70.8%)	33,788 (72.2%)	298,724 (63.0%)	1,022,164 (66.0%)	97,163 (50.4%)	72,042 (54.8%)

Myocardial Infarction: n (%)	1,002 (1.4%)	4,331 (1.4%)	228 (0.6%)	337 (0.7%)	6,598 (1.4%)	25,003 (1.6%)	1,053 (0.5%)	886 (0.7%)
Angina: n (%)	2,698 (3.6%)	10,461 (3.4%)	1,152 (2.8%)	1,338 (2.9%)	16,354 (3.4%)	52,708 (3.4%)	5,085 (2.6%)	3,195 (2.4%)
Heart failure: n (%)	2,618 (3.5%)	10,463 (3.4%)	1,270 (3.1%)	1,424 (3.0%)	19,603 (4.1%)	66,415 (4.3%)	6,572 (3.4%)	4,621 (3.5%)
Other forms of chronic heart diseases: n (%)	7,769 (10.5%)	27,971 (9.1%)	5,673 (13.8%)	5,324 (11.4%)	51,192 (10.8%)	161,979 (10.5%)	26,672 (13.8%)	14,506 (11.0%)
Cerebrovascular accident: n (%)	2,131 (2.9%)	8,894 (2.9%)	798 (1.9%)	863 (1.8%)	15,845 (3.3%)	56,778 (3.7%)	3,935 (2.0%)	2,759 (2.1%)
Venous thromboembolism: n (%)	1,137 (1.5%)	4,736 (1.5%)	433 (1.1%)	541 (1.2%)	7,474 (1.6%)	27,600 (1.8%)	2,143 (1.1%)	1,541 (1.2%)
Atrial fibrillation: n (%)	2,401 (3.2%)	8,498 (2.8%)	1,514 (3.7%)	1,263 (2.7%)	20,032 (4.2%)	62,249 (4.0%)	8,110 (4.2%)	4,333 (3.3%)
Overweight or obese: n (%)	5,427 (7.3%)	25,643 (8.4%)	1,676 (4.1%)	2,475 (5.3%)	25,889 (5.5%)	102,563 (6.6%)	1,302 (0.7%)	2,034 (1.5%)
Tobacco use: n (%)	3,902 (5.3%)	19,717 (6.4%)	1,181 (2.9%)	1,946 (4.2%)	17,176 (3.6%)	73,918 (4.8%)	1,086 (0.6%)	1,546 (1.2%)
Alcohol abuse or dependence: n (%)	846 (1.1%)	4,153 (1.4%)	235 (0.6%)	369 (0.8%)	4,176 (0.9%)	16,746 (1.1%)	406 (0.2%)	456 (0.3%)
Renal disease: n (%)	2,580 (3.5%)	10,862 (3.6%)	1,163 (2.8%)	1,476 (3.2%)	19,430 (4.1%)	57,805 (3.7%)	3,628 (1.9%)	3,167 (2.4%)
Liver disease: n (%)	2,338 (3.2%)	9,356 (3.1%)	931 (2.3%)	1,184 (2.5%)	11,369 (2.4%)	39,911 (2.6%)	2,424 (1.3%)	2,051 (1.6%)
Aspirin: n (%)	36 (0.0%)	268 (0.1%)	25 (0.1%)	57 (0.1%)	3,874 (0.8%)	10,612 (0.7%)	1,799 (0.9%)	1,074 (0.8%)
Antiplatelets: n (%)	3,150 (4.3%)	11,251 (3.7%)	2,824 (6.9%)	2,836 (6.1%)	24,768 (5.2%)	72,561 (4.7%)	19,653 (10.2%)	10,470 (8.0%)
Statins: n (%)	24,171 (32.7%)	84,710 (27.7%)	18,520 (44.9%)	17,203 (36.8%)	169,112 (35.7%)	470,673 (30.4%)	94,135 (48.8%)	53,333 (40.6%)
Other lipid lowering agents: n (%)	6,283 (8.5%)	19,772 (6.5%)	6,935 (16.8%)	6,349 (13.6%)	37,401 (7.9%)	96,185 (6.2%)	33,486 (17.4%)	19,039 (14.5%)
Insulin preparations: n (%)	3,102 (4.2%)	12,471 (4.1%)	1,658 (4.0%)	1,893 (4.0%)	13,511 (2.9%)	45,972 (3.0%)	5,126 (2.7%)	3,766 (2.9%)
Oral hypoglycemic agents: n (%)	10,028 (13.6%)	38,667 (12.6%)	7,057 (17.1%)	7,737 (16.5%)	72,752 (15.3%)	209,927 (13.6%)	37,487 (19.5%)	23,574 (17.9%)
ACE inhibitors: n (%)	25,825 (34.9%)	107,122 (35.0%)	12,571 (30.5%)	14,497 (31.0%)	167,119 (35.3%)	523,498 (33.8%)	57,896 (30.0%)	39,125 (29.8%)
ARBs: n (%)	17,933 (24.2%)	63,418 (20.7%)	12,029 (29.2%)	12,139 (26.0%)	112,388 (23.7%)	336,929 (21.8%)	57,564 (29.9%)	35,873 (27.3%)
Calcium channel blockers (without amlodipine): n (%)	4,194 (5.7%)	14,960 (4.9%)	577 (1.4%)	793 (1.7%)	40,256 (8.5%)	79,107 (5.1%)	2,989 (1.6%)	2,639 (2.0%)
Diuretics: n (%)	27,166 (36.7%)	111,271 (36.4%)	20,144 (48.9%)	23,061 (49.3%)	187,790 (39.6%)	579,414 (37.4%)	98,748 (51.2%)	66,291 (50.5%)
Beta-blockers: n (%)	17,575 (23.8%)	70,117 (22.9%)	14,269 (34.6%)	15,746 (33.7%)	137,627 (29.0%)	401,544 (25.9%)	76,129 (39.5%)	47,043 (35.8%)
Anticoagulants: n (%)	2,075 (2.8%)	7,687 (2.5%)	1,837 (4.5%)	1,704 (3.6%)	18,745 (4.0%)	56,609 (3.7%)	12,378 (6.4%)	6,768 (5.2%)
NSAIDs: n (%)	10,537 (14.2%)	44,513 (14.6%)	5,480 (13.3%)	6,520 (13.9%)	68,858 (14.5%)	230,798 (14.9%)	27,006 (14.0%)	18,958 (14.4%)
Coxibs: n (%)	1,450 (2.0%)	4,410 (1.4%)	1,431 (3.5%)	1,144 (2.4%)	9,122 (1.9%)	25,344 (1.6%)	8,635 (4.5%)	4,209 (3.2%)

Outpatient visits: mean (sd)	0.15 (0.42)	0.17 (0.48)	0.12 (0.39)	0.13 (0.38)	0.11 (0.35)	0.14 (0.39)	0.07 (0.27)	0.09 (0.31)
Inpatient hospitalization: mean (sd)	0.01 (0.16)	0.01 (0.15)	0.01 (0.12)	0.01 (0.11)	0.00 (0.02)	0.00 (0.01)	0.00 (0.01)	0.00 (0.01)
ED visit: mean (sd)	0.28 (0.79)	0.32 (0.89)	0.14 (0.53)	0.19 (0.76)	0.36 (1.10)	0.44 (1.23)	0.19 (0.65)	0.24 (0.85)
Number of distinct medication prescriptions: mean (sd)	5.52 (4.63)	5.20 (4.55)	7.44 (4.51)	7.11 (4.57)	6.03 (4.85)	5.67 (4.80)	8.31 (4.77)	7.83 (4.86)
Combined Comorbidity Score: mean (sd)	0.13 (1.63)	0.11 (1.61)	0.01 (1.38)	-0.01 (1.42)	0.25 (1.63)	0.25 (1.68)	0.16 (1.29)	0.11 (1.35)

Table C- Characteristics of patients included in the evaluation of comparative outcomes for *amlodipine* between authorized generic (AG) and generic initiators and switchers before propensity score matching in each database

	Optum				Truven			
	AG initiators	Generic initiators	AG switchers	Generic switchers	AG initiators	Generic initiators	AG switchers	Generic switchers
Cohort selection steps								
Total number of patients filling prescriptions for the version of interest after 6-month continuous enrollment	476,857		668,221		2,055,563		3,425,397	
Patients meeting new drug use restriction	379,901		192,321		2,022,728		862,070	
Patients meeting prior brand-name use requirement (to define switchers, applicable for switchers comparison only)	NA		87,990		N/A		324,079	
Total eligible by exposure group	73,973	305,928	41,217	46,773	474,066	1,548,662	192,720	131,359
Baseline characteristics								
Age: mean (sd)	55.32 (12.37)	53.20 (11.94)	59.50 (11.20)	55.71 (11.19)	60.50 (14.33)	57.68 (14.31)	67.10 (12.64)	61.55 (13.85)
Gender								
MALE: n (%)	41,444 (56.0%)	167,735 (54.8%)	22,326 (54.2%)	24,824 (53.1%)	237,280 (50.1%)	781,087 (50.4%)	91,202 (47.3%)	63,184 (48.1%)
FEMALE: n (%)	32,524 (44.0%)	138,177 (45.2%)	18,887 (45.8%)	21,945 (46.9%)	236,786 (49.9%)	767,575 (49.6%)	101,518 (52.7%)	68,175 (51.9%)
UNKNOWN: n (%)	5 (0.0%)	16 (0.0%)	4 (0.0%)	4 (0.0%)	0 (0.0%)	0 (0.0%)	0 (0.0%)	0 (0.0%)
Region								
Northeast: n (%)	5,649 (7.6%)	26,295 (8.6%)	4,492 (10.9%)	5,822 (12.4%)	58,444 (12.3%)	274,518 (17.7%)	20,808 (10.8%)	16,036 (12.2%)
Midwest: n (%)	12,032 (16.3%)	75,751 (24.8%)	9,853 (23.9%)	15,191 (32.5%)	86,982 (18.3%)	404,515 (26.1%)	75,186 (39.0%)	38,204 (29.1%)
South: n (%)	43,869 (59.3%)	168,589 (55.1%)	19,129 (46.4%)	21,667 (46.3%)	189,519 (40.0%)	658,259 (42.5%)	69,916 (36.3%)	62,494 (47.6%)
West: n (%)	12,380 (16.7%)	35,112 (11.5%)	7,721 (18.7%)	4,051 (8.7%)	134,745 (28.4%)	175,643 (11.3%)	26,211 (13.6%)	14,065 (10.7%)
Unknown/other: n (%)	43 (0.1%)	181 (0.1%)	22 (0.1%)	42 (0.1%)	4,376 (0.9%)	35,727 (2.3%)	599 (0.3%)	560 (0.4%)
Hyperlipidemia: n (%)	33,015 (44.6%)	124,770 (40.8%)	19,307 (46.8%)	20,051 (42.9%)	149,235 (31.5%)	504,309 (32.6%)	40,109 (20.8%)	31,922 (24.3%)
Diabetes mellitus: n (%)	15,101 (20.4%)	60,122 (19.7%)	8,887 (21.6%)	10,031 (21.4%)	98,037 (20.7%)	310,530 (20.1%)	39,632 (20.6%)	26,174 (19.9%)
Hypertension: n (%)	54,407 (73.5%)	224,354 (73.3%)	29,185 (70.8%)	33,788 (72.2%)	298,724 (63.0%)	1,022,164 (66.0%)	97,163 (50.4%)	72,042 (54.8%)
Myocardial Infarction: n (%)	1,002 (1.4%)	4,331 (1.4%)	228 (0.6%)	337 (0.7%)	6,598 (1.4%)	25,003	1,053 (0.5%)	886 (0.7%)

						(1.6%)		
Angina: n (%)	2,698 (3.6%)	10,461 (3.4%)	1,152 (2.8%)	1,338 (2.9%)	16,354 (3.4%)	52,708 (3.4%)	5,085 (2.6%)	3,195 (2.4%)
Heart failure: n (%)	2,618 (3.5%)	10,463 (3.4%)	1,270 (3.1%)	1,424 (3.0%)	19,603 (4.1%)	66,415 (4.3%)	6,572 (3.4%)	4,621 (3.5%)
Other forms of chronic heart diseases: n (%)	7,769 (10.5%)	27,971 (9.1%)	5,673 (13.8%)	5,324 (11.4%)	51,192 (10.8%)	161,979 (10.5%)	26,672 (13.8%)	14,506 (11.0%)
Cerebrovascular accident: n (%)	2,131 (2.9%)	8,894 (2.9%)	798 (1.9%)	863 (1.8%)	15,845 (3.3%)	56,778 (3.7%)	3,935 (2.0%)	2,759 (2.1%)
Venous thromboembolism: n (%)	1,137 (1.5%)	4,736 (1.5%)	433 (1.1%)	541 (1.2%)	7,474 (1.6%)	27,600 (1.8%)	2,143 (1.1%)	1,541 (1.2%)
Atrial fibrillation: n (%)	2,401 (3.2%)	8,498 (2.8%)	1,514 (3.7%)	1,263 (2.7%)	20,032 (4.2%)	62,249 (4.0%)	8,110 (4.2%)	4,333 (3.3%)
Overweight or obese: n (%)	5,427 (7.3%)	25,643 (8.4%)	1,676 (4.1%)	2,475 (5.3%)	25,889 (5.5%)	102,563 (6.6%)	1,302 (0.7%)	2,034 (1.5%)
Tobacco use: n (%)	3,902 (5.3%)	19,717 (6.4%)	1,181 (2.9%)	1,946 (4.2%)	17,176 (3.6%)	73,918 (4.8%)	1,086 (0.6%)	1,546 (1.2%)
Alcohol abuse or dependence: n (%)	846 (1.1%)	4,153 (1.4%)	235 (0.6%)	369 (0.8%)	4,176 (0.9%)	16,746 (1.1%)	406 (0.2%)	456 (0.3%)
Renal disease: n (%)	2,580 (3.5%)	10,862 (3.6%)	1,163 (2.8%)	1,476 (3.2%)	19,430 (4.1%)	57,805 (3.7%)	3,628 (1.9%)	3,167 (2.4%)
Liver disease: n (%)	2,338 (3.2%)	9,356 (3.1%)	931 (2.3%)	1,184 (2.5%)	11,369 (2.4%)	39,911 (2.6%)	2,424 (1.3%)	2,051 (1.6%)
Aspirin: n (%)	36 (0.0%)	268 (0.1%)	25 (0.1%)	57 (0.1%)	3,874 (0.8%)	10,612 (0.7%)	1,799 (0.9%)	1,074 (0.8%)
Antiplatelets: n (%)	3,150 (4.3%)	11,251 (3.7%)	2,824 (6.9%)	2,836 (6.1%)	24,768 (5.2%)	72,561 (4.7%)	19,653 (10.2%)	10,470 (8.0%)
Statins: n (%)	24,171 (32.7%)	84,710 (27.7%)	18,520 (44.9%)	17,203 (36.8%)	169,112 (35.7%)	470,673 (30.4%)	94,135 (48.8%)	53,333 (40.6%)
Other lipid lowering agents: n (%)	6,283 (8.5%)	19,772 (6.5%)	6,935 (16.8%)	6,349 (13.6%)	37,401 (7.9%)	96,185 (6.2%)	33,486 (17.4%)	19,039 (14.5%)
Insulin preparations: n (%)	3,102 (4.2%)	12,471 (4.1%)	1,658 (4.0%)	1,893 (4.0%)	13,511 (2.9%)	45,972 (3.0%)	5,126 (2.7%)	3,766 (2.9%)
Oral hypoglycemic agents: n (%)	10,028 (13.6%)	38,667 (12.6%)	7,057 (17.1%)	7,737 (16.5%)	72,752 (15.3%)	209,927 (13.6%)	37,487 (19.5%)	23,574 (17.9%)
ACE inhibitors: n (%)	25,825 (34.9%)	107,122 (35.0%)	12,571 (30.5%)	14,497 (31.0%)	167,119 (35.3%)	523,498 (33.8%)	57,896 (30.0%)	39,125 (29.8%)
ARBs: n (%)	17,933 (24.2%)	63,418 (20.7%)	12,029 (29.2%)	12,139 (26.0%)	112,388 (23.7%)	336,929 (21.8%)	57,564 (29.9%)	35,873 (27.3%)
Calcium channel blockers (without amlodipine): n (%)	4,194 (5.7%)	14,960 (4.9%)	577 (1.4%)	793 (1.7%)	40,256 (8.5%)	79,107 (5.1%)	2,989 (1.6%)	2,639 (2.0%)
Diuretics: n (%)	27,166 (36.7%)	111,271 (36.4%)	20,144 (48.9%)	23,061 (49.3%)	187,790 (39.6%)	579,414 (37.4%)	98,748 (51.2%)	66,291 (50.5%)

Beta-blockers: n (%)	17,575 (23.8%)	70,117 (22.9%)	14,269 (34.6%)	15,746 (33.7%)	137,627 (29.0%)	401,544 (25.9%)	76,129 (39.5%)	47,043 (35.8%)
Anticoagulants: n (%)	2,075 (2.8%)	7,687 (2.5%)	1,837 (4.5%)	1,704 (3.6%)	18,745 (4.0%)	56,609 (3.7%)	12,378 (6.4%)	6,768 (5.2%)
NSAIDs: n (%)	10,537 (14.2%)	44,513 (14.6%)	5,480 (13.3%)	6,520 (13.9%)	68,858 (14.5%)	230,798 (14.9%)	27,006 (14.0%)	18,958 (14.4%)
Coxibs: n (%)	1,450 (2.0%)	4,410 (1.4%)	1,431 (3.5%)	1,144 (2.4%)	9,122 (1.9%)	25,344 (1.6%)	8,635 (4.5%)	4,209 (3.2%)
Outpatient visits: mean (sd)	0.15 (0.42)	0.17 (0.48)	0.12 (0.39)	0.13 (0.38)	0.11 (0.35)	0.14 (0.39)	0.07 (0.27)	0.09 (0.31)
Inpatient hospitalization: mean (sd)	0.01 (0.16)	0.01 (0.15)	0.01 (0.12)	0.01 (0.11)	0.00 (0.02)	0.00 (0.01)	0.00 (0.01)	0.00 (0.01)
ED visit: mean (sd)	0.28 (0.79)	0.32 (0.89)	0.14 (0.53)	0.19 (0.76)	0.36 (1.10)	0.44 (1.23)	0.19 (0.65)	0.24 (0.85)
Number of distinct medication prescriptions: mean (sd)	5.52 (4.63)	5.20 (4.55)	7.44 (4.51)	7.11 (4.57)	6.03 (4.85)	5.67 (4.80)	8.31 (4.77)	7.83 (4.86)
Combined Comorbidity Score: mean (sd)	0.13 (1.63)	0.11 (1.61)	0.01 (1.38)	-0.01 (1.42)	0.25 (1.63)	0.25 (1.68)	0.16 (1.29)	0.11 (1.35)

Table D- Characteristics of patients included in the evaluation of comparative outcomes for *amlodipine-benazepril* between authorized generic (AG) and generic initiators and switchers <u>before propensity score</u> matching in each database

	Optum				Truven			
	AG initiators	Generic initiators	AG switchers	Generic switchers	AG initiators	Generic initiators	AG switchers	Generic switchers
Cohort selection steps								
Total number of patients filling prescriptions for the version of interest after 6-month continuous enrollment	72,221		128,725		665,841		665,928	
Patients meeting new drug use restriction	43,323		57,074		236,740		240,935	
Patients meeting prior brand-name use requirement (to define switchers, applicable for switchers comparison only)	NA		43,217		N/A		186,425	
Total eligible by exposure group	10,953	32,370	6,056	37,161	47,375	175,465	29,713	156,712
Baseline characteristics								
Age: mean (sd)	52.85 (11.40)	51.81 (11.26)	55.23 (10.24)	55.14 (10.37)	54.58 (12.39)	54.49 (12.51)	58.12 (12.00)	60.00 (12.50)
Gender								
MALE: n (%)	6,546 (59.8%)	19,941 (61.6%)	3,946 (65.2%)	23,392 (62.9%)	26,949 (56.9%)	101,602 (57.9%)	17,803 (59.9%)	89,062 (56.8%)
FEMALE: n (%)	4,406 (40.2%)	12,426 (38.4%)	2,110 (34.8%)	13,765 (37.0%)	20,426 (43.1%)	73,863 (42.1%)	11,910 (40.1%)	67,650 (43.2%)
UNKNOWN: n (%)	1 (0.0%)	3 (0.0%)	0 (0.0%)	4 (0.0%)	0 (0.0%)	0 (0.0%)	0 (0.0%)	0 (0.0%)
Region								
Northeast: n (%)	616 (5.6%)	2,440 (7.5%)	379 (6.3%)	3,102 (8.3%)	6,684 (14.1%)	25,526 (14.5%)	3,161 (10.6%)	14,886 (9.5%)
Midwest: n (%)	1,715 (15.7%)	5,559 (17.2%)	1,228 (20.3%)	7,977 (21.5%)	11,606 (24.5%)	37,813 (21.6%)	6,530 (22.0%)	45,437 (29.0%)
South: n (%)	7,864 (71.8%)	21,184 (65.4%)	3,987 (65.8%)	22,200 (59.7%)	23,753 (50.1%)	89,056 (50.8%)	16,974 (57.1%)	81,396 (51.9%)
West: n (%)	743 (6.8%)	3,176 (9.8%)	460 (7.6%)	3,875 (10.4%)	4,052 (8.6%)	17,559 (10.0%)	2,642 (8.9%)	14,182 (9.0%)
Unknown/other: n (%)	15 (0.1%)	11 (0.0%)	2 (0.0%)	7 (0.0%)	1,280 (2.7%)	5,511 (3.1%)	406 (1.4%)	811 (0.5%)
Hyperlipidemia: n (%)	4,070 (37.2%)	11,633 (35.9%)	2,708 (44.7%)	16,920 (45.5%)	13,726 (29.0%)	49,425 (28.2%)	9,350 (31.5%)	40,491 (25.8%)
Diabetes mellitus: n (%)	1,922 (17.5%)	4,905 (15.2%)	1,339 (22.1%)	7,177 (19.3%)	7,518 (15.9%)	27,382 (15.6%)	6,399 (21.5%)	30,380 (19.4%)
Hypertension: n (%)	7,659 (69.9%)	22,041 (68.1%)	4,221 (69.7%)	26,147 (70.4%)	28,929 (61.1%)	105,327 (60.0%)	16,931 (57.0%)	83,301 (53.2%)

Myocardial Infarction: n (%)	51 (0.5%)	132 (0.4%)	13 (0.2%)	81 (0.2%)	188 (0.4%)	697 (0.4%)	68 (0.2%)	362 (0.2%)
Angina: n (%)	198 (1.8%)	538 (1.7%)	93 (1.5%)	564 (1.5%)	786 (1.7%)	2,447 (1.4%)	361 (1.2%)	2,222 (1.4%)
Heart failure: n (%)	146 (1.3%)	399 (1.2%)	85 (1.4%)	450 (1.2%)	656 (1.4%)	2,195 (1.3%)	383 (1.3%)	2,050 (1.3%)
Other forms of chronic heart diseases: n (%)	658 (6.0%)	1,539 (4.8%)	433 (7.1%)	2,552 (6.9%)	2,491 (5.3%)	8,646 (4.9%)	1,860 (6.3%)	10,812 (6.9%)
Cerebrovascular accident: n (%)	170 (1.6%)	434 (1.3%)	68 (1.1%)	378 (1.0%)	670 (1.4%)	2,438 (1.4%)	295 (1.0%)	1,574 (1.0%)
Venous thromboembolism: n (%)	63 (0.6%)	254 (0.8%)	47 (0.8%)	245 (0.7%)	321 (0.7%)	1,249 (0.7%)	194 (0.7%)	1,139 (0.7%)
Atrial fibrillation: n (%)	137 (1.3%)	357 (1.1%)	119 (2.0%)	608 (1.6%)	681 (1.4%)	2,534 (1.4%)	595 (2.0%)	3,091 (2.0%)
Overweight or obese: n (%)	663 (6.1%)	2,125 (6.6%)	301 (5.0%)	1,611 (4.3%)	1,738 (3.7%)	8,384 (4.8%)	835 (2.8%)	2,125 (1.4%)
Tobacco use: n (%)	510 (4.7%)	1,519 (4.7%)	228 (3.8%)	1,191 (3.2%)	1,359 (2.9%)	5,239 (3.0%)	467 (1.6%)	1,611 (1.0%)
Alcohol abuse or dependence: n (%)	81 (0.7%)	290 (0.9%)	28 (0.5%)	227 (0.6%)	315 (0.7%)	1,095 (0.6%)	98 (0.3%)	442 (0.3%)
Renal disease: n (%)	138 (1.3%)	392 (1.2%)	97 (1.6%)	429 (1.2%)	509 (1.1%)	2,087 (1.2%)	361 (1.2%)	1,502 (1.0%)
Liver disease: n (%)	204 (1.9%)	649 (2.0%)	118 (1.9%)	675 (1.8%)	722 (1.5%)	2,791 (1.6%)	393 (1.3%)	1,837 (1.2%)
Aspirin: n (%)	1 (0.0%)	12 (0.0%)	3 (0.0%)	9 (0.0%)	193 (0.4%)	777 (0.4%)	146 (0.5%)	927 (0.6%)
Antiplatelets: n (%)	210 (1.9%)	621 (1.9%)	264 (4.4%)	1,351 (3.6%)	1,202 (2.5%)	4,111 (2.3%)	1,401 (4.7%)	8,045 (5.1%)
Statins: n (%)	2,185 (19.9%)	6,803 (21.0%)	2,343 (38.7%)	14,541 (39.1%)	10,298 (21.7%)	39,176 (22.3%)	12,015 (40.4%)	66,099 (42.2%)
Other lipid lowering agents: n (%)	612 (5.6%)	1,748 (5.4%)	734 (12.1%)	5,274 (14.2%)	2,492 (5.3%)	8,650 (4.9%)	3,718 (12.5%)	22,646 (14.5%)
Insulin preparations: n (%)	245 (2.2%)	693 (2.1%)	248 (4.1%)	947 (2.5%)	713 (1.5%)	2,664 (1.5%)	688 (2.3%)	2,863 (1.8%)
Oral hypoglycemic agents: n (%)	1,118 (10.2%)	3,160 (9.8%)	1,092 (18.0%)	6,029 (16.2%)	4,939 (10.4%)	18,216 (10.4%)	5,964 (20.1%)	29,697 (19.0%)
ACE inhibitors: n (%)	2,513 (22.9%)	7,403 (22.9%)	164 (2.7%)	891 (2.4%)	12,383 (26.1%)	44,250 (25.2%)	28,750 (96.8%)	151,737 (96.8%)
ARBs: n (%)	1,076 (9.8%)	3,394 (10.5%)	533 (8.8%)	2,970 (8.0%)	4,581 (9.7%)	17,000 (9.7%)	2,414 (8.1%)	13,468 (8.6%)
Calcium channel blockers (without amlodipine-benazepril): n (%)	515 (4.7%)	1,491 (4.6%)	82 (1.4%)	431 (1.2%)	2,301 (4.9%)	7,307 (4.2%)	386 (1.3%)	1,963 (1.3%)
Diuretics: n (%)	2,501 (22.8%)	7,504 (23.2%)	2,092 (34.5%)	12,044 (32.4%)	11,214 (23.7%)	40,355 (23.0%)	10,716 (36.1%)	54,964 (35.1%)
Beta-blockers: n (%)	1,616 (14.8%)	5,158 (15.9%)	1,449 (23.9%)	8,547 (23.0%)	8,168 (17.2%)	29,571 (16.9%)	7,805 (26.3%)	41,414 (26.4%)
Anticoagulants: n (%)	125 (1.1%)	428 (1.3%)	131 (2.2%)	825 (2.2%)	740 (1.6%)	2,788 (1.6%)	832 (2.8%)	4,810 (3.1%)
NSAIDs: n (%)	1,360 (12.4%)	4,313 (13.3%)	857 (14.2%)	5,085 (13.7%)	5,935 (12.5%)	23,513 (13.4%)	4,407 (14.8%)	23,783 (15.2%)
Coxibs: n (%)	168 (1.5%)	404 (1.2%)	139 (2.3%)	1,015 (2.7%)	685 (1.4%)	2,424 (1.4%)	827 (2.8%)	5,543 (3.5%)
Outpatient visits: mean (sd)	0.13 (0.35)	0.16 (0.40)	0.11 (0.34)	0.12 (0.35)	0.12 (0.36)	0.14 (0.38)	0.10 (0.31)	0.09 (0.31)

Inpatient hospitalization: mean (sd)	0.00 (0.06)	0.00 (0.06)	0.00 (0.07)	0.00 (0.05)	0.00 (0.00)	0.00 (0.01)	0.00 (0.00)	0.00 (0.01)
ED visit: mean (sd)	0.19 (0.57)	0.19 (0.64)	0.12 (0.46)	0.11 (0.42)	0.25 (0.85)	0.26 (0.89)	0.16 (0.62)	0.15 (0.62)
Number of distinct medication prescriptions: mean (sd)	3.76 (3.85)	4.01 (3.91)	5.79 (4.04)	5.71 (3.85)	3.96 (4.06)	4.09 (4.17)	6.12 (4.11)	6.32 (4.16)
Combined Comorbidity Score: mean (sd)	-0.26 (1.01)	-0.26 (1.02)	-0.24 (1.03)	-0.30 (0.98)	-0.23 (1.01)	-0.21 (1.00)	-0.18 (0.99)	-0.16 (0.97)

Table E- Characteristics of patients included in the evaluation of comparative outcomes for *calcitonin salmon* between authorized generic (AG) and generic initiators and switchers before propensity score matching in each database

	Optum				Truven			
	AG initiators	Generic initiators	AG switchers	Generic switchers	AG initiators	Generic initiators	AG switchers	Generic switchers
Cohort selection steps								
Total number of patients filling prescriptions for the version of interest after 6-month continuous enrollment	5,584		6,651		55,964		55,961	
Patients meeting new drug use restriction	3,679		4,236		33,798		34,059	
Patients meeting prior brand-name use requirement (to define switchers, applicable for switchers comparison only)	NA		1,879		NA		15,577	
Total eligible by exposure group	1,078	2,601	468	1,411	7,421	22,530	2,896	12,681
Baseline characteristics								
Age: mean (sd)	58.73 (13.30)	61.26 (12.94)	64.95 (11.47)	66.71 (10.17)	67.25 (15.23)	68.73 (14.49)	70.00 (13.19)	72.98 (11.64)
Gender								
MALE: n (%)	186 (17.3%)	410 (15.8%)	42 (9.0%)	105 (7.4%)	1,243 (16.7%)	3,578 (15.9%)	244 (8.4%)	984 (7.8%)
FEMALE: n (%)	892 (82.7%)	2,191 (84.2%)	426 (91.0%)	1,306 (92.6%)	6,178 (83.3%)	18,952 (84.1%)	2,652 (91.6%)	11,697 (92.2%)
UNKNOWN: n (%)	0 (0.0%)	0 (0.0%)	0 (0.0%)	0 (0.0%)	0 (0.0%)	0 (0.0%)	0 (0.0%)	0 (0.0%)
Region								
Northeast: n (%)	97 (9.0%)	255 (9.8%)	53 (11.3%)	130 (9.2%)	1,531 (20.6%)	4,220 (18.7%)	487 (16.8%)	1,963 (15.5%)
Midwest: n (%)	263 (24.4%)	595 (22.9%)	145 (31.0%)	342 (24.2%)	2,013 (27.1%)	6,242 (27.7%)	1,024 (35.4%)	4,878 (38.5%)
South: n (%)	518 (48.1%)	1,235 (47.5%)	193 (41.2%)	619 (43.9%)	2,573 (34.7%)	7,895 (35.0%)	808 (27.9%)	3,934 (31.0%)
West: n (%)	200 (18.6%)	510 (19.6%)	77 (16.5%)	319 (22.6%)	1,211 (16.3%)	3,935 (17.5%)	526 (18.2%)	1,827 (14.4%)
Unknown/other: n (%)	0 (0.0%)	6 (0.2%)	0 (0.0%)	1 (0.1%)	93 (1.3%)	238 (1.1%)	51 (1.8%)	79 (0.6%)
Outpatient visits: mean (sd)	0.28 (0.50)	0.31 (0.54)	0.22 (0.71)	0.24 (0.48)	0.20 (0.48)	0.19 (0.46)	0.18 (0.51)	0.11 (0.35)
Inpatient hospitalization: mean (sd)	0.05 (0.29)	0.05 (0.28)	0.03 (0.22)	0.03 (0.24)	0.00 (0.00)	0.00 (0.02)	0.00 (0.00)	0.00 (0.00)
ED visit: mean (sd)	0.44 (1.07)	0.44 (0.99)	0.29 (0.92)	0.17 (0.59)	0.71 (1.82)	0.64 (1.43)	0.30 (0.94)	0.28 (0.83)
Number of distinct medication prescriptions: mean (sd)	9.39 (6.11)	9.20 (5.85)	8.81 (6.00)	8.55 (5.28)	9.94 (6.04)	9.80 (5.84)	9.09 (5.58)	9.25 (5.45)
Combined Comorbidity Score: mean (sd)	0.83 (1.97)	0.83 (1.90)	0.47 (1.40)	0.54 (1.54)	1.01 (2.05)	0.93 (1.98)	0.65 (1.61)	0.48 (1.44)

Osteoporosis: n (%)	447 (41.5%)	1,067 (41.0%)	135 (28.8%)	457 (32.4%)	2,506 (33.8%)	7,645 (33.9%)	634 (21.9%)	2,318 (18.3%)
Kyphosis: n (%)	38 (3.5%)	116 (4.5%)	6 (1.3%)	26 (1.8%)	288 (3.9%)	863 (3.8%)	47 (1.6%)	158 (1.2%)
Vertebral facture: n (%)	209 (19.4%)	557 (21.4%)	19 (4.1%)	55 (3.9%)	1,834 (24.7%)	5,463 (24.2%)	126 (4.4%)	502 (4.0%)
Humerus fracture: n (%)	20 (1.9%)	33 (1.3%)	2 (0.4%)	12 (0.9%)	116 (1.6%)	327 (1.5%)	16 (0.6%)	84 (0.7%)
Wrist fracture: n (%)	16 (1.5%)	41 (1.6%)	2 (0.4%)	13 (0.9%)	109 (1.5%)	331 (1.5%)	15 (0.5%)	84 (0.7%)
Hip fracture: n (%)	18 (1.7%)	49 (1.9%)	6 (1.3%)	20 (1.4%)	200 (2.7%)	646 (2.9%)	44 (1.5%)	156 (1.2%)
Pelvis fracture: n (%)	21 (1.9%)	55 (2.1%)	8 (1.7%)	8 (0.6%)	174 (2.3%)	554 (2.5%)	28 (1.0%)	84 (0.7%)
Any other fractures: n (%)	163 (15.1%)	341 (13.1%)	25 (5.3%)	58 (4.1%)	1,032 (13.9%)	2,883 (12.8%)	139 (4.8%)	549 (4.3%)
Alzheimer disease or other dementia: n (%)	37 (3.4%)	98 (3.8%)	22 (4.7%)	47 (3.3%)	522 (7.0%)	1,426 (6.3%)	153 (5.3%)	530 (4.2%)
Asthma or chronic obstructive pulmonary disease: n (%)	125 (11.6%)	325 (12.5%)	48 (10.3%)	145 (10.3%)	930 (12.5%)	2,697 (12.0%)	257 (8.9%)	1,131 (8.9%)
Cataracts: n (%)	75 (7.0%)	231 (8.9%)	53 (11.3%)	193 (13.7%)	632 (8.5%)	2,240 (9.9%)	288 (9.9%)	1,440 (11.4%)
Crohn disease or gastroenteritis: n (%)	45 (4.2%)	98 (3.8%)	16 (3.4%)	48 (3.4%)	207 (2.8%)	615 (2.7%)	73 (2.5%)	306 (2.4%)
Depression: n (%)	33 (3.1%)	97 (3.7%)	15 (3.2%)	36 (2.6%)	285 (3.8%)	759 (3.4%)	68 (2.3%)	217 (1.7%)
Diabetes mellitus: n (%)	148 (13.7%)	323 (12.4%)	51 (10.9%)	128 (9.1%)	1,140 (15.4%)	3,263 (14.5%)	363 (12.5%)	1,488 (11.7%)
History of falls, syncope, or gait abnormality: n (%)	188 (17.4%)	460 (17.7%)	65 (13.9%)	146 (10.3%)	1,359 (18.3%)	4,001 (17.8%)	304 (10.5%)	1,115 (8.8%)
Hyperthyroidism: n (%)	15 (1.4%)	28 (1.1%)	9 (1.9%)	16 (1.1%)	91 (1.2%)	206 (0.9%)	22 (0.8%)	92 (0.7%)
Hyperparathyroidism: n (%)	12 (1.1%)	30 (1.2%)	3 (0.6%)	12 (0.9%)	81 (1.1%)	217 (1.0%)	28 (1.0%)	75 (0.6%)
Ischemic stroke: n (%)	9 (0.8%)	35 (1.3%)	4 (0.9%)	9 (0.6%)	140 (1.9%)	439 (1.9%)	38 (1.3%)	156 (1.2%)
Liver disease: n (%)	49 (4.5%)	103 (4.0%)	12 (2.6%)	30 (2.1%)	257 (3.5%)	707 (3.1%)	60 (2.1%)	189 (1.5%)
Malignant neoplasm: n (%)	123 (11.4%)	331 (12.7%)	47 (10.0%)	152 (10.8%)	844 (11.4%)	2,703 (12.0%)	338 (11.7%)	1,370 (10.8%)
Overweight or obese: n (%)	43 (4.0%)	116 (4.5%)	7 (1.5%)	21 (1.5%)	240 (3.2%)	716 (3.2%)	29 (1.0%)	91 (0.7%)
Parkinson disease: n (%)	9 (0.8%)	25 (1.0%)	5 (1.1%)	12 (0.9%)	83 (1.1%)	302 (1.3%)	31 (1.1%)	134 (1.1%)
Renal disease: n (%)	28 (2.6%)	78 (3.0%)	10 (2.1%)	25 (1.8%)	284 (3.8%)	776 (3.4%)	71 (2.5%)	208 (1.6%)
Rheumatoid arthritis: n (%)	57 (5.3%)	151 (5.8%)	17 (3.6%)	60 (4.3%)	342 (4.6%)	1,082 (4.8%)	98 (3.4%)	355 (2.8%)
Oral glucocorticoids: n (%)	321 (29.8%)	775 (29.8%)	121 (25.9%)	351 (24.9%)	2,341 (31.5%)	6,719 (29.8%)	735 (25.4%)	3,216 (25.4%)
Anticonvulsants/antiepileptic drugs: n (%)	178 (16.5%)	377 (14.5%)	62 (13.2%)	143 (10.1%)	1,200 (16.2%)	3,416 (15.2%)	367 (12.7%)	1,475 (11.6%)
Benzodiazepines: n (%)	270 (25.0%)	596 (22.9%)	112 (23.9%)	291 (20.6%)	1,694 (22.8%)	5,062 (22.5%)	603 (20.8%)	2,555 (20.1%)
SSRIs: n (%)	230 (21.3%)	528 (20.3%)	105 (22.4%)	275 (19.5%)	1,700 (22.9%)	5,032 (22.3%)	610 (21.1%)	2,457 (19.4%)
Beta-blockers: n (%)	214 (19.9%)	510 (19.6%)	97 (20.7%)	332 (23.5%)	1,903 (25.6%)	6,043 (26.8%)	799 (27.6%)	3,918 (30.9%)

Proton pump inhibitors: n (%)	258 (23.9%)	644 (24.8%)	122 (26.1%)	448 (31.8%)	2,240 (30.2%)	6,574 (29.2%)	1,022 (35.3%)	5,122 (40.4%)
Opioids: n (%)	577 (53.5%)	1,354 (52.1%)	151 (32.3%)	442 (31.3%)	4,076 (54.9%)	12,001 (53.3%)	1,026 (35.4%)	4,489 (35.4%)
Other anti-osteoporosis medications (without calcitonin): n (%)	268 (24.9%)	574 (22.1%)	76 (16.2%)	280 (19.8%)	1,594 (21.5%)	4,854 (21.5%)	445 (15.4%)	2,267 (17.9%)

Table F- Characteristics of patients included in the evaluation of comparative outcomes for *escitalopram* between authorized generic (AG) and generic initiators and switchers before propensity score matching in each database

	Optum				Truven			
	AG initiators	Generic initiators	AG switchers	Generic switchers	AG initiators	Generic initiators	AG switchers	Generic switchers
Cohort selection steps								
Total number of patients filling prescriptions for the version of interest after 6-month continuous enrollment	139,206		180,712		1,353,918		1,353,211	
Patients meeting new drug use restriction	79,631		95,726		741,945		607,818	
Patients meeting prior brand-name use requirement (to define switchers, applicable for switchers comparison only)	NA		58,459		NA		322,248	
Total eligible by exposure group	25,580	54,051	44,335	14,124	127,804	614,141	181,555	140,693
Baseline characteristics								
Age: mean (sd)	39.25 (14.98)	39.10 (14.76)	46.74 (14.49)	45.83 (14.74)	42.83 (17.75)	42.01 (17.56)	49.14 (16.00)	49.77 (15.83)
Gender								
MALE: n (%)	8,802 (34.4%)	17,996 (33.3%)	13,887 (31.3%)	4,221 (29.9%)	41,048 (32.1%)	198,619 (32.3%)	54,756 (30.2%)	41,831 (29.7%)
FEMALE: n (%)	16,774 (65.6%)	36,048 (66.7%)	30,445 (68.7%)	9,901 (70.1%)	86,756 (67.9%)	415,522 (67.7%)	126,799 (69.8%)	98,862 (70.3%)
UNKNOWN: n (%)	4 (0.0%)	7 (0.0%)	3 (0.0%)	2 (0.0%)	0 (0.0%)	0 (0.0%)	0 (0.0%)	0 (0.0%)
Region								
Northeast: n (%)	2,022 (7.9%)	5,609 (10.4%)	4,649 (10.5%)	1,712 (12.1%)	21,201 (16.6%)	108,343 (17.6%)	40,650 (22.4%)	30,043 (21.4%)
Midwest: n (%)	6,310 (24.7%)	14,102 (26.1%)	11,426 (25.8%)	2,843 (20.1%)	25,973 (20.3%)	136,621 (22.2%)	42,562 (23.4%)	31,756 (22.6%)
South: n (%)	13,025 (50.9%)	25,613 (47.4%)	20,894 (47.1%)	7,156 (50.7%)	59,243 (46.4%)	259,111 (42.2%)	73,923 (40.7%)	54,455 (38.7%)
West: n (%)	4,214 (16.5%)	8,716 (16.1%)	7,355 (16.6%)	2,413 (17.1%)	18,806 (14.7%)	94,415 (15.4%)	21,339 (11.8%)	21,378 (15.2%)
Unknown/other: n (%)	9 (0.0%)	11 (0.0%)	11 (0.0%)	0 (0.0%)	2,581 (2.0%)	15,651 (2.5%)	3,081 (1.7%)	3,061 (2.2%)
Epilepsy: n (%)	357 (1.4%)	714 (1.3%)	494 (1.1%)	175 (1.2%)	1,839 (1.4%)	8,704 (1.4%)	2,116 (1.2%)	1,586 (1.1%)

Depression: n (%)	2,597 (10.2%)	5,376 (9.9%)	3,968 (9.0%)	1,291 (9.1%)	12,928 (10.1%)	61,656 (10.0%)	15,243 (8.4%)	10,747 (7.6%)
Anxiety: n (%)	2,668 (10.4%)	5,694 (10.5%)	4,370 (9.9%)	1,464 (10.4%)	11,363 (8.9%)	56,743 (9.2%)	14,168 (7.8%)	10,174 (7.2%)
Alcohol abuse or dependence: n (%)	578 (2.3%)	1,204 (2.2%)	585 (1.3%)	207 (1.5%)	2,267 (1.8%)	11,072 (1.8%)	1,948 (1.1%)	1,284 (0.9%)
Drug abuse or dependence: n (%)	604 (2.4%)	1,354 (2.5%)	522 (1.2%)	207 (1.5%)	2,834 (2.2%)	13,505 (2.2%)	1,816 (1.0%)	1,269 (0.9%)
Sleep disorder: n (%)	2,214 (8.7%)	4,552 (8.4%)	3,405 (7.7%)	1,039 (7.4%)	8,822 (6.9%)	41,582 (6.8%)	10,396 (5.7%)	7,391 (5.3%)
Psychotic disorder: n (%)	431 (1.7%)	796 (1.5%)	486 (1.1%)	179 (1.3%)	2,211 (1.7%)	10,996 (1.8%)	1,914 (1.1%)	1,448 (1.0%)
Personality disorder: n (%)	119 (0.5%)	298 (0.6%)	192 (0.4%)	67 (0.5%)	570 (0.4%)	2,745 (0.4%)	582 (0.3%)	422 (0.3%)
Adjustment reaction/post-traumatic stress disorder: n (%)	1,568 (6.1%)	3,359 (6.2%)	1,940 (4.4%)	637 (4.5%)	7,158 (5.6%)	34,217 (5.6%)	7,056 (3.9%)	4,915 (3.5%)
ADHD: n (%)	1,472 (5.8%)	3,192 (5.9%)	1,978 (4.5%)	754 (5.3%)	5,831 (4.6%)	29,790 (4.9%)	5,798 (3.2%)	4,270 (3.0%)
Delirium: n (%)	322 (1.3%)	670 (1.2%)	390 (0.9%)	151 (1.1%)	1,964 (1.5%)	8,994 (1.5%)	1,447 (0.8%)	1,123 (0.8%)
Bipolar disorder: n (%)	80 (0.3%)	138 (0.3%)	114 (0.3%)	50 (0.4%)	287 (0.2%)	1,279 (0.2%)	393 (0.2%)	292 (0.2%)
Other psychiatric disorder: n (%)	1,273 (5.0%)	2,715 (5.0%)	1,264 (2.9%)	440 (3.1%)	5,142 (4.0%)	25,938 (4.2%)	4,267 (2.4%)	3,011 (2.1%)
Psychiatric hospitalization: mean (sd)	0.04 (0.24)	0.03 (0.27)	0.01 (0.15)	0.02 (0.19)	0.03 (0.21)	0.03 (0.21)	0.01 (0.14)	0.01 (0.11)
Psychiatric office visit: mean (sd)	2.45 (8.29)	2.53 (11.21)	2.04 (8.17)	2.18 (7.11)	2.52 (10.58)	2.51 (12.38)	2.07 (7.21)	1.93 (7.62)
Anticonvulsants/antiepileptic drugs: n (%)	2,046 (8.0%)	4,180 (7.7%)	4,465 (10.1%)	1,394 (9.9%)	10,879 (8.5%)	51,387 (8.4%)	19,919 (11.0%)	14,630 (10.4%)
SSRIs (without escitalopram): n (%)	5,668 (22.2%)	11,325 (21.0%)	2,170 (4.9%)	921 (6.5%)	26,094 (20.4%)	116,644 (19.0%)	8,187 (4.5%)	5,577 (4.0%)
SNRIs: n (%)	1,358 (5.3%)	2,915 (5.4%)	880 (2.0%)	332 (2.4%)	6,710 (5.3%)	30,495 (5.0%)	3,920 (2.2%)	2,942 (2.1%)
Tricyclic antidepressants: n (%)	596 (2.3%)	1,249 (2.3%)	993 (2.2%)	354 (2.5%)	3,315 (2.6%)	15,328 (2.5%)	4,562 (2.5%)	3,449 (2.5%)
Antipsychotics: n (%)	1,109 (4.3%)	2,249 (4.2%)	2,625 (5.9%)	878 (6.2%)	5,698 (4.5%)	25,744 (4.2%)	11,796 (6.5%)	8,523 (6.1%)
Benzodiazepines: n (%)	6,316 (24.7%)	12,845 (23.8%)	11,311 (25.5%)	3,826 (27.1%)	30,808 (24.1%)	140,052 (22.8%)	48,227 (26.6%)	34,827 (24.8%)

Non-BZD sedative hypnotics: n (%)	3,017 (11.8%)	6,236 (11.5%)	6,348 (14.3%)	2,025 (14.3%)	15,072 (11.8%)	66,593 (10.8%)	25,461 (14.0%)	18,886 (13.4%)
Outpatient visits: mean (sd)	0.26 (0.51)	0.26 (0.55)	0.27 (0.57)	0.27 (0.51)	0.22 (0.48)	0.23 (0.48)	0.21 (0.45)	0.22 (0.47)
Inpatient hospitalization: mean (sd)	0.01 (0.12)	0.01 (0.17)	0.01 (0.12)	0.01 (0.15)	0.00 (0.01)	0.00 (0.01)	0.00 (0.01)	0.00 (0.02)
ED visit: mean (sd)	0.28 (0.87)	0.27 (0.79)	0.15 (0.62)	0.17 (0.60)	0.42 (1.27)	0.43 (1.32)	0.25 (0.93)	0.24 (0.89)
Number of distinct medication prescriptions: mean (sd)	4.54 (4.29)	4.47 (4.17)	6.27 (4.45)	6.23 (4.55)	4.98 (4.61)	4.83 (4.50)	6.78 (4.66)	6.69 (4.67)
Combined Comorbidity Score: mean (sd)	0.35 (1.14)	0.32 (1.07)	0.30 (1.07)	0.33 (1.11)	0.40 (1.29)	0.39 (1.25)	0.29 (1.10)	0.28 (1.09)

Table G- Characteristics of patients included in the evaluation of comparative outcomes for *glipizide* between authorized generic (AG) and generic initiators and switchers <u>before propensity score</u> matching in each database

	Optum				Truven			
	AG initiators	Generic initiators	AG switchers	Generic switchers	AG initiators	Generic initiators	AG switchers	Generic switchers
Cohort selection steps								
Total number of patients filling prescriptions for the version of interest after 6-month continuous enrollment	**46,110**		**148,589**		**543,534**		**538,518**	
Patients meeting new drug use restriction	**39,996**		**31,623**		**261,240**		**131,887**	
Patients meeting prior brand-name use requirement (to define switchers, applicable for switchers comparison only)	NA		**13,131**		NA		**60,974**	
Total eligible by exposure group	**2,194**	**37,802**	**735**	**12,396**	**66,713**	**194,527**	**2,849**	**58,125**
Baseline characteristics								
Age: mean (sd)	58.49 (11.34)	57.16 (12.17)	59.75 (10.21)	57.64 (10.81)	58.65 (12.63)	58.36 (13.42)	65.13 (11.47)	63.74 (12.21)
Gender								
MALE: n (%)	1,243 (56.7%)	21,067 (55.7%)	437 (59.5%)	7,066 (57.0%)	37,126 (55.7%)	103,144 (53.0%)	1,444 (50.7%)	31,970 (55.0%)
FEMALE: n (%)	950 (43.3%)	16,728 (44.3%)	298 (40.5%)	5,330 (43.0%)	29,587 (44.3%)	91,383 (47.0%)	1,405 (49.3%)	26,155 (45.0%)
UNKNOWN: n (%)	1 (0.0%)	7 (0.0%)	0 (0.0%)	0 (0.0%)	0 (0.0%)	0 (0.0%)	0 (0.0%)	0 (0.0%)
Region								
Northeast: n (%)	199 (9.1%)	3,605 (9.5%)	119 (16.2%)	1,587 (12.8%)	12,711 (19.1%)	29,194 (15.0%)	511 (17.9%)	6,175 (10.6%)
Midwest: n (%)	388 (17.7%)	10,628 (28.1%)	160 (21.8%)	4,355 (35.1%)	18,947 (28.4%)	51,495 (26.5%)	839 (29.4%)	22,567 (38.8%)
South: n (%)	1,179 (53.7%)	19,134 (50.6%)	371 (50.5%)	5,702 (46.0%)	25,348 (38.0%)	84,828 (43.6%)	1,137 (39.9%)	22,249 (38.3%)
West: n (%)	425 (19.4%)	4,371 (11.6%)	85 (11.6%)	732 (5.9%)	8,333 (12.5%)	25,403 (13.1%)	334 (11.7%)	6,779 (11.7%)
Unknown/other: n (%)	3 (0.1%)	64 (0.2%)	0 (0.0%)	20 (0.2%)	1,374 (2.1%)	3,607 (1.9%)	28 (1.0%)	355 (0.6%)
Hyperlipidemia: n (%)	873 (39.8%)	10,963 (29.0%)	449 (61.1%)	6,169 (49.8%)	26,762 (40.1%)	71,477 (36.7%)	691 (24.3%)	9,973 (17.2%)
Diabetes mellitus: n (%)	1,415 (64.5%)	18,942 (50.1%)	654 (89.0%)	10,325 (83.3%)	52,524 (78.7%)	146,405 (75.3%)	2,125 (74.6%)	38,936 (67.0%)
Hypertension: n (%)	996 (45.4%)	12,657 (33.5%)	454 (61.8%)	6,801 (54.9%)	31,789 (47.7%)	89,887 (46.2%)	991 (34.8%)	16,378 (28.2%)

Myocardial Infarction: n (%)	23 (1.0%)	289 (0.8%)	9 (1.2%)	106 (0.9%)	621 (0.9%)	2,682 (1.4%)	21 (0.7%)	493 (0.8%)
Angina: n (%)	62 (2.8%)	824 (2.2%)	26 (3.5%)	383 (3.1%)	1,562 (2.3%)	5,547 (2.9%)	71 (2.5%)	1,889 (3.2%)
Heart failure: n (%)	85 (3.9%)	1,425 (3.8%)	37 (5.0%)	627 (5.1%)	2,877 (4.3%)	10,876 (5.6%)	116 (4.1%)	2,756 (4.7%)
Other forms of chronic heart diseases: n (%)	228 (10.4%)	2,965 (7.8%)	102 (13.9%)	1,670 (13.5%)	7,232 (10.8%)	21,910 (11.3%)	374 (13.1%)	6,761 (11.6%)
Cerebrovascular accident: n (%)	28 (1.3%)	424 (1.1%)	9 (1.2%)	171 (1.4%)	1,171 (1.8%)	4,365 (2.2%)	42 (1.5%)	872 (1.5%)
Venous thromboembolism: n (%)	26 (1.2%)	328 (0.9%)	12 (1.6%)	124 (1.0%)	863 (1.3%)	3,035 (1.6%)	35 (1.2%)	547 (0.9%)
Atrial fibrillation: n (%)	60 (2.7%)	976 (2.6%)	24 (3.3%)	431 (3.5%)	2,538 (3.8%)	8,652 (4.4%)	124 (4.4%)	1,878 (3.2%)
Overweight or obese: n (%)	101 (4.6%)	1,408 (3.7%)	46 (6.3%)	613 (4.9%)	4,194 (6.3%)	11,058 (5.7%)	40 (1.4%)	473 (0.8%)
Tobacco use: n (%)	36 (1.6%)	840 (2.2%)	14 (1.9%)	258 (2.1%)	2,126 (3.2%)	6,013 (3.1%)	24 (0.8%)	350 (0.6%)
Alcohol abuse or dependence: n (%)	6 (0.3%)	155 (0.4%)	4 (0.5%)	55 (0.4%)	325 (0.5%)	975 (0.5%)	3 (0.1%)	101 (0.2%)
Renal disease: n (%)	50 (2.3%)	641 (1.7%)	31 (4.2%)	322 (2.6%)	2,331 (3.5%)	6,739 (3.5%)	62 (2.2%)	916 (1.6%)
Liver disease: n (%)	48 (2.2%)	699 (1.8%)	21 (2.9%)	301 (2.4%)	1,874 (2.8%)	5,525 (2.8%)	42 (1.5%)	653 (1.1%)
Aspirin: n (%)	0 (0.0%)	3 (0.0%)	0 (0.0%)	72 (0.6%)	156 (0.2%)	385 (0.2%)	1 (0.0%)	28 (0.0%)
Antiplatelets: n (%)	89 (4.1%)	936 (2.5%)	47 (6.4%)	635 (5.1%)	3,827 (5.7%)	10,812 (5.6%)	251 (8.8%)	4,100 (7.1%)
Statins: n (%)	621 (28.3%)	7,291 (19.3%)	406 (55.2%)	5,369 (43.3%)	30,738 (46.1%)	82,362 (42.3%)	1,572 (55.2%)	27,960 (48.1%)
Other lipid lowering agents: n (%)	205 (9.3%)	2,154 (5.7%)	124 (16.9%)	1,393 (11.2%)	8,257 (12.4%)	21,302 (11.0%)	454 (15.9%)	4,566 (7.9%)
Insulin preparations: n (%)	0 (0.0%)	0 (0.0%)	0 (0.0%)	0 (0.0%)	0 (0.0%)	0 (0.0%)	0 (0.0%)	0 (0.0%)
Oral hypoglycemic agents (without glipizide): n (%)	913 (41.6%)	11,793 (31.2%)	507 (69.0%)	7,575 (61.1%)	41,818 (62.7%)	113,417 (58.3%)	1,879 (66.0%)	34,697 (59.7%)
ACE inhibitors: n (%)	617 (28.1%)	8,014 (21.2%)	331 (45.0%)	5,750 (46.4%)	24,457 (36.7%)	68,760 (35.3%)	1,224 (43.0%)	27,561 (47.4%)
ARBs: n (%)	272 (12.4%)	3,155 (8.3%)	169 (23.0%)	2,125 (17.1%)	12,621 (18.9%)	33,957 (17.5%)	809 (28.4%)	11,292 (19.4%)
Calcium channel blockers: n (%)	333 (15.2%)	3,847 (10.2%)	154 (21.0%)	2,629 (21.2%)	13,048 (19.6%)	37,814 (19.4%)	774 (27.2%)	15,272 (26.3%)
Diuretics: n (%)	541 (24.7%)	7,415 (19.6%)	275 (37.4%)	4,499 (36.3%)	22,742 (34.1%)	67,599 (34.8%)	1,282 (45.0%)	25,428 (43.7%)
Beta-blockers: n (%)	372 (17.0%)	4,904 (13.0%)	170 (23.1%)	2,958 (23.9%)	15,826 (23.7%)	45,688 (23.5%)	857 (30.1%)	17,128 (29.5%)
Anticoagulants: n (%)	63 (2.9%)	974 (2.6%)	29 (3.9%)	547 (4.4%)	2,873 (4.3%)	9,259 (4.8%)	192 (6.7%)	3,724 (6.4%)
NSAIDs: n (%)	195 (8.9%)	2,358 (6.2%)	80 (10.9%)	1,492 (12.0%)	8,828 (13.2%)	26,057 (13.4%)	397 (13.9%)	7,113 (12.2%)
Coxibs: n (%)	66 (3.0%)	1,064 (2.8%)	39 (5.3%)	1,076 (8.7%)	1,370 (2.1%)	5,554 (2.9%)	218 (7.7%)	7,825 (13.5%)
Outpatient visits: mean (sd)	0.08 (0.28)	0.05 (0.23)	0.11 (0.32)	0.10 (0.34)	0.11 (0.40)	0.11 (0.37)	0.07 (0.35)	0.05 (0.27)
Inpatient hospitalization: mean (sd)	0.00 (0.06)	0.01 (0.11)	0.00 (0.05)	0.01 (0.08)	0.00 (0.01)	0.00 (0.01)	0.00 (0.00)	0.00 (0.00)
ED visit: mean (sd)	0.20 (0.73)	0.16 (0.66)	0.17 (0.60)	0.22 (1.03)	0.27 (0.93)	0.33 (1.00)	0.18 (0.61)	0.17 (0.60)

Number of distinct medication prescriptions: mean (sd)	4.33 (4.31)	3.38 (4.04)	8.27 (4.23)	7.71 (4.43)	6.61 (4.82)	6.75 (5.06)	8.93 (4.69)	8.53 (4.67)
Combined Comorbidity Score	0.21 (1.34)	0.19 (1.25)	0.21 (1.37)	0.20 (1.39)	0.29 (1.43)	0.39 (1.57)	0.33 (1.20)	0.32 (1.20)

Table H- Characteristics of patients included in the evaluation of comparative outcomes for *quinapril* between authorized generic (AG) and generic initiators and switchers before propensity score matching in each database

	Optum				Truven			
	AG initiators	Generic initiators	AG switchers	Generic switchers	AG initiators	Generic initiators	AG switchers	Generic switchers
Cohort selection steps								
Total number of patients filling prescriptions for the version of interest after 6-month continuous enrollment	36,723		92,337		326,298		326,014	
Patients meeting new drug use restriction	20,458		40,444		82,411		108,556	
Patients meeting prior brand-name use requirement (to define switchers, applicable for switchers comparison only)	NA		33,514		NA		82,982	
Total eligible by exposure group	9,622	10,836	15,371	18,143	41,347	41,064	55,591	27,391
Baseline characteristics								
Age: mean (sd)	54.09 (11.79)	52.63 (11.75)	57.18 (10.25)	55.30 (10.64)	58.47 (13.34)	56.56 (13.00)	63.99 (11.95)	59.07 (12.65)
Gender								
MALE: n (%)	5,551 (57.7%)	6,258 (57.8%)	9,337 (60.7%)	10,714 (59.1%)	22,920 (55.4%)	22,346 (54.4%)	30,967 (55.7%)	14,787 (54.0%)
FEMALE: n (%)	4,070 (42.3%)	4,576 (42.2%)	6,033 (39.2%)	7,428 (40.9%)	18,427 (44.6%)	18,718 (45.6%)	24,624 (44.3%)	12,604 (46.0%)
UNKNOWN: n (%)	1 (0.0%)	2 (0.0%)	1 (0.0%)	1 (0.0%)	0 (0.0%)	0 (0.0%)	0 (0.0%)	0 (0.0%)
Region								
Northeast: n (%)	1,771 (18.4%)	1,177 (10.9%)	2,238 (14.6%)	2,292 (12.6%)	12,689 (30.7%)	8,936 (21.8%)	8,214 (14.8%)	2,955 (10.8%)
Midwest: n (%)	2,188 (22.7%)	2,991 (27.6%)	4,843 (31.5%)	5,798 (32.0%)	10,194 (24.7%)	10,791 (26.3%)	21,785 (39.2%)	7,074 (25.8%)
South: n (%)	4,857 (50.5%)	5,711 (52.7%)	6,499 (42.3%)	8,612 (47.5%)	14,143 (34.2%)	16,107 (39.2%)	17,284 (31.1%)	13,501 (49.3%)
West: n (%)	794 (8.3%)	945 (8.7%)	1,775 (11.5%)	1,431 (7.9%)	3,711 (9.0%)	4,247 (10.3%)	7,711 (13.9%)	3,692 (13.5%)
Unknown/other: n (%)	12 (0.1%)	12 (0.1%)	16 (0.1%)	10 (0.1%)	610 (1.5%)	983 (2.4%)	597 (1.1%)	169 (0.6%)
Hyperlipidemia: n (%)	4,071 (42.3%)	4,449 (41.1%)	7,593 (49.4%)	8,367 (46.1%)	13,400 (32.4%)	13,471 (32.8%)	12,055 (21.7%)	5,957 (21.7%)

Diabetes mellitus: n (%)	2,627 (27.3%)	3,036 (28.0%)	4,507 (29.3%)	5,316 (29.3%)	11,524 (27.9%)	11,214 (27.3%)	15,054 (27.1%)	7,062 (25.8%)
Hypertension: n (%)	5,918 (61.5%)	6,607 (61.0%)	10,038 (65.3%)	12,060 (66.5%)	21,294 (51.5%)	21,492 (52.3%)	24,069 (43.3%)	12,055 (44.0%)
Myocardial Infarction: n (%)	199 (2.1%)	280 (2.6%)	122 (0.8%)	110 (0.6%)	872 (2.1%)	840 (2.0%)	327 (0.6%)	158 (0.6%)
Angina: n (%)	376 (3.9%)	436 (4.0%)	431 (2.8%)	476 (2.6%)	1,459 (3.5%)	1,296 (3.2%)	1,618 (2.9%)	638 (2.3%)
Heart failure: n (%)	401 (4.2%)	492 (4.5%)	544 (3.5%)	654 (3.6%)	1,991 (4.8%)	1,776 (4.3%)	2,358 (4.2%)	1,013 (3.7%)
Other forms of chronic heart diseases: n (%)	1,099 (11.4%)	1,233 (11.4%)	2,112 (13.7%)	2,107 (11.6%)	5,081 (12.3%)	4,316 (10.5%)	7,902 (14.2%)	2,816 (10.3%)
Cerebrovascular accident: n (%)	196 (2.0%)	245 (2.3%)	148 (1.0%)	223 (1.2%)	922 (2.2%)	906 (2.2%)	821 (1.5%)	344 (1.3%)
Venous thromboembolism: n (%)	111 (1.2%)	129 (1.2%)	144 (0.9%)	158 (0.9%)	502 (1.2%)	480 (1.2%)	491 (0.9%)	213 (0.8%)
Atrial fibrillation: n (%)	320 (3.3%)	357 (3.3%)	494 (3.2%)	510 (2.8%)	1,745 (4.2%)	1,636 (4.0%)	2,257 (4.1%)	854 (3.1%)
Overweight or obese: n (%)	501 (5.2%)	708 (6.5%)	562 (3.7%)	729 (4.0%)	1,412 (3.4%)	1,874 (4.6%)	344 (0.6%)	260 (0.9%)
Tobacco use: n (%)	386 (4.0%)	528 (4.9%)	325 (2.1%)	419 (2.3%)	973 (2.4%)	1,092 (2.7%)	234 (0.4%)	189 (0.7%)
Alcohol abuse or dependence: n (%)	85 (0.9%)	125 (1.2%)	52 (0.3%)	73 (0.4%)	221 (0.5%)	222 (0.5%)	102 (0.2%)	62 (0.2%)
Renal disease: n (%)	221 (2.3%)	238 (2.2%)	258 (1.7%)	275 (1.5%)	850 (2.1%)	894 (2.2%)	688 (1.2%)	368 (1.3%)
Liver disease: n (%)	229 (2.4%)	237 (2.2%)	251 (1.6%)	341 (1.9%)	740 (1.8%)	699 (1.7%)	559 (1.0%)	280 (1.0%)
Aspirin: n (%)	3 (0.0%)	11 (0.1%)	13 (0.1%)	31 (0.2%)	206 (0.5%)	192 (0.5%)	406 (0.7%)	184 (0.7%)
Antiplatelets: n (%)	328 (3.4%)	319 (2.9%)	828 (5.4%)	888 (4.9%)	1,902 (4.6%)	1,633 (4.0%)	4,310 (7.8%)	1,651 (6.0%)
Statins: n (%)	2,317 (24.1%)	2,471 (22.8%)	7,318 (47.6%)	7,306 (40.3%)	11,810 (28.6%)	10,577 (25.8%)	29,151 (52.4%)	11,863 (43.3%)
Other lipid lowering agents: n (%)	713 (7.4%)	752 (6.9%)	1,868 (12.2%)	1,936 (10.7%)	3,262 (7.9%)	2,681 (6.5%)	5,701 (10.3%)	2,488 (9.1%)
Insulin preparations: n (%)	459 (4.8%)	567 (5.2%)	851 (5.5%)	1,020 (5.6%)	1,428 (3.5%)	1,547 (3.8%)	2,012 (3.6%)	1,057 (3.9%)
Oral hypoglycemic agents: n (%)	1,541 (16.0%)	1,859 (17.2%)	3,790 (24.7%)	4,554 (25.1%)	7,274 (17.6%)	6,936 (16.9%)	15,352 (27.6%)	7,231 (26.4%)
ACE inhibitors: n (%)	1,119 (11.6%)	1,281 (11.8%)	122 (0.8%)	243 (1.3%)	6,768 (16.4%)	6,200 (15.1%)	53,478 (96.2%)	26,856 (98.0%)
ARBs: n (%)	645 (6.7%)	766 (7.1%)	577 (3.8%)	714 (3.9%)	3,269 (7.9%)	2,819 (6.9%)	2,613 (4.7%)	1,241 (4.5%)
Calcium channel blockers (without quinapril): n (%)	1,340 (13.9%)	1,553 (14.3%)	3,443 (22.4%)	4,055 (22.4%)	2,635 (6.4%)	2,453 (6.0%)	7,137 (12.8%)	3,056 (11.2%)
Diuretics: n (%)	2,180 (22.7%)	2,576 (23.8%)	5,684 (37.0%)	6,698 (36.9%)	10,892 (26.3%)	10,306 (25.1%)	24,194 (43.5%)	11,441 (41.8%)

Beta-blockers: n (%)	1,611 (16.7%)	1,826 (16.9%)	4,132 (26.9%)	4,710 (26.0%)	8,364 (20.2%)	7,361 (17.9%)	17,965 (32.3%)	7,621 (27.8%)
Anticoagulants: n (%)	251 (2.6%)	307 (2.8%)	619 (4.0%)	736 (4.1%)	1,524 (3.7%)	1,340 (3.3%)	3,765 (6.8%)	1,462 (5.3%)
NSAIDs: n (%)	1,078 (11.2%)	1,309 (12.1%)	1,926 (12.5%)	2,284 (12.6%)	4,566 (11.0%)	4,656 (11.3%)	7,389 (13.3%)	3,612 (13.2%)
Coxibs: n (%)	168 (1.7%)	200 (1.8%)	1,199 (7.8%)	1,243 (6.9%)	834 (2.0%)	754 (1.8%)	6,449 (11.6%)	2,341 (8.5%)
Outpatient visits: mean (sd)	0.14 (0.38)	0.15 (0.40)	0.13 (0.41)	0.13 (0.40)	0.12 (0.37)	0.13 (0.38)	0.07 (0.27)	0.08 (0.29)
Inpatient hospitalization: mean (sd)	0.01 (0.09)	0.01 (0.13)	0.00 (0.07)	0.00 (0.08)	0.00 (0.01)	0.00 (0.00)	0.00 (0.01)	0.00 (0.00)
ED visit: mean (sd)	0.22 (0.73)	0.25 (0.78)	0.12 (0.51)	0.15 (0.61)	0.25 (0.86)	0.27 (0.90)	0.15 (0.56)	0.17 (0.64)
Number of distinct medication prescriptions: mean (sd)	4.08 (4.12)	4.29 (4.22)	6.65 (4.14)	6.52 (4.17)	4.60 (4.80)	4.43 (4.53)	7.72 (4.53)	7.19 (4.55)
Combined Comorbidity Score: mean (sd)	0.04 (1.35)	0.08 (1.40)	-0.09 (1.19)	-0.12 (1.18)	0.16 (1.37)	0.12 (1.35)	0.10 (1.15)	0.02 (1.10)

Table I- Characteristics of patients included in the evaluation of comparative outcomes for *sertraline* between authorized generic (AG) and generic initiators and switchers before propensity score matching in each database

	Optum				Truven			
	AG initiators	Generic initiators	AG switchers	Generic switchers	AG initiators	Generic initiators	AG switchers	Generic switchers
Cohort selection steps								
Total number of patients filling prescriptions for the version of interest after 6-month continuous enrollment	544,275		758,096		2,909,191		3,392,102	
Patients meeting new drug use restriction	420,347		272,990		1,782,673		903,262	
Patients meeting prior brand-name use requirement (to define switchers, applicable for switchers comparison only)	NA		111,191		NA		253,183	
Total eligible by exposure group	193,445	226,902	52,385	58,806	688,454	1,094,219	112,479	140,704
Baseline characteristics								
Age: mean (sd)	37.72 (14.86)	38.93 (15.47)	43.34 (14.13)	46.05 (14.68)	40.58 (17.22)	41.86 (18.19)	49.19 (16.86)	52.76 (17.32)
Gender								
MALE: n (%)	64,247 (33.2%)	75,287 (33.2%)	15,300 (29.2%)	16,852 (28.7%)	222,999 (32.4%)	359,263 (32.8%)	32,127 (28.6%)	40,969 (29.1%)
FEMALE: n (%)	129,183 (66.8%)	151,593 (66.8%)	37,080 (70.8%)	41,946 (71.3%)	465,455 (67.6%)	734,956 (67.2%)	80,352 (71.4%)	99,735 (70.9%)
UNKNOWN: n (%)	15 (0.0%)	22 (0.0%)	5 (0.0%)	8 (0.0%)	0 (0.0%)	0 (0.0%)	0 (0.0%)	0 (0.0%)
Region								
Northeast: n (%)	15,226 (7.9%)	20,171 (8.9%)	3,290 (6.3%)	7,629 (13.0%)	85,769 (12.5%)	184,573 (16.9%)	9,700 (8.6%)	18,407 (13.1%)
Midwest: n (%)	61,357 (31.7%)	60,568 (26.7%)	17,989 (34.3%)	17,679 (30.1%)	204,062 (29.6%)	272,017 (24.9%)	31,455 (28.0%)	45,171 (32.1%)
South: n (%)	88,340 (45.7%)	114,522 (50.5%)	22,548 (43.0%)	26,325 (44.8%)	271,782 (39.5%)	451,398 (41.3%)	52,037 (46.3%)	60,661 (43.1%)
West: n (%)	28,468 (14.7%)	31,550 (13.9%)	8,538 (16.3%)	7,142 (12.1%)	114,430 (16.6%)	159,352 (14.6%)	18,784 (16.7%)	15,492 (11.0%)
Unknown/other: n (%)	54 (0.0%)	91 (0.0%)	20 (0.0%)	31 (0.1%)	12,411 (1.8%)	26,879 (2.5%)	503 (0.4%)	973 (0.7%)
Epilepsy: n (%)	2,550 (1.3%)	2,767 (1.2%)	590 (1.1%)	676 (1.1%)	8,652 (1.3%)	14,730 (1.3%)	1,131 (1.0%)	1,458 (1.0%)
Depression: n (%)	18,880 (9.8%)	21,187 (9.3%)	4,287 (8.2%)	4,537 (7.7%)	64,958 (9.4%)	103,014 (9.4%)	6,113 (5.4%)	6,706 (4.8%)
Anxiety: n (%)	17,495 (9.0%)	19,851 (8.7%)	3,858 (7.4%)	3,875 (6.6%)	52,367 (7.6%)	88,662 (8.1%)	4,556 (4.1%)	5,058 (3.6%)

Alcohol abuse or dependence: n (%)	4,182 (2.2%)	4,405 (1.9%)	621 (1.2%)	537 (0.9%)	11,427 (1.7%)	17,999 (1.6%)	648 (0.6%)	678 (0.5%)
Drug abuse or dependence: n (%)	4,409 (2.3%)	4,648 (2.0%)	527 (1.0%)	429 (0.7%)	13,135 (1.9%)	20,448 (1.9%)	566 (0.5%)	471 (0.3%)
Sleep disorder: n (%)	16,158 (8.4%)	19,024 (8.4%)	3,948 (7.5%)	4,538 (7.7%)	40,969 (6.0%)	67,160 (6.1%)	5,607 (5.0%)	7,051 (5.0%)
Psychotic disorder: n (%)	2,877 (1.5%)	3,415 (1.5%)	559 (1.1%)	577 (1.0%)	10,775 (1.6%)	19,472 (1.8%)	997 (0.9%)	1,194 (0.8%)
Personality disorder: n (%)	1,187 (0.6%)	1,314 (0.6%)	291 (0.6%)	266 (0.5%)	3,040 (0.4%)	5,004 (0.5%)	212 (0.2%)	225 (0.2%)
Adjustment reaction/post-traumatic stress disorder: n (%)	11,517 (6.0%)	12,555 (5.5%)	2,037 (3.9%)	1,965 (3.3%)	36,624 (5.3%)	56,754 (5.2%)	2,919 (2.6%)	3,042 (2.2%)
ADHD: n (%)	9,854 (5.1%)	10,785 (4.8%)	1,748 (3.3%)	1,751 (3.0%)	28,148 (4.1%)	46,600 (4.3%)	2,032 (1.8%)	2,112 (1.5%)
Delirium: n (%)	2,147 (1.1%)	2,521 (1.1%)	346 (0.7%)	385 (0.7%)	7,906 (1.1%)	14,256 (1.3%)	638 (0.6%)	830 (0.6%)
Bipolar disorder: n (%)	462 (0.2%)	631 (0.3%)	108 (0.2%)	133 (0.2%)	1,341 (0.2%)	2,271 (0.2%)	135 (0.1%)	160 (0.1%)
Other psychiatric disorder: n (%)	10,427 (5.4%)	11,649 (5.1%)	1,938 (3.7%)	1,838 (3.1%)	28,446 (4.1%)	45,850 (4.2%)	2,047 (1.8%)	2,071 (1.5%)
Psychiatric hospitalization: mean (sd)	0.03 (0.24)	0.03 (0.23)	0.01 (0.19)	0.01 (0.15)	0.03 (0.21)	0.03 (0.22)	0.01 (0.13)	0.01 (0.10)
Psychiatric office visit: mean (sd)	2.09 (6.98)	1.95 (6.87)	1.67 (4.99)	1.44 (4.91)	2.15 (8.69)	2.22 (10.17)	1.39 (4.77)	1.20 (4.26)
Anticonvulsants/antiepileptic drugs: n (%)	12,417 (6.4%)	14,628 (6.4%)	4,079 (7.8%)	4,637 (7.9%)	48,716 (7.1%)	81,990 (7.5%)	9,962 (8.9%)	13,331 (9.5%)
SSRIs (without sertraline): n (%)	32,948 (17.0%)	38,648 (17.0%)	2,048 (3.9%)	1,769 (3.0%)	112,003 (16.3%)	178,579 (16.3%)	4,034 (3.6%)	4,167 (3.0%)
SNRIs: n (%)	9,820 (5.1%)	11,377 (5.0%)	1,049 (2.0%)	1,033 (1.8%)	34,890 (5.1%)	53,156 (4.9%)	2,162 (1.9%)	2,516 (1.8%)
Tricyclic antidepressants: n (%)	4,542 (2.3%)	5,086 (2.2%)	1,440 (2.7%)	1,775 (3.0%)	17,234 (2.5%)	27,062 (2.5%)	3,667 (3.3%)	4,735 (3.4%)
Antipsychotics: n (%)	8,166 (4.2%)	8,990 (4.0%)	2,816 (5.4%)	2,852 (4.8%)	30,926 (4.5%)	47,921 (4.4%)	6,631 (5.9%)	7,983 (5.7%)
Benzodiazepines: n (%)	40,742 (21.1%)	45,319 (20.0%)	11,359 (21.7%)	11,765 (20.0%)	148,144 (21.5%)	226,219 (20.7%)	24,896 (22.1%)	30,535 (21.7%)
Non-BZD sedative hypnotics: n (%)	21,792 (11.3%)	24,340 (10.7%)	6,167 (11.8%)	6,380 (10.8%)	77,066 (11.2%)	116,714 (10.7%)	14,198 (12.6%)	17,534 (12.5%)
Outpatient visits: mean (sd)	0.22 (0.47)	0.23 (0.50)	0.22 (0.47)	0.23 (0.48)	0.19 (0.49)	0.20 (0.45)	0.17 (0.41)	0.15 (0.43)
Inpatient hospitalization: mean (sd)	0.00 (0.10)	0.01 (0.12)	0.00 (0.12)	0.00 (0.10)	0.00 (0.01)	0.00 (0.01)	0.00 (0.01)	0.00 (0.01)
ED visit: mean (sd)	0.30 (1.07)	0.28 (0.88)	0.18 (0.69)	0.16 (0.61)	0.41 (1.32)	0.42 (1.31)	0.22 (0.82)	0.21 (0.74)
Number of distinct medication prescriptions: mean (sd)	4.43 (4.21)	4.37 (4.16)	5.78 (4.30)	6.02 (4.34)	4.76 (4.53)	4.81 (4.53)	6.67 (4.80)	7.17 (4.98)
Combined Comorbidity Score: mean (sd)	0.32 (1.06)	0.30 (1.08)	0.26 (0.97)	0.24 (1.03)	0.35 (1.13)	0.36 (1.20)	0.24 (0.97)	0.27 (1.04)

Table J- Characteristics of patients included in the evaluation of comparative outcomes for *alendronate* between authorized generic (AG) and generic initiators and switchers after 1:1 propensity score matching in each database

	Optum				Truven			
	AG initiators	Generic initiators	AG switchers	Generic switchers	AG initiators	Generic initiators	AG switchers	Generic switchers
**Cohort selection steps**								
Total number of patients filling prescriptions for the version of interest after 6-month continuous enrollment	**103,017**		**140,057**		**880,618**		**876,577**	
Patients meeting new drug use restriction	**67,853**		**60,187**		**443,069**		**338,291**	
Patients meeting prior brand-name use requirement (to define switchers, applicable for switchers comparison only)	**NA**		**33,931**		**NA**		**164,688**	
Total eligible by exposure group	**2,439**	**65,414**	**6.343**	**27,588**	**11,963**	**431,106**	**29,989**	**134,699**
**1:1 PS matched**	**2,433**	**2,433**	**6,332**	**6,332**	**11,963**	**11,963**	**29,985**	**29,985**
**Baseline characteristics**								
Age: mean (sd)	60 (10.12)	61 (9.70)	62 (9.56)	62 (9.43)	63.48 (11.93)	63.34 (11.75)	66.84 (11.82)	66.79 (11.55)
Gender								
MALE: n (%)	217 (8.9%)	190 (7.8%)	508 (8.0%)	530 (8.4%)	1,233 (10.3%)	1,216 (10.2%)	2,540 (8.5%)	2,390 (8.0%)
FEMALE: n (%)	2215 (91.0%)	2242 (92.1%)	5,823 (92.0%)	5,802 (91.6%)	10,730 (89.7%)	10,747 (89.8%)	27,445 (91.5%)	27,595 (92.0%)
UNKNOWN: n (%)	1 (0.0%)	1 (0.0%)	1 (0.0%)	0 (0.0%)	0 (0.0%)	0 (0.0%)	0 (0.0%)	0 (0.0%)
Region								
Northeast: n (%)	421 (17.3%)	395 (16.2%)	1,435 (22.7%)	1,435 (22.7%)	2,315 (19.4%)	2,301 (19.2%)	4,856 (16.2%)	4,986 (16.6%)
Midwest: n (%)	566 (23.3%)	860 (35.3%)	1,826 (28.8%)	1,756 (27.7%)	4,017 (33.6%)	4,083 (34.1%)	10,912 (36.4%)	10,830 (36.1%)
South: n (%)	1231 (50.6%)	1028 (42.3%)	2,263 (35.7%)	2,343 (37.0%)	3,810 (31.8%)	3,795 (31.7%)	8,715 (29.1%)	8,682 (29.0%)
West: n (%)	215 (8.8%)	149 (6.1%)	805 (12.7%)	795 (12.6%)	1,696 (14.2%)	1,669 (14.0%)	5,374 (17.9%)	5,365 (17.9%)
Unknown/other: n (%)	0 (0.0%)	1 (0.0%)	3 (0.0%)	3 (0.0%)	125 (1.0%)	115 (1.0%)	128 (0.4%)	122 (0.4%)
Osteoporosis: n (%)	976 (40.1%)	959 (39.4)	1,638 (25.9%)	1,640 (25.9%)	3,469 (29.0%)	3,347 (28.0%)	4,264 (14.2%)	4,202 (14.0%)
Kyphosis: n (%)	25 (1.0%)	17 (0.7%)	58 (0.9%)	53 (0.8%)	99 (0.8%)	87 (0.7%)	172 (0.6%)	156 (0.5%)

Vertebral facture: n (%)	48 (2.0%)	31 (1.3%)	76 (1.2%)	74 (1.2%)	335 (2.8%)	324 (2.7%)	318 (1.1%)	325 (1.1%)
Humerus fracture: n (%)	11 (0.5%)	10 (0.4%)	20 (0.3%)	23 (0.4%)	69 (0.6%)	74 (0.6%)	113 (0.4%)	110 (0.4%)
Wrist fracture: n (%)	20 (0.8%)	14 (0.6%)	43 (0.7%)	45 (0.7%)	135 (1.1%)	124 (1.0%)	196 (0.7%)	188 (0.6%)
Hip fracture: n (%)	19 (0.8%)	11 (0.5%)	30 (0.5%)	36 (0.6%)	163 (1.4%)	145 (1.2%)	179 (0.6%)	176 (0.6%)
Pelvis fracture: n (%)	9 (0.4%)	6 (0.2%)	14 (0.2%)	8 (0.1%)	68 (0.6%)	56 (0.5%)	76 (0.3%)	94 (0.3%)
Any other fractures: n (%)	110 (4.5%)	113 (4.6%)	190 (3.0%)	174 (2.7%)	541 (4.5%)	526 (4.4%)	819 (2.7%)	806 (2.7%)
Alzheimer disease or other dementia: n (%)	48 (2.0%)	48 (2.0%)	111 (1.8%)	95 (1.5%)	239 (2.0%)	244 (2.0%)	654 (2.2%)	561 (1.9%)
Asthma or chronic obstructive pulmonary disease: n (%)	229 (9.4%)	247 (10.2%)	463 (7.3%)	442 (7.0%)	823 (6.9%)	791 (6.6%)	1,852 (6.2%)	1,768 (5.9%)
Cataracts: n (%)	211 (8.7%)	231 (9.5%)	637 (10.1%)	610 (9.6%)	892 (7.5%)	869 (7.3%)	2,551 (8.5%)	2,451 (8.2%)
Crohn disease or gastroenteritis: n (%)	55 (2.3%)	68 (2.8%)	131 (2.1%)	127 (2.0%)	238 (2.0%)	206 (1.7%)	502 (1.7%)	479 (1.6%)
Depression: n (%)	65 (2.7%)	63 (2.6%)	161 (2.5%)	159 (2.5%)	197 (1.6%)	191 (1.6%)	431 (1.4%)	404 (1.3%)
Diabetes mellitus: n (%)	253 (10.4%)	246 (10.1%)	571 (9.0%)	584 (9.2%)	1,322 (11.1%)	1,245 (10.4%)	2,714 (9.1%)	2,480 (8.3%)
History of falls, syncope, or gait abnormality: n (%)	172 (7.1%)	178 (7.3%)	401 (6.3%)	402 (6.3%)	648 (5.4%)	630 (5.3%)	1,272 (4.2%)	1,214 (4.0%)
Hyperthyroidism: n (%)	29 (1.2%)	32 (1.3%)	79 (1.2%)	83 (1.3%)	121 (1.0%)	123 (1.0%)	214 (0.7%)	180 (0.6%)
Hyperparathyroidism: n (%)	23 (0.9%)	36 (1.5%)	42 (0.7%)	44 (0.7%)	65 (0.5%)	48 (0.4%)	137 (0.5%)	138 (0.5%)
Ischemic stroke: n (%)	18 (0.7%)	24 (1.0%)	38 (0.6%)	45 (0.7%)	106 (0.9%)	94 (0.8%)	186 (0.6%)	156 (0.5%)
Liver disease: n (%)	64 (2.6%)	57 (2.3%)	119 (1.9%)	113 (1.8%)	226 (1.9%)	214 (1.8%)	340 (1.1%)	308 (1.0%)
Malignant neoplasm: n (%)	212 (8.7%)	230 (9.5%)	665 (10.5%)	644 (10.2%)	1,106 (9.2%)	1,061 (8.9%)	2,869 (9.6%)	2,733 (9.1%)
Overweight or obese: n (%)	54 (2.2%)	43 (1.8%)	87 (1.4%)	82 (1.3%)	153 (1.3%)	151 (1.3%)	138 (0.5%)	116 (0.4%)
Parkinson disease: n (%)	12 (0.5%)	14 (0.6%)	26 (0.4%)	24 (0.4%)	57 (0.5%)	74 (0.6%)	164 (0.5%)	130 (0.4%)
Renal disease: n (%)	24 (1.0%)	20 (0.8%)	52 (0.8%)	67 (1.1%)	95 (0.8%)	85 (0.7%)	184 (0.6%)	153 (0.5%)
Rheumatoid arthritis: n (%)	120 (4.9%)	141 (5.8%)	231 (3.6%)	198 (3.1%)	458 (3.8%)	454 (3.8%)	865 (2.9%)	773 (2.6%)
Oral glucocorticoids: n (%)	501 (20.6%)	479 (19.7%)	1,185 (18.7%)	1,143 (18.1%)	2,629 (22.0%)	2,477 (20.7%)	6,038 (20.1%)	5,828 (19.4%)
Anticonvulsants/antiepileptic drugs: n (%)	190 (7.8%)	193 (7.9%)	415 (6.6%)	403 (6.4%)	995 (8.3%)	941 (7.9%)	2,270 (7.6%)	2,131 (7.1%)
Benzodiazepines: n (%)	402 (16.5%)	456 (18.7%)	752 (11.9%)	736 (11.6%)	1,737 (14.5%)	1,666 (13.9%)	3,909 (13.0%)	3,747 (12.5%)
SSRIs: n (%)	391 (16.1%)	389 (16.0%)	979 (15.5%)	990 (15.6%)	1,980 (16.6%)	1,990 (16.6%)	4,724 (15.8%)	4,428 (14.8%)
Beta-blockers: n (%)	341 (14.0%)	291 (12.0%)	1,130 (17.8%)	1,085 (17.1%)	2,493 (20.8%)	2,416 (20.2%)	6,857 (22.9%)	6,735 (22.5%)
Proton pump inhibitors: n (%)	330 (13.6%)	310 (12.7%)	811 (12.8%)	772 (12.2%)	2,265 (18.9%)	2,197 (18.4%)	5,647 (18.8%)	5,430 (18.1%)
Opioids: n (%)	685 (28.2%)	669 (27.5%)	1,459 (23.0%)	1,394 (22.0%)	3,685 (30.8%)	3,535 (29.5%)	7,609 (25.4%)	7,300 (24.3%)

Other anti-osteoporosis medications (without alendronate): n (%)	295 (12.1%)	308 (12.7%)	198 (3.1%)	186 (2.9%)	2,458 (20.5%)	2,489 (20.8%)	832 (2.8%)	795 (2.7%)
Outpatient visits (occurrence): mean (sd)	0.36 (0.53)	0.32 (0.50)	0.26 (0.48)	0.25 (0.47)	0.27 (0.51)	0.27 (0.49)	0.17 (0.45)	0.16 (0.41)
Inpatient hospitalization (occurrence): mean (sd)	0.01 (0.09)	0.00 (0.04)	0.01 (0.15)	0.01 (0.16)	0.00 (0.01)	0.00 (0.01)	0.00 (0.00)	0.00 (0.00)
ED visit (occurrence): mean (sd)	0.16 (0.57)	0.14 (0.49)	0.12 (0.55)	0.12 (0.51)	0.24 (0.76)	0.24 (0.93)	0.17 (0.63)	0.16 (0.62)
Number of distinct medication prescriptions: mean (sd)	6.68 (4.72)	6.70 (4.66)	6.28 (4.37)	6.18 (4.37)	7.32 (4.90)	7.19 (5.19)	7.01 (4.64)	6.81 (4.52)
Combined Comorbidity Score: mean (sd)	0.31 (1.29)	0.31 (1.28)	0.24 (1.17)	0.23 (1.17)	0.27 (1.17)	0.26 (1.17)	0.22 (1.07)	0.19 (1.02)

Table K- Characteristics of patients included in the evaluation of comparative outcomes for *amlodipine* between authorized generic (AG) and generic initiators and switchers after 1:1 propensity score matching in each database

	Optum				Truven			
	AG initiators	Generic initiators	AG switchers	Generic switchers	AG initiators	Generic initiators	AG switchers	Generic switchers
**Cohort selection steps**								
Total number of patients filling prescriptions for the version of interest after 6-month continuous enrollment	476,857		668,221		**2,055,563**		**3,425,397**	
Patients meeting new drug use restriction	379,901		192,321		**2,022,728**		**862,070**	
Patients meeting prior brand-name use requirement (to define switchers, applicable for switchers comparison only)	NA		87,990		**N/A**		**324,079**	
Total eligible by exposure group	73,973	305,928	41,217	46,773	**474,066**	**1,548,662**	**192,720**	**131,359**
**1:1 PS matched**	73,853	73,853	35,004	35,004	**461,045**	**461,045**	**116,521**	**116,521**
**Baseline characteristics**								
Age: mean (sd)	55 (12.37)	55 (11.82)	58 (10.86)	58 (10.68)	60.16 (14.27)	60.09 (14.23)	63.18 (12.74)	63.16 (13.28)
Gender								
MALE: n (%)	41,374 (56.0%)	41,591 (56.3%)	18,954 (54.1%)	18,987 (54.2%)	231,251 (50.2%)	231,779 (50.3%)	56,127 (48.2%)	56,315 (48.3%)
FEMALE: n (%)	32,474 (44.0%)	32,260 (43.7%)	16,046 (45.8%)	16,013 (45.7%)	229,794 (49.8%)	229,266 (49.7%)	60,394 (51.8%)	60,206 (51.7%)
UNKNOWN: n (%)	5 (0.0%)	2 (0.0%)	4 (0.0%)	4 (0.0%)	0 (0.0%)	0 (0.0%)	0 (0.0%)	0 (0.0%)
Region								
Northeast: n (%)	5,634 (7.6%)	5,605 (7.6%)	4,219 (12.1%)	4,145 (11.8%)	58,444 (12.7%)	58,242 (12.6%)	13,648 (11.7%)	13,846 (11.9%)
Midwest: n (%)	12,007 (16.3%)	11,768 (15.9%)	9,628 (27.5%)	9,662 (27.6%)	86,981 (18.9%)	86,388 (18.7%)	36,426 (31.3%)	36,817 (31.6%)
South: n (%)	43,811 (59.3%)	44,237 (59.9%)	17,345 (49.6%)	17,232 (49.2%)	189,519 (41.1%)	193,677 (42.0%)	52,509 (45.1%)	51,983 (44.6%)
West: n (%)	12,358 (16.7%)	12,209 (16.5%)	3,790 (10.8%)	3,950 (11.3%)	121,725 (26.4%)	118,620 (25.7%)	13,466 (11.6%)	13,426 (11.5%)
Unknown/other: n (%)	43 (0.1%)	34 (0.0%)	22 (0.1%)	15 (0.0%)	4,376 (0.9%)	4,118 (0.9%)	472 (0.4%)	449 (0.4%)
Hyperlipidemia: n (%)	32,964 (44.6%)	32,334 (43.8%)	16,137 (46.1%)	16,153 (46.1%)	147,351 (32.0%)	146,042 (31.7%)	27,277 (23.4%)	27,433 (23.5%)
Diabetes mellitus: n (%)	15,072 (20.4%)	14,622 (19.8%)	7,656 (21.9%)	7,648 (21.8%)	95,262 (20.7%)	93,607 (20.3%)	23,208 (19.9%)	23,593 (20.2%)

Hypertension: n (%)	54,318 (73.5%)	53,875 (72.9%)	25,023 (71.5%)	25,083 (71.7%)	294,395 (63.9%)	293,231 (63.6%)	61,749 (53.0%)	61,963 (53.2%)
Myocardial Infarction: n (%)	999 (1.4%)	921 (1.2%)	206 (0.6%)	206 (0.6%)	6,536 (1.4%)	6,259 (1.4%)	710 (0.6%)	714 (0.6%)
Angina: n (%)	2,698 (3.7%)	2,579 (3.5%)	975 (2.8%)	992 (2.8%)	16,002 (3.5%)	15,503 (3.4%)	2,853 (2.4%)	2,889 (2.5%)
Heart failure: n (%)	2,613 (3.5%)	2,478 (3.4%)	1,034 (3.0%)	1,055 (3.0%)	19,248 (4.2%)	18,882 (4.1%)	3,985 (3.4%)	4,034 (3.5%)
Other forms of chronic heart diseases: n (%)	7,756 (10.5%)	7,504 (10.2%)	4,416 (12.6%)	4,429 (12.7%)	50,190 (10.9%)	49,256 (10.7%)	13,459 (11.6%)	13,648 (11.7%)
Cerebrovascular accident: n (%)	2,128 (2.9%)	2,046 (2.8%)	639 (1.8%)	663 (1.9%)	15,665 (3.4%)	15,328 (3.3%)	2,317 (2.0%)	2,395 (2.1%)
Venous thromboembolism: n (%)	1,134 (1.5%)	1,132 (1.5%)	368 (1.1%)	375 (1.1%)	7,395 (1.6%)	7,248 (1.6%)	1,300 (1.1%)	1,312 (1.1%)
Atrial fibrillation: n (%)	2,399 (3.2%)	2,321 (3.1%)	1,095 (3.1%)	1,104 (3.2%)	19,592 (4.2%)	18,882 (4.1%)	4,087 (3.5%)	4,058 (3.5%)
Overweight or obese: n (%)	5,412 (7.3%)	5,190 (7.0%)	1,549 (4.4%)	1,544 (4.4%)	25,593 (5.6%)	24,427 (5.3%)	1,197 (1.0%)	1,206 (1.0%)
Tobacco use: n (%)	3,894 (5.3%)	3,821 (5.2%)	1,114 (3.2%)	1,144 (3.3%)	16,995 (3.7%)	16,169 (3.5%)	983 (0.8%)	962 (0.8%)
Alcohol abuse or dependence: n (%)	844 (1.1%)	833 (1.1%)	212 (0.6%)	213 (0.6%)	4,143 (0.9%)	3,917 (0.8%)	335 (0.3%)	335 (0.3%)
Renal disease: n (%)	2,572 (3.5%)	2,424 (3.3%)	999 (2.9%)	1,009 (2.9%)	18,439 (4.0%)	17,815 (3.9%)	2,492 (2.1%)	2,568 (2.2%)
Liver disease: n (%)	2,334 (3.2%)	2,270 (3.1%)	838 (2.4%)	809 (2.3%)	11,279 (2.4%)	11,036 (2.4%)	1,712 (1.5%)	1,703 (1.5%)
Aspirin: n (%)	36 (0.0%)	26 (0.0%)	24 (0.1%)	26 (0.1%)	3,585 (0.8%)	3,430 (0.7%)	952 (0.8%)	974 (0.8%)
Antiplatelets: n (%)	3,145 (4.3%)	3,010 (4.1%)	2,277 (6.5%)	2,287 (6.5%)	23,956 (5.2%)	23,605 (5.1%)	9,844 (8.4%)	9,913 (8.5%)
Statins: n (%)	24,136 (32.7%)	23,705 (32.1%)	14,619 (41.8%)	14,596 (41.7%)	160,457 (34.8%)	158,486 (34.4%)	49,919 (42.8%)	50,323 (43.2%)
Other lipid lowering agents: n (%)	6,277 (8.5%)	6,193 (8.4%)	5,494 (15.7%)	5,485 (15.7%)	35,343 (7.7%)	34,632 (7.5%)	17,706 (15.2%)	18,091 (15.5%)
Insulin preparations: n (%)	3,096 (4.2%)	2,965 (4.0%)	1,413 (4.0%)	1,439 (4.1%)	13,289 (2.9%)	13,209 (2.9%)	3,247 (2.8%)	3,295 (2.8%)
Oral hypoglycemic agents: n (%)	10,011 (13.6%)	9,775 (13.2%)	6,016 (17.2%)	5,991 (17.1%)	69,163 (15.0%)	68,011 (14.8%)	21,148 (18.1%)	21,626 (18.6%)
ACE inhibitors: n (%)	25,788 (34.9%)	25,584 (34.6%)	10,785 (30.8%)	10,759 (30.7%)	161,775 (35.1%)	160,192 (34.7%)	34,790 (29.9%)	35,051 (30.1%)
ARBs: n (%)	17,892 (24.2%)	17,637 (23.9%)	9,747 (27.8%)	9,747 (27.8%)	108,698 (23.6%)	108,046 (23.4%)	32,634 (28.0%)	32,731 (28.1%)
Calcium channel blockers (without amlodipine): n (%)	4,190 (5.7%)	4,104 (5.6%)	503 (1.4%)	527 (1.5%)	31,580 (6.8%)	32,757 (7.1%)	2,111 (1.8%)	2,156 (1.9%)
Diuretics: n (%)	27,118 (36.7%)	26,743 (36.2%)	17,154 (49.0%)	17,166 (49.0%)	180,753 (39.2%)	178,872 (38.8%)	58,904 (50.6%)	59,152 (50.8%)
Beta-blockers: n (%)	17,544 (23.8%)	17,015 (23.0%)	11,887 (34.0%)	11,947 (34.1%)	130,454 (28.3%)	127,636 (27.7%)	42,613 (36.6%)	42,890 (36.8%)
Anticoagulants: n (%)	2,070 (2.8%)	2,019 (2.7%)	1,392 (4.0%)	1,395 (4.0%)	17,975 (3.9%)	17,698 (3.8%)	6,244 (5.4%)	6,307 (5.4%)
NSAIDs: n (%)	10,521 (14.2%)	10,387 (14.1%)	4,787 (13.7%)	4,786 (13.7%)	66,969 (14.5%)	65,347 (14.2%)	16,586 (14.2%)	16,721 (14.4%)
Coxibs: n (%)	1,450 (2.0%)	1,434 (1.9%)	1,008 (2.9%)	1,025 (2.9%)	8,918 (1.9%)	8,836 (1.9%)	4,103 (3.5%)	4,063 (3.5%)

Outpatient visits: mean (sd)	0.15 (0.42)	0.15 (0.47)	0.13 (0.36)	0.13 (0.38)	0.11 (0.36)	0.11 (0.35)	0.08 (0.30)	0.08 (0.31)
Inpatient hospitalization: mean (sd)	0.01 (0.16)	0.01 (0.16)	0.01 (0.11)	0.01 (0.11)	0.00 (0.02)	0.00 (0.02)	0.00 (0.01)	0.00 (0.01)
ED visit: mean (sd)	0.28 (0.79)	0.27 (0.74)	0.15 (0.56)	0.15 (0.56)	0.37 (1.12)	0.36 (1.03)	0.21 (0.72)	0.21 (0.72)
Number of distinct medication prescriptions: mean (sd)	5.52 (4.63)	5.42 (4.67)	7.27 (4.49)	7.29 (4.59)	5.96 (4.84)	5.88 (4.82)	7.94 (4.83)	7.96 (4.85)
Combined Comorbidity Score: mean (sd)	0.13 (1.63)	0.11 (1.61)	-0.02 (1.37)	-0.01 (1.39)	0.25 (1.64)	0.23 (1.61)	0.11 (1.32)	0.12 (1.32)

Table L- Characteristics of patients included in the evaluation of comparative outcomes for *amlodipine-benazepril* between authorized generic (AG) and generic initiators and switchers after 1:1 propensity score matching in each database.

	Optum				Truven			
	AG initiators	Generic initiators	AG switchers	Generic switchers	AG initiators	Generic initiators	AG switchers	Generic switchers
**Cohort selection steps**								
Total number of patients filling prescriptions for the version of interest after 6-month continuous enrollment	72,221		128,725		**665,841**		**665,928**	
Patients meeting new drug use restriction	43,323		57,074		**236,740**		**240,935**	
Patients meeting prior brand-name use requirement (to define switchers, applicable for switchers comparison only)	NA		43,217		**N/A**		**186,425**	
Total eligible by exposure group	10,953	32,370	6,056	37,161	**47,375**	**175,465**	**29,713**	**156,712**
**1:1 PS matched**	10,941	10,941	6,034	6,034	**47,375**	**47,375**	**29,652**	**29,652**
**Baseline characteristics**								
Age: mean (sd)	52.86 (11.39)	52.83 (11.50)	55 (10.25)	55 (10.23)	54.58 (12.39)	54.42 (12.43)	58.14 (11.99)	58.06 (11.88)
Gender								
MALE: n (%)	6,537 (59.7%)	6,626 (60.6%)	3,931 (65.1%)	3,926 (65.1%)	26,949 (56.9%)	27,314 (57.7%)	17,765 (59.9%)	17,878 (60.3%)
FEMALE: n (%)	4,403 (40.2%)	4,314 (39.4%)	2,103 (34.9%)	2,108 (34.9%)	20,426 (43.1%)	20,061 (42.3%)	11,887 (40.1%)	11,774 (39.7%)
UNKNOWN: n (%)	1 (0.0%)	1 (0.0%)	0 (0.0%)	0 (0.0%)	0 (0.0%)	0 (0.0%)	0 (0.0%)	0 (0.0%)
Region								
Northeast: n (%)	615 (5.6%)	584 (5.3%)	379 (6.3%)	382 (6.3%)	6,684 (14.1%)	6,580 (13.9%)	3,161 (10.7%)	3,045 (10.3%)
Midwest: n (%)	1,713 (15.7%)	1,739 (15.9%)	1,223 (20.3%)	1,267 (21.0%)	11,606 (24.5%)	11,718 (24.7%)	6,530 (22.0%)	6,208 (20.9%)
South: n (%)	7,859 (71.8%)	7,793 (71.2%)	3,970 (65.8%)	3,924 (65.0%)	23,753 (50.1%)	23,809 (50.3%)	16,947 (57.2%)	17,435 (58.8%)
West: n (%)	741 (6.8%)	816 (7.5%)	460 (7.6%)	459 (7.6%)	4,052 (8.6%)	4,063 (8.6%)	2,642 (8.9%)	2,601 (8.8%)
Unknown/other: n (%)	13 (0.1%)	9 (0.1%)	2 (0.0%)	2 (0.0%)	1,280 (2.7%)	1,205 (2.5%)	372 (1.3%)	363 (1.2%)
Hyperlipidemia: n (%)	4,066 (37.2%)	3,930 (35.9%)	2,697 (44.7%)	2,672 (44.3%)	13,726 (29.0%)	13,298 (28.1%)	9,311 (31.4%)	9,191 (31.0%)
Diabetes mellitus: n (%)	1,920 (17.5%)	1,831 (16.7%)	1,332 (22.1%)	1,331 (22.1%)	7,518 (15.9%)	7,265 (15.3%)	6,380 (21.5%)	6,068 (20.5%)
Hypertension: n (%)	7,648 (69.9%)	7,442 (68.0%)	4,205 (69.7%)	4,133 (68.5%)	28,929 (61.1%)	28,519 (60.2%)	16,882 (56.9%)	16,649 (56.1%)

Myocardial Infarction: n (%)	51 (0.5%)	52 (0.5%)	13 (0.2%)	15 (0.2%)	188 (0.4%)	176 (0.4%)	68 (0.2%)	58 (0.2%)
Angina: n (%)	198 (1.8%)	181 (1.7%)	93 (1.5%)	101 (1.7%)	786 (1.7%)	751 (1.6%)	360 (1.2%)	358 (1.2%)
Heart failure: n (%)	146 (1.3%)	162 (1.5%)	84 (1.4%)	77 (1.3%)	656 (1.4%)	649 (1.4%)	382 (1.3%)	365 (1.2%)
Other forms of chronic heart diseases: n (%)	658 (6.0%)	645 (5.9%)	430 (7.1%)	439 (7.3%)	2,491 (5.3%)	2,393 (5.1%)	1,858 (6.3%)	1,805 (6.1%)
Cerebrovascular accident: n (%)	170 (1.6%)	159 (1.5%)	68 (1.1%)	73 (1.2%)	670 (1.4%)	651 (1.4%)	295 (1.0%)	300 (1.0%)
Venous thromboembolism: n (%)	63 (0.6%)	59 (0.5%)	46 (0.8%)	30 (0.5%)	321 (0.7%)	308 (0.7%)	194 (0.7%)	181 (0.6%)
Atrial fibrillation: n (%)	137 (1.3%)	138 (1.3%)	117 (1.9%)	136 (2.3%)	681 (1.4%)	661 (1.4%)	593 (2.0%)	557 (1.9%)
Overweight or obese: n (%)	663 (6.1%)	624 (5.7%)	301 (5.0%)	281 (4.7%)	1,738 (3.7%)	1,710 (3.6%)	804 (2.7%)	807 (2.7%)
Tobacco use: n (%)	510 (4.7%)	494 (4.5%)	222 (3.7%)	204 (3.4%)	1,359 (2.9%)	1,314 (2.8%)	460 (1.6%)	414 (1.4%)
Alcohol abuse or dependence: n (%)	80 (0.7%)	71 (0.6%)	28 (0.5%)	26 (0.4%)	315 (0.7%)	335 (0.7%)	98 (0.3%)	99 (0.3%)
Renal disease: n (%)	138 (1.3%)	140 (1.3%)	96 (1.6%)	81 (1.3%)	509 (1.1%)	484 (1.0%)	359 (1.2%)	339 (1.1%)
Liver disease: n (%)	204 (1.9%)	178 (1.6%)	118 (2.0%)	116 (1.9%)	722 (1.5%)	746 (1.6%)	391 (1.3%)	390 (1.3%)
Aspirin: n (%)	1 (0.0%)	0 (0.0%)	3 (0.0%)	1 (0.0%)	193 (0.4%)	178 (0.4%)	146 (0.5%)	142 (0.5%)
Antiplatelets: n (%)	209 (1.9%)	196 (1.8%)	262 (4.3%)	256 (4.2%)	1,202 (2.5%)	1,180 (2.5%)	1,400 (4.7%)	1,318 (4.4%)
Statins: n (%)	2,184 (20.0%)	2,122 (19.4%)	2,336 (38.7%)	2,329 (38.6%)	10,298 (21.7%)	9,851 (20.8%)	12,001 (40.5%)	11,644 (39.3%)
Other lipid lowering agents: n (%)	612 (5.6%)	594 (5.4%)	731 (12.1%)	768 (12.7%)	2,492 (5.3%)	2,385 (5.0%)	3,716 (12.5%)	3,585 (12.1%)
Insulin preparations: n (%)	245 (2.2%)	220 (2.0%)	243 (4.0%)	264 (4.4%)	713 (1.5%)	734 (1.5%)	686 (2.3%)	670 (2.3%)
Oral hypoglycemic agents: n (%)	1,117 (10.2%)	1,076 (9.8%)	1,087 (18.0%)	1,093 (18.1%)	4,939 (10.4%)	4,753 (10.0%)	5,950 (20.1%)	5,644 (19.0%)
ACE inhibitors: n (%)	2,510 (22.9%)	2,450 (22.4%)	164 (2.7%)	160 (2.7%)	12,383 (26.1%)	12,012 (25.4%)	28,690 (96.8%)	28,620 (96.5%)
ARBs: n (%)	1,075 (9.8%)	1,073 (9.8%)	529 (8.8%)	524 (8.7%)	4,581 (9.7%)	4,470 (9.4%)	2,414 (8.1%)	2,403 (8.1%)
Calcium channel blockers (without amlodipine-benazepril): n (%)	515 (4.7%)	497 (4.5%)	81 (1.3%)	82 (1.4%)	2,301 (4.9%)	2,188 (4.6%)	385 (1.3%)	378 (1.3%)
Diuretics: n (%)	2,501 (22.9%)	2,430 (22.2%)	2,082 (34.5%)	2,120 (35.1%)	11,214 (23.7%)	10,703 (22.6%)	10,682 (36.0%)	10,401 (35.1%)
Beta-blockers: n (%)	1,616 (14.8%)	1,577 (14.4%)	1,442 (23.9%)	1,421 (23.5%)	8,168 (17.2%)	7,852 (16.6%)	7,795 (26.3%)	7,629 (25.7%)
Anticoagulants: n (%)	125 (1.1%)	126 (1.2%)	131 (2.2%)	137 (2.3%)	740 (1.6%)	694 (1.5%)	831 (2.8%)	749 (2.5%)
NSAIDs: n (%)	1,358 (12.4%)	1,338 (12.2%)	851 (14.1%)	835 (13.8%)	5,935 (12.5%)	5,766 (12.2%)	4,398 (14.8%)	4,180 (14.1%)
Coxibs: n (%)	168 (1.5%)	162 (1.5%)	139 (2.3%)	143 (2.4%)	685 (1.4%)	624 (1.3%)	827 (2.8%)	764 (2.6%)
Outpatient visits: mean (sd)	0.13 (0.35)	0.13 (0.36)	0.11 (0.34)	0.11 (0.33)	0.12 (0.36)	0.12 (0.35)	0.10 (0.31)	0.09 (0.31)
Inpatient hospitalization: mean (sd)	0.00 (0.06)	0.00 (0.07)	0.00 (0.06)	0.00 (0.07)	0.00 (0.00)	0.00 (0.00)	0.00 (0.00)	0.00 (0.00)
ED visit: mean (sd)	0.19 (0.56)	0.18 (0.59)	0.12 (0.46)	0.12 (0.47)	0.25 (0.85)	0.24 (0.91)	0.16 (0.62)	0.15 (0.72)

Number of distinct medication prescriptions: mean (sd)	3.76 (3.85)	3.64 (3.72)	5.78 (4.04)	5.78 (3.98)	3.96 (4.06)	3.80 (3.95)	6.12 (4.11)	5.97 (4.04)
Combined Comorbidity Score: mean (sd)	-0.26 (1.01)	-0.26 (1.00)	-0.25 (1.03)	-0.25 (1.03)	-0.23 (1.01)	-0.23 (0.98)	-0.18 (0.99)	-0.19 (0.95)

Table M- Characteristics of patients included in the evaluation of comparative outcomes for *calcitonin salmon* between authorized generic (AG) and generic initiators and switchers after 1:1 propensity score matching in each database

	Optum				Truven			
	**AG initiators**	**Generic initiators**	**AG switchers**	**Generic switchers**	**AG initiators**	**Generic initiators**	**AG switchers**	**Generic switchers**
**Cohort selection steps**								
Total number of patients filling prescriptions for the version of interest after 6-month continuous enrollment	**5,584**		**6,651**		**55,964**		**55,961**	
Patients meeting new drug use restriction	**3,679**		**4,236**		**33,798**		**34,059**	
Patients meeting prior brand-name use requirement (to define switchers, applicable for switchers comparison only)	**NA**		**1,879**		**NA**		**15,577**	
Total eligible by exposure group	**1,078**	**2,601**	**468**	**1,411**	**7,421**	**22,530**	**2,896**	**12,681**
**1:1 PS matched**	**1,054**	**1,054**	**458**	**458**	**7,420**	**7,420**	**2,892**	**2,892**
**Baseline characteristics**								
Age: mean (sd)	59 (13.12)	59 (13.25)	65 (11.49)	65 (10.52)	67.25 (15.23)	66.96 (14.98)	70.03 (13.15)	69.90 (12.33)
Gender								
MALE: n (%)	181 (17.2%)	191 (18.1%)	42 (9.2%)	44 (9.6%)	1,243 (16.8%)	1,265 (17.0%)	244 (8.4%)	252 (8.7%)
FEMALE: n (%)	873 (82.8%)	863 (81.9%)	416 (90.8%)	414 (90.4%)	6,177 (83.2%)	6,155 (83.0%)	2,648 (91.6%)	2,640 (91.3%)
UNKNOWN: n (%)	0 (0.0%)	0 (0.0%)	0 (0.0%)	0 (0.0%)	0 (0.0%)	0 (0.0%)	0 (0.0%)	0 (0.0%)
Region								
Northeast: n (%)	96 (9.1%)	113 (10.7%)	51 (11.1%)	48 (10.5%)	1,531 (20.6%)	1,526 (20.6%)	486 (16.8%)	490 (16.9%)
Midwest: n (%)	256 (24.3%)	241 (22.9%)	141 (30.8%)	156 (34.1%)	2,012 (27.1%)	2,026 (27.3%)	1,024 (35.4%)	1,044 (36.1%)
South: n (%)	504 (47.8%)	495 (47.0%)	190 (41.5%)	186 (40.6%)	2,573 (34.7%)	2,567 (34.6%)	806 (27.9%)	778 (26.9%)
West: n (%)	198 (18.8%)	205 (19.4%)	76 (16.6%)	68 (14.8%)	1,211 (16.3%)	1,215 (16.4%)	525 (18.2%)	535 (18.5%)
Unknown/other: n (%)	0 (0.0%)	0 (0.0%)	0 (0.0%)	0 (0.0%)	93 (1.3%)	86 (1.2%)	51 (1.8%)	45 (1.6%)
Outpatient visits: mean (sd)	0.29 (0.50)	0.29 (0.51)	0.22 (0.72)	0.24 (0.52)	0.20 (0.48)	0.20 (0.48)	0.17 (0.43)	0.18 (0.44)
Inpatient hospitalization: mean (sd)	0.05 (0.29)	0.05 (0.31)	0.03 (0.23)	0.02 (0.16)	0.00 (0.00)	0.00 (0.00)	0.00 (0.00)	0.00 (0.00)
ED visit: mean (sd)	0.44 (1.04)	0.42 (0.95)	0.25 (0.70)	0.22 (0.76)	0.70 (1.64)	0.68 (1.46)	0.30 (0.94)	0.30 (0.90)

Number of distinct medication prescriptions: mean (sd)	9.36 (6.11)	9.09 (5.96)	8.71 (5.82)	8.42 (5.54)	9.94 (6.04)	9.90 (5.98)	9.10 (5.58)	9.05 (5.40)
Combined Comorbidity Score: mean (sd)	0.83 (1.97)	0.82 (1.97)	0.48 (1.41)	0.48 (1.56)	1.01 (2.05)	0.99 (2.01)	0.65 (1.61)	0.64 (1.58)
Osteoporosis: n (%)	438 (41.6%)	443 (42.0%)	133 (29.0%)	128 (27.9%)	2,506 (33.8%)	2,493 (33.6%)	633 (21.9%)	634 (21.9%)
Kyphosis: n (%)	38 (3.6%)	39 (3.7%)	5 (1.1%)	4 (0.9%)	288 (3.9%)	295 (4.0%)	47 (1.6%)	49 (1.7%)
Vertebral facture: n (%)	203 (19.3%)	197 (18.7%)	19 (4.1%)	11 (2.4%)	1,834 (24.7%)	1,849 (24.9%)	125 (4.3%)	123 (4.3%)
Humerus fracture: n (%)	19 (1.8%)	14 (1.3%)	2 (0.4%)	3 (0.7%)	116 (1.6%)	125 (1.7%)	16 (0.6%)	16 (0.6%)
Wrist fracture: n (%)	16 (1.5%)	15 (1.4%)	2 (0.4%)	3 (0.7%)	109 (1.5%)	103 (1.4%)	15 (0.5%)	17 (0.6%)
Hip fracture: n (%)	18 (1.7%)	21 (2.0%)	6 (1.3%)	7 (1.5%)	200 (2.7%)	170 (2.3%)	43 (1.5%)	40 (1.4%)
Pelvis fracture: n (%)	21 (2.0%)	21 (2.0%)	6 (1.3%)	6 (1.3%)	174 (2.3%)	138 (1.9%)	28 (1.0%)	26 (0.9%)
Any other fractures: n (%)	154 (14.6%)	149 (14.1%)	22 (4.8%)	21 (4.6%)	1,032 (13.9%)	988 (13.3%)	138 (4.8%)	150 (5.2%)
Alzheimer disease or other dementia: n (%)	37 (3.5%)	41 (3.9%)	21 (4.6%)	19 (4.1%)	522 (7.0%)	514 (6.9%)	153 (5.3%)	164 (5.7%)
Asthma or chronic obstructive pulmonary disease: n (%)	123 (11.7%)	110 (10.4%)	47 (10.3%)	48 (10.5%)	929 (12.5%)	926 (12.5%)	257 (8.9%)	266 (9.2%)
Cataracts: n (%)	75 (7.1%)	67 (6.4%)	51 (11.1%)	50 (10.9%)	632 (8.5%)	583 (7.9%)	288 (10.0%)	284 (9.8%)
Crohn disease or gastroenteritis: n (%)	45 (4.3%)	45 (4.3%)	16 (3.5%)	11 (2.4%)	207 (2.8%)	191 (2.6%)	73 (2.5%)	71 (2.5%)
Depression: n (%)	33 (3.1%)	27 (2.6%)	14 (3.1%)	14 (3.1%)	285 (3.8%)	275 (3.7%)	66 (2.3%)	52 (1.8%)
Diabetes mellitus: n (%)	143 (13.6%)	137 (13.0%)	49 (10.7%)	42 (9.2%)	1,140 (15.4%)	1,156 (15.6%)	362 (12.5%)	370 (12.8%)
History of falls, syncope, or gait abnormality: n (%)	181 (17.2%)	174 (16.5%)	62 (13.5%)	61 (13.3%)	1,359 (18.3%)	1,329 (17.9%)	303 (10.5%)	309 (10.7%)
Hyperthyroidism: n (%)	14 (1.3%)	15 (1.4%)	9 (2.0%)	7 (1.5%)	91 (1.2%)	90 (1.2%)	22 (0.8%)	26 (0.9%)
Hyperparathyroidism: n (%)	11 (1.0%)	13 (1.2%)	3 (0.7%)	3 (0.7%)	81 (1.1%)	72 (1.0%)	28 (1.0%)	26 (0.9%)
Ischemic stroke: n (%)	9 (0.9%)	5 (0.5%)	4 (0.9%)	0 (0.0%)	140 (1.9%)	143 (1.9%)	38 (1.3%)	35 (1.2%)
Liver disease: n (%)	46 (4.4%)	46 (4.4%)	11 (2.4%)	12 (2.6%)	256 (3.5%)	259 (3.5%)	60 (2.1%)	57 (2.0%)
Malignant neoplasm: n (%)	121 (11.5%)	125 (11.9%)	47 (10.3%)	45 (9.8%)	844 (11.4%)	862 (11.6%)	336 (11.6%)	339 (11.7%)
Overweight or obese: n (%)	43 (4.1%)	40 (3.8%)	7 (1.5%)	7 (1.5%)	240 (3.2%)	235 (3.2%)	29 (1.0%)	29 (1.0%)
Parkinson disease: n (%)	9 (0.9%)	9 (0.9%)	5 (1.1%)	4 (0.9%)	83 (1.1%)	90 (1.2%)	31 (1.1%)	36 (1.2%)
Renal disease: n (%)	27 (2.6%)	28 (2.7%)	10 (2.2%)	9 (2.0%)	284 (3.8%)	292 (3.9%)	71 (2.5%)	68 (2.4%)
Rheumatoid arthritis: n (%)	55 (5.2%)	55 (5.2%)	16 (3.5%)	19 (4.1%)	342 (4.6%)	331 (4.5%)	97 (3.4%)	92 (3.2%)
Oral glucocorticoids: n (%)	312 (29.6%)	306 (29.0%)	116 (25.3%)	113 (24.7%)	2,341 (31.5%)	2,346 (31.6%)	735 (25.4%)	755 (26.1%)

Anticonvulsants/antiepileptic drugs: n (%)	171 (16.2%)	166 (15.7%)	60 (13.1%)	61 (13.3%)	1,200 (16.2%)	1,229 (16.6%)	366 (12.7%)	360 (12.4%)
Benzodiazepines: n (%)	260 (24.7%)	260 (24.7%)	108 (23.6%)	96 (21.0%)	1,693 (22.8%)	1,690 (22.8%)	602 (20.8%)	599 (20.7%)
SSRIs: n (%)	224 (21.3%)	210 (19.9%)	102 (22.3%)	98 (21.4%)	1,700 (22.9%)	1,673 (22.5%)	610 (21.1%)	569 (19.7%)
Beta-blockers: n (%)	209 (19.8%)	212 (20.1%)	96 (21.0%)	88 (19.2%)	1,902 (25.6%)	1,852 (25.0%)	799 (27.6%)	789 (27.3%)
Proton pump inhibitors: n (%)	256 (24.3%)	237 (22.5%)	120 (26.2%)	110 (24.0%)	2,240 (30.2%)	2,201 (29.7%)	1,021 (35.3%)	998 (34.5%)
Opioids: n (%)	558 (52.9%)	544 (51.6%)	145 (31.7%)	136 (29.7%)	4,075 (54.9%)	4,079 (55.0%)	1,025 (35.4%)	1,019 (35.2%)
Other anti-osteoporosis medications (without calcitonin): n (%)	262 (24.9%)	261 (24.8%)	76 (16.6%)	71 (15.5%)	1,594 (21.5%)	1,628 (21.9%)	445 (15.4%)	435 (15.0%)

Table N- Characteristics of patients included in the evaluation of comparative outcomes for *escitalopram* between authorized generic (AG) and generic initiators and switchers after 1:1 propensity score matching in each database

	Optum				Truven			
	AG initiators	Generic initiators	AG switchers	Generic switchers	AG initiators	Generic initiators	AG switchers	Generic switchers
**Cohort selection steps**								
Total number of patients filling prescriptions for the version of interest after 6-month continuous enrollment	139,206		180,712		1,353,918		1,353,211	
Patients meeting new drug use restriction	79,631		95,726		741,945		607,818	
Patients meeting prior brand-name use requirement (to define switchers, applicable for switchers comparison only)	NA		58,459		NA		322,248	
Total eligible by exposure group	25,580	54,051	44,335	14,124	127,804	614,141	181,555	140,693
**1:1 PS matched**	24,445	24,445	12,693	12,693	127,803	127,803	134,298	134,298
**Baseline characteristics**								
Age: mean (sd)	39 (14.97)	39 (14.86)	46 (14.65)	46 (14.84)	42.83 (17.75)	42.93 (17.56)	49.63 (15.90)	49.76 (15.83)
Gender								
MALE: n (%)	8,340 (34.1%)	8,313 (34.0%)	3,785 (29.8%)	3,830 (30.2%)	41,048 (32.1%)	40,628 (31.8%)	39,991 (29.8%)	40,070 (29.8%)
FEMALE: n (%)	16,101 (65.9%)	16,125 (66.0%)	8,906 (70.2%)	8,861 (69.8%)	86,755 (67.9%)	87,175 (68.2%)	94,307 (70.2%)	94,228 (70.2%)
UNKNOWN: n (%)	4 (0.0%)	7 (0.0%)	2 (0.0%)	2 (0.0%)	0 (0.0%)	0 (0.0%)	0 (0.0%)	0 (0.0%)
Region								
Northeast: n (%)	2,020 (8.3%)	2,028 (8.3%)	1,477 (11.6%)	1,529 (12.0%)	21,201 (16.6%)	22,064 (17.3%)	28,433 (21.2%)	28,816 (21.5%)
Midwest: n (%)	5,958 (24.4%)	5,797 (23.7%)	2,487 (19.6%)	2,535 (20.0%)	25,972 (20.3%)	24,907 (19.5%)	30,566 (22.8%)	30,576 (22.8%)
South: n (%)	12,430 (50.8%)	12,489 (51.1%)	6,570 (51.8%)	6,455 (50.9%)	59,243 (46.4%)	59,023 (46.2%)	52,830 (39.3%)	52,348 (39.0%)
West: n (%)	4,030 (16.5%)	4,124 (16.9%)	2,159 (17.0%)	2,174 (17.1%)	18,806 (14.7%)	19,100 (14.9%)	19,619 (14.6%)	19,700 (14.7%)
Unknown/other: n (%)	7 (0.0%)	7 (0.0%)	0 (0.0%)	0 (0.0%)	2,581 (2.0%)	2,709 (2.1%)	2,850 (2.1%)	2,858 (2.1%)

Epilepsy: n (%)	331 (1.4%)	321 (1.3%)	139 (1.1%)	152 (1.2%)	1,839 (1.4%)	1,740 (1.4%)	1,475 (1.1%)	1,511 (1.1%)
Depression: n (%)	2,455 (10.0%)	2,451 (10.0%)	1,136 (8.9%)	1,185 (9.3%)	12,928 (10.1%)	12,301 (9.6%)	9,975 (7.4%)	10,222 (7.6%)
Anxiety: n (%)	2,541 (10.4%)	2,544 (10.4%)	1,258 (9.9%)	1,297 (10.2%)	11,363 (8.9%)	10,881 (8.5%)	9,352 (7.0%)	9,565 (7.1%)
Alcohol abuse or dependence: n (%)	546 (2.2%)	539 (2.2%)	180 (1.4%)	182 (1.4%)	2,267 (1.8%)	2,133 (1.7%)	1,190 (0.9%)	1,229 (0.9%)
Drug abuse or dependence: n (%)	578 (2.4%)	559 (2.3%)	186 (1.5%)	187 (1.5%)	2,834 (2.2%)	2,660 (2.1%)	1,197 (0.9%)	1,208 (0.9%)
Sleep disorder: n (%)	2,104 (8.6%)	2,116 (8.7%)	895 (7.1%)	939 (7.4%)	8,822 (6.9%)	8,288 (6.5%)	6,907 (5.1%)	7,033 (5.2%)
Psychotic disorder: n (%)	377 (1.5%)	376 (1.5%)	163 (1.3%)	168 (1.3%)	2,211 (1.7%)	2,128 (1.7%)	1,373 (1.0%)	1,362 (1.0%)
Personality disorder: n (%)	118 (0.5%)	118 (0.5%)	61 (0.5%)	61 (0.5%)	570 (0.4%)	533 (0.4%)	394 (0.3%)	399 (0.3%)
Adjustment reaction/post-traumatic stress disorder: n (%)	1,502 (6.1%)	1,513 (6.2%)	578 (4.6%)	587 (4.6%)	7,158 (5.6%)	6,782 (5.3%)	4,564 (3.4%)	4,668 (3.5%)
ADHD: n (%)	1,398 (5.7%)	1,369 (5.6%)	662 (5.2%)	682 (5.4%)	5,831 (4.6%)	5,418 (4.2%)	3,939 (2.9%)	4,012 (3.0%)
Delirium: n (%)	302 (1.2%)	303 (1.2%)	121 (1.0%)	139 (1.1%)	1,964 (1.5%)	1,933 (1.5%)	1,089 (0.8%)	1,058 (0.8%)
Bipolar disorder: n (%)	74 (0.3%)	78 (0.3%)	41 (0.3%)	44 (0.3%)	287 (0.2%)	274 (0.2%)	265 (0.2%)	275 (0.2%)
Other psychiatric disorder: n (%)	1,206 (4.9%)	1,209 (4.9%)	386 (3.0%)	405 (3.2%)	5,142 (4.0%)	4,899 (3.8%)	2,805 (2.1%)	2,875 (2.1%)
Psychiatric hospitalization: mean (sd)	0.03 (0.23)	0.03 (0.22)	0.02 (0.18)	0.02 (0.20)	0.03 (0.21)	0.03 (0.21)	0.01 (0.11)	0.01 (0.11)
Psychiatric office visit: mean (sd)	2.43 (8.31)	2.39 (9.85)	2.12 (6.55)	2.18 (7.20)	2.52 (10.58)	2.31 (10.01)	1.88 (6.85)	1.90 (7.48)
Anticonvulsants/antiepileptic drugs: n (%)	1,940 (7.9%)	1,895 (7.8%)	1,214 (9.6%)	1,254 (9.9%)	10,879 (8.5%)	10,537 (8.2%)	13,725 (10.2%)	14,053 (10.5%)
SSRIs (without escitalopram): n (%)	5,377 (22.0%)	5,373 (22.0%)	851 (6.7%)	859 (6.8%)	26,093 (20.4%)	25,149 (19.7%)	5,234 (3.9%)	5,294 (3.9%)
SNRIs: n (%)	1,290 (5.3%)	1,280 (5.2%)	277 (2.2%)	296 (2.3%)	6,710 (5.3%)	6,408 (5.0%)	2,704 (2.0%)	2,790 (2.1%)
Tricyclic antidepressants: n (%)	561 (2.3%)	579 (2.4%)	330 (2.6%)	328 (2.6%)	3,314 (2.6%)	3,162 (2.5%)	3,202 (2.4%)	3,319 (2.5%)
Antipsychotics: n (%)	1,052 (4.3%)	1,019 (4.2%)	770 (6.1%)	808 (6.4%)	5,698 (4.5%)	5,513 (4.3%)	7,962 (5.9%)	8,174 (6.1%)
Benzodiazepines: n (%)	5,970 (24.4%)	5,900 (24.1%)	3,331 (26.2%)	3,441 (27.1%)	30,807 (24.1%)	29,509 (23.1%)	33,180 (24.7%)	33,331 (24.8%)
Non-BZD sedative hypnotics: n (%)	2,889 (11.8%)	2,891 (11.8%)	1,790 (14.1%)	1,841 (14.5%)	15,072 (11.8%)	14,466 (11.3%)	17,822 (13.3%)	18,144 (13.5%)
Outpatient visits: mean (sd)	0.26 (0.51)	0.26 (0.57)	0.26 (0.49)	0.26 (0.50)	0.22 (0.48)	0.22 (0.46)	0.22 (0.46)	0.22 (0.46)
Inpatient hospitalization: mean (sd)	0.01 (0.11)	0.01 (0.11)	0.01 (0.14)	0.01 (0.15)	0.00 (0.01)	0.00 (0.01)	0.00 (0.01)	0.00 (0.02)
ED visit: mean (sd)	0.27 (0.84)	0.27 (0.83)	0.17 (0.81)	0.17 (0.61)	0.42 (1.26)	0.41 (1.30)	0.23 (0.89)	0.24 (0.89)
Number of distinct medication prescriptions: mean (sd)	4.52 (4.28)	4.49 (4.17)	6.13 (4.43)	6.28 (4.61)	4.98 (4.61)	4.88 (4.50)	6.65 (4.57)	6.71 (4.68)
Combined Comorbidity Score: mean (sd)	0.34 (1.12)	0.33 (1.10)	0.30 (1.04)	0.34 (1.12)	0.40 (1.29)	0.39 (1.28)	0.27 (1.08)	0.28 (1.09)

Table O- Characteristics of patients included in the evaluation of comparative outcomes for *glipizide* between authorized generic (AG) and generic initiators and switchers after 1:1 propensity score matching in each database

	Optum				Truven			
	AG initiators	Generic initiators	AG switchers	Generic switchers	AG initiators	Generic initiators	AG switchers	Generic switchers
**Cohort selection steps**								
Total number of patients filling prescriptions for the version of interest after 6-month continuous enrollment	**46,110**		**148,589**		**543,534**		**538,518**	
Patients meeting new drug use restriction	**39,996**		**31,623**		**261,240**		**131,887**	
Patients meeting prior brand-name use requirement (to define switchers, applicable for switchers comparison only)	**NA**		**13,131**		**NA**		**60,974**	
Total eligible by exposure group	**2,194**	**37,802**	**735**	**12,396**	**66,713**	**194,527**	**2,849**	**58,125**
**1:1 PS matched**	**2,193**	**2,193**	**723**	**723**	**66,713**	**66,713**	**2,840**	**2,840**
**Baseline characteristics**								
Age: mean (sd)	58.48 (11.33)	58.41 (12.42)	59.68 (10.17)	59.98 (10.50)	58.65 (12.63)	58.63 (13.15)	65.11 (11.46)	65.31 (12.15)
Gender								
MALE: n (%)	1,242 (56.6%)	1,237 (56.4%)	429 (59.3%)	438 (60.6%)	37,126 (55.7%)	37,120 (55.6%)	1440 (50.7)	1413 (49.75)
FEMALE: n (%)	950 (43.3%)	956 (43.6%)	294 (40.7%)	285 (39.4%)	29,587 (44.3%)	29,593 (44.4%)	1400 (49.3)	1427 (50.25)
UNKNOWN: n (%)	1 (0.0%)	0 (0.0%)	0 (0.0%)	0 (0.0%)	0 (0.0%)	0 (0.0%)	0 (0.0%)	0 (0.0%)
Region								
Northeast: n (%)	199 (9.1%)	189 (8.6%)	117 (16.2%)	106 (14.7%)	12,711 (19.1%)	12,653 (19.0%)	510 (17.96)	416 (14.65)
Midwest: n (%)	388 (17.7%)	379 (17.3%)	160 (22.1%)	164 (22.7%)	18,947 (28.4%)	19,190 (28.8%)	835 (29.4)	880 (30.99)
South: n (%)	1,179 (53.8%)	1,166 (53.2%)	368 (50.9%)	372 (51.5%)	25,348 (38.0%)	25,258 (37.9%)	1134 (39.93)	1207 (42.5)
West: n (%)	424 (19.3%)	456 (20.8%)	78 (10.8%)	81 (11.2%)	8,333 (12.5%)	8,229 (12.3%)	333 (11.73)	306 (10.77)
Unknown/other: n (%)	3 (0.1%)	3 (0.1%)	0 (0.0%)	0 (0.0%)	1,374 (2.1%)	1,383 (2.1%)	28 (0.99)	31 (1.09)
Hyperlipidemia: n (%)	873 (39.8%)	864 (39.4%)	438 (60.6%)	427 (59.1%)	26,762 (40.1%)	26,651 (39.9%)	690 (24.3)	649 (22.85)
Diabetes mellitus: n (%)	1,414 (64.5%)	1,424 (64.9%)	642 (88.8%)	637 (88.1%)	52,524 (78.7%)	52,218 (78.3%)	2118 (74.58)	2164 (76.2)
Hypertension: n (%)	996 (45.4%)	983 (44.8%)	446 (61.7%)	450 (62.2%)	31,789 (47.7%)	31,428 (47.1%)	986 (34.72)	937 (32.99)

Myocardial Infarction: n (%)	23 (1.0%)	28 (1.3%)	9 (1.2%)	8 (1.1%)	621 (0.9%)	638 (1.0%)	21 (0.74)	18 (0.63)
Angina: n (%)	62 (2.8%)	57 (2.6%)	26 (3.6%)	21 (2.9%)	1,562 (2.3%)	1,585 (2.4%)	71 (2.5)	56 (1.97)
Heart failure: n (%)	85 (3.9%)	78 (3.6%)	36 (5.0%)	36 (5.0%)	2,877 (4.3%)	2,829 (4.2%)	115 (4.05)	126 (4.44)
Other forms of chronic heart diseases: n (%)	228 (10.4%)	217 (9.9%)	101 (14.0%)	101 (14.0%)	7,232 (10.8%)	7,236 (10.8%)	374 (13.17)	402 (14.15)
Cerebrovascular accident: n (%)	28 (1.3%)	26 (1.2%)	9 (1.2%)	6 (0.8%)	1,171 (1.8%)	1,192 (1.8%)	42 (1.48)	47 (1.65)
Venous thromboembolism: n (%)	26 (1.2%)	25 (1.1%)	11 (1.5%)	12 (1.7%)	863 (1.3%)	840 (1.3%)	35 (1.23)	38 (1.34)
Atrial fibrillation: n (%)	60 (2.7%)	60 (2.7%)	24 (3.3%)	25 (3.5%)	2,538 (3.8%)	2,511 (3.8%)	124 (4.37)	141 (4.96)
Overweight or obese: n (%)	101 (4.6%)	101 (4.6%)	46 (6.4%)	49 (6.8%)	4,194 (6.3%)	4,040 (6.1%)	40 (1.41)	47 (1.65)
Tobacco use: n (%)	36 (1.6%)	40 (1.8%)	14 (1.9%)	14 (1.9%)	2,126 (3.2%)	2,146 (3.2%)	24 (0.85)	29 (1.02)
Alcohol abuse or dependence: n (%)	6 (0.3%)	7 (0.3%)	3 (0.4%)	4 (0.6%)	325 (0.5%)	325 (0.5%)	3 (0.11)	5 (0.18)
Renal disease: n (%)	50 (2.3%)	39 (1.8%)	30 (4.1%)	36 (5.0%)	2,331 (3.5%)	2,300 (3.4%)	62 (2.18)	47 (1.65)
Liver disease: n (%)	48 (2.2%)	46 (2.1%)	21 (2.9%)	18 (2.5%)	1,874 (2.8%)	1,858 (2.8%)	42 (1.48)	42 (1.48)
Aspirin: n (%)	0 (0.0%)	0 (0.0%)	0 (0.0%)	0 (0.0%)	156 (0.2%)	167 (0.3%)	1 (0.04)	2 (0.07)
Antiplatelets: n (%)	88 (4.0%)	79 (3.6%)	47 (6.5%)	41 (5.7%)	3,827 (5.7%)	3,837 (5.8%)	251 (8.84)	288 (10.14)
Statins: n (%)	620 (28.3%)	600 (27.4%)	398 (55.0%)	408 (56.4%)	30,738 (46.1%)	30,538 (45.8%)	1570 (55.28)	1594 (56.13)
Other lipid lowering agents: n (%)	204 (9.3%)	202 (9.2%)	120 (16.6%)	121 (16.7%)	8,257 (12.4%)	8,150 (12.2%)	454 (15.99)	598 (21.06)
Insulin preparations: n (%)	0 (0.0%)	0 (0.0%)	0 (0.0%)	0 (0.0%)	0 (0.0%)	0 (0.0%)	0 (0.0%)	0 (0.0%)
Oral hypoglycemic agents (without glipizide): n (%)	913 (41.6%)	913 (41.6%)	495 (68.5%)	480 (66.4%)	41,818 (62.7%)	41,358 (62.0%)	1874 (65.99)	1871 (65.88)
ACE inhibitors: n (%)	616 (28.1%)	594 (27.1%)	324 (44.8%)	311 (43.0%)	24,457 (36.7%)	24,244 (36.3%)	1222 (43.03)	1202 (42.32)
ARBs: n (%)	271 (12.4%)	264 (12.0%)	164 (22.7%)	185 (25.6%)	12,621 (18.9%)	12,490 (18.7%)	805 (28.35)	892 (31.41)
Calcium channel blockers: n (%)	332 (15.1%)	321 (14.6%)	148 (20.5%)	154 (21.3%)	13,048 (19.6%)	12,989 (19.5%)	771 (27.15)	738 (25.99)
Diuretics: n (%)	540 (24.6%)	545 (24.9%)	269 (37.2%)	282 (39.0%)	22,742 (34.1%)	22,500 (33.7%)	1276 (44.93)	1355 (47.71)
Beta-blockers: n (%)	371 (16.9%)	343 (15.6%)	168 (23.2%)	162 (22.4%)	15,826 (23.7%)	15,726 (23.6%)	855 (30.11)	841 (29.61)
Anticoagulants: n (%)	63 (2.9%)	66 (3.0%)	28 (3.9%)	27 (3.7%)	2,873 (4.3%)	2,865 (4.3%)	192 (6.76)	187 (6.58)
NSAIDs: n (%)	195 (8.9%)	205 (9.3%)	79 (10.9%)	61 (8.4%)	8,828 (13.2%)	8,722 (13.1%)	397 (13.98)	417 (14.68)
Coxibs: n (%)	65 (3.0%)	65 (3.0%)	38 (5.3%)	30 (4.1%)	1,370 (2.1%)	1,367 (2.0%)	218 (7.68)	174 (6.13)
Outpatient visits: mean (sd)	0.08 (0.28)	0.08 (0.28)	0.11 (0.32)	0.12 (0.35)	0.11 (0.40)	0.11 (0.35)	0.07 (0.35)	0.06 (0.27)
Inpatient hospitalization: mean (sd)	0.00 (0.06)	0.01 (0.09)	0.00 (0.05)	0.00 (0.05)	0.00 (0.01)	0.00 (0.00)	0.00 (0.01)	0.00 (0.00)
ED visit: mean (sd)	0.20 (0.73)	0.19 (0.63)	0.17 (0.60)	0.17 (0.56)	0.27 (0.93)	0.27 (0.84)	0.18 (0.61)	0.19 (0.65)

Number of distinct medication prescriptions: mean (sd)	4.33 (4.30)	4.35 (4.43)	8.19 (4.17)	8.06 (4.32)	6.61 (4.82)	6.56 (4.82)	8.93 (4.69)	9.28 (4.93)
Combined Comorbidity Score	0.21 (1.34)	0.20 (1.40)	0.20 (1.38)	0.16 (1.34)	0.29 (1.43)	0.29 (1.43)	0.33 (1.2)	0.35 (1.26)

Table P- Characteristics of patients included in the evaluation of comparative outcomes for *quinapril* between authorized generic (AG) and generic initiators and switchers after 1:1 propensity score matching in each database

	Optum				Truven			
	AG initiators	Generic initiators	AG switchers	Generic switchers	AG initiators	Generic initiators	AG switchers	Generic switchers
**Cohort selection steps**								
Total number of patients filling prescriptions for the version of interest after 6-month continuous enrollment	36,723		**92,337**		**326,298**		326,014	
Patients meeting new drug use restriction	20,458		**40,444**		**82,411**		108,556	
Patients meeting prior brand-name use requirement (to define switchers, applicable for switchers comparison only)	NA		**33,514**		NA		82,982	
Total eligible by exposure group	9,622	10,836	**15,371**	**18,143**	**41,347**	**41,064**	55,591	27,391
**1:1 PS matched**	8,335	8,335	**14,369**	**14,369**	**32,074**	**32,074**	25,766	25,766
**Baseline characteristics**								
Age: mean (sd)	53 (11.72)	53 (11.57)	57 (10.17)	57 (10.28)	57.08 (13.01)	57.13 (12.87)	59.92 (11.83)	59.87 (12.38)
Gender								
MALE: n (%)	4,784 (57.4%)	4,787 (57.4%)	8,659 (60.3%)	8,625 (60.0%)	17,551 (54.7%)	17,642 (55.0%)	13,992 (54.3%)	14,004 (54.4%)
FEMALE: n (%)	3,550 (42.6%)	3,548 (42.6%)	5,709 (39.7%)	5,743 (40.0%)	14,523 (45.3%)	14,432 (45.0%)	11,774 (45.7%)	11,762 (45.6%)
UNKNOWN: n (%)	1 (0.0%)	0 (0.0%)	1 (0.0%)	1 (0.0%)	0 (0.0%)	0 (0.0%)	0 (0.0%)	0 (0.0%)
Region								
Northeast: n (%)	1,024 (12.3%)	1,108 (13.3%)	2,004 (13.9%)	1,992 (13.9%)	7,014 (21.9%)	7,237 (22.6%)	2,839 (11.0%)	2,916 (11.3%)
Midwest: n (%)	2,101 (25.2%)	2,107 (25.3%)	4,661 (32.4%)	4,685 (32.6%)	8,739 (27.2%)	8,659 (27.0%)	7,087 (27.5%)	7,042 (27.3%)
South: n (%)	4,456 (53.5%)	4,380 (52.5%)	6,296 (43.8%)	6,300 (43.8%)	12,407 (38.7%)	12,301 (38.4%)	12,097 (46.9%)	12,002 (46.6%)
West: n (%)	743 (8.9%)	729 (8.7%)	1,398 (9.7%)	1,382 (9.6%)	3,314 (10.3%)	3,298 (10.3%)	3,583 (13.9%)	3,637 (14.1%)
Unknown/other: n (%)	11 (0.1%)	11 (0.1%)	10 (0.1%)	10 (0.1%)	600 (1.9%)	579 (1.8%)	160 (0.6%)	169 (0.7%)
Hyperlipidemia: n (%)	3,475 (41.7%)	3,463 (41.5%)	7,020 (48.9%)	7,025 (48.9%)	10,197 (31.8%)	10,283 (32.1%)	5,544 (21.5%)	5,701 (22.1%)

Diabetes mellitus: n (%)	2,312 (27.7%)	2,263 (27.2%)	4,229 (29.4%)	4,219 (29.4%)	8,740 (27.2%)	8,769 (27.3%)	6,640 (25.8%)	6,719 (26.1%)
Hypertension: n (%)	5,085 (61.0%)	5,091 (61.1%)	9,467 (65.9%)	9,452 (65.8%)	16,398 (51.1%)	16,483 (51.4%)	11,250 (43.7%)	11,303 (43.9%)
Myocardial Infarction: n (%)	187 (2.2%)	179 (2.1%)	93 (0.6%)	102 (0.7%)	648 (2.0%)	676 (2.1%)	158 (0.6%)	145 (0.6%)
Angina: n (%)	330 (4.0%)	329 (3.9%)	392 (2.7%)	386 (2.7%)	1,052 (3.3%)	1,072 (3.3%)	636 (2.5%)	613 (2.4%)
Heart failure: n (%)	355 (4.3%)	349 (4.2%)	509 (3.5%)	505 (3.5%)	1,435 (4.5%)	1,450 (4.5%)	1,008 (3.9%)	970 (3.8%)
Other forms of chronic heart diseases: n (%)	930 (11.2%)	932 (11.2%)	1,879 (13.1%)	1,838 (12.8%)	3,505 (10.9%)	3,529 (11.0%)	2,752 (10.7%)	2,762 (10.7%)
Cerebrovascular accident: n (%)	174 (2.1%)	175 (2.1%)	142 (1.0%)	143 (1.0%)	720 (2.2%)	704 (2.2%)	314 (1.2%)	325 (1.3%)
Venous thromboembolism: n (%)	98 (1.2%)	99 (1.2%)	128 (0.9%)	123 (0.9%)	364 (1.1%)	378 (1.2%)	190 (0.7%)	200 (0.8%)
Atrial fibrillation: n (%)	258 (3.1%)	261 (3.1%)	448 (3.1%)	436 (3.0%)	1,275 (4.0%)	1,294 (4.0%)	841 (3.3%)	829 (3.2%)
Overweight or obese: n (%)	469 (5.6%)	469 (5.6%)	537 (3.7%)	514 (3.6%)	1,170 (3.6%)	1,207 (3.8%)	240 (0.9%)	225 (0.9%)
Tobacco use: n (%)	356 (4.3%)	349 (4.2%)	304 (2.1%)	294 (2.0%)	781 (2.4%)	800 (2.5%)	144 (0.6%)	154 (0.6%)
Alcohol abuse or dependence: n (%)	79 (0.9%)	77 (0.9%)	50 (0.3%)	50 (0.3%)	171 (0.5%)	178 (0.6%)	54 (0.2%)	57 (0.2%)
Renal disease: n (%)	179 (2.1%)	178 (2.1%)	238 (1.7%)	233 (1.6%)	653 (2.0%)	671 (2.1%)	338 (1.3%)	340 (1.3%)
Liver disease: n (%)	188 (2.3%)	190 (2.3%)	240 (1.7%)	234 (1.6%)	557 (1.7%)	548 (1.7%)	256 (1.0%)	266 (1.0%)
Aspirin: n (%)	3 (0.0%)	3 (0.0%)	13 (0.1%)	10 (0.1%)	143 (0.4%)	151 (0.5%)	181 (0.7%)	170 (0.7%)
Antiplatelets: n (%)	260 (3.1%)	273 (3.3%)	743 (5.2%)	753 (5.2%)	1,387 (4.3%)	1,349 (4.2%)	1,636 (6.3%)	1,603 (6.2%)
Statins: n (%)	1,982 (23.8%)	1,955 (23.5%)	6,567 (45.7%)	6,560 (45.7%)	8,564 (26.7%)	8,527 (26.6%)	11,425 (44.3%)	11,604 (45.0%)
Other lipid lowering agents: n (%)	604 (7.2%)	602 (7.2%)	1,669 (11.6%)	1,671 (11.6%)	2,303 (7.2%)	2,314 (7.2%)	2,396 (9.3%)	2,418 (9.4%)
Insulin preparations: n (%)	418 (5.0%)	415 (5.0%)	806 (5.6%)	802 (5.6%)	1,149 (3.6%)	1,146 (3.6%)	945 (3.7%)	984 (3.8%)
Oral hypoglycemic agents: n (%)	1,375 (16.5%)	1,352 (16.2%)	3,574 (24.9%)	3,518 (24.5%)	5,528 (17.2%)	5,483 (17.1%)	6,876 (26.7%)	6,872 (26.7%)
ACE inhibitors: n (%)	964 (11.6%)	994 (11.9%)	121 (0.8%)	125 (0.9%)	5,049 (15.7%)	5,009 (15.6%)	25,186 (97.7%)	25,240 (98.0%)
ARBs: n (%)	575 (6.9%)	564 (6.8%)	544 (3.8%)	551 (3.8%)	2,347 (7.3%)	2,355 (7.3%)	1,168 (4.5%)	1,172 (4.5%)
Calcium channel blockers (without quinapril): n (%)	1,179 (14.1%)	1,148 (13.8%)	3,225 (22.4%)	3,216 (22.4%)	1,971 (6.1%)	2,002 (6.2%)	2,881 (11.2%)	2,965 (11.5%)
Diuretics: n (%)	1,930 (23.2%)	1,917 (23.0%)	5,324 (37.1%)	5,326 (37.1%)	8,202 (25.6%)	8,138 (25.4%)	10,801 (41.9%)	10,818 (42.0%)
Beta-blockers: n (%)	1,395 (16.7%)	1,400 (16.8%)	3,829 (26.6%)	3,825 (26.6%)	5,987 (18.7%)	5,995 (18.7%)	7,343 (28.5%)	7,346 (28.5%)
Anticoagulants: n (%)	215 (2.6%)	217 (2.6%)	582 (4.1%)	554 (3.9%)	1,070 (3.3%)	1,071 (3.3%)	1,424 (5.5%)	1,412 (5.5%)

NSAIDs: n (%)	979 (11.7%)	967 (11.6%)	1,782 (12.4%)	1,758 (12.2%)	3,590 (11.2%)	3,602 (11.2%)	3,380 (13.1%)	3,417 (13.3%)
Coxibs: n (%)	150 (1.8%)	154 (1.8%)	1,082 (7.5%)	1,091 (7.6%)	650 (2.0%)	636 (2.0%)	2,360 (9.2%)	2,297 (8.9%)
Outpatient visits: mean (sd)	0.15 (0.39)	0.15 (0.40)	0.13 (0.41)	0.13 (0.41)	0.12 (0.38)	0.12 (0.37)	0.08 (0.29)	0.08 (0.29)
Inpatient hospitalization: mean (sd)	0.01 (0.10)	0.01 (0.10)	0.00 (0.07)	0.00 (0.07)	0.00 (0.00)	0.00 (0.01)	0.00 (0.00)	0.00 (0.00)
ED visit: mean (sd)	0.23 (0.76)	0.22 (0.65)	0.13 (0.52)	0.12 (0.51)	0.26 (0.90)	0.27 (0.86)	0.16 (0.62)	0.16 (0.59)
Number of distinct medication prescriptions: mean (sd)	4.17 (4.18)	4.16 (4.08)	6.61 (4.13)	6.58 (4.16)	4.50 (4.87)	4.49 (4.58)	7.28 (4.44)	7.28 (4.58)
Combined Comorbidity Score: mean (sd)	0.06 (1.37)	0.05 (1.32)	-0.11 (1.19)	-0.11 (1.18)	0.12 (1.33)	0.13 (1.36)	0.04 (1.10)	0.03 (1.10)

Table Q- Characteristics of patients included in the evaluation of comparative outcomes for *sertraline* between authorized generic (AG) and generic initiators and switchers after 1:1 propensity score matching in each database

	Optum				Truven			
	AG initiators	Generic initiators	AG switchers	Generic switchers	AG initiators	Generic initiators	AG switchers	Generic switchers
**Cohort selection steps**								
Total number of patients filling prescriptions for the version of interest after 6-month continuous enrollment	544,275		758,096		**2,909,191**		**3,392,102**	
Patients meeting new drug use restriction	420,347		272,990		**1,782,673**		**903,262**	
Patients meeting prior brand-name use requirement (to define switchers, applicable for switchers comparison only)	NA		111,191		NA		**253,183**	
Total eligible by exposure group	193,445	226,902	52,385	58,806	**688,454**	**1,094,219**	**112,479**	**140,704**
**1:1 PS matched**	177,959	177,959	48,019	48,019	**639,272**	**639,272**	**107,150**	**107,150**
**Baseline characteristics**								
Age: mean (sd)	38 (14.87)	38 (15.20)	44 (13.80)	44 (14.48)	41.01 (17.29)	41.09 (17.70)	49.96 (16.61)	49.95 (16.96)
Gender								
MALE: n (%)	58,874 (33.1%)	58,888 (33.1%)	13,879 (28.9%)	13,789 (28.7%)	206,972 (32.4%)	206,752 (32.3%)	30,635 (28.6%)	30,560 (28.5%)
FEMALE: n (%)	119,070 (66.9%)	119,055 (66.9%)	34,135 (71.1%)	34,228 (71.3%)	432,300 (67.6%)	432,520 (67.7%)	76,515 (71.4%)	76,590 (71.5%)
UNKNOWN: n (%)	15 (0.0%)	16 (0.0%)	5 (0.0%)	2 (0.0%)	0 (0.0%)	0 (0.0%)	0 (0.0%)	0 (0.0%)
Region								
Northeast: n (%)	14,523 (8.2%)	14,085 (7.9%)	3,284 (6.8%)	3,364 (7.0%)	83,992 (13.1%)	76,598 (12.0%)	9,696 (9.0%)	9,824 (9.2%)
Midwest: n (%)	52,992 (29.8%)	52,595 (29.6%)	16,219 (33.8%)	16,081 (33.5%)	180,006 (28.2%)	178,685 (28.0%)	31,282 (29.2%)	30,787 (28.7%)
South: n (%)	84,632 (47.6%)	85,641 (48.1%)	21,815 (45.4%)	21,768 (45.3%)	259,334 (40.6%)	268,766 (42.0%)	50,813 (47.4%)	51,209 (47.8%)
West: n (%)	25,759 (14.5%)	25,594 (14.4%)	6,681 (13.9%)	6,787 (14.1%)	103,733 (16.2%)	103,311 (16.2%)	14,856 (13.9%)	14,833 (13.8%)
Unknown/other: n (%)	53 (0.0%)	44 (0.0%)	20 (0.0%)	19 (0.0%)	12,207 (1.9%)	11,912 (1.9%)	503 (0.5%)	497 (0.5%)

Epilepsy: n (%)	2,247 (1.3%)	2,232 (1.3%)	546 (1.1%)	548 (1.1%)	8,101 (1.3%)	7,895 (1.2%)	1,075 (1.0%)	1,064 (1.0%)
Depression: n (%)	16,977 (9.5%)	16,764 (9.4%)	3,762 (7.8%)	3,764 (7.8%)	59,013 (9.2%)	57,461 (9.0%)	5,552 (5.2%)	5,479 (5.1%)
Anxiety: n (%)	15,636 (8.8%)	15,353 (8.6%)	3,280 (6.8%)	3,293 (6.9%)	47,928 (7.5%)	46,244 (7.2%)	4,166 (3.9%)	4,056 (3.8%)
Alcohol abuse or dependence: n (%)	3,604 (2.0%)	3,561 (2.0%)	478 (1.0%)	473 (1.0%)	10,277 (1.6%)	9,926 (1.6%)	570 (0.5%)	570 (0.5%)
Drug abuse or dependence: n (%)	3,819 (2.1%)	3,777 (2.1%)	385 (0.8%)	394 (0.8%)	11,760 (1.8%)	11,422 (1.8%)	417 (0.4%)	432 (0.4%)
Sleep disorder: n (%)	14,847 (8.3%)	14,710 (8.3%)	3,637 (7.6%)	3,594 (7.5%)	38,004 (5.9%)	37,303 (5.8%)	5,367 (5.0%)	5,319 (5.0%)
Psychotic disorder: n (%)	2,621 (1.5%)	2,561 (1.4%)	482 (1.0%)	464 (1.0%)	10,059 (1.6%)	9,729 (1.5%)	902 (0.8%)	874 (0.8%)
Personality disorder: n (%)	1,064 (0.6%)	1,047 (0.6%)	229 (0.5%)	236 (0.5%)	2,804 (0.4%)	2,630 (0.4%)	183 (0.2%)	192 (0.2%)
Adjustment reaction/post-traumatic stress disorder: n (%)	10,180 (5.7%)	10,122 (5.7%)	1,667 (3.5%)	1,691 (3.5%)	32,932 (5.2%)	31,743 (5.0%)	2,595 (2.4%)	2,580 (2.4%)
ADHD: n (%)	8,698 (4.9%)	8,530 (4.8%)	1,517 (3.2%)	1,497 (3.1%)	25,554 (4.0%)	25,030 (3.9%)	1,818 (1.7%)	1,776 (1.7%)
Delirium: n (%)	1,927 (1.1%)	1,919 (1.1%)	302 (0.6%)	310 (0.6%)	7,371 (1.2%)	7,317 (1.1%)	601 (0.6%)	586 (0.5%)
Bipolar disorder: n (%)	444 (0.2%)	441 (0.2%)	101 (0.2%)	101 (0.2%)	1,256 (0.2%)	1,186 (0.2%)	121 (0.1%)	119 (0.1%)
Other psychiatric disorder: n (%)	9,319 (5.2%)	9,259 (5.2%)	1,613 (3.4%)	1,589 (3.3%)	25,918 (4.1%)	25,385 (4.0%)	1,766 (1.6%)	1,759 (1.6%)
Psychiatric hospitalization: mean (sd)	0.03 (0.23)	0.03 (0.23)	0.01 (0.13)	0.01 (0.16)	0.03 (0.21)	0.03 (0.21)	0.01 (0.11)	0.01 (0.11)
Psychiatric office visit: mean (sd)	2.00 (6.75)	1.97 (7.14)	1.49 (4.48)	1.48 (4.90)	2.11 (8.82)	2.03 (8.89)	1.30 (4.51)	1.29 (4.47)
Anticonvulsants/antiepileptic drugs: n (%)	11,282 (6.3%)	11,129 (6.3%)	3,724 (7.8%)	3,678 (7.7%)	45,397 (7.1%)	44,254 (6.9%)	9,577 (8.9%)	9,330 (8.7%)
SSRIs (without sertraline): n (%)	30,122 (16.9%)	29,716 (16.7%)	1,615 (3.4%)	1,611 (3.4%)	103,518 (16.2%)	101,620 (15.9%)	3,566 (3.3%)	3,544 (3.3%)
SNRIs: n (%)	8,915 (5.0%)	8,827 (5.0%)	886 (1.8%)	886 (1.8%)	31,958 (5.0%)	31,401 (4.9%)	2,016 (1.9%)	2,005 (1.9%)
Tricyclic antidepressants: n (%)	4,084 (2.3%)	4,050 (2.3%)	1,366 (2.8%)	1,334 (2.8%)	15,920 (2.5%)	15,596 (2.4%)	3,539 (3.3%)	3,400 (3.2%)
Antipsychotics: n (%)	7,248 (4.1%)	7,157 (4.0%)	2,393 (5.0%)	2,352 (4.9%)	28,143 (4.4%)	27,438 (4.3%)	6,088 (5.7%)	5,939 (5.5%)
Benzodiazepines: n (%)	36,505 (20.5%)	36,002 (20.2%)	10,024 (20.9%)	9,941 (20.7%)	135,024 (21.1%)	132,844 (20.8%)	23,506 (21.9%)	23,177 (21.6%)
Non-BZD sedative hypnotics: n (%)	19,560 (11.0%)	19,359 (10.9%)	5,446 (11.3%)	5,407 (11.3%)	70,702 (11.1%)	69,718 (10.9%)	13,486 (12.6%)	13,272 (12.4%)
Outpatient visits: mean (sd)	0.22 (0.47)	0.22 (0.47)	0.22 (0.47)	0.22 (0.45)	0.19 (0.49)	0.19 (0.44)	0.16 (0.41)	0.16 (0.45)
Inpatient hospitalization: mean (sd)	0.01 (0.10)	0.01 (0.10)	0.00 (0.12)	0.00 (0.08)	0.00 (0.01)	0.00 (0.01)	0.00 (0.01)	0.00 (0.01)
ED visit: mean (sd)	0.29 (0.94)	0.28 (0.90)	0.16 (0.62)	0.16 (0.64)	0.41 (1.32)	0.40 (1.28)	0.21 (0.75)	0.20 (0.76)
Number of distinct medication prescriptions: mean (sd)	4.39 (4.18)	4.36 (4.19)	5.85 (4.33)	5.81 (4.23)	4.76 (4.53)	4.72 (4.51)	6.77 (4.84)	6.71 (4.74)
Combined Comorbidity Score: mean (sd)	0.31 (1.05)	0.31 (1.07)	0.25 (0.98)	0.24 (0.99)	0.35 (1.13)	0.34 (1.15)	0.24 (0.98)	0.23 (0.96)

Table R- Characteristics of patients included in the evaluation of comparative outcomes for *alendronate* between generic or authorized generics (AG) and brand initiators before 1:1 propensity score matching in each database

	Optum				Truven			
	Generic initiators	Brand initiators	AG initiators	Brand initiators	Generic initiators	Brand initiators	AG initiators	Brand initiators
Cohort selection steps								
Total number of patients filling prescriptions for the version of interest after 6-month continuous enrollment	97,191		59,484		659,262		627,150	
Patients meeting new drug use restriction	59,402		29,543		285,497		302,521	
Total eligible by exposure group	32,289	27,113	2,430	27,113	169,015	113,572	11,959	287,545
Baseline characteristics								
Age: mean (sd)	59.41 (9.57)	58.78 (9.95)	60.00 (10.12)	58.78 (9.95)	63.77 (11.64)	64.30 (11.84)	63.48 (11.93)	64.82 (12.05)
Gender								
MALE: n (%)	2,970 (9.2%)	2,645 (9.8%)	217 (8.9%)	2,645 (9.8%)	17,184 (10.2%)	12,770 (11.2%)	1,234 (10.3%)	29,023 (10.1%)
FEMALE: n (%)	29,316 (90.8%)	24,466 (90.2%)	2,212 (91.0%)	24,466 (90.2%)	151,831 (89.8%)	100,802 (88.8%)	10,725 (89.7%)	258,522 (89.9%)
UNKNOWN: n (%)	3 (0.0%)	2 (0.0%)	1 (0.0%)	2 (0.0%)	0 (0%)	0 (0%)	0 (0%)	0 (0%)
Region								
Northeast: n (%)	2,384 (7.4%)	2,760 (10.2%)	422 (17.4%)	2,760 (10.2%)	24,844 (14.7%)	11,336 (10.0%)	2,315 (19.4%)	27,163 (9.4%)
Midwest: n (%)	8,596 (26.6%)	7,568 (27.9%)	564 (23.2%)	7,568 (27.9%)	54,008 (32.0%)	39,424 (34.7%)	4,016 (33.6%)	90,238 (31.4%)
South: n (%)	16,244 (50.3%)	12,346 (45.5%)	1,229 (50.6%)	12,346 (45.5%)	55,394 (32.8%)	41,036 (36.1%)	3,808 (31.8%)	99,244 (34.5%)
West: n (%)	5,049 (15.6%)	4,421 (16.3%)	215 (8.8%)	4,421 (16.3%)	32,603 (19.3%)	21,334 (18.8%)	1,696 (14.2%)	69,419 (24.1%)
Unknown/other: n (%)	16 (0.0%)	18 (0.1%)	0 (0.0%)	18 (0.1%)	2,166 (1.3%)	442 (0.4%)	124 (1.0%)	1,481 (0.5%)
Osteoporosis: n (%)	12,999 (40.3%)	10,442 (38.5%)	973 (40.0%)	10,442 (38.5%)	51,859 (30.7%)	30,971 (27.3%)	3,466 (29.0%)	85,571 (29.8%)
Kyphosis: n (%)	450 (1.4%)	374 (1.4%)	24 (1.0%)	374 (1.4%)	1,587 (0.9%)	829 (0.7%)	99 (0.8%)	2,139 (0.7%)
Vertebral facture: n (%)	758 (2.3%)	624 (2.3%)	48 (2.0%)	624 (2.3%)	4,355 (2.6%)	2,927 (2.6%)	336 (2.8%)	8,016 (2.8%)
Humerus fracture: n (%)	152 (0.5%)	129 (0.5%)	11 (0.5%)	129 (0.5%)	1,009 (0.6%)	584 (0.5%)	69 (0.6%)	1,665 (0.6%)
Wrist fracture: n (%)	306 (0.9%)	284 (1.0%)	19 (0.8%)	284 (1.0%)	1,752 (1.0%)	1,073 (0.9%)	135 (1.1%)	2,880 (1.0%)
Hip fracture: n (%)	301 (0.9%)	259 (1.0%)	19 (0.8%)	259 (1.0%)	2,077 (1.2%)	1,471 (1.3%)	163 (1.4%)	4,249 (1.5%)

Pelvis fracture: n (%)	127 (0.4%)	122 (0.4%)	9 (0.4%)	122 (0.4%)	727 (0.4%)	553 (0.5%)	68 (0.6%)	1,440 (0.5%)
Any other fractures: n (%)	1,516 (4.7%)	1,279 (4.7%)	109 (4.5%)	1,279 (4.7%)	7,586 (4.5%)	4,751 (4.2%)	541 (4.5%)	12,826 (4.5%)
Alzheimer disease or other dementia: n (%)	513 (1.6%)	374 (1.4%)	48 (2.0%)	374 (1.4%)	3,403 (2.0%)	1,760 (1.5%)	239 (2.0%)	4,833 (1.7%)
Asthma or chronic obstructive pulmonary disease: n (%)	2,807 (8.7%)	2,326 (8.6%)	230 (9.5%)	2,326 (8.6%)	12,265 (7.3%)	7,446 (6.6%)	823 (6.9%)	18,896 (6.6%)
Cataracts: n (%)	2,533 (7.8%)	2,027 (7.5%)	210 (8.6%)	2,027 (7.5%)	13,111 (7.8%)	8,246 (7.3%)	892 (7.5%)	20,812 (7.2%)
Crohn disease or gastroenteritis: n (%)	726 (2.2%)	697 (2.6%)	56 (2.3%)	697 (2.6%)	3,241 (1.9%)	2,115 (1.9%)	238 (2.0%)	5,059 (1.8%)
Depression: n (%)	923 (2.9%)	755 (2.8%)	65 (2.7%)	755 (2.8%)	3,277 (1.9%)	1,608 (1.4%)	197 (1.6%)	3,890 (1.4%)
Diabetes mellitus: n (%)	3,603 (11.2%)	2,561 (9.4%)	253 (10.4%)	2,561 (9.4%)	19,651 (11.6%)	11,681 (10.3%)	1,323 (11.1%)	25,997 (9.0%)
History of falls, syncope, or gait abnormality: n (%)	2,466 (7.6%)	1,903 (7.0%)	171 (7.0%)	1,903 (7.0%)	10,289 (6.1%)	5,295 (4.7%)	648 (5.4%)	13,570 (4.7%)
Hyperthyroidism: n (%)	444 (1.4%)	336 (1.2%)	29 (1.2%)	336 (1.2%)	1,684 (1.0%)	994 (0.9%)	121 (1.0%)	2,468 (0.9%)
Hyperparathyroidism: n (%)	260 (0.8%)	164 (0.6%)	23 (0.9%)	164 (0.6%)	1,133 (0.7%)	592 (0.5%)	65 (0.5%)	1,384 (0.5%)
Ischemic stroke: n (%)	225 (0.7%)	174 (0.6%)	18 (0.7%)	174 (0.6%)	1,311 (0.8%)	805 (0.7%)	106 (0.9%)	1,692 (0.6%)
Liver disease: n (%)	822 (2.5%)	713 (2.6%)	63 (2.6%)	713 (2.6%)	3,138 (1.9%)	1,625 (1.4%)	226 (1.9%)	3,881 (1.3%)
Malignant neoplasm: n (%)	2,851 (8.8%)	2,728 (10.1%)	214 (8.8%)	2,728 (10.1%)	15,222 (9.0%)	10,616 (9.3%)	1,107 (9.3%)	26,346 (9.2%)
Overweight or obese: n (%)	851 (2.6%)	552 (2.0%)	54 (2.2%)	552 (2.0%)	2,456 (1.5%)	676 (0.6%)	153 (1.3%)	1,434 (0.5%)
Parkinson disease: n (%)	112 (0.3%)	99 (0.4%)	11 (0.5%)	99 (0.4%)	848 (0.5%)	573 (0.5%)	57 (0.5%)	1,472 (0.5%)
Renal disease: n (%)	364 (1.1%)	239 (0.9%)	24 (1.0%)	239 (0.9%)	1,756 (1.0%)	693 (0.6%)	95 (0.8%)	1,620 (0.6%)
Rheumatoid arthritis: n (%)	1,417 (4.4%)	1,018 (3.8%)	119 (4.9%)	1,018 (3.8%)	6,379 (3.8%)	3,808 (3.4%)	458 (3.8%)	8,997 (3.1%)
Oral glucocorticoids: n (%)	6,865 (21.3%)	5,300 (19.5%)	500 (20.6%)	5,300 (19.5%)	36,201 (21.4%)	24,462 (21.5%)	2,629 (22.0%)	62,764 (21.8%)
Anticonvulsants/antiepileptic drugs: n (%)	2,567 (8.0%)	1,732 (6.4%)	190 (7.8%)	1,732 (6.4%)	13,526 (8.0%)	8,458 (7.4%)	997 (8.3%)	19,803 (6.9%)
Benzodiazepines: n (%)	4,630 (14.3%)	3,537 (13.0%)	403 (16.6%)	3,537 (13.0%)	23,526 (13.9%)	15,890 (14.0%)	1,736 (14.5%)	41,529 (14.4%)
SSRIs: n (%)	5,685 (17.6%)	4,206 (15.5%)	391 (16.1%)	4,206 (15.5%)	28,375 (16.8%)	18,365 (16.2%)	1,980 (16.6%)	44,595 (15.5%)
Beta-blockers: n (%)	4,870 (15.1%)	3,728 (13.7%)	341 (14.0%)	3,728 (13.7%)	32,556 (19.3%)	23,086 (20.3%)	2,491 (20.8%)	57,375 (20.0%)
Proton pump inhibitors: n (%)	4,439 (13.7%)	3,809 (14.0%)	328 (13.5%)	3,809 (14.0%)	32,017 (18.9%)	23,902 (21.0%)	2,264 (18.9%)	54,927 (19.1%)
Opioids: n (%)	9,277 (28.7%)	7,122 (26.3%)	684 (28.1%)	7,122 (26.3%)	48,658 (28.8%)	33,081 (29.1%)	3,685 (30.8%)	85,689 (29.8%)
Other anti-osteoporosis medications (without alendronate): n (%)	4,721 (14.6%)	2,616 (9.6%)	292 (12.0%)	2,616 (9.6%)	29,794 (17.6%)	24,682 (21.7%)	2,457 (20.5%)	40,654 (14.1%)
Outpatient visits (occurrence): mean (sd)	0.41 (0.56)	0.39 (0.56)	0.36 (0.53)	0.39 (0.56)	0.28 (0.51)	0.24 (0.73)	0.27 (0.51)	0.23 (0.60)

Inpatient hospitalization (occurrence): mean (sd)	0.01 (0.15)	0.01 (0.14)	0.01 (0.09)	0.01 (0.14)	0.00 (0.01)	0.00 (0.01)	0.00 (0.01)	0.00 (0.01)
ED visit (occurrence): mean (sd)	0.17 (0.64)	0.17 (0.64)	0.16 (0.57)	0.17 (0.64)	0.22 (0.83)	0.20 (0.71)	0.24 (0.76)	0.21 (0.76)
Number of distinct medication prescriptions: mean (sd)	6.71 (4.67)	6.26 (4.65)	6.68 (4.72)	6.26 (4.65)	7.11 (4.86)	7.39 (5.07)	7.32 (4.90)	7.23 (5.00)
Combined Comorbidity Score: mean (sd)	0.29 (1.27)	0.30 (1.25)	0.32 (1.29)	0.30 (1.25)	0.26 (1.19)	0.25 (1.11)	0.27 (1.17)	0.25 (1.11)

Table S- Characteristics of patients included in the evaluation of comparative outcomes for *amlodipine* between generic or authorized generics (AG) and brand initiators before 1:1 propensity score matching in each database

	Optum				Truven			
	Generic initiators	Brand initiators	AG initiators	Brand initiators	Generic initiators	Brand initiators	AG initiators	Brand initiators
Cohort selection steps								
Total number of patients filling prescriptions for the version of interest after 6-month continuous enrollment	228,673		166,273		2,464,127		1,369,663	
Patients meeting new drug use restriction	162,366		100,190		1,312,094		515,057	
Total eligible by exposure group	87,454	74,912	25,278	74,912	1,234,533	32,740	417,590	32,740
Baseline characteristics								
Age: mean (sd)	53.98 (12.34)	54.79 (14.76)	56.06 (12.64)	54.79 (14.76)	57.89 (14.39)	60.64 (14.85)	60.90 (14.30)	60.64 (14.85)
Gender								
MALE: n (%)	46,532 (53.2%)	38,511 (51.4%)	13,747 (54.4%)	38,511 (51.4%)	620,983 (50.3%)	14,896 (45.5%)	208,504 (49.9%)	14,896 (45.5%)
FEMALE: n (%)	40,916 (46.8%)	36,391 (48.6%)	11,527 (45.6%)	36,391 (48.6%)	613,550 (49.7%)	17,844 (54.5%)	209,086 (50.1%)	17,844 (54.5%)
UNKNOWN: n (%)	6 (0.0%)	10 (0.0%)	4 (0.0%)	10 (0.0%)	0 (0%)	0 (0%)	0 (0%)	0 (0%)
Region								
Northeast: n (%)	7,541 (8.6%)	8,753 (11.7%)	1,962 (7.8%)	8,753 (11.7%)	216,077 (17.5%)	4,230 (12.9%)	52,714 (12.6%)	4,228 (12.9%)
Midwest: n (%)	21,749 (24.9%)	21,149 (28.2%)	4,389 (17.4%)	21,149 (28.2%)	336,456 (27.3%)	9,076 (27.7%)	80,759 (19.3%)	9,076 (27.7%)
South: n (%)	49,298 (56.4%)	35,969 (48.0%)	15,019 (59.4%)	35,969 (48.0%)	504,670 (40.9%)	13,780 (42.1%)	157,852 (37.8%)	13,780 (42.1%)
West: n (%)	8,829 (10.1%)	8,937 (11.9%)	3,905 (15.4%)	8,937 (11.9%)	147,468 (11.9%)	5,410 (16.5%)	122,270 (29.3%)	5,412 (16.5%)
Unknown/other: n (%)			3 (0.0%)	104 (0.1%)	29,862 (2.4%)	244 (0.7%)	3,995 (1.0%)	244 (0.7%)
Hyperlipidemia: n (%)	37,627 (43.0%)	30,165 (40.3%)	11,845 (46.9%)	30,165 (40.3%)	388,945 (31.5%)	7,592 (23.2%)	128,626 (30.8%)	7,592 (23.2%)
Diabetes mellitus: n (%)	17,890 (20.5%)	14,458 (19.3%)	5,320 (21.0%)	14,458 (19.3%)	245,642 (19.9%)	5,936 (18.1%)	86,532 (20.7%)	5,935 (18.1%)
Hypertension: n (%)	66,210 (75.7%)	55,974 (74.7%)	19,317 (76.4%)	55,974 (74.7%)	807,455 (65.4%)	19,530 (59.7%)	260,839 (62.5%)	19,530 (59.7%)

Myocardial Infarction: n (%)	1,402 (1.6%)	1,561 (2.1%)	398 (1.6%)	1,561 (2.1%)	20,213 (1.6%)	637 (1.9%)	5,811 (1.4%)	637 (1.9%)
Angina: n (%)	3,605 (4.1%)	4,788 (6.4%)	1,066 (4.2%)	4,788 (6.4%)	43,891 (3.6%)	1,613 (4.9%)	14,626 (3.5%)	1,612 (4.9%)
Heart failure: n (%)	3,561 (4.1%)	4,339 (5.8%)	1,076 (4.3%)	4,339 (5.8%)	54,009 (4.4%)	1,835 (5.6%)	17,364 (4.2%)	1,835 (5.6%)
Other forms of chronic heart diseases: n (%)	9,545 (10.9%)	10,702 (14.3%)	3,157 (12.5%)	10,702 (14.3%)	132,124 (10.7%)	4,304 (13.1%)	45,763 (11.0%)	4,301 (13.1%)
Cerebrovascular accident: n (%)	2,725 (3.1%)	3,005 (4.0%)	771 (3.1%)	3,005 (4.0%)	45,585 (3.7%)	1,429 (4.4%)	13,829 (3.3%)	1,429 (4.4%)
Venous thromboembolism: n (%)	1,348 (1.5%)	1,409 (1.9%)	409 (1.6%)	1,409 (1.9%)	22,184 (1.8%)	623 (1.9%)	6,485 (1.6%)	623 (1.9%)
Atrial fibrillation: n (%)	2,688 (3.1%)	3,035 (4.1%)	896 (3.5%)	3,035 (4.1%)	49,697 (4.0%)	1,600 (4.9%)	17,713 (4.2%)	1,601 (4.9%)
Overweight or obese: n (%)	6,081 (7.0%)	4,469 (6.0%)	1,525 (6.0%)	4,469 (6.0%)	67,205 (5.4%)	678 (2.1%)	19,652 (4.7%)	679 (2.1%)
Tobacco use: n (%)	5,272 (6.0%)	4,206 (5.6%)	1,301 (5.1%)	4,206 (5.6%)	52,902 (4.3%)	613 (1.9%)	13,928 (3.3%)	613 (1.9%)
Alcohol abuse or dependence: n (%)	1,066 (1.2%)	1,101 (1.5%)	271 (1.1%)	1,101 (1.5%)	12,631 (1.0%)	212 (0.6%)	3,453 (0.8%)	212 (0.6%)
Renal disease: n (%)	3,306 (3.8%)	3,089 (4.1%)	901 (3.6%)	3,089 (4.1%)	44,692 (3.6%)	930 (2.8%)	16,805 (4.0%)	931 (2.8%)
Liver disease: n (%)	2,559 (2.9%)	2,401 (3.2%)	747 (3.0%)	2,401 (3.2%)	30,630 (2.5%)	639 (2.0%)	9,517 (2.3%)	639 (2.0%)
Aspirin: n (%)	62 (0.1%)	150 (0.2%)	15 (0.1%)	150 (0.2%)	7,668 (0.6%)	259 (0.8%)	3,254 (0.8%)	259 (0.8%)
Antiplatelets: n (%)	3,652 (4.2%)	3,781 (5.0%)	1,228 (4.9%)	3,781 (5.0%)	61,140 (5.0%)	2,070 (6.3%)	22,663 (5.4%)	2,070 (6.3%)
Statins: n (%)	24,879 (28.4%)	18,954 (25.3%)	8,577 (33.9%)	18,954 (25.3%)	381,977 (30.9%)	9,797 (29.9%)	152,601 (36.5%)	9,797 (29.9%)
Other lipid lowering agents: n (%)	7,294 (8.3%)	6,302 (8.4%)	2,607 (10.3%)	6,302 (8.4%)	82,262 (6.7%)	3,213 (9.8%)	34,716 (8.3%)	3,214 (9.8%)
Insulin preparations: n (%)	3,300 (3.8%)	2,488 (3.3%)	934 (3.7%)	2,488 (3.3%)	36,343 (2.9%)	796 (2.4%)	11,929 (2.9%)	796 (2.4%)
Oral hypoglycemic agents: n (%)	11,111 (12.7%)	9,032 (12.1%)	3,419 (13.5%)	9,032 (12.1%)	168,825 (13.7%)	4,298 (13.1%)	65,101 (15.6%)	4,298 (13.1%)
ACE inhibitors: n (%)	31,734 (36.3%)	23,883 (31.9%)	8,900 (35.2%)	23,883 (31.9%)	431,095 (34.9%)	10,013 (30.6%)	150,919 (36.1%)	10,012 (30.6%)
ARBs: n (%)	17,154 (19.6%)	15,480 (20.7%)	5,799 (22.9%)	15,480 (20.7%)	265,462 (21.5%)	7,629 (23.3%)	98,186 (23.5%)	7,628 (23.3%)

Calcium channel blockers (without amlodipine): n (%)	6,076 (6.9%)	5,185 (6.9%)	1,982 (7.8%)	5,185 (6.9%)	68,905 (5.6%)	2,709 (8.3%)	38,364 (9.2%)	2,709 (8.3%)
Diuretics: n (%)	33,890 (38.8%)	29,875 (39.9%)	9,758 (38.6%)	29,875 (39.9%)	473,587 (38.4%)	12,862 (39.3%)	168,855 (40.4%)	12,863 (39.3%)
Beta-blockers: n (%)	22,185 (25.4%)	21,416 (28.6%)	6,361 (25.2%)	21,416 (28.6%)	330,317 (26.8%)	9,919 (30.3%)	124,706 (29.9%)	9,921 (30.3%)
Anticoagulants: n (%)	2,348 (2.7%)	2,575 (3.4%)	748 (3.0%)	2,575 (3.4%)	45,948 (3.7%)	1,493 (4.6%)	16,767 (4.0%)	1,494 (4.6%)
NSAIDs: n (%)	12,013 (13.7%)	10,378 (13.9%)	3,395 (13.4%)	10,378 (13.9%)	182,099 (14.8%)	4,167 (12.7%)	60,007 (14.4%)	4,169 (12.7%)
Coxibs: n (%)	1,595 (1.8%)	1,817 (2.4%)	595 (2.4%)	1,817 (2.4%)	21,543 (1.7%)	924 (2.8%)	8,388 (2.0%)	922 (2.8%)
Outpatient visits: mean (sd)	0.14 (0.39)	0.13 (0.40)	0.13 (0.39)	0.13 (0.40)	0.13 (0.39)	0.10 (0.33)	0.10 (0.34)	0.10 (0.33)
Inpatient hospitalization: mean (sd)	0.01 (0.15)	0.02 (0.19)	0.02 (0.17)	0.02 (0.19)	0.00 (0.02)	0.00 (0.01)	0.00 (0.01)	0.00 (0.01)
ED visit: mean (sd)	0.32 (0.91)	0.40 (1.08)	0.30 (0.86)	0.40 (1.08)	0.44 (1.22)	0.40 (1.01)	0.36 (1.09)	0.40 (1.01)
Number of distinct medication prescriptions: mean (sd)	5.25 (4.60)	5.53 (4.85)	5.54 (4.70)	5.53 (4.85)	5.76 (4.82)	6.12 (5.30)	6.09 (4.85)	6.12 (5.30)
Combined Comorbidity Score: mean (sd)	0.13 (1.65)	0.30 (1.81)	0.16 (1.71)	0.30 (1.81)	0.24 (1.67)	0.31 (1.62)	0.25 (1.62)	0.31 (1.62)

Table T- Characteristics of patients included in the evaluation of comparative outcomes for *amlodipine-benazepril* between generic or authorized generics (AG) and brand initiators before 1:1 propensity score matching in each database

	Optum				Truven			
	Generic initiators	Brand initiators	AG initiators	Brand initiators	Generic initiators	Brand initiators	AG initiators	Brand initiators
Cohort selection steps								
Total number of patients filling prescriptions for the version of interest after 6-month continuous enrollment	78,491		69,255		445,257		388,039	
Patients meeting new drug use restriction	53,638		44,919		166,986		136,170	
Total eligible by exposure group	14,718	38,920	5,999	38,920	53,891	109,164	23,175	109,167
Baseline characteristics								
Age: mean (sd)	52.49 (11.66)	52.30 (11.21)	53.75 (11.76)	52.30 (11.21)	54.62 (12.64)	56.45 (13.19)	54.88 (12.35)	56.44 (13.19)
Gender								
MALE: n (%)	8,675 (58.9%)	23,279 (59.8%)	3,412 (56.9%)	23,279 (59.8%)	30,010 (55.7%)	59,912 (54.9%)	13,001 (56.1%)	59,915 (54.9%)
FEMALE: n (%)	6,041 (41.0%)	15,636 (40.2%)	2,587 (43.1%)	15,636 (40.2%)	23,881 (44.3%)	49,252 (45.1%)	10,174 (43.9%)	49,252 (45.1%)
UNKNOWN: n (%)	2 (0.0%)	5 (0.0%)	0 (0.0%)	5 (0.0%)	0 (0%)	0 (0%)	0 (0%)	0 (0%)
Region								
Northeast: n (%)	927 (6.3%)	2,847 (7.3%)	258 (4.3%)	2,847 (7.3%)	5,558 (10.3%)	8,280 (7.6%)	2,718 (11.7%)	8,280 (7.6%)
Midwest: n (%)	2,458 (16.7%)	7,874 (20.2%)	911 (15.2%)	7,874 (20.2%)	14,670 (27.2%)	27,561 (25.2%)	6,773 (29.2%)	27,562 (25.2%)
South: n (%)	10,025 (68.1%)	24,468 (62.9%)	4,473 (74.6%)	24,468 (62.9%)	27,898 (51.8%)	60,201 (55.1%)	11,627 (50.2%)	60,204 (55.1%)
West: n (%)	1,305 (8.9%)	3,713 (9.5%)	351 (5.9%)	3,713 (9.5%)	5,244 (9.7%)	12,459 (11.4%)	1,722 (7.4%)	12,458 (11.4%)
Unknown/other: n (%)	3 (0.0%)	18 (0.0%)	6 (0.1%)	18 (0.0%)	521 (1.0%)	663 (0.6%)	335 (1.4%)	663 (0.6%)
Hyperlipidemia: n (%)	5,653 (38.4%)	17,008 (43.7%)	2,335 (38.9%)	17,008 (43.7%)	14,020 (26.0%)	26,713 (24.5%)	6,572 (28.4%)	26,715 (24.5%)
Diabetes mellitus: n (%)	2,468 (16.8%)	7,210 (18.5%)	1,124 (18.7%)	7,210 (18.5%)	8,271 (15.3%)	18,909 (17.3%)	3,641 (15.7%)	18,908 (17.3%)
Hypertension: n (%)	10,547 (71.7%)	32,310 (83.0%)	4,281 (71.4%)	32,310 (83.0%)	32,014 (59.4%)	76,708 (70.3%)	13,844 (59.7%)	76,707 (70.3%)

Myocardial Infarction: n (%)	66 (0.4%)	215 (0.6%)	24 (0.4%)	215 (0.6%)	229 (0.4%)	634 (0.6%)	103 (0.4%)	634 (0.6%)
Angina: n (%)	308 (2.1%)	1,211 (3.1%)	117 (2.0%)	1,211 (3.1%)	925 (1.7%)	2,998 (2.7%)	409 (1.8%)	2,998 (2.7%)
Heart failure: n (%)	217 (1.5%)	888 (2.3%)	90 (1.5%)	888 (2.3%)	722 (1.3%)	2,336 (2.1%)	318 (1.4%)	2,336 (2.1%)
Other forms of chronic heart diseases: n (%)	857 (5.8%)	3,059 (7.9%)	408 (6.8%)	3,059 (7.9%)	2,875 (5.3%)	8,436 (7.7%)	1,269 (5.5%)	8,436 (7.7%)
Cerebrovascular accident: n (%)	201 (1.4%)	740 (1.9%)	93 (1.6%)	740 (1.9%)	734 (1.4%)	2,168 (2.0%)	326 (1.4%)	2,168 (2.0%)
Venous thromboembolism: n (%)	122 (0.8%)	279 (0.7%)	44 (0.7%)	279 (0.7%)	341 (0.6%)	814 (0.7%)	135 (0.6%)	814 (0.7%)
Atrial fibrillation: n (%)	180 (1.2%)	642 (1.6%)	83 (1.4%)	642 (1.6%)	722 (1.3%)	2,113 (1.9%)	361 (1.6%)	2,114 (1.9%)
Overweight or obese: n (%)	849 (5.8%)	2,485 (6.4%)	307 (5.1%)	2,485 (6.4%)	1,413 (2.6%)	1,818 (1.7%)	605 (2.6%)	1,818 (1.7%)
Tobacco use: n (%)	661 (4.5%)	1,764 (4.5%)	256 (4.3%)	1,764 (4.5%)	1,043 (1.9%)	1,560 (1.4%)	514 (2.2%)	1,560 (1.4%)
Alcohol abuse or dependence: n (%)	124 (0.8%)	329 (0.8%)	32 (0.5%)	329 (0.8%)	277 (0.5%)	490 (0.4%)	131 (0.6%)	490 (0.4%)
Renal disease: n (%)	190 (1.3%)	575 (1.5%)	82 (1.4%)	575 (1.5%)	516 (1.0%)	1,287 (1.2%)	216 (0.9%)	1,286 (1.2%)
Liver disease: n (%)	306 (2.1%)	814 (2.1%)	101 (1.7%)	814 (2.1%)	749 (1.4%)	1,541 (1.4%)	328 (1.4%)	1,541 (1.4%)
Aspirin: n (%)	4 (0.0%)	14 (0.0%)	1 (0.0%)	14 (0.0%)	244 (0.5%)	618 (0.6%)	85 (0.4%)	618 (0.6%)
Antiplatelets: n (%)	303 (2.1%)	1,146 (2.9%)	110 (1.8%)	1,146 (2.9%)	1,452 (2.7%)	4,704 (4.3%)	587 (2.5%)	4,704 (4.3%)
Statins: n (%)	2,931 (19.9%)	8,791 (22.6%)	1,109 (18.5%)	8,791 (22.6%)	11,812 (21.9%)	29,073 (26.6%)	4,699 (20.3%)	29,073 (26.6%)
Other lipid lowering agents: n (%)	957 (6.5%)	3,338 (8.6%)	386 (6.4%)	3,338 (8.6%)	3,799 (7.0%)	10,195 (9.3%)	1,469 (6.3%)	10,195 (9.3%)
Insulin preparations: n (%)	296 (2.0%)	894 (2.3%)	125 (2.1%)	894 (2.3%)	700 (1.3%)	1,804 (1.7%)	307 (1.3%)	1,804 (1.7%)
Oral hypoglycemic agents: n (%)	1,443 (9.8%)	4,729 (12.2%)	616 (10.3%)	4,729 (12.2%)	5,523 (10.2%)	14,707 (13.5%)	2,340 (10.1%)	14,705 (13.5%)
ACE inhibitors: n (%)	3,201 (21.7%)	10,055 (25.8%)	1,247 (20.8%)	10,055 (25.8%)	13,249 (24.6%)	33,987 (31.1%)	5,390 (23.3%)	33,988 (31.1%)
ARBs: n (%)	1,451 (9.9%)	5,677 (14.6%)	524 (8.7%)	5,677 (14.6%)	5,200 (9.6%)	17,155 (15.7%)	2,011 (8.7%)	17,152 (15.7%)
Calcium channel blockers (without amlodipine-benazepril): n (%)	905 (6.1%)	2,884 (7.4%)	335 (5.6%)	2,884 (7.4%)	3,305 (6.1%)	10,045 (9.2%)	1,331 (5.7%)	10,045 (9.2%)
Diuretics: n (%)	3,550 (24.1%)	12,545 (32.2%)	1,333 (22.2%)	12,545 (32.2%)	13,548 (25.1%)	37,698 (34.5%)	5,416 (23.4%)	37,697 (34.5%)
Beta-blockers: n (%)	2,473 (16.8%)	8,444 (21.7%)	852 (14.2%)	8,444 (21.7%)	9,606 (17.8%)	27,139 (24.9%)	3,958 (17.1%)	27,140 (24.9%)

Anticoagulants: n (%)	201 (1.4%)	668 (1.7%)	67 (1.1%)	668 (1.7%)	870 (1.6%)	2,576 (2.4%)	380 (1.6%)	2,576 (2.4%)
NSAIDs: n (%)	1,798 (12.2%)	5,416 (13.9%)	676 (11.3%)	5,416 (13.9%)	6,683 (12.4%)	16,335 (15.0%)	2,647 (11.4%)	16,335 (15.0%)
Coxibs: n (%)	219 (1.5%)	799 (2.1%)	104 (1.7%)	799 (2.1%)	1,026 (1.9%)	3,076 (2.8%)	380 (1.6%)	3,076 (2.8%)
Outpatient visits: mean (sd)	0.14 (0.39)	0.13 (0.38)	0.11 (0.33)	0.13 (0.38)	0.12 (0.35)	0.10 (0.32)	0.11 (0.35)	0.10 (0.32)
Inpatient hospitalization: mean (sd)	0.00 (0.05)	0.00 (0.06)	0.00 (0.06)	0.00 (0.06)	0.00 (0.00)	0.00 (0.01)	0.00 (0.00)	0.00 (0.01)
ED visit: mean (sd)	0.18 (0.56)	0.20 (0.66)	0.18 (0.57)	0.20 (0.66)	0.24 (0.86)	0.25 (0.85)	0.24 (0.83)	0.25 (0.85)
Number of distinct medication prescriptions: mean (sd)	3.90 (3.91)	4.62 (4.17)	3.56 (3.83)	4.62 (4.17)	4.11 (4.40)	5.27 (4.62)	3.75 (4.06)	5.27 (4.62)
Combined Comorbidity Score: mean (sd)	-0.26 (1.06)	-0.32 (1.10)	-0.25 (1.01)	-0.32 (1.10)	-0.24 (0.97)	-0.28 (1.03)	-0.23 (0.98)	-0.28 (1.03)

Table U- Characteristics of patients included in the evaluation of comparative outcomes for *calcitonin salmon* between generic or authorized generics (AG) and brand initiators <u>before 1:1 propensity score matching</u> in each database

	Optum				Truven			
	Generic initiators	Brand initiators	AG initiators	Brand initiators	Generic initiators	Brand initiators	AG initiators	Brand initiators
Cohort selection steps								
Total number of patients filling prescriptions for the version of interest after 6-month continuous enrollment	8,864		8,442		67,478		62,915	
Patients meeting new drug use restriction	5,806		5,496		31,375		28,482	
Total eligible by exposure group	946	4,860	636	4,860	6,308	24,772	3,426	24,775
Baseline characteristics								
Age: mean (sd)	61.90 (12.26)	62.11 (11.94)	59.43 (12.58)	62.11 (11.94)	68.37 (13.73)	68.89 (14.01)	66.20 (14.76)	68.89 (14.01)
Gender								
MALE: n (%)	142 (15.0%)	590 (12.1%)	102 (16.0%)	590 (12.1%)	848 (13.4%)	3,291 (13.3%)	518 (15.1%)	3,291 (13.3%)
FEMALE: n (%)	804 (85.0%)	4,270 (87.9%)	534 (84.0%)	4,270 (87.9%)	5,460 (86.6%)	21,481 (86.7%)	2,908 (84.9%)	21,484 (86.7%)
UNKNOWN: n (%)	0 (0.0%)	0 (0.0%)	0 (0.0%)	0 (0.0%)	0 (0%)	0 (0%)	0 (0%)	0 (0%)
Region								
Northeast: n (%)	95 (10.0%)	479 (9.9%)	51 (8.0%)	479 (9.9%)	961 (15.2%)	2,970 (12.0%)	575 (16.8%)	2,971 (12.0%)
Midwest: n (%)	193 (20.4%)	1,039 (21.4%)	151 (23.7%)	1,039 (21.4%)	1,939 (30.7%)	8,012 (32.3%)	1,060 (30.9%)	8,014 (32.3%)
South: n (%)	452 (47.8%)	2,547 (52.4%)	310 (48.7%)	2,547 (52.4%)	2,243 (35.6%)	8,746 (35.3%)	1,182 (34.5%)	8,746 (35.3%)
West: n (%)	206 (21.8%)	791 (16.3%)	124 (19.5%)	791 (16.3%)	1,121 (17.8%)	4,866 (19.6%)	566 (16.5%)	4,866 (19.6%)
Unknown/other: n (%)	0 (0.0%)	4 (0.1%)	0 (0.0%)	4 (0.1%)	44 (0.7%)	178 (0.7%)	43 (1.3%)	178 (0.7%)
Outpatient visits: mean (sd)	0.29 (0.52)	0.27 (0.51)	0.27 (0.50)	0.27 (0.51)	0.18 (0.47)	0.15 (0.39)	0.20 (0.50)	0.15 (0.39)
Inpatient hospitalization: mean (sd)	0.05 (0.30)	0.06 (0.34)	0.06 (0.33)	0.06 (0.34)	0.00 (0.01)	0.00 (0.02)	0.00 (0.00)	0.00 (0.02)

ED visit: mean (sd)	0.40 (0.94)	0.43 (1.20)	0.48 (1.13)	0.43 (1.20)	0.51 (1.24)	0.53 (1.25)	0.63 (1.81)	0.53 (1.25)
Number of distinct medication prescriptions: mean (sd)	8.98 (5.80)	9.09 (6.07)	9.83 (6.34)	9.09 (6.07)	9.56 (5.75)	9.91 (6.08)	9.85 (6.04)	9.91 (6.08)
Combined Comorbidity Score: mean (sd)	0.85 (1.90)	0.84 (1.96)	0.88 (1.95)	0.84 (1.96)	0.74 (1.72)	0.79 (1.78)	0.86 (1.85)	0.78 (1.78)
Osteoporosis: n (%)	437 (46.2%)	2,047 (42.1%)	272 (42.8%)	2,047 (42.1%)	2,227 (35.3%)	7,572 (30.6%)	1,189 (34.7%)	7,572 (30.6%)
Kyphosis: n (%)	41 (4.3%)	168 (3.5%)	23 (3.6%)	168 (3.5%)	187 (3.0%)	477 (1.9%)	109 (3.2%)	477 (1.9%)
Vertebral facture: n (%)	164 (17.3%)	679 (14.0%)	124 (19.5%)	679 (14.0%)	1,035 (16.4%)	3,814 (15.4%)	652 (19.0%)	3,815 (15.4%)
Humerus fracture: n (%)	9 (1.0%)	65 (1.3%)	16 (2.5%)	65 (1.3%)	82 (1.3%)	306 (1.2%)	48 (1.4%)	307 (1.2%)
Wrist fracture: n (%)	16 (1.7%)	56 (1.2%)	12 (1.9%)	56 (1.2%)	88 (1.4%)	348 (1.4%)	51 (1.5%)	348 (1.4%)
Hip fracture: n (%)	21 (2.2%)	101 (2.1%)	11 (1.7%)	101 (2.1%)	166 (2.6%)	710 (2.9%)	87 (2.5%)	710 (2.9%)
Pelvis fracture: n (%)	23 (2.4%)	84 (1.7%)	11 (1.7%)	84 (1.7%)	118 (1.9%)	456 (1.8%)	62 (1.8%)	457 (1.8%)
Any other fractures: n (%)	97 (10.3%)	507 (10.4%)	82 (12.9%)	507 (10.4%)	614 (9.7%)	2,433 (9.8%)	421 (12.3%)	2,433 (9.8%)
Alzheimer disease or other dementia: n (%)	41 (4.3%)	236 (4.9%)	27 (4.2%)	236 (4.9%)	311 (4.9%)	1,388 (5.6%)	182 (5.3%)	1,389 (5.6%)
Asthma or chronic obstructive pulmonary disease: n (%)	122 (12.9%)	604 (12.4%)	80 (12.6%)	604 (12.4%)	669 (10.6%)	2,445 (9.9%)	396 (11.6%)	2,445 (9.9%)
Cataracts: n (%)	94 (9.9%)	497 (10.2%)	47 (7.4%)	497 (10.2%)	578 (9.2%)	2,224 (9.0%)	290 (8.5%)	2,224 (9.0%)
Crohn disease or gastroenteritis: n (%)	35 (3.7%)	193 (4.0%)	22 (3.5%)	193 (4.0%)	169 (2.7%)	742 (3.0%)	99 (2.9%)	742 (3.0%)
Depression: n (%)	29 (3.1%)	175 (3.6%)	21 (3.3%)	175 (3.6%)	166 (2.6%)	600 (2.4%)	115 (3.4%)	601 (2.4%)
Diabetes mellitus: n (%)	114 (12.1%)	615 (12.7%)	100 (15.7%)	615 (12.7%)	819 (13.0%)	3,200 (12.9%)	500 (14.6%)	3,201 (12.9%)
History of falls, syncope, or gait abnormality: n (%)	151 (16.0%)	753 (15.5%)	111 (17.5%)	753 (15.5%)	803 (12.7%)	2,944 (11.9%)	494 (14.4%)	2,945 (11.9%)
Hyperthyroidism: n (%)	18 (1.9%)	59 (1.2%)	10 (1.6%)	59 (1.2%)	52 (0.8%)	223 (0.9%)	41 (1.2%)	223 (0.9%)
Hyperparathyroidism: n (%)	15 (1.6%)	38 (0.8%)	9 (1.4%)	38 (0.8%)	56 (0.9%)	187 (0.8%)	36 (1.1%)	187 (0.8%)
Ischemic stroke: n (%)	12 (1.3%)	62 (1.3%)	7 (1.1%)	62 (1.3%)	99 (1.6%)	403 (1.6%)	63 (1.8%)	404 (1.6%)
Liver disease: n (%)	40 (4.2%)	162 (3.3%)	30 (4.7%)	162 (3.3%)	144 (2.3%)	539 (2.2%)	114 (3.3%)	539 (2.2%)
Malignant neoplasm: n (%)	120 (12.7%)	544 (11.2%)	67 (10.5%)	544 (11.2%)	677 (10.7%)	2,630 (10.6%)	368 (10.7%)	2,631 (10.6%)

Overweight or obese: n (%)	32 (3.4%)	128 (2.6%)	22 (3.5%)	128 (2.6%)	107 (1.7%)	316 (1.3%)	67 (2.0%)	316 (1.3%)
Parkinson disease: n (%)	9 (1.0%)	45 (0.9%)	5 (0.8%)	45 (0.9%)	74 (1.2%)	310 (1.3%)	33 (1.0%)	310 (1.3%)
Renal disease: n (%)	26 (2.7%)	108 (2.2%)	15 (2.4%)	108 (2.2%)	168 (2.7%)	597 (2.4%)	116 (3.4%)	597 (2.4%)
Rheumatoid arthritis: n (%)	50 (5.3%)	260 (5.3%)	30 (4.7%)	260 (5.3%)	282 (4.5%)	996 (4.0%)	165 (4.8%)	996 (4.0%)
Oral glucocorticoids: n (%)	245 (25.9%)	1,300 (26.7%)	198 (31.1%)	1,300 (26.7%)	1,756 (27.8%)	6,919 (27.9%)	1,035 (30.2%)	6,919 (27.9%)
Anticonvulsants/antiepileptic drugs: n (%)	131 (13.8%)	610 (12.6%)	102 (16.0%)	610 (12.6%)	822 (13.0%)	3,090 (12.5%)	493 (14.4%)	3,090 (12.5%)
Benzodiazepines: n (%)	216 (22.8%)	1,102 (22.7%)	168 (26.4%)	1,102 (22.7%)	1,370 (21.7%)	5,422 (21.9%)	784 (22.9%)	5,422 (21.9%)
SSRIs: n (%)	170 (18.0%)	1,010 (20.8%)	137 (21.5%)	1,010 (20.8%)	1,309 (20.8%)	5,473 (22.1%)	765 (22.3%)	5,473 (22.1%)
Beta-blockers: n (%)	184 (19.5%)	975 (20.1%)	122 (19.2%)	975 (20.1%)	1,686 (26.7%)	6,697 (27.0%)	839 (24.5%)	6,699 (27.0%)
Proton pump inhibitors: n (%)	245 (25.9%)	1,292 (26.6%)	165 (25.9%)	1,292 (26.6%)	1,983 (31.4%)	8,737 (35.3%)	1,074 (31.3%)	8,738 (35.3%)
Opioids: n (%)	447 (47.3%)	2,258 (46.5%)	357 (56.1%)	2,258 (46.5%)	3,063 (48.6%)	12,180 (49.2%)	1,769 (51.6%)	12,181 (49.2%)
Other anti-osteoporosis medications (without calcitonin): n (%)	249 (26.3%)	1,402 (28.8%)	177 (27.8%)	1,402 (28.8%)	1,771 (28.1%)	7,541 (30.4%)	902 (26.3%)	7,544 (30.5%)

Table V- Characteristics of patients included in the evaluation of comparative outcomes for *escitalopram* between generic or authorized generics (AG) and brand initiators before 1:1 propensity score matching in each database

	Optum				Truven			
	**Generic initiators**	**Brand initiators**	**AG initiators**	**Brand initiators**	**Generic initiators**	**Brand initiators**	**AG initiators**	**Brand initiators**
**Cohort selection steps**								
Total number of patients filling prescriptions for the version of interest after 6-month continuous enrollment	235,490		197,080		1,350,114		1,045,259	
Patients meeting new drug use restriction	136,104		107,633		643,412		425,365	
Total eligible by exposure group	54,051	82,053	25,580	82,053	321,070	322,342	103,017	322,348
**Baseline characteristics**								
Age: mean (sd)	39.10 (14.76)	39.86 (14.96)	39.25 (14.98)	39.86 (14.96)	42.43 (17.54)	42.52 (16.95)	42.61 (17.50)	42.52 (16.95)
Gender								
MALE: n (%)	17,996 (33.3%)	27,338 (33.3%)	8,802 (34.4%)	27,338 (33.3%)	103,142 (32.1%)	102,368 (31.8%)	32,963 (32.0%)	102,369 (31.8%)
FEMALE: n (%)	36,048 (66.7%)	54,707 (66.7%)	16,774 (65.6%)	54,707 (66.7%)	217,928 (67.9%)	219,974 (68.2%)	70,054 (68.0%)	219,979 (68.2%)
UNKNOWN: n (%)	7 (0.0%)	8 (0.0%)	4 (0.0%)	8 (0.0%)	0 (0%)	0 (0%)	0 (0%)	0 (0%)
Region								
Northeast: n (%)	5,609 (10.4%)	8,128 (9.9%)	2,022 (7.9%)	8,128 (9.9%)	**57,797 (18.0%)**	**63,612 (19.7%)**	**16,757 (16.3%)**	**63,612 (19.7%)**
Midwest: n (%)	14,102 (26.1%)	17,334 (21.1%)	6,310 (24.7%)	17,334 (21.1%)	71,818 (22.4%)	75,260 (23.3%)	23,121 (22.4%)	75,263 (23.3%)
South: n (%)	25,613 (47.4%)	44,632 (54.4%)	13,025 (50.9%)	44,632 (54.4%)	126,948 (39.5%)	131,372 (40.8%)	46,091 (44.7%)	131,375 (40.8%)
West: n (%)	8,716 (16.1%)	11,942 (14.6%)	4,214 (16.5%)	11,942 (14.6%)	54,163 (16.9%)	41,364 (12.8%)	15,016 (14.6%)	41,364 (12.8%)
Unknown/other: n (%)	11 (0.0%)	17 (0.0%)	9 (0.0%)	17 (0.0%)	10,344 (3.2%)	10,734 (3.3%)	2,032 (2.0%)	10,734 (3.3%)
Epilepsy: n (%)	714 (1.3%)	1,254 (1.5%)	357 (1.4%)	1,254 (1.5%)	4,605 (1.4%)	4,665 (1.4%)	1,453 (1.4%)	4,666 (1.4%)
Depression: n (%)	5,376 (9.9%)	10,034 (12.2%)	2,597 (10.2%)	10,034 (12.2%)	32,189 (10.0%)	37,520 (11.6%)	10,401 (10.1%)	37,520 (11.6%)
Anxiety: n (%)	5,694 (10.5%)	9,273 (11.3%)	2,668 (10.4%)	9,273 (11.3%)	28,003 (8.7%)	29,581 (9.2%)	8,883 (8.6%)	29,582 (9.2%)
Alcohol abuse or dependence: n (%)	1,204 (2.2%)	1,748 (2.1%)	578 (2.3%)	1,748 (2.1%)	5,516 (1.7%)	5,228 (1.6%)	1,766 (1.7%)	5,228 (1.6%)
Drug abuse or dependence: n (%)	1,354 (2.5%)	1,948 (2.4%)	604 (2.4%)	1,948 (2.4%)	6,534 (2.0%)	5,937 (1.8%)	2,144 (2.1%)	5,938 (1.8%)
Sleep disorder: n (%)	4,552 (8.4%)	8,741 (10.7%)	2,214 (8.7%)	8,741 (10.7%)	20,581 (6.4%)	24,435 (7.6%)	6,834 (6.6%)	24,436 (7.6%)
Psychotic disorder: n (%)	796 (1.5%)	1,274 (1.6%)	431 (1.7%)	1,274 (1.6%)	5,547 (1.7%)	4,945 (1.5%)	1,681 (1.6%)	4,945 (1.5%)

Personality disorder: n (%)	298 (0.6%)	494 (0.6%)	119 (0.5%)	494 (0.6%)	1,388 (0.4%)	1,444 (0.4%)	468 (0.5%)	1,444 (0.4%)
Adjustment reaction/post-traumatic stress disorder: n (%)	3,359 (6.2%)	5,548 (6.8%)	1,568 (6.1%)	5,548 (6.8%)	17,789 (5.5%)	18,744 (5.8%)	5,767 (5.6%)	18,744 (5.8%)
ADHD: n (%)	3,192 (5.9%)	4,378 (5.3%)	1,472 (5.8%)	4,378 (5.3%)	14,286 (4.4%)	13,083 (4.1%)	4,603 (4.5%)	13,083 (4.1%)
Delirium: n (%)	670 (1.2%)	1,214 (1.5%)	322 (1.3%)	1,214 (1.5%)	4,769 (1.5%)	4,556 (1.4%)	1,454 (1.4%)	4,557 (1.4%)
Bipolar disorder: n (%)	138 (0.3%)	274 (0.3%)	80 (0.3%)	274 (0.3%)	704 (0.2%)	869 (0.3%)	241 (0.2%)	869 (0.3%)
Other psychiatric disorder: n (%)	2,715 (5.0%)	4,720 (5.8%)	1,273 (5.0%)	4,720 (5.8%)	12,955 (4.0%)	14,057 (4.4%)	4,103 (4.0%)	14,057 (4.4%)
Psychiatric hospitalization: mean (sd)	0.03 (0.27)	0.04 (0.26)	0.04 (0.24)	0.04 (0.26)	0.03 (0.21)	0.03 (0.22)	0.03 (0.21)	0.03 (0.22)
Psychiatric office visit: mean (sd)	2.53 (11.21)	2.36 (6.78)	2.45 (8.29)	2.36 (6.78)	2.42 (9.72)	2.41 (7.33)	2.45 (8.89)	2.41 (7.33)
Anticonvulsants/antiepileptic drugs: n (%)	4,180 (7.7%)	6,212 (7.6%)	2,046 (8.0%)	6,212 (7.6%)	26,800 (8.3%)	25,610 (7.9%)	8,644 (8.4%)	25,611 (7.9%)
SSRIs (without escitalopram): n (%)	11,325 (21.0%)	15,084 (18.4%)	5,668 (22.2%)	15,084 (18.4%)	64,220 (20.0%)	65,555 (20.3%)	21,765 (21.1%)	65,555 (20.3%)
SNRIs: n (%)	2,915 (5.4%)	4,457 (5.4%)	1,358 (5.3%)	4,457 (5.4%)	16,561 (5.2%)	18,840 (5.8%)	5,545 (5.4%)	18,840 (5.8%)
Tricyclic antidepressants: n (%)	1,249 (2.3%)	1,854 (2.3%)	596 (2.3%)	1,854 (2.3%)	8,155 (2.5%)	7,991 (2.5%)	2,667 (2.6%)	7,991 (2.5%)
Antipsychotics: n (%)	2,249 (4.2%)	3,749 (4.6%)	1,109 (4.3%)	3,749 (4.6%)	14,426 (4.5%)	15,773 (4.9%)	4,665 (4.5%)	15,772 (4.9%)
Benzodiazepines: n (%)	12,845 (23.8%)	21,604 (26.3%)	6,316 (24.7%)	21,604 (26.3%)	76,471 (23.8%)	84,875 (26.3%)	25,194 (24.5%)	84,876 (26.3%)
Non-BZD sedative hypnotics: n (%)	6,236 (11.5%)	11,218 (13.7%)	3,017 (11.8%)	11,218 (13.7%)	36,841 (11.5%)	42,814 (13.3%)	12,452 (12.1%)	42,813 (13.3%)
Outpatient visits: mean (sd)	0.26 (0.55)	0.26 (0.53)	0.26 (0.51)	0.26 (0.53)	0.22 (0.47)	0.22 (0.46)	0.22 (0.48)	0.22 (0.46)
Inpatient hospitalization: mean (sd)	0.01 (0.17)	0.01 (0.14)	0.01 (0.12)	0.01 (0.14)	0.00 (0.01)	0.00 (0.01)	0.00 (0.01)	0.00 (0.01)
ED visit: mean (sd)	0.27 (0.79)	0.27 (0.85)	0.28 (0.87)	0.27 (0.85)	0.42 (1.30)	0.40 (1.27)	0.42 (1.25)	0.40 (1.27)
Number of distinct medication prescriptions: mean (sd)	4.47 (4.17)	4.73 (4.36)	4.54 (4.29)	4.73 (4.36)	4.89 (4.54)	5.03 (4.63)	4.99 (4.59)	5.03 (4.63)
Combined Comorbidity Score: mean (sd)	0.32 (1.07)	0.39 (1.23)	0.35 (1.14)	0.39 (1.23)	0.39 (1.25)	0.39 (1.24)	0.39 (1.24)	0.39 (1.24)

Table W- Characteristics of patients included in the evaluation of comparative outcomes for *glipizide* between generic or authorized generics (AG) and brand initiators before 1:1 propensity score matching in each database

	Optum				Truven			
	**Generic initiators**	**Brand initiators**	**AG initiators**	**Brand initiators**	**Generic initiators**	**Brand initiators**	**AG initiators**	**Brand initiators**
**Cohort selection steps**								
Total number of patients filling prescriptions for the version of interest after 6-month continuous enrollment	**22,864**		**5,355**		**132,724**		**59,108**	
Patients meeting new drug use restriction	**16,754**		**2,117**		**36,924**		**5,947**	
Total eligible by exposure group	**15,757**	**997**	**1,120**	**997**	**34,590**	**2,334**	**3,607**	**2,340**
**Baseline characteristics**								
Age: mean (sd)	55.16 (12.53)	58.30 (12.53)	57.72 (10.97)	58.30 (12.53)	60.13 (14.01)	61.61 (13.48)	62.29 (12.63)	61.60 (13.48)
Gender								
MALE: n (%)	8,485 (53.8%)	506 (50.8%)	636 (56.8%)	506 (50.8%)	17,741 (51.3%)	1,172 (50.2%)	1,885 (52.3%)	1,176 (50.3%)
FEMALE: n (%)	7,266 (46.1%)	491 (49.2%)	483 (43.1%)	491 (49.2%)	16,849 (48.7%)	1,162 (49.8%)	1,722 (47.7%)	1,164 (49.7%)
UNKNOWN: n (%)	6 (0.0%)	0 (0.0%)	1 (0.1%)	0 (0.0%)	0 (0%)	0 (0%)	0 (0%)	0 (0%)
Region								
Northeast: n (%)	1,795 (11.4%)	187 (18.8%)	175 (15.6%)	187 (18.8%)	2,966 (8.6%)	199 (8.5%)	363 (10.1%)	199 (8.5%)
Midwest: n (%)	5,008 (31.8%)	285 (28.6%)	328 (29.3%)	285 (28.6%)	10,023 (29.0%)	605 (25.9%)	1,409 (39.1%)	607 (25.9%)
South: n (%)	7,951 (50.5%)	462 (46.3%)	496 (44.3%)	462 (46.3%)	15,698 (45.4%)	1,052 (45.1%)	1,307 (36.2%)	1,055 (45.1%)
West: n (%)	972 (6.2%)	57 (5.7%)	120 (10.7%)	57 (5.7%)	5,647 (16.3%)	451 (19.3%)	503 (13.9%)	452 (19.3%)
Unknown/other: n (%)	31 (0.2%)	6 (0.6%)	1 (0.1%)	6 (0.6%)	256 (0.7%)	27 (1.2%)	25 (0.7%)	27 (1.2%)
Hyperlipidemia: n (%)	7,697 (48.8%)	446 (44.7%)	587 (52.4%)	446 (44.7%)	7,565 (21.9%)	473 (20.3%)	821 (22.8%)	473 (20.2%)
Diabetes mellitus: n (%)	12,099 (76.8%)	722 (72.4%)	864 (77.1%)	722 (72.4%)	22,920 (66.3%)	1,501 (64.3%)	2,599 (72.1%)	1,505 (64.3%)

Hypertension: n (%)	8,758 (55.6%)	517 (51.9%)	645 (57.6%)	517 (51.9%)	12,240 (35.4%)	771 (33.0%)	1,397 (38.7%)	774 (33.1%)
Myocardial Infarction: n (%)	240 (1.5%)	11 (1.1%)	14 (1.2%)	11 (1.1%)	551 (1.6%)	27 (1.2%)	53 (1.5%)	27 (1.2%)
Angina: n (%)	662 (4.2%)	39 (3.9%)	41 (3.7%)	39 (3.9%)	1,421 (4.1%)	84 (3.6%)	154 (4.3%)	84 (3.6%)
Heart failure: n (%)	1,063 (6.7%)	62 (6.2%)	60 (5.4%)	62 (6.2%)	2,538 (7.3%)	170 (7.3%)	255 (7.1%)	170 (7.3%)
Other forms of chronic heart diseases: n (%)	2,019 (12.8%)	146 (14.6%)	155 (13.8%)	146 (14.6%)	4,228 (12.2%)	266 (11.4%)	513 (14.2%)	267 (11.4%)
Cerebrovascular accident: n (%)	322 (2.0%)	20 (2.0%)	18 (1.6%)	20 (2.0%)	875 (2.5%)	54 (2.3%)	97 (2.7%)	54 (2.3%)
Venous thromboembolism: n (%)	253 (1.6%)	11 (1.1%)	18 (1.6%)	11 (1.1%)	598 (1.7%)	40 (1.7%)	51 (1.4%)	40 (1.7%)
Atrial fibrillation: n (%)	652 (4.1%)	54 (5.4%)	34 (3.0%)	54 (5.4%)	1,562 (4.5%)	109 (4.7%)	151 (4.2%)	109 (4.7%)
Overweight or obese: n (%)	1,126 (7.1%)	55 (5.5%)	59 (5.3%)	55 (5.5%)	552 (1.6%)	30 (1.3%)	53 (1.5%)	30 (1.3%)
Tobacco use: n (%)	589 (3.7%)	20 (2.0%)	19 (1.7%)	20 (2.0%)	480 (1.4%)	23 (1.0%)	47 (1.3%)	23 (1.0%)
Alcohol abuse or dependence: n (%)	134 (0.9%)	3 (0.3%)	5 (0.4%)	3 (0.3%)	133 (0.4%)	12 (0.5%)	21 (0.6%)	12 (0.5%)
Renal disease: n (%)	422 (2.7%)	31 (3.1%)	27 (2.4%)	31 (3.1%)	792 (2.3%)	52 (2.2%)	94 (2.6%)	52 (2.2%)
Liver disease: n (%)	600 (3.8%)	30 (3.0%)	31 (2.8%)	30 (3.0%)	647 (1.9%)	47 (2.0%)	67 (1.9%)	47 (2.0%)
Aspirin: n (%)	74 (0.5%)	1 (0.1%)	0 (0.0%)	1 (0.1%)	1 (0.0%)	0 (0.0%)	0 (0.0%)	0 (0.0%)
Antiplatelets: n (%)	583 (3.7%)	37 (3.7%)	58 (5.2%)	37 (3.7%)	2,007 (5.8%)	117 (5.0%)	258 (7.2%)	117 (5.0%)
Statins: n (%)	4,434 (28.1%)	247 (24.8%)	405 (36.2%)	247 (24.8%)	12,211 (35.3%)	735 (31.5%)	1,646 (45.6%)	736 (31.5%)
Other lipid lowering agents: n (%)	1,351 (8.6%)	69 (6.9%)	134 (12.0%)	69 (6.9%)	2,912 (8.4%)	166 (7.1%)	439 (12.2%)	167 (7.1%)
Insulin preparations: n (%)	0 (0.0%)	0 (0.0%)	0 (0.0%)	0 (0.0%)	0 (0.0%)	0 (0.0%)	0 (0.0%)	0 (0.0%)
Oral hypoglycemic agents (without glipizide): n (%)	7,394 (46.9%)	365 (36.6%)	586 (52.3%)	365 (36.6%)	17,078 (49.4%)	1,015 (43.5%)	2,126 (58.9%)	1,020 (43.6%)
ACE inhibitors: n (%)	4,797 (30.4%)	214 (21.5%)	375 (33.5%)	214 (21.5%)	11,843 (34.2%)	707 (30.3%)	1,429 (39.6%)	710 (30.3%)
ARBs: n (%)	1,947 (12.4%)	120 (12.0%)	172 (15.4%)	120 (12.0%)	5,466 (15.8%)	386 (16.5%)	684 (19.0%)	386 (16.5%)
Calcium channel blockers: n (%)	2,389 (15.2%)	123 (12.3%)	208 (18.6%)	123 (12.3%)	6,908 (20.0%)	472 (20.2%)	852 (23.6%)	474 (20.3%)
Diuretics: n (%)	4,775 (30.3%)	265 (26.6%)	342 (30.5%)	265 (26.6%)	13,086 (37.8%)	851 (36.5%)	1,486 (41.2%)	852 (36.4%)

Beta-blockers: n (%)	3,200 (20.3%)	151 (15.1%)	244 (21.8%)	151 (15.1%)	8,400 (24.3%)	515 (22.1%)	1,019 (28.3%)	516 (22.1%)
Anticoagulants: n (%)	590 (3.7%)	55 (5.5%)	35 (3.1%)	55 (5.5%)	1,911 (5.5%)	136 (5.8%)	205 (5.7%)	136 (5.8%)
NSAIDs: n (%)	1,849 (11.7%)	77 (7.7%)	125 (11.2%)	77 (7.7%)	4,352 (12.6%)	244 (10.5%)	535 (14.8%)	244 (10.4%)
Coxibs: n (%)	731 (4.6%)	59 (5.9%)	52 (4.6%)	59 (5.9%)	2,644 (7.6%)	192 (8.2%)	287 (8.0%)	192 (8.2%)
Outpatient visits: mean (sd)	0.11 (0.37)	0.09 (0.36)	0.10 (0.33)	0.09 (0.36)	0.07 (0.31)	0.06 (0.28)	0.07 (0.38)	0.06 (0.28)
Inpatient hospitalization: mean (sd)	0.02 (0.16)	0.01 (0.13)	0.01 (0.08)	0.01 (0.13)	0.00 (0.01)	0.00 (0.00)	0.00 (0.00)	0.00 (0.00)
ED visit: mean (sd)	0.36 (1.05)	0.29 (0.88)	0.19 (0.65)	0.29 (0.88)	0.32 (0.91)	0.27 (0.78)	0.25 (0.68)	0.27 (0.78)
Number of distinct medication prescriptions: mean (sd)	5.78 (4.90)	5.17 (4.98)	5.72 (4.59)	5.17 (4.98)	6.90 (5.35)	6.47 (5.47)	7.34 (5.12)	6.47 (5.48)
Combined Comorbidity Score: mean (sd)	0.37 (1.68)	0.45 (1.65)	0.24 (1.53)	0.45 (1.65)	0.47 (1.53)	0.46 (1.46)	0.40 (1.45)	0.46 (1.46)

Table X- Characteristics of patients included in the evaluation of comparative outcomes for *quinapril* between generic or authorized generics (AG) and brand initiators <u>before 1:1 propensity score matching</u> in each database

	Optum				Truven			
	Generic initiators	Brand initiators	AG initiators	Brand initiators	Generic initiators	Brand initiators	AG initiators	Brand initiators
Cohort selection steps								
Total number of patients filling prescriptions for the version of interest after 6-month continuous enrollment	17,732		18,781		138,664		155,376	
Patients meeting new drug use restriction	11,041		10,162		21,781		23,813	
Total eligible by exposure group	4,597	6,444	3,718	6,444	8,255	12,739	9,985	12,740
Baseline characteristics								
Age: mean (sd)	52.67 (11.95)	54.14 (12.67)	54.41 (11.93)	54.14 (12.67)	56.48 (14.23)	58.90 (14.21)	58.70 (13.79)	58.91 (14.21)
Gender								
MALE: n (%)	2,649 (57.6%)	3,581 (55.6%)	2,147 (57.7%)	3,581 (55.6%)	4,259 (51.6%)	6,569 (51.6%)	5,452 (54.6%)	6,569 (51.6%)
FEMALE: n (%)	1,946 (42.3%)	2,861 (44.4%)	1,570 (42.2%)	2,861 (44.4%)	3,996 (48.4%)	6,170 (48.4%)	4,533 (45.4%)	6,171 (48.4%)
UNKNOWN: n (%)	2 (0.0%)	2 (0.0%)	1 (0.0%)	2 (0.0%)	0 (0%)	0 (0%)	0 (0%)	0 (0%)
Region								
Northeast: n (%)	592 (12.9%)	987 (15.3%)	653 (17.6%)	987 (15.3%)	855 (10.4%)	1,636 (12.8%)	1,604 (16.1%)	1,636 (12.8%)
Midwest: n (%)	1,239 (27.0%)	1,877 (29.1%)	1,005 (27.0%)	1,877 (29.1%)	2,113 (25.6%)	3,734 (29.3%)	3,028 (30.3%)	3,734 (29.3%)
South: n (%)	2,377 (51.7%)	3,049 (47.3%)	1,695 (45.6%)	3,049 (47.3%)	4,106 (49.7%)	5,052 (39.7%)	4,031 (40.4%)	5,053 (39.7%)
West: n (%)	381 (8.3%)	525 (8.1%)	360 (9.7%)	525 (8.1%)	1,132 (13.7%)	2,227 (17.5%)	1,263 (12.6%)	2,227 (17.5%)
Unknown/other: n (%)	8 (0.2%)	6 (0.1%)	5 (0.1%)	6 (0.1%)	49 (0.6%)	90 (0.7%)	59 (0.6%)	90 (0.7%)
Hyperlipidemia: n (%)	1,798 (39.1%)	2,496 (38.7%)	1,535 (41.3%)	2,496 (38.7%)	1,674 (20.3%)	2,378 (18.7%)	2,306 (23.1%)	2,378 (18.7%)
Diabetes mellitus: n (%)	1,262 (27.5%)	1,679 (26.1%)	989 (26.6%)	1,679 (26.1%)	1,916 (23.2%)	2,966 (23.3%)	2,613 (26.2%)	2,966 (23.3%)

Hypertension: n (%)	2,758 (60.0%)	4,109 (63.8%)	2,276 (61.2%)	4,109 (63.8%)	3,662 (44.4%)	5,820 (45.7%)	4,573 (45.8%)	5,821 (45.7%)
Myocardial Infarction: n (%)	148 (3.2%)	201 (3.1%)	77 (2.1%)	201 (3.1%)	242 (2.9%)	352 (2.8%)	249 (2.5%)	352 (2.8%)
Angina: n (%)	229 (5.0%)	344 (5.3%)	172 (4.6%)	344 (5.3%)	327 (4.0%)	643 (5.0%)	425 (4.3%)	643 (5.0%)
Heart failure: n (%)	233 (5.1%)	423 (6.6%)	183 (4.9%)	423 (6.6%)	525 (6.4%)	890 (7.0%)	544 (5.4%)	890 (7.0%)
Other forms of chronic heart diseases: n (%)	561 (12.2%)	935 (14.5%)	462 (12.4%)	935 (14.5%)	861 (10.4%)	1,698 (13.3%)	1,194 (12.0%)	1,698 (13.3%)
Cerebrovascular accident: n (%)	108 (2.3%)	131 (2.0%)	67 (1.8%)	131 (2.0%)	214 (2.6%)	360 (2.8%)	245 (2.5%)	360 (2.8%)
Venous thromboembolism: n (%)	62 (1.3%)	85 (1.3%)	43 (1.2%)	85 (1.3%)	99 (1.2%)	144 (1.1%)	135 (1.4%)	144 (1.1%)
Atrial fibrillation: n (%)	168 (3.7%)	262 (4.1%)	141 (3.8%)	262 (4.1%)	352 (4.3%)	522 (4.1%)	373 (3.7%)	522 (4.1%)
Overweight or obese: n (%)	271 (5.9%)	334 (5.2%)	134 (3.6%)	334 (5.2%)	117 (1.4%)	157 (1.2%)	127 (1.3%)	157 (1.2%)
Tobacco use: n (%)	211 (4.6%)	269 (4.2%)	140 (3.8%)	269 (4.2%)	113 (1.4%)	177 (1.4%)	120 (1.2%)	177 (1.4%)
Alcohol abuse or dependence: n (%)	57 (1.2%)	60 (0.9%)	36 (1.0%)	60 (0.9%)	31 (0.4%)	62 (0.5%)	46 (0.5%)	62 (0.5%)
Renal disease: n (%)	104 (2.3%)	148 (2.3%)	77 (2.1%)	148 (2.3%)	147 (1.8%)	226 (1.8%)	158 (1.6%)	226 (1.8%)
Liver disease: n (%)	109 (2.4%)	132 (2.0%)	82 (2.2%)	132 (2.0%)	106 (1.3%)	171 (1.3%)	141 (1.4%)	171 (1.3%)
Aspirin: n (%)	7 (0.2%)	6 (0.1%)	1 (0.0%)	6 (0.1%)	49 (0.6%)	92 (0.7%)	62 (0.6%)	92 (0.7%)
Antiplatelets: n (%)	138 (3.0%)	216 (3.4%)	120 (3.2%)	216 (3.4%)	350 (4.2%)	603 (4.7%)	496 (5.0%)	603 (4.7%)
Statins: n (%)	909 (19.8%)	1,170 (18.2%)	841 (22.6%)	1,170 (18.2%)	1,917 (23.2%)	3,102 (24.4%)	2,925 (29.3%)	3,102 (24.3%)
Other lipid lowering agents: n (%)	304 (6.6%)	288 (4.5%)	256 (6.9%)	288 (4.5%)	559 (6.8%)	497 (3.9%)	907 (9.1%)	497 (3.9%)
Insulin preparations: n (%)	216 (4.7%)	224 (3.5%)	147 (4.0%)	224 (3.5%)	219 (2.7%)	346 (2.7%)	311 (3.1%)	346 (2.7%)
Oral hypoglycemic agents: n (%)	775 (16.9%)	987 (15.3%)	610 (16.4%)	987 (15.3%)	1,438 (17.4%)	2,302 (18.1%)	1,914 (19.2%)	2,302 (18.1%)
ACE inhibitors: n (%)	602 (13.1%)	660 (10.2%)	488 (13.1%)	660 (10.2%)	1,406 (17.0%)	2,040 (16.0%)	2,001 (20.0%)	2,040 (16.0%)
ARBs: n (%)	288 (6.3%)	321 (5.0%)	236 (6.3%)	321 (5.0%)	565 (6.8%)	842 (6.6%)	759 (7.6%)	843 (6.6%)
Calcium channel blockers (without quinapril): n (%)	643 (14.0%)	816 (12.7%)	514 (13.8%)	816 (12.7%)	682 (8.3%)	1,215 (9.5%)	826 (8.3%)	1,215 (9.5%)
Diuretics: n (%)	1,088 (23.7%)	1,565 (24.3%)	859 (23.1%)	1,565 (24.3%)	2,314 (28.0%)	3,804 (29.9%)	2,935 (29.4%)	3,805 (29.9%)
Beta-blockers: n (%)	823 (17.9%)	1,145 (17.8%)	628 (16.9%)	1,145 (17.8%)	1,673 (20.3%)	2,820 (22.1%)	2,235 (22.4%)	2,821 (22.1%)
Anticoagulants: n (%)	134 (2.9%)	158 (2.5%)	112 (3.0%)	158 (2.5%)	306 (3.7%)	537 (4.2%)	391 (3.9%)	537 (4.2%)

NSAIDs: n (%)	556 (12.1%)	587 (9.1%)	417 (11.2%)	587 (9.1%)	1,083 (13.1%)	1,345 (10.6%)	1,233 (12.3%)	1,345 (10.6%)
Coxibs: n (%)	102 (2.2%)	387 (6.0%)	87 (2.3%)	387 (6.0%)	283 (3.4%)	1,257 (9.9%)	319 (3.2%)	1,257 (9.9%)
Outpatient visits: mean (sd)	0.14 (0.41)	0.13 (0.45)	0.13 (0.38)	0.13 (0.45)	0.10 (0.33)	0.09 (0.33)	0.10 (0.35)	0.09 (0.33)
Inpatient hospitalization: mean (sd)	0.01 (0.12)	0.01 (0.12)	0.01 (0.10)	0.01 (0.12)	0.00 (0.00)	0.00 (0.03)	0.00 (0.00)	0.00 (0.03)
ED visit: mean (sd)	0.27 (0.92)	0.29 (0.98)	0.23 (0.75)	0.29 (0.98)	0.30 (0.90)	0.27 (0.79)	0.26 (0.79)	0.27 (0.79)
Number of distinct medication prescriptions: mean (sd)	4.26 (4.29)	4.02 (4.18)	3.99 (3.98)	4.02 (4.18)	5.03 (4.95)	5.21 (4.88)	5.12 (4.77)	5.21 (4.88)
Combined Comorbidity Score: mean (sd)	0.11 (1.43)	0.11 (1.49)	0.03 (1.32)	0.11 (1.49)	0.19 (1.36)	0.23 (1.39)	0.14 (1.30)	0.23 (1.39)

Table Y- Characteristics of patients included in the evaluation of comparative outcomes for *sertraline* between generic or authorized generics (AG) and brand initiators before 1:1 propensity score matching in each database

	Optum				Truven			
	**Generic initiators**	**Brand initiators**	**AG initiators**	**Brand initiators**	**Generic initiators**	**Brand initiators**	**AG initiators**	**Brand initiators**
**Cohort selection steps**								
Total number of patients filling prescriptions for the version of interest after 6-month continuous enrollment	263,912		259,228		821,509		796,518	
Patients meeting new drug use restriction	190,914		192,092		387,055		386,146	
Total eligible by exposure group	54,572	136,342	55,750	136,342	133,010	254,045	132,099	254,047
**Baseline characteristics**								
Age: mean (sd)	39.52 (15.33)	39.59 (15.40)	38.51 (14.91)	39.59 (15.40)	43.82 (17.80)	45.76 (18.78)	42.44 (17.30)	45.76 (18.78)
Gender								
MALE: n (%)	17,137 (31.4%)	42,750 (31.4%)	17,491 (31.4%)	42,750 (31.4%)	41,634 (31.3%)	79,671 (31.4%)	41,603 (31.5%)	79,672 (31.4%)
FEMALE: n (%)	37,428 (68.6%)	93,562 (68.6%)	38,254 (68.6%)	93,562 (68.6%)	91,376 (68.7%)	174,374 (68.6%)	90,496 (68.5%)	174,375 (68.6%)
UNKNOWN: n (%)	7 (0.0%)	30 (0.0%)	5 (0.0%)	30 (0.0%)	0 (0%)	0 (0%)	0 (0%)	0 (0%)
Region								
Northeast: n (%)	5,804 (10.6%)	12,804 (9.4%)	3,475 (6.2%)	12,804 (9.4%)	**14,516 (10.9%)**	**22,246 (8.8%)**	**9,339 (7.1%)**	**22,246 (8.8%)**
Midwest: n (%)	15,325 (28.1%)	43,661 (32.0%)	18,068 (32.4%)	43,661 (32.0%)	38,527 (29.0%)	71,550 (28.2%)	44,581 (33.7%)	71,551 (28.2%)
South: n (%)	27,883 (51.1%)	63,069 (46.3%)	25,598 (45.9%)	63,069 (46.3%)	65,819 (49.5%)	115,978 (45.7%)	55,219 (41.8%)	115,979 (45.7%)
West: n (%)	5,531 (10.1%)	16,718 (12.3%)	8,587 (15.4%)	16,718 (12.3%)	13,581 (10.2%)	42,179 (16.6%)	22,395 (17.0%)	42,179 (16.6%)
Unknown/other: n (%)	29 (0.1%)	90 (0.1%)	22 (0.0%)	90 (0.1%)	567 (0.4%)	2,092 (0.8%)	565 (0.4%)	2,092 (0.8%)
Epilepsy: n (%)	645 (1.2%)	1,919 (1.4%)	723 (1.3%)	1,919 (1.4%)	1,541 (1.2%)	3,525 (1.4%)	1,466 (1.1%)	3,526 (1.4%)
Depression: n (%)	4,991 (9.1%)	15,844 (11.6%)	5,279 (9.5%)	15,844 (11.6%)	9,315 (7.0%)	19,628 (7.7%)	9,925 (7.5%)	19,628 (7.7%)
Anxiety: n (%)	4,031 (7.4%)	11,816 (8.7%)	4,331 (7.8%)	11,816 (8.7%)	6,105 (4.6%)	12,171 (4.8%)	6,667 (5.0%)	12,171 (4.8%)
Alcohol abuse or dependence: n (%)	942 (1.7%)	2,533 (1.9%)	1,122 (2.0%)	2,533 (1.9%)	1,432 (1.1%)	2,856 (1.1%)	1,614 (1.2%)	2,856 (1.1%)
Drug abuse or dependence: n (%)	897 (1.6%)	2,404 (1.8%)	1,034 (1.9%)	2,404 (1.8%)	1,267 (1.0%)	2,509 (1.0%)	1,573 (1.2%)	2,509 (1.0%)

Sleep disorder: n (%)	4,645 (8.5%)	12,564 (9.2%)	4,814 (8.6%)	12,564 (9.2%)	6,877 (5.2%)	14,134 (5.6%)	6,897 (5.2%)	14,134 (5.6%)
Psychotic disorder: n (%)	614 (1.1%)	1,848 (1.4%)	702 (1.3%)	1,848 (1.4%)	1,357 (1.0%)	3,393 (1.3%)	1,533 (1.2%)	3,393 (1.3%)
Personality disorder: n (%)	316 (0.6%)	1,052 (0.8%)	350 (0.6%)	1,052 (0.8%)	388 (0.3%)	811 (0.3%)	414 (0.3%)	811 (0.3%)
Adjustment reaction/post-traumatic stress disorder: n (%)	2,779 (5.1%)	7,956 (5.8%)	3,074 (5.5%)	7,956 (5.8%)	4,961 (3.7%)	9,732 (3.8%)	5,412 (4.1%)	9,732 (3.8%)
ADHD: n (%)	2,012 (3.7%)	5,175 (3.8%)	2,197 (3.9%)	5,175 (3.8%)	3,045 (2.3%)	5,416 (2.1%)	3,416 (2.6%)	5,416 (2.1%)
Delirium: n (%)	498 (0.9%)	1,400 (1.0%)	545 (1.0%)	1,400 (1.0%)	1,171 (0.9%)	2,600 (1.0%)	1,174 (0.9%)	2,599 (1.0%)
Bipolar disorder: n (%)	121 (0.2%)	308 (0.2%)	116 (0.2%)	308 (0.2%)	210 (0.2%)	427 (0.2%)	218 (0.2%)	427 (0.2%)
Other psychiatric disorder: n (%)	2,747 (5.0%)	8,549 (6.3%)	2,904 (5.2%)	8,549 (6.3%)	3,772 (2.8%)	8,484 (3.3%)	4,204 (3.2%)	8,484 (3.3%)
Psychiatric hospitalization: mean (sd)	0.03 (0.22)	0.03 (0.23)	0.03 (0.22)	0.03 (0.23)	0.02 (0.17)	0.03 (0.20)	0.02 (0.18)	0.03 (0.20)
Psychiatric office visit: mean (sd)	1.71 (5.51)	2.33 (7.01)	1.87 (6.19)	2.33 (7.01)	1.45 (4.87)	1.66 (4.89)	1.64 (5.11)	1.66 (4.89)
Anticonvulsants/antiepileptic drugs: n (%)	3,139 (5.8%)	7,588 (5.6%)	3,314 (5.9%)	7,588 (5.6%)	8,249 (6.2%)	17,475 (6.9%)	8,254 (6.2%)	17,475 (6.9%)
SSRIs (without sertraline): n (%)	8,870 (16.3%)	20,737 (15.2%)	9,237 (16.6%)	20,737 (15.2%)	19,994 (15.0%)	39,968 (15.7%)	19,796 (15.0%)	39,968 (15.7%)
SNRIs: n (%)	2,733 (5.0%)	5,818 (4.3%)	2,888 (5.2%)	5,818 (4.3%)	6,575 (4.9%)	11,260 (4.4%)	6,497 (4.9%)	11,260 (4.4%)
Tricyclic antidepressants: n (%)	1,281 (2.3%)	4,064 (3.0%)	1,387 (2.5%)	4,064 (3.0%)	3,416 (2.6%)	9,037 (3.6%)	3,426 (2.6%)	9,037 (3.6%)
Antipsychotics: n (%)	1,995 (3.7%)	5,820 (4.3%)	2,248 (4.0%)	5,820 (4.3%)	5,128 (3.9%)	12,506 (4.9%)	5,257 (4.0%)	12,506 (4.9%)
Benzodiazepines: n (%)	10,344 (19.0%)	29,025 (21.3%)	11,296 (20.3%)	29,025 (21.3%)	25,616 (19.3%)	59,212 (23.3%)	26,292 (19.9%)	59,212 (23.3%)
Non-BZD sedative hypnotics: n (%)	5,660 (10.4%)	14,543 (10.7%)	6,251 (11.2%)	14,543 (10.7%)	14,573 (11.0%)	31,816 (12.5%)	14,913 (11.3%)	31,816 (12.5%)
Outpatient visits: mean (sd)	0.21 (0.45)	0.23 (0.51)	0.20 (0.44)	0.23 (0.51)	0.17 (0.42)	0.16 (0.40)	0.17 (0.42)	0.16 (0.40)
Inpatient hospitalization: mean (sd)	0.01 (0.11)	0.01 (0.14)	0.01 (0.11)	0.01 (0.14)	0.00 (0.01)	0.00 (0.02)	0.00 (0.01)	0.00 (0.02)
ED visit: mean (sd)	0.27 (0.88)	0.31 (1.05)	0.30 (1.28)	0.31 (1.05)	0.35 (1.10)	0.34 (1.06)	0.36 (1.14)	0.34 (1.06)
Number of distinct medication prescriptions: mean (sd)	4.34 (4.18)	4.54 (4.35)	4.46 (4.28)	4.54 (4.35)	4.86 (4.75)	5.47 (5.05)	4.68 (4.70)	5.47 (5.05)
Combined Comorbidity Score: mean (sd)	0.29 (1.08)	0.36 (1.16)	0.32 (1.10)	0.36 (1.16)	0.30 (1.12)	0.37 (1.20)	0.32 (1.09)	0.37 (1.20)

Table Z- Characteristics of patients included in the evaluation of comparative outcomes for *alendronate* between generic or authorized generics (AG) and brand initiators after 1:1 propensity score matching in each database

	Optum				Truven			
	**Generic initiators**	**Brand initiators**	**AG initiators**	**Brand initiators**	**Generic initiators**	**Brand initiators**	**AG initiators**	**Brand initiators**
**Cohort selection steps**								
Total number of patients filling prescriptions for the version of interest after 6-month continuous enrollment	**97,191**		**59,484**		**659,262**		**627,150**	
Patients meeting new drug use restriction	**59,402**		**29,543**		**285,497**		**302,521**	
Total eligible by exposure group	**32,289**	**27,113**	**2,430**	**27,113**	**169,015**	**113,572**	**11,959**	**287,545**
**1:1 PS matched**	**26,421**	**26,421**	**2,428**	**2,428**	**112,820**	**112,820**	**11,958**	**11,958**
**Baseline characteristics**								
Age: mean (sd)	59 (9.58)	59 (9.89)	60 (10.12)	60 (10.05)	64.01 (11.67)	64.25 (11.83)	63.48 (11.93)	63.22 (11.89)
Gender								
MALE: n (%)	2,525 (9.6%)	2,535 (9.6%)	217 (8.9%)	223 (9.2%)	12,369 (11.0%)	12,545 (11.1%)	1,234 (10.3%)	1,253 (10.5%)
FEMALE: n (%)	23,894 (90.4%)	23,884 (90.4%)	2,210 (91.0%)	2,205 (90.8%)	100,451 (89.0%)	100,275 (88.9%)	10,724 (89.7%)	10,705 (89.5%)
UNKNOWN: n (%)	2 (0.0%)	2 (0.0%)	1 (0.0%)	0 (0.0%)	0 (0%)	0 (0%)	0 (0%)	0 (0%)
Region								
Northeast: n (%)	2,291 (8.7%)	2,337 (8.8%)	422 (17.4%)	443 (18.2%)	11,153 (9.9%)	11,335 (10.0%)	2,314 (19.4%)	2,296 (19.2%)
Midwest: n (%)	7,456 (28.2%)	7,436 (28.1%)	564 (23.2%)	562 (23.1%)	38,965 (34.5%)	39,087 (34.6%)	4,016 (33.6%)	4,101 (34.3%)
South: n (%)	12,334 (46.7%)	12,287 (46.5%)	1,227 (50.5%)	1,193 (49.1%)	41,094 (36.4%)	40,656 (36.0%)	3,808 (31.8%)	3,774 (31.6%)
West: n (%)	4,325 (16.4%)	4,345 (16.4%)	215 (8.9%)	230 (9.5%)	21,193 (18.8%)	21,300 (18.9%)	1,696 (14.2%)	1,673 (14.0%)
Unknown/other: n (%)	15 (0.1%)	16 (0.1%)	0 (0.0%)	0 (0.0%)	415 (0.4%)	442 (0.4%)	124 (1.0%)	114 (1.0%)
Osteoporosis: n (%)	10,342 (39.1%)	10,229 (38.7%)	972 (40.0%)	951 (39.2%)	30,753 (27.3%)	30,929 (27.4%)	3,465 (29.0%)	3,417 (28.6%)
Kyphosis: n (%)	352 (1.3%)	361 (1.4%)	24 (1.0%)	33 (1.4%)	795 (0.7%)	829 (0.7%)	99 (0.8%)	86 (0.7%)
Vertebral facture: n (%)	598 (2.3%)	603 (2.3%)	48 (2.0%)	47 (1.9%)	2,841 (2.5%)	2,897 (2.6%)	336 (2.8%)	314 (2.6%)
Humerus fracture: n (%)	119 (0.5%)	121 (0.5%)	11 (0.5%)	7 (0.3%)	539 (0.5%)	583 (0.5%)	69 (0.6%)	67 (0.6%)
Wrist fracture: n (%)	261 (1.0%)	270 (1.0%)	19 (0.8%)	13 (0.5%)	1,026 (0.9%)	1,070 (0.9%)	134 (1.1%)	141 (1.2%)

Hip fracture: n (%)	252 (1.0%)	246 (0.9%)	19 (0.8%)	21 (0.9%)	1,378 (1.2%)	1,427 (1.3%)	163 (1.4%)	148 (1.2%)
Pelvis fracture: n (%)	117 (0.4%)	115 (0.4%)	9 (0.4%)	8 (0.3%)	531 (0.5%)	542 (0.5%)	68 (0.6%)	76 (0.6%)
Any other fractures: n (%)	1,234 (4.7%)	1,242 (4.7%)	109 (4.5%)	99 (4.1%)	4,540 (4.0%)	4,714 (4.2%)	541 (4.5%)	527 (4.4%)
Alzheimer disease or other dementia: n (%)	365 (1.4%)	368 (1.4%)	48 (2.0%)	46 (1.9%)	1,660 (1.5%)	1,760 (1.6%)	239 (2.0%)	216 (1.8%)
Asthma or chronic obstructive pulmonary disease: n (%)	2,228 (8.4%)	2,263 (8.6%)	229 (9.4%)	224 (9.2%)	7,187 (6.4%)	7,412 (6.6%)	823 (6.9%)	818 (6.8%)
Cataracts: n (%)	1,990 (7.5%)	1,988 (7.5%)	210 (8.6%)	194 (8.0%)	7,961 (7.1%)	8,222 (7.3%)	892 (7.5%)	858 (7.2%)
Crohn disease or gastroenteritis: n (%)	633 (2.4%)	642 (2.4%)	55 (2.3%)	56 (2.3%)	2,001 (1.8%)	2,103 (1.9%)	238 (2.0%)	205 (1.7%)
Depression: n (%)	733 (2.8%)	733 (2.8%)	65 (2.7%)	54 (2.2%)	1,547 (1.4%)	1,607 (1.4%)	197 (1.6%)	182 (1.5%)
Diabetes mellitus: n (%)	2,488 (9.4%)	2,541 (9.6%)	253 (10.4%)	274 (11.3%)	11,333 (10.0%)	11,642 (10.3%)	1,322 (11.1%)	1,342 (11.2%)
History of falls, syncope, or gait abnormality: n (%)	1,865 (7.1%)	1,869 (7.1%)	171 (7.0%)	183 (7.5%)	5,085 (4.5%)	5,290 (4.7%)	647 (5.4%)	613 (5.1%)
Hyperthyroidism: n (%)	337 (1.3%)	327 (1.2%)	29 (1.2%)	28 (1.2%)	959 (0.9%)	988 (0.9%)	121 (1.0%)	107 (0.9%)
Hyperparathyroidism: n (%)	168 (0.6%)	162 (0.6%)	23 (0.9%)	30 (1.2%)	584 (0.5%)	592 (0.5%)	65 (0.5%)	51 (0.4%)
Ischemic stroke: n (%)	175 (0.7%)	172 (0.7%)	18 (0.7%)	10 (0.4%)	784 (0.7%)	796 (0.7%)	106 (0.9%)	111 (0.9%)
Liver disease: n (%)	686 (2.6%)	683 (2.6%)	63 (2.6%)	64 (2.6%)	1,585 (1.4%)	1,623 (1.4%)	226 (1.9%)	233 (1.9%)
Malignant neoplasm: n (%)	2,542 (9.6%)	2,564 (9.7%)	212 (8.7%)	191 (7.9%)	10,311 (9.1%)	10,511 (9.3%)	1,107 (9.3%)	1,083 (9.1%)
Overweight or obese: n (%)	546 (2.1%)	550 (2.1%)	54 (2.2%)	49 (2.0%)	687 (0.6%)	676 (0.6%)	152 (1.3%)	130 (1.1%)
Parkinson disease: n (%)	91 (0.3%)	91 (0.3%)	11 (0.5%)	9 (0.4%)	528 (0.5%)	557 (0.5%)	57 (0.5%)	40 (0.3%)
Renal disease: n (%)	231 (0.9%)	238 (0.9%)	24 (1.0%)	23 (0.9%)	665 (0.6%)	693 (0.6%)	94 (0.8%)	106 (0.9%)
Rheumatoid arthritis: n (%)	989 (3.7%)	1,009 (3.8%)	119 (4.9%)	134 (5.5%)	3,701 (3.3%)	3,796 (3.4%)	458 (3.8%)	472 (3.9%)
Oral glucocorticoids: n (%)	5,210 (19.7%)	5,209 (19.7%)	499 (20.6%)	477 (19.6%)	23,572 (20.9%)	24,226 (21.5%)	2,629 (22.0%)	2,568 (21.5%)
Anticonvulsants/antiepileptic drugs: n (%)	1,707 (6.5%)	1,723 (6.5%)	189 (7.8%)	208 (8.6%)	8,236 (7.3%)	8,422 (7.5%)	996 (8.3%)	921 (7.7%)
Benzodiazepines: n (%)	3,451 (13.1%)	3,471 (13.1%)	401 (16.5%)	388 (16.0%)	15,352 (13.6%)	15,737 (13.9%)	1,736 (14.5%)	1,586 (13.3%)
SSRIs: n (%)	4,151 (15.7%)	4,162 (15.8%)	390 (16.1%)	350 (14.4%)	17,901 (15.9%)	18,259 (16.2%)	1,980 (16.6%)	1,942 (16.2%)
Beta-blockers: n (%)	3,634 (13.8%)	3,666 (13.9%)	341 (14.0%)	326 (13.4%)	22,356 (19.8%)	22,791 (20.2%)	2,491 (20.8%)	2,392 (20.0%)
Proton pump inhibitors: n (%)	3,658 (13.8%)	3,676 (13.9%)	328 (13.5%)	318 (13.1%)	23,001 (20.4%)	23,434 (20.8%)	2,264 (18.9%)	2,154 (18.0%)
Opioids: n (%)	7,012 (26.5%)	7,009 (26.5%)	683 (28.1%)	677 (27.9%)	32,033 (28.4%)	32,763 (29.0%)	3,684 (30.8%)	3,528 (29.5%)
Other anti-osteoporosis medications (without alendronate): n (%)	2,522 (9.5%)	2,614 (9.9%)	292 (12.0%)	302 (12.4%)	23,988 (21.3%)	24,094 (21.4%)	2,457 (20.5%)	2,502 (20.9%)

Outpatient visits (occurrence): mean (sd)	0.40 (0.55)	0.40 (0.57)	0.36 (0.53)	0.36 (0.54)	0.24 (0.47)	0.24 (0.48)	0.27 (0.51)	0.25 (0.57)
Inpatient hospitalization (occurrence): mean (sd)	0.01 (0.15)	0.01 (0.14)	0.01 (0.09)	0.01 (0.09)	0.00 (0.01)	0.00 (0.01)	0.00 (0.01)	0.00 (0.01)
ED visit (occurrence): mean (sd)	0.16 (0.65)	0.17 (0.63)	0.16 (0.57)	0.15 (0.58)	0.20 (0.72)	0.20 (0.71)	0.24 (0.76)	0.22 (0.85)
Number of distinct medication prescriptions: mean (sd)	6.28 (4.42)	6.30 (4.67)	6.68 (4.72)	6.62 (5.03)	7.23 (4.88)	7.35 (5.03)	7.32 (4.90)	7.17 (5.00)
Combined Comorbidity Score: mean (sd)	0.28 (1.25)	0.29 (1.24)	0.31 (1.29)	0.31 (1.25)	0.23 (1.15)	0.24 (1.10)	0.27 (1.17)	0.26 (1.13)

Table AA- Characteristics of patients included in the evaluation of comparative outcomes for a*mlodipine* between generic or authorized generics (AG) and brand initiators after 1:1 propensity score matching in each database

	Optum				Truven			
	**Generic initiators**	**Brand initiators**	**AG initiators**	**Brand initiators**	**Generic initiators**	**Brand initiators**	**AG initiators**	**Brand initiators**
**Cohort selection steps**								
Total number of patients filling prescriptions for the version of interest after 6-month continuous enrollment	228,673		166,273		**2,464,127**		**1,369,663**	
Patients meeting new drug use restriction	162,366		100,190		**1,312,094**		**515,057**	
Total eligible by exposure group	87,454	74,912	25,278	74,912	**1,234,533**	**32,740**	**417,590**	**32,740**
**1:1 PS matched**	69,478	69,478	25,259	25,259	**32,740**	**32,740**	**32,740**	**32,740**
**Baseline characteristics**								
Age: mean (sd)	54 (12.50)	54 (12.83)	56 (12.64)	56 (12.33)	60.60 (14.85)	60.64 (14.85)	60.37 (14.81)	60.64 (14.85)
Gender								
MALE: n (%)	36,359 (52.3%)	36,178 (52.1%)	13,732 (54.4%)	13,754 (54.5%)	14,510 (44.3%)	14,896 (45.5%)	14,816 (45.3%)	14,896 (45.5%)
FEMALE: n (%)	33,113 (47.7%)	33,296 (47.9%)	11,523 (45.6%)	11,503 (45.5%)	18,230 (55.7%)	17,844 (54.5%)	17,924 (54.7%)	17,844 (54.5%)
UNKNOWN: n (%)	6 (0.0%)	4 (0.0%)	4 (0.0%)	2 (0.0%)	0 (0%)	0 (0%)	0 (0%)	0 (0%)
Region								
Northeast: n (%)	7,189 (10.3%)	7,094 (10.2%)	1,958 (7.8%)	1,859 (7.4%)	4,253 (13.0%)	4,230 (12.9%)	4,296 (13.1%)	4,228 (12.9%)
Midwest: n (%)	19,193 (27.6%)	19,227 (27.7%)	4,388 (17.4%)	4,399 (17.4%)	8,836 (27.0%)	9,076 (27.7%)	8,883 (27.1%)	9,076 (27.7%)
South: n (%)	35,073 (50.5%)	35,184 (50.6%)	15,008 (59.4%)	15,055 (59.6%)	13,854 (42.3%)	13,780 (42.1%)	13,827 (42.2%)	13,780 (42.1%)
West: n (%)	7,986 (11.5%)	7,933 (11.4%)	3,902 (15.4%)	3,941 (15.6%)	5,548 (16.9%)	5,410 (16.5%)	5,451 (16.6%)	5,412 (16.5%)
Unknown/other: n (%)	37 (0.1%)	40 (0.1%)	3 (0.0%)	5 (0.0%)	249 (0.8%)	244 (0.7%)	283 (0.9%)	244 (0.7%)
Hyperlipidemia: n (%)	27,769 (40.0%)	28,206 (40.6%)	11,839 (46.9%)	11,792 (46.7%)	7,722 (23.6%)	7,592 (23.2%)	7,499 (22.9%)	7,592 (23.2%)

Diabetes mellitus: n (%)	13,073 (18.8%)	13,408 (19.3%)	5,317 (21.0%)	5,235 (20.7%)	5,632 (17.2%)	5,936 (18.1%)	5,705 (17.4%)	5,935 (18.1%)
Hypertension: n (%)	51,437 (74.0%)	51,891 (74.7%)	19,303 (76.4%)	19,223 (76.1%)	19,678 (60.1%)	19,530 (59.7%)	19,477 (59.5%)	19,530 (59.7%)
Myocardial Infarction: n (%)	1,271 (1.8%)	1,266 (1.8%)	397 (1.6%)	365 (1.4%)	630 (1.9%)	637 (1.9%)	608 (1.9%)	637 (1.9%)
Angina: n (%)	3,479 (5.0%)	3,609 (5.2%)	1,066 (4.2%)	1,063 (4.2%)	1,496 (4.6%)	1,613 (4.9%)	1,493 (4.6%)	1,612 (4.9%)
Heart failure: n (%)	3,282 (4.7%)	3,377 (4.9%)	1,076 (4.3%)	1,083 (4.3%)	1,686 (5.1%)	1,835 (5.6%)	1,765 (5.4%)	1,835 (5.6%)
Other forms of chronic heart diseases: n (%)	8,611 (12.4%)	8,735 (12.6%)	3,154 (12.5%)	3,081 (12.2%)	4,126 (12.6%)	4,304 (13.1%)	4,062 (12.4%)	4,301 (13.1%)
Cerebrovascular accident: n (%)	2,427 (3.5%)	2,524 (3.6%)	770 (3.0%)	781 (3.1%)	1,347 (4.1%)	1,429 (4.4%)	1,385 (4.2%)	1,429 (4.4%)
Venous thromboembolism: n (%)	1,163 (1.7%)	1,202 (1.7%)	409 (1.6%)	393 (1.6%)	589 (1.8%)	623 (1.9%)	595 (1.8%)	623 (1.9%)
Atrial fibrillation: n (%)	2,418 (3.5%)	2,438 (3.5%)	896 (3.5%)	901 (3.6%)	1,517 (4.6%)	1,600 (4.9%)	1,523 (4.7%)	1,601 (4.9%)
Overweight or obese: n (%)	4,312 (6.2%)	4,301 (6.2%)	1,525 (6.0%)	1,507 (6.0%)	669 (2.0%)	678 (2.1%)	673 (2.1%)	679 (2.1%)
Tobacco use: n (%)	3,948 (5.7%)	3,961 (5.7%)	1,300 (5.1%)	1,349 (5.3%)	574 (1.8%)	613 (1.9%)	608 (1.9%)	613 (1.9%)
Alcohol abuse or dependence: n (%)	942 (1.4%)	952 (1.4%)	271 (1.1%)	266 (1.1%)	207 (0.6%)	212 (0.6%)	190 (0.6%)	212 (0.6%)
Renal disease: n (%)	2,662 (3.8%)	2,723 (3.9%)	901 (3.6%)	871 (3.4%)	879 (2.7%)	930 (2.8%)	903 (2.8%)	931 (2.8%)
Liver disease: n (%)	2,118 (3.0%)	2,143 (3.1%)	747 (3.0%)	742 (2.9%)	590 (1.8%)	639 (2.0%)	573 (1.8%)	639 (2.0%)
Aspirin: n (%)	62 (0.1%)	75 (0.1%)	15 (0.1%)	21 (0.1%)	248 (0.8%)	259 (0.8%)	228 (0.7%)	259 (0.8%)
Antiplatelets: n (%)	3,168 (4.6%)	3,240 (4.7%)	1,228 (4.9%)	1,245 (4.9%)	1,928 (5.9%)	2,070 (6.3%)	2,014 (6.2%)	2,070 (6.3%)
Statins: n (%)	17,578 (25.3%)	17,908 (25.8%)	8,572 (33.9%)	8,565 (33.9%)	9,386 (28.7%)	9,797 (29.9%)	9,291 (28.4%)	9,797 (29.9%)
Other lipid lowering agents: n (%)	5,683 (8.2%)	5,774 (8.3%)	2,606 (10.3%)	2,611 (10.3%)	3,108 (9.5%)	3,213 (9.8%)	3,043 (9.3%)	3,214 (9.8%)
Insulin preparations: n (%)	2,252 (3.2%)	2,324 (3.3%)	934 (3.7%)	907 (3.6%)	736 (2.2%)	796 (2.4%)	817 (2.5%)	796 (2.4%)
Oral hypoglycemic agents: n (%)	8,203 (11.8%)	8,387 (12.1%)	3,418 (13.5%)	3,396 (13.4%)	4,038 (12.3%)	4,298 (13.1%)	4,047 (12.4%)	4,298 (13.1%)
ACE inhibitors: n (%)	22,674 (32.6%)	22,892 (32.9%)	8,895 (35.2%)	8,963 (35.5%)	9,629 (29.4%)	10,013 (30.6%)	9,580 (29.3%)	10,012 (30.6%)
ARBs: n (%)	14,024 (20.2%)	14,150 (20.4%)	5,792 (22.9%)	5,843 (23.1%)	7,254 (22.2%)	7,629 (23.3%)	7,281 (22.2%)	7,628 (23.3%)
Calcium channel blockers (without amlodipine): n (%)	4,672 (6.7%)	4,741 (6.8%)	1,980 (7.8%)	1,943 (7.7%)	2,651 (8.1%)	2,709 (8.3%)	2,582 (7.9%)	2,709 (8.3%)
Diuretics: n (%)	27,117 (39.0%)	27,345 (39.4%)	9,748 (38.6%)	9,728 (38.5%)	12,464 (38.1%)	12,862 (39.3%)	12,373 (37.8%)	12,863 (39.3%)

Beta-blockers: n (%)	18,790 (27.0%)	19,007 (27.4%)	6,353 (25.2%)	6,341 (25.1%)	9,488 (29.0%)	9,919 (30.3%)	9,439 (28.8%)	9,921 (30.3%)
Anticoagulants: n (%)	2,095 (3.0%)	2,127 (3.1%)	748 (3.0%)	725 (2.9%)	1,428 (4.4%)	1,493 (4.6%)	1,434 (4.4%)	1,494 (4.6%)
NSAIDs: n (%)	9,477 (13.6%)	9,565 (13.8%)	3,394 (13.4%)	3,393 (13.4%)	3,966 (12.1%)	4,167 (12.7%)	3,877 (11.8%)	4,169 (12.7%)
Coxibs: n (%)	1,480 (2.1%)	1,494 (2.2%)	595 (2.4%)	574 (2.3%)	846 (2.6%)	924 (2.8%)	888 (2.7%)	922 (2.8%)
Outpatient visits: mean (sd)	0.14 (0.38)	0.14 (0.40)	0.13 (0.39)	0.13 (0.39)	0.10 (0.32)	0.10 (0.33)	0.10 (0.32)	0.10 (0.33)
Inpatient hospitalization: mean (sd)	0.01 (0.16)	0.01 (0.16)	0.01 (0.16)	0.01 (0.17)	0.00 (0.01)	0.00 (0.01)	0.00 (0.01)	0.00 (0.01)
ED visit: mean (sd)	0.35 (0.95)	0.36 (0.95)	0.30 (0.86)	0.30 (0.77)	0.38 (1.00)	0.40 (1.01)	0.37 (1.05)	0.40 (1.01)
Number of distinct medication prescriptions: mean (sd)	5.32 (4.75)	5.38 (4.70)	5.54 (4.70)	5.55 (4.64)	5.87 (5.06)	6.12 (5.30)	5.84 (5.09)	6.12 (5.30)
Combined Comorbidity Score: mean (sd)	0.21 (1.72)	0.21 (1.71)	0.16 (1.71)	0.16 (1.65)	0.25 (1.60)	0.31 (1.62)	0.27 (1.64)	0.31 (1.62)

Table AB- Characteristics of patients included in the evaluation of comparative outcomes for *amlodipine-benazepril* between generic or authorized generics (AG) and brand initiators after 1:1 propensity score matching in each database

	Optum				Truven			
	**Generic initiators**	**Brand initiators**	**AG initiators**	**Brand initiators**	**Generic initiators**	**Brand initiators**	**AG initiators**	**Brand initiators**
**Cohort selection steps**								
Total number of patients filling prescriptions for the version of interest after 6-month continuous enrollment	78,491		69,255		**445,257**		**388,039**	
Patients meeting new drug use restriction	53,638		44,919		**166,986**		**136,170**	
Total eligible by exposure group	14,718	38,920	5,999	38,920	**53,891**	**109,164**	**23,175**	**109,167**
**1:1 PS matched**	14,704	14,704	5,992	5,992	**53,495**	**53,495**	**23,158**	**23,158**
**Baseline characteristics**								
Age: mean (sd)	52 (11.65)	52 (11.14)	54 (11.75)	53 (11.41)	54.65 (12.64)	54.65 (12.82)	54.88 (12.35)	54.65 (12.59)
Gender								
MALE: n (%)	8,670 (59.0%)	8,758 (59.6%)	3,408 (56.9%)	3,491 (58.3%)	29,835 (55.8%)	30,014 (56.1%)	12,988 (56.1%)	13,250 (57.2%)
FEMALE: n (%)	6,032 (41.0%)	5,944 (40.4%)	2,584 (43.1%)	2,501 (41.7%)	23,660 (44.2%)	23,481 (43.9%)	10,170 (43.9%)	9,908 (42.8%)
UNKNOWN: n (%)	0 (0.0%)	0 (0.0%)	0 (0.0%)	0 (0.0%)	0 (0.0%)	0 (0.0%)	0 (0.0%)	0 (0.0%)
Region								
Northeast: n (%)	927 (6.3%)	946 (6.4%)	258 (4.3%)	271 (4.5%)	5,393 (10.1%)	5,311 (9.9%)	2,715 (11.7%)	2,615 (11.3%)
Midwest: n (%)	2,457 (16.7%)	2,524 (17.2%)	911 (15.2%)	936 (15.6%)	14,532 (27.2%)	14,378 (26.9%)	6,771 (29.2%)	6,839 (29.5%)
South: n (%)	10,012 (68.1%)	9,992 (68.0%)	4,466 (74.5%)	4,397 (73.4%)	27,844 (52.0%)	28,027 (52.4%)	11,627 (50.2%)	11,678 (50.4%)
West: n (%)	1,305 (8.9%)	1,242 (8.4%)	351 (5.9%)	383 (6.4%)	5,241 (9.8%)	5,316 (9.9%)	1,722 (7.4%)	1,711 (7.4%)
Unknown/other: n (%)	3 (0.0%)	0 (0.0%)	6 (0.1%)	5 (0.1%)	485 (0.9%)	463 (0.9%)	323 (1.4%)	315 (1.4%)
Hyperlipidemia: n (%)	5,647 (38.4%)	5,568 (37.9%)	2,332 (38.9%)	2,243 (37.4%)	13,836 (25.9%)	13,689 (25.6%)	6,560 (28.3%)	6,310 (27.2%)
Diabetes mellitus: n (%)	2,466 (16.8%)	2,371 (16.1%)	1,120 (18.7%)	1,095 (18.3%)	8,192 (15.3%)	8,069 (15.1%)	3,636 (15.7%)	3,483 (15.0%)
Hypertension: n (%)	10,543 (71.7%)	10,453 (71.1%)	4,280 (71.4%)	4,131 (68.9%)	31,951 (59.7%)	31,660 (59.2%)	13,840 (59.8%)	13,584 (58.7%)

Myocardial Infarction: n (%)	66 (0.4%)	63 (0.4%)	24 (0.4%)	23 (0.4%)	227 (0.4%)	241 (0.5%)	103 (0.4%)	97 (0.4%)
Angina: n (%)	308 (2.1%)	327 (2.2%)	117 (2.0%)	111 (1.9%)	924 (1.7%)	943 (1.8%)	408 (1.8%)	421 (1.8%)
Heart failure: n (%)	217 (1.5%)	215 (1.5%)	90 (1.5%)	80 (1.3%)	719 (1.3%)	753 (1.4%)	317 (1.4%)	298 (1.3%)
Other forms of chronic heart diseases: n (%)	857 (5.8%)	835 (5.7%)	407 (6.8%)	390 (6.5%)	2,868 (5.4%)	2,923 (5.5%)	1,268 (5.5%)	1,203 (5.2%)
Cerebrovascular accident: n (%)	201 (1.4%)	223 (1.5%)	93 (1.6%)	81 (1.4%)	734 (1.4%)	730 (1.4%)	326 (1.4%)	324 (1.4%)
Venous thromboembolism: n (%)	119 (0.8%)	110 (0.7%)	44 (0.7%)	41 (0.7%)	338 (0.6%)	340 (0.6%)	135 (0.6%)	113 (0.5%)
Atrial fibrillation: n (%)	180 (1.2%)	183 (1.2%)	83 (1.4%)	79 (1.3%)	720 (1.3%)	747 (1.4%)	361 (1.6%)	347 (1.5%)
Overweight or obese: n (%)	849 (5.8%)	800 (5.4%)	307 (5.1%)	257 (4.3%)	1,317 (2.5%)	1,290 (2.4%)	598 (2.6%)	547 (2.4%)
Tobacco use: n (%)	661 (4.5%)	679 (4.6%)	256 (4.3%)	222 (3.7%)	1,002 (1.9%)	980 (1.8%)	507 (2.2%)	485 (2.1%)
Alcohol abuse or dependence: n (%)	124 (0.8%)	139 (0.9%)	31 (0.5%)	28 (0.5%)	273 (0.5%)	262 (0.5%)	129 (0.6%)	124 (0.5%)
Renal disease: n (%)	190 (1.3%)	171 (1.2%)	82 (1.4%)	75 (1.3%)	513 (1.0%)	532 (1.0%)	216 (0.9%)	219 (0.9%)
Liver disease: n (%)	305 (2.1%)	310 (2.1%)	101 (1.7%)	97 (1.6%)	745 (1.4%)	760 (1.4%)	327 (1.4%)	305 (1.3%)
Aspirin: n (%)	4 (0.0%)	6 (0.0%)	1 (0.0%)	1 (0.0%)	240 (0.4%)	233 (0.4%)	85 (0.4%)	68 (0.3%)
Antiplatelets: n (%)	303 (2.1%)	321 (2.2%)	110 (1.8%)	112 (1.9%)	1,450 (2.7%)	1,496 (2.8%)	587 (2.5%)	569 (2.5%)
Statins: n (%)	2,929 (19.9%)	2,776 (18.9%)	1,109 (18.5%)	1,060 (17.7%)	11,766 (22.0%)	11,523 (21.5%)	4,698 (20.3%)	4,445 (19.2%)
Other lipid lowering agents: n (%)	957 (6.5%)	960 (6.5%)	386 (6.4%)	370 (6.2%)	3,786 (7.1%)	3,777 (7.1%)	1,469 (6.3%)	1,411 (6.1%)
Insulin preparations: n (%)	296 (2.0%)	305 (2.1%)	125 (2.1%)	117 (2.0%)	697 (1.3%)	734 (1.4%)	307 (1.3%)	293 (1.3%)
Oral hypoglycemic agents: n (%)	1,442 (9.8%)	1,395 (9.5%)	616 (10.3%)	587 (9.8%)	5,513 (10.3%)	5,443 (10.2%)	2,339 (10.1%)	2,170 (9.4%)
ACE inhibitors: n (%)	3,198 (21.7%)	3,183 (21.6%)	1,247 (20.8%)	1,134 (18.9%)	13,231 (24.7%)	12,899 (24.1%)	5,390 (23.3%)	5,257 (22.7%)
ARBs: n (%)	1,451 (9.9%)	1,481 (10.1%)	524 (8.7%)	572 (9.5%)	5,195 (9.7%)	5,307 (9.9%)	2,011 (8.7%)	2,116 (9.1%)
Calcium channel blockers (without amlodipine-benazepril): n (%)	904 (6.1%)	872 (5.9%)	335 (5.6%)	344 (5.7%)	3,302 (6.2%)	3,315 (6.2%)	1,331 (5.7%)	1,300 (5.6%)
Diuretics: n (%)	3,548 (24.1%)	3,531 (24.0%)	1,333 (22.2%)	1,309 (21.8%)	13,532 (25.3%)	13,450 (25.1%)	5,416 (23.4%)	5,361 (23.1%)
Beta-blockers: n (%)	2,471 (16.8%)	2,409 (16.4%)	852 (14.2%)	853 (14.2%)	9,597 (17.9%)	9,465 (17.7%)	3,958 (17.1%)	3,741 (16.2%)
Anticoagulants: n (%)	200 (1.4%)	193 (1.3%)	67 (1.1%)	62 (1.0%)	868 (1.6%)	889 (1.7%)	380 (1.6%)	385 (1.7%)
NSAIDs: n (%)	1,797 (12.2%)	1,791 (12.2%)	676 (11.3%)	664 (11.1%)	6,663 (12.5%)	6,463 (12.1%)	2,645 (11.4%)	2,537 (11.0%)
Coxibs: n (%)	219 (1.5%)	224 (1.5%)	104 (1.7%)	91 (1.5%)	1,023 (1.9%)	1,049 (2.0%)	380 (1.6%)	413 (1.8%)
Outpatient visits: mean (sd)	0.14 (0.38)	0.14 (0.39)	0.11 (0.33)	0.11 (0.34)	0.12 (0.35)	0.11 (0.35)	0.11 (0.35)	0.11 (0.35)

Inpatient hospitalization: mean (sd)	0.00 (0.05)	0.00 (0.05)	0.00 (0.06)	0.00 (0.04)	0.00 (0.00)	0.00 (0.00)	0.00 (0.00)	0.00 (0.00)
ED visit: mean (sd)	0.18 (0.56)	0.18 (0.62)	0.18 (0.57)	0.17 (0.65)	0.24 (0.82)	0.24 (0.85)	0.24 (0.83)	0.22 (0.82)
Number of distinct medication prescriptions: mean (sd)	3.90 (3.91)	3.84 (3.81)	3.57 (3.83)	3.46 (3.72)	4.12 (4.25)	4.11 (4.11)	3.75 (4.07)	3.69 (3.94)
Combined Comorbidity Score: mean (sd)	-0.26 (1.06)	-0.26 (1.05)	-0.25 (1.01)	-0.26 (1.04)	-0.24 (0.97)	-0.23 (0.97)	-0.23 (0.98)	-0.24 (0.96)

Table AC- Characteristics of patients included in the evaluation of comparative outcomes for *calcitonin salmon* between generic or authorized generics (AG) and brand initiators after 1:1 propensity score matching in each database

	Optum				Truven			
	Generic initiators	Brand initiators	AG initiators	Brand initiators	Generic initiators	Brand initiators	AG initiators	Brand initiators
**Cohort selection steps**								
Total number of patients filling prescriptions for the version of interest after 6-month continuous enrollment	**8,864**		**8,442**		**67,478**		**62,915**	
Patients meeting new drug use restriction	**5,806**		**5,496**		**31,375**		**28,482**	
Total eligible by exposure group	**946**	**4,860**	**636**	**4,860**	**6,308**	**24,772**	**3,426**	**24,775**
**1:1 PS matched**	**944**	**944**	**636**	**636**	**6,306**	**6,306**	**3,422**	**3,422**
**Baseline characteristics**								
Age: mean (sd)	62 (12.27)	62 (11.61)	59 (12.58)	60 (12.85)	68.37 (13.73)	68.42 (14.05)	66.23 (14.73)	65.77 (14.84)
Gender								
MALE: n (%)	142 (15.0%)	145 (15.4%)	102 (16.0%)	96 (15.1%)	848 (13.4%)	847 (13.4%)	518 (15.1%)	537 (15.7%)
FEMALE: n (%)	802 (85.0%)	799 (84.6%)	534 (84.0%)	540 (84.9%)	5,458 (86.6%)	5,459 (86.6%)	2,904 (84.9%)	2,885 (84.3%)
UNKNOWN: n (%)	0 (0.0%)	0 (0.0%)	0 (0.0%)	0 (0.0%)	0 (0.0%)	0 (0.0%)	0 (0.0%)	0 (0.0%)
Region								
Northeast: n (%)	95 (10.1%)	93 (9.9%)	51 (8.0%)	53 (8.3%)	959 (15.2%)	954 (15.1%)	575 (16.8%)	546 (16.0%)
Midwest: n (%)	193 (20.4%)	194 (20.6%)	151 (23.7%)	163 (25.6%)	1,939 (30.7%)	1,949 (30.9%)	1,058 (30.9%)	1,079 (31.5%)
South: n (%)	450 (47.7%)	448 (47.5%)	310 (48.7%)	286 (45.0%)	2,243 (35.6%)	2,227 (35.3%)	1,180 (34.5%)	1,203 (35.2%)
West: n (%)	206 (21.8%)	209 (22.1%)	124 (19.5%)	134 (21.1%)	1,121 (17.8%)	1,131 (17.9%)	566 (16.5%)	554 (16.2%)
Unknown/other: n (%)								
Outpatient visits: mean (sd)	0.29 (0.52)	0.27 (0.54)	0.27 (0.50)	0.29 (0.56)	0.18 (0.42)	0.19 (0.44)	0.20 (0.47)	0.21 (0.48)
Inpatient hospitalization: mean (sd)	0.05 (0.30)	0.05 (0.30)	0.06 (0.33)	0.06 (0.34)	0.00 (0.01)	0.00 (0.01)	0.00 (0.00)	0.00 (0.00)
ED visit: mean (sd)	0.40 (0.94)	0.41 (0.96)	0.48 (1.13)	0.47 (1.14)	0.51 (1.24)	0.50 (1.20)	0.61 (1.40)	0.59 (1.34)
Number of distinct medication prescriptions: mean (sd)	8.99 (5.80)	8.73 (6.00)	9.83 (6.34)	10.01 (6.50)	9.56 (5.75)	9.40 (5.93)	9.85 (6.03)	9.59 (6.06)

Combined Comorbidity Score: mean (sd)	0.85 (1.90)	0.87 (1.84)	0.88 (1.95)	0.98 (2.24)	0.74 (1.72)	0.68 (1.67)	0.86 (1.85)	0.81 (1.82)
Osteoporosis: n (%)	437 (46.3%)	440 (46.6%)	272 (42.8%)	262 (41.2%)	2,227 (35.3%)	2,165 (34.3%)	1,189 (34.7%)	1,196 (35.0%)
Kyphosis: n (%)	41 (4.3%)	40 (4.2%)	23 (3.6%)	23 (3.6%)	187 (3.0%)	183 (2.9%)	108 (3.2%)	88 (2.6%)
Vertebral facture: n (%)	164 (17.4%)	157 (16.6%)	124 (19.5%)	125 (19.7%)	1,035 (16.4%)	1,052 (16.7%)	651 (19.0%)	608 (17.8%)
Humerus fracture: n (%)	9 (1.0%)	5 (0.5%)	16 (2.5%)	17 (2.7%)	82 (1.3%)	86 (1.4%)	48 (1.4%)	48 (1.4%)
Wrist fracture: n (%)	16 (1.7%)	18 (1.9%)	12 (1.9%)	16 (2.5%)	88 (1.4%)	93 (1.5%)	51 (1.5%)	55 (1.6%)
Hip fracture: n (%)	21 (2.2%)	23 (2.4%)	11 (1.7%)	10 (1.6%)	166 (2.6%)	176 (2.8%)	87 (2.5%)	92 (2.7%)
Pelvis fracture: n (%)	23 (2.4%)	20 (2.1%)	11 (1.7%)	11 (1.7%)	118 (1.9%)	118 (1.9%)	62 (1.8%)	70 (2.0%)
Any other fractures: n (%)	97 (10.3%)	92 (9.7%)	82 (12.9%)	89 (14.0%)	614 (9.7%)	628 (10.0%)	419 (12.2%)	417 (12.2%)
Alzheimer disease or other dementia: n (%)	41 (4.3%)	40 (4.2%)	27 (4.2%)	31 (4.9%)	311 (4.9%)	293 (4.6%)	182 (5.3%)	160 (4.7%)
Asthma or chronic obstructive pulmonary disease: n (%)	122 (12.9%)	123 (13.0%)	80 (12.6%)	78 (12.3%)	669 (10.6%)	651 (10.3%)	394 (11.5%)	371 (10.8%)
Cataracts: n (%)	93 (9.9%)	111 (11.8%)	47 (7.4%)	49 (7.7%)	578 (9.2%)	562 (8.9%)	290 (8.5%)	292 (8.5%)
Crohn disease or gastroenteritis: n (%)	35 (3.7%)	44 (4.7%)	22 (3.5%)	21 (3.3%)	169 (2.7%)	157 (2.5%)	99 (2.9%)	87 (2.5%)
Depression: n (%)	29 (3.1%)	36 (3.8%)	21 (3.3%)	21 (3.3%)	165 (2.6%)	137 (2.2%)	114 (3.3%)	115 (3.4%)
Diabetes mellitus: n (%)	114 (12.1%)	114 (12.1%)	100 (15.7%)	92 (14.5%)	819 (13.0%)	817 (13.0%)	498 (14.6%)	478 (14.0%)
History of falls, syncope, or gait abnormality: n (%)	151 (16.0%)	138 (14.6%)	111 (17.5%)	121 (19.0%)	803 (12.7%)	775 (12.3%)	492 (14.4%)	485 (14.2%)
Hyperthyroidism: n (%)	18 (1.9%)	19 (2.0%)	10 (1.6%)	14 (2.2%)	52 (0.8%)	43 (0.7%)	41 (1.2%)	40 (1.2%)
Hyperparathyroidism: n (%)	15 (1.6%)	11 (1.2%)	9 (1.4%)	8 (1.3%)	56 (0.9%)	45 (0.7%)	36 (1.1%)	38 (1.1%)
Ischemic stroke: n (%)	12 (1.3%)	15 (1.6%)	7 (1.1%)	9 (1.4%)	99 (1.6%)	81 (1.3%)	63 (1.8%)	54 (1.6%)
Liver disease: n (%)	39 (4.1%)	48 (5.1%)	30 (4.7%)	25 (3.9%)	144 (2.3%)	136 (2.2%)	111 (3.2%)	109 (3.2%)
Malignant neoplasm: n (%)	120 (12.7%)	131 (13.9%)	67 (10.5%)	62 (9.7%)	676 (10.7%)	654 (10.4%)	368 (10.8%)	367 (10.7%)
Overweight or obese: n (%)	32 (3.4%)	40 (4.2%)	22 (3.5%)	16 (2.5%)	106 (1.7%)	92 (1.5%)	67 (2.0%)	63 (1.8%)
Parkinson disease: n (%)	9 (1.0%)	12 (1.3%)	5 (0.8%)	7 (1.1%)	74 (1.2%)	83 (1.3%)	33 (1.0%)	35 (1.0%)
Renal disease: n (%)	26 (2.8%)	24 (2.5%)	15 (2.4%)	12 (1.9%)	168 (2.7%)	152 (2.4%)	116 (3.4%)	95 (2.8%)
Rheumatoid arthritis: n (%)	50 (5.3%)	47 (5.0%)	30 (4.7%)	40 (6.3%)	282 (4.5%)	275 (4.4%)	165 (4.8%)	169 (4.9%)
Oral glucocorticoids: n (%)	244 (25.8%)	230 (24.4%)	198 (31.1%)	209 (32.9%)	1,756 (27.8%)	1,724 (27.3%)	1,034 (30.2%)	1,022 (29.9%)
Anticonvulsants/antiepileptic drugs: n (%)	130 (13.8%)	132 (14.0%)	102 (16.0%)	106 (16.7%)	821 (13.0%)	811 (12.9%)	492 (14.4%)	477 (13.9%)

Benzodiazepines: n (%)	216 (22.9%)	229 (24.3%)	168 (26.4%)	166 (26.1%)	1,370 (21.7%)	1,336 (21.2%)	781 (22.8%)	784 (22.9%)
SSRIs: n (%)	170 (18.0%)	184 (19.5%)	137 (21.5%)	145 (22.8%)	1,308 (20.7%)	1,243 (19.7%)	762 (22.3%)	738 (21.6%)
Beta-blockers: n (%)	183 (19.4%)	176 (18.6%)	122 (19.2%)	117 (18.4%)	1,686 (26.7%)	1,676 (26.6%)	837 (24.5%)	790 (23.1%)
Proton pump inhibitors: n (%)	245 (26.0%)	239 (25.3%)	165 (25.9%)	158 (24.8%)	1,982 (31.4%)	1,924 (30.5%)	1,074 (31.4%)	1,036 (30.3%)
Opioids: n (%)	446 (47.2%)	439 (46.5%)	357 (56.1%)	355 (55.8%)	3,062 (48.6%)	3,012 (47.8%)	1,766 (51.6%)	1,706 (49.9%)
Other anti-osteoporosis medications (without calcitonin): n (%)	249 (26.4%)	255 (27.0%)	177 (27.8%)	161 (25.3%)	1,771 (28.1%)	1,772 (28.1%)	902 (26.4%)	876 (25.6%)

Table AD- Characteristics of patients included in the evaluation of comparative outcomes for *escitalopram* between generic or authorized generics (AG) and brand initiators after 1:1 propensity score matching in each database

	Optum				Truven			
	**Generic initiators**	**Brand initiators**	**AG initiators**	**Brand initiators**	**Generic initiators**	**Brand initiators**	**AG initiators**	**Brand initiators**
**Cohort selection steps**								
Total number of patients filling prescriptions for the version of interest after 6-month continuous enrollment	235,490		197,080		1,350,114		1,045,259	
Patients meeting new drug use restriction	136,104		107,633		643,412		425,365	
Total eligible by exposure group	54,051	82,053	25,580	82,053	321,070	322,342	103,017	322,348
**1:1 PS matched**	53,711	53,711	25,540	25,540	301,337	301,337	103,010	103,010
**Baseline characteristics**								
Age: mean (sd)	39 (14.76)	39 (14.82)	39 (14.98)	39 (14.86)	42.52 (17.47)	42.43 (17.02)	42.61 (17.50)	42.32 (17.04)
Gender								
MALE: n (%)	17,870 (33.3%)	17,982 (33.5%)	8,790 (34.4%)	8,725 (34.2%)	96,097 (31.9%)	96,230 (31.9%)	32,959 (32.0%)	32,884 (31.9%)
FEMALE: n (%)	35,834 (66.7%)	35,723 (66.5%)	16,746 (65.6%)	16,810 (65.8%)	205,240 (68.1%)	205,107 (68.1%)	70,051 (68.0%)	70,126 (68.1%)
UNKNOWN: n (%)	7 (0.0%)	6 (0.0%)	4 (0.0%)	5 (0.0%)	0 (0.0%)	0 (0.0%)	0 (0.0%)	0 (0.0%)
Region								
Northeast: n (%)	5,572 (10.4%)	5,444 (10.1%)	2,020 (7.9%)	2,000 (7.8%)	57,233 (19.0%)	57,191 (19.0%)	16,756 (16.3%)	16,771 (16.3%)
Midwest: n (%)	14,010 (26.1%)	13,895 (25.9%)	6,300 (24.7%)	6,298 (24.7%)	70,027 (23.2%)	69,967 (23.2%)	23,119 (22.4%)	22,943 (22.3%)
South: n (%)	25,464 (47.4%)	25,799 (48.0%)	13,005 (50.9%)	13,109 (51.3%)	122,497 (40.7%)	122,818 (40.8%)	46,088 (44.7%)	46,468 (45.1%)
West: n (%)	8,654 (16.1%)	8,563 (15.9%)	4,206 (16.5%)	4,124 (16.1%)	41,465 (13.8%)	41,233 (13.7%)	15,015 (14.6%)	14,706 (14.3%)
Unknown/other: n (%)	11 (0.0%)	10 (0.0%)	9 (0.0%)	9 (0.0%)	10,115 (3.4%)	10,128 (3.4%)	2,032 (2.0%)	2,122 (2.1%)
Epilepsy: n (%)	709 (1.3%)	676 (1.3%)	357 (1.4%)	306 (1.2%)	4,301 (1.4%)	4,265 (1.4%)	1,452 (1.4%)	1,334 (1.3%)
Depression: n (%)	5,349 (10.0%)	5,160 (9.6%)	2,593 (10.2%)	2,556 (10.0%)	31,420 (10.4%)	30,961 (10.3%)	10,400 (10.1%)	9,757 (9.5%)

Anxiety: n (%)	5,657 (10.5%)	5,383 (10.0%)	2,666 (10.4%)	2,436 (9.5%)	26,592 (8.8%)	26,541 (8.8%)	8,880 (8.6%)	8,197 (8.0%)
Alcohol abuse or dependence: n (%)	1,176 (2.2%)	1,117 (2.1%)	576 (2.3%)	538 (2.1%)	4,986 (1.7%)	4,949 (1.6%)	1,762 (1.7%)	1,575 (1.5%)
Drug abuse or dependence: n (%)	1,316 (2.5%)	1,268 (2.4%)	601 (2.4%)	565 (2.2%)	5,782 (1.9%)	5,650 (1.9%)	2,137 (2.1%)	1,985 (1.9%)
Sleep disorder: n (%)	4,528 (8.4%)	4,412 (8.2%)	2,211 (8.7%)	2,112 (8.3%)	20,096 (6.7%)	19,981 (6.6%)	6,834 (6.6%)	6,372 (6.2%)
Psychotic disorder: n (%)	792 (1.5%)	780 (1.5%)	426 (1.7%)	411 (1.6%)	4,827 (1.6%)	4,758 (1.6%)	1,680 (1.6%)	1,560 (1.5%)
Personality disorder: n (%)	295 (0.5%)	281 (0.5%)	119 (0.5%)	114 (0.4%)	1,295 (0.4%)	1,282 (0.4%)	467 (0.5%)	439 (0.4%)
Adjustment reaction/post-traumatic stress disorder: n (%)	3,331 (6.2%)	3,235 (6.0%)	1,564 (6.1%)	1,543 (6.0%)	16,792 (5.6%)	16,654 (5.5%)	5,766 (5.6%)	5,340 (5.2%)
ADHD: n (%)	3,162 (5.9%)	3,075 (5.7%)	1,467 (5.7%)	1,413 (5.5%)	12,895 (4.3%)	12,650 (4.2%)	4,601 (4.5%)	4,213 (4.1%)
Delirium: n (%)	667 (1.2%)	670 (1.2%)	321 (1.3%)	317 (1.2%)	4,351 (1.4%)	4,269 (1.4%)	1,454 (1.4%)	1,342 (1.3%)
Bipolar disorder: n (%)	138 (0.3%)	135 (0.3%)	80 (0.3%)	70 (0.3%)	689 (0.2%)	683 (0.2%)	240 (0.2%)	222 (0.2%)
Other psychiatric disorder: n (%)	2,691 (5.0%)	2,582 (4.8%)	1,269 (5.0%)	1,205 (4.7%)	12,367 (4.1%)	12,192 (4.0%)	4,102 (4.0%)	3,782 (3.7%)
Psychiatric hospitalization: mean (sd)	0.03 (0.25)	0.03 (0.23)	0.04 (0.24)	0.04 (0.24)	0.03 (0.21)	0.03 (0.21)	0.03 (0.21)	0.03 (0.20)
Psychiatric office visit: mean (sd)	2.39 (8.19)	2.33 (7.31)	2.42 (8.02)	2.30 (7.72)	2.38 (9.12)	2.33 (7.31)	2.42 (8.24)	2.19 (7.39)
Anticonvulsants/antiepileptic drugs: n (%)	4,148 (7.7%)	4,035 (7.5%)	2,040 (8.0%)	1,908 (7.5%)	24,429 (8.1%)	24,182 (8.0%)	8,641 (8.4%)	7,826 (7.6%)
SSRIs (without escitalopram): n (%)	11,243 (20.9%)	10,792 (20.1%)	5,657 (22.1%)	5,416 (21.2%)	60,456 (20.1%)	59,865 (19.9%)	21,762 (21.1%)	20,633 (20.0%)
SNRIs: n (%)	2,901 (5.4%)	2,790 (5.2%)	1,355 (5.3%)	1,306 (5.1%)	16,099 (5.3%)	15,905 (5.3%)	5,544 (5.4%)	5,120 (5.0%)
Tricyclic antidepressants: n (%)	1,242 (2.3%)	1,186 (2.2%)	595 (2.3%)	560 (2.2%)	7,506 (2.5%)	7,430 (2.5%)	2,666 (2.6%)	2,461 (2.4%)
Antipsychotics: n (%)	2,227 (4.1%)	2,187 (4.1%)	1,102 (4.3%)	1,046 (4.1%)	13,756 (4.6%)	13,703 (4.5%)	4,664 (4.5%)	4,296 (4.2%)
Benzodiazepines: n (%)	12,759 (23.8%)	12,387 (23.1%)	6,304 (24.7%)	5,943 (23.3%)	73,937 (24.5%)	73,036 (24.2%)	25,192 (24.5%)	23,894 (23.2%)
Non-BZD sedative hypnotics: n (%)	6,198 (11.5%)	5,986 (11.1%)	3,010 (11.8%)	2,854 (11.2%)	35,919 (11.9%)	35,641 (11.8%)	12,451 (12.1%)	11,900 (11.6%)
Outpatient visits: mean (sd)	0.26 (0.55)	0.25 (0.53)	0.26 (0.51)	0.25 (0.50)	0.22 (0.47)	0.22 (0.47)	0.22 (0.48)	0.21 (0.47)
Inpatient hospitalization: mean (sd)	0.01 (0.17)	0.01 (0.11)	0.01 (0.12)	0.01 (0.10)	0.00 (0.01)	0.00 (0.01)	0.00 (0.01)	0.00 (0.01)
ED visit: mean (sd)	0.27 (0.77)	0.26 (0.88)	0.28 (0.87)	0.26 (0.91)	0.41 (1.20)	0.40 (1.22)	0.42 (1.25)	0.38 (1.26)
Number of distinct medication prescriptions: mean (sd)	4.47 (4.17)	4.36 (4.12)	4.54 (4.29)	4.39 (4.18)	4.93 (4.56)	4.90 (4.56)	4.99 (4.59)	4.79 (4.48)
Combined Comorbidity Score: mean (sd)	0.32 (1.07)	0.31 (1.05)	0.35 (1.14)	0.34 (1.11)	0.39 (1.25)	0.38 (1.23)	0.39 (1.24)	0.35 (1.19)

Table AE- Characteristics of patients included in the evaluation of comparative outcomes for *glipizide* between generic or authorized generics (AG) and brand initiators after 1:1 propensity score matching in each database

	Optum				Truven			
	Generic initiators	Brand initiators	AG initiators	Brand initiators	Generic initiators	Brand initiators	AG initiators	Brand initiators
**Cohort selection steps**								
Total number of patients filling prescriptions for the version of interest after 6-month continuous enrollment	**22,864**		**5,355**		**132,724**		**59,108**	
Patients meeting new drug use restriction	**16,754**		**2,117**		**36,924**		**5,947**	
Total eligible by exposure group	**15,757**	**997**	**1,120**	**997**	**34,590**	**2,334**	**3,607**	**2,340**
**1:1 PS matched**	**997**	**997**	**825**	**825**	**2,334**	**2,334**	**2,202**	**2,202**
**Baseline characteristics**								
Age: mean (sd)	57.74 (12.60)	58.30 (12.53)	57.98 (11.28)	57.97 (12.27)	61.25 (13.80)	61.61 (13.48)	61.47 (13.16)	61.61 (13.39)
Gender								
MALE: n (%)	539 (54.1%)	506 (50.8%)	452 (54.8%)	440 (53.3%)	1,193 (51.1%)	1,172 (50.2%)	1,118 (50.8%)	1,107 (50.3%)
FEMALE: n (%)	458 (45.9%)	491 (49.2%)	373 (45.2%)	385 (46.7%)	1,141 (48.9%)	1,162 (49.8%)	1,084 (49.2%)	1,095 (49.7%)
UNKNOWN: n (%)	0 (0.0%)	0 (0.0%)	0 (0.0%)	0 (0.0%)	0 (0.0%)	0 (0.0%)	0 (0.0%)	0 (0.0%)
Region								
Northeast: n (%)	175 (17.6%)	187 (18.8%)	152 (18.4%)	159 (19.3%)	215 (9.2%)	199 (8.5%)	202 (9.2%)	199 (9.0%)
Midwest: n (%)	272 (27.3%)	285 (28.6%)	224 (27.2%)	236 (28.6%)	575 (24.6%)	605 (25.9%)	604 (27.4%)	606 (27.5%)
South: n (%)	471 (47.2%)	462 (46.3%)	388 (47.0%)	374 (45.3%)	1,058 (45.3%)	1,052 (45.1%)	980 (44.5%)	974 (44.2%)
West: n (%)	72 (7.2%)	57 (5.7%)	60 (7.3%)	56 (6.8%)	461 (19.8%)	451 (19.3%)	395 (17.9%)	403 (18.3%)
Unknown/other: n (%)	7 (0.7%)	6 (0.6%)	1 (0.1%)	0 (0.0%)	25 (1.1%)	27 (1.2%)	21 (1.0%)	20 (0.9%)
Hyperlipidemia: n (%)	453 (45.4%)	446 (44.7%)	385 (46.7%)	386 (46.8%)	464 (19.9%)	473 (20.3%)	442 (20.1%)	457 (20.8%)
Diabetes mellitus: n (%)	695 (69.7%)	722 (72.4%)	596 (72.2%)	605 (73.3%)	1,540 (66.0%)	1,501 (64.3%)	1,456 (66.1%)	1,459 (66.3%)
Hypertension: n (%)	497 (49.8%)	517 (51.9%)	424 (51.4%)	431 (52.2%)	759 (32.5%)	771 (33.0%)	746 (33.9%)	753 (34.2%)
Myocardial Infarction: n (%)	9 (0.9%)	11 (1.1%)	9 (1.1%)	9 (1.1%)	25 (1.1%)	27 (1.2%)	27 (1.2%)	27 (1.2%)
Angina: n (%)	42 (4.2%)	39 (3.9%)	32 (3.9%)	31 (3.8%)	81 (3.5%)	84 (3.6%)	74 (3.4%)	79 (3.6%)

Heart failure: n (%)	59 (5.9%)	62 (6.2%)	46 (5.6%)	46 (5.6%)	156 (6.7%)	170 (7.3%)	161 (7.3%)	155 (7.0%)
Other forms of chronic heart diseases: n (%)	160 (16.0%)	146 (14.6%)	108 (13.1%)	111 (13.5%)	256 (11.0%)	266 (11.4%)	261 (11.9%)	260 (11.8%)
Cerebrovascular accident: n (%)	17 (1.7%)	20 (2.0%)	10 (1.2%)	14 (1.7%)	46 (2.0%)	54 (2.3%)	48 (2.2%)	52 (2.4%)
Venous thromboembolism: n (%)	9 (0.9%)	11 (1.1%)	8 (1.0%)	10 (1.2%)	45 (1.9%)	40 (1.7%)	34 (1.5%)	35 (1.6%)
Atrial fibrillation: n (%)	59 (5.9%)	54 (5.4%)	29 (3.5%)	33 (4.0%)	107 (4.6%)	109 (4.7%)	100 (4.5%)	99 (4.5%)
Overweight or obese: n (%)	45 (4.5%)	55 (5.5%)	44 (5.3%)	39 (4.7%)	33 (1.4%)	30 (1.3%)	29 (1.3%)	29 (1.3%)
Tobacco use: n (%)	22 (2.2%)	20 (2.0%)	16 (1.9%)	16 (1.9%)	23 (1.0%)	23 (1.0%)	23 (1.0%)	22 (1.0%)
Alcohol abuse or dependence: n (%)	1 (0.1%)	3 (0.3%)	4 (0.5%)	2 (0.2%)	10 (0.4%)	12 (0.5%)	8 (0.4%)	11 (0.5%)
Renal disease: n (%)	23 (2.3%)	31 (3.1%)	21 (2.5%)	23 (2.8%)	45 (1.9%)	52 (2.2%)	49 (2.2%)	50 (2.3%)
Liver disease: n (%)	27 (2.7%)	30 (3.0%)	20 (2.4%)	22 (2.7%)	43 (1.8%)	47 (2.0%)	45 (2.0%)	42 (1.9%)
Aspirin: n (%)	1 (0.1%)	1 (0.1%)	0 (0.0%)	0 (0.0%)	0 (0.0%)	0 (0.0%)	0 (0.0%)	0 (0.0%)
Antiplatelets: n (%)	38 (3.8%)	37 (3.7%)	33 (4.0%)	30 (3.6%)	106 (4.5%)	117 (5.0%)	110 (5.0%)	116 (5.3%)
Statins: n (%)	232 (23.3%)	247 (24.8%)	225 (27.3%)	225 (27.3%)	752 (32.2%)	735 (31.5%)	747 (33.9%)	732 (33.2%)
Other lipid lowering agents: n (%)	66 (6.6%)	69 (6.9%)	62 (7.5%)	62 (7.5%)	152 (6.5%)	166 (7.1%)	170 (7.7%)	166 (7.5%)
Insulin preparations: n (%)	0 (0.0%)	0 (0.0%)	0 (0.0%)	0 (0.0%)	0 (0.0%)	0 (0.0%)	0 (0.0%)	0 (0.0%)
Oral hypoglycemic agents (without glipizide): n (%)	345 (34.6%)	365 (36.6%)	341 (41.3%)	338 (41.0%)	1,012 (43.4%)	1,015 (43.5%)	1,032 (46.9%)	1,018 (46.2%)
ACE inhibitors: n (%)	198 (19.9%)	214 (21.5%)	200 (24.2%)	198 (24.0%)	751 (32.2%)	707 (30.3%)	699 (31.7%)	703 (31.9%)
ARBs: n (%)	115 (11.5%)	120 (12.0%)	100 (12.1%)	98 (11.9%)	366 (15.7%)	386 (16.5%)	382 (17.3%)	375 (17.0%)
Calcium channel blockers: n (%)	119 (11.9%)	123 (12.3%)	107 (13.0%)	109 (13.2%)	453 (19.4%)	472 (20.2%)	453 (20.6%)	458 (20.8%)
Diuretics: n (%)	251 (25.2%)	265 (26.6%)	219 (26.5%)	219 (26.5%)	819 (35.1%)	851 (36.5%)	822 (37.3%)	822 (37.3%)
Beta-blockers: n (%)	140 (14.0%)	151 (15.1%)	130 (15.8%)	137 (16.6%)	487 (20.9%)	515 (22.1%)	505 (22.9%)	507 (23.0%)
Anticoagulants: n (%)	60 (6.0%)	55 (5.5%)	32 (3.9%)	28 (3.4%)	119 (5.1%)	136 (5.8%)	126 (5.7%)	125 (5.7%)
NSAIDs: n (%)	82 (8.2%)	77 (7.7%)	64 (7.8%)	70 (8.5%)	226 (9.7%)	244 (10.5%)	226 (10.3%)	242 (11.0%)
Coxibs: n (%)	45 (4.5%)	59 (5.9%)	36 (4.4%)	36 (4.4%)	175 (7.5%)	192 (8.2%)	184 (8.4%)	183 (8.3%)
Outpatient visits: mean (sd)	0.09 (0.30)	0.09 (0.36)	0.10 (0.33)	0.09 (0.37)	0.05 (0.25)	0.06 (0.28)	0.06 (0.25)	0.06 (0.29)
Inpatient hospitalization: mean (sd)	0.01 (0.14)	0.01 (0.13)	0.01 (0.09)	0.01 (0.10)	0.00 (0.00)	0.00 (0.00)	0.00 (0.00)	0.00 (0.00)
ED visit: mean (sd)	0.27 (0.82)	0.29 (0.88)	0.21 (0.71)	0.22 (0.67)	0.25 (0.71)	0.27 (0.78)	0.27 (0.73)	0.26 (0.73)
Number of distinct medication prescriptions: mean (sd)	4.91 (4.88)	5.17 (4.98)	5.01 (4.63)	4.97 (4.61)	6.26 (5.23)	6.47 (5.47)	6.64 (5.32)	6.58 (5.43)

Combined Comorbidity Score: mean (sd)	0.43 (1.69)	0.45 (1.65)	0.31 (1.57)	0.33 (1.51)	0.42 (1.40)	0.46 (1.46)	0.44 (1.43)	0.45 (1.44)

Table AF- Characteristics of patients included in the evaluation of comparative outcomes for *quinapril* between generic or authorized generics (AG) and brand initiators after 1:1 propensity score matching in each database

	Optum				Truven			
	**Generic initiators**	**Brand initiators**	**AG initiators**	**Brand initiators**	**Generic initiators**	**Brand initiators**	**AG initiators**	**Brand initiators**
**Cohort selection steps**								
Total number of patients filling prescriptions for the version of interest after 6-month continuous enrollment	17,732		18,781		138,664		155,376	
Patients meeting new drug use restriction	11,041		10,162		21,781		23,813	
Total eligible by exposure group	4,597	6,444	3,718	6,444	8,255	12,739	9,985	12,740
**1:1 PS matched**	4,480	4,480	3,684	3,684	8,049	8,049	9,262	9,262
**Baseline characteristics**								
Age: mean (sd)	53 (11.89)	53 (12.52)	54 (11.92)	54 (12.39)	56.58 (14.21)	56.55 (14.17)	58.48 (13.93)	58.47 (13.92)
Gender								
MALE: n (%)	2,579 (57.6%)	2,599 (58.0%)	2,121 (57.6%)	2,138 (58.0%)	4,163 (51.7%)	4,189 (52.0%)	5,006 (54.0%)	5,039 (54.4%)
FEMALE: n (%)	1,899 (42.4%)	1,880 (42.0%)	1,562 (42.4%)	1,545 (41.9%)	3,886 (48.3%)	3,860 (48.0%)	4,256 (46.0%)	4,223 (45.6%)
UNKNOWN: n (%)	2 (0.0%)	1 (0.0%)	1 (0.0%)	1 (0.0%)	0 (0.0%)	0 (0.0%)	0 (0.0%)	0 (0.0%)
Region								
Northeast: n (%)	586 (13.1%)	571 (12.7%)	643 (17.5%)	664 (18.0%)	845 (10.5%)	856 (10.6%)	1,427 (15.4%)	1,406 (15.2%)
Midwest: n (%)	1,222 (27.3%)	1,189 (26.5%)	997 (27.1%)	1,003 (27.2%)	2,092 (26.0%)	2,113 (26.3%)	2,778 (30.0%)	2,777 (30.0%)
South: n (%)	2,301 (51.4%)	2,331 (52.0%)	1,687 (45.8%)	1,677 (45.5%)	3,937 (48.9%)	3,932 (48.9%)	3,760 (40.6%)	3,749 (40.5%)
West: n (%)	369 (8.2%)	384 (8.6%)	353 (9.6%)	337 (9.1%)	1,126 (14.0%)	1,104 (13.7%)	1,243 (13.4%)	1,272 (13.7%)
Unknown/other: n (%)	2 (0.0%)	5 (0.1%)	4 (0.1%)	3 (0.1%)	49 (0.6%)	44 (0.5%)	54 (0.6%)	58 (0.6%)
Hyperlipidemia: n (%)	1,735 (38.7%)	1,736 (38.8%)	1,513 (41.1%)	1,512 (41.0%)	1,593 (19.8%)	1,585 (19.7%)	1,951 (21.1%)	1,942 (21.0%)
Diabetes mellitus: n (%)	1,206 (26.9%)	1,215 (27.1%)	978 (26.5%)	1,004 (27.3%)	1,855 (23.0%)	1,841 (22.9%)	2,310 (24.9%)	2,316 (25.0%)
Hypertension: n (%)	2,694 (60.1%)	2,653 (59.2%)	2,256 (61.2%)	2,242 (60.9%)	3,577 (44.4%)	3,481 (43.2%)	4,218 (45.5%)	4,160 (44.9%)
Myocardial Infarction: n (%)	144 (3.2%)	143 (3.2%)	76 (2.1%)	72 (2.0%)	230 (2.9%)	241 (3.0%)	230 (2.5%)	237 (2.6%)
Angina: n (%)	225 (5.0%)	224 (5.0%)	170 (4.6%)	168 (4.6%)	324 (4.0%)	329 (4.1%)	395 (4.3%)	397 (4.3%)

Heart failure: n (%)	231 (5.2%)	227 (5.1%)	180 (4.9%)	180 (4.9%)	508 (6.3%)	518 (6.4%)	518 (5.6%)	515 (5.6%)
Other forms of chronic heart diseases: n (%)	554 (12.4%)	555 (12.4%)	456 (12.4%)	464 (12.6%)	843 (10.5%)	839 (10.4%)	1,089 (11.8%)	1,111 (12.0%)
Cerebrovascular accident: n (%)	103 (2.3%)	94 (2.1%)	66 (1.8%)	68 (1.8%)	209 (2.6%)	209 (2.6%)	228 (2.5%)	230 (2.5%)
Venous thromboembolism: n (%)	61 (1.4%)	60 (1.3%)	42 (1.1%)	40 (1.1%)	89 (1.1%)	94 (1.2%)	116 (1.3%)	112 (1.2%)
Atrial fibrillation: n (%)	161 (3.6%)	147 (3.3%)	137 (3.7%)	125 (3.4%)	331 (4.1%)	331 (4.1%)	347 (3.7%)	332 (3.6%)
Overweight or obese: n (%)	255 (5.7%)	248 (5.5%)	133 (3.6%)	132 (3.6%)	109 (1.4%)	108 (1.3%)	117 (1.3%)	119 (1.3%)
Tobacco use: n (%)	203 (4.5%)	206 (4.6%)	139 (3.8%)	139 (3.8%)	110 (1.4%)	102 (1.3%)	114 (1.2%)	111 (1.2%)
Alcohol abuse or dependence: n (%)	54 (1.2%)	53 (1.2%)	35 (1.0%)	27 (0.7%)	30 (0.4%)	28 (0.3%)	44 (0.5%)	41 (0.4%)
Renal disease: n (%)	101 (2.3%)	98 (2.2%)	73 (2.0%)	68 (1.8%)	140 (1.7%)	138 (1.7%)	141 (1.5%)	143 (1.5%)
Liver disease: n (%)	102 (2.3%)	100 (2.2%)	81 (2.2%)	77 (2.1%)	103 (1.3%)	103 (1.3%)	126 (1.4%)	120 (1.3%)
Aspirin: n (%)	4 (0.1%)	6 (0.1%)	1 (0.0%)	0 (0.0%)	49 (0.6%)	39 (0.5%)	60 (0.6%)	49 (0.5%)
Antiplatelets: n (%)	132 (2.9%)	131 (2.9%)	118 (3.2%)	124 (3.4%)	334 (4.1%)	344 (4.3%)	427 (4.6%)	432 (4.7%)
Statins: n (%)	865 (19.3%)	854 (19.1%)	817 (22.2%)	827 (22.4%)	1,826 (22.7%)	1,791 (22.3%)	2,439 (26.3%)	2,468 (26.6%)
Other lipid lowering agents: n (%)	263 (5.9%)	251 (5.6%)	237 (6.4%)	228 (6.2%)	435 (5.4%)	425 (5.3%)	471 (5.1%)	464 (5.0%)
Insulin preparations: n (%)	200 (4.5%)	197 (4.4%)	145 (3.9%)	151 (4.1%)	208 (2.6%)	217 (2.7%)	261 (2.8%)	266 (2.9%)
Oral hypoglycemic agents: n (%)	736 (16.4%)	739 (16.5%)	601 (16.3%)	638 (17.3%)	1,393 (17.3%)	1,393 (17.3%)	1,691 (18.3%)	1,676 (18.1%)
ACE inhibitors: n (%)	559 (12.5%)	546 (12.2%)	470 (12.8%)	460 (12.5%)	1,343 (16.7%)	1,327 (16.5%)	1,659 (17.9%)	1,676 (18.1%)
ARBs: n (%)	258 (5.8%)	254 (5.7%)	226 (6.1%)	233 (6.3%)	548 (6.8%)	538 (6.7%)	650 (7.0%)	648 (7.0%)
Calcium channel blockers (without quinapril): n (%)	608 (13.6%)	593 (13.2%)	500 (13.6%)	494 (13.4%)	674 (8.4%)	669 (8.3%)	765 (8.3%)	748 (8.1%)
Diuretics: n (%)	1,054 (23.5%)	1,049 (23.4%)	848 (23.0%)	849 (23.0%)	2,243 (27.9%)	2,196 (27.3%)	2,640 (28.5%)	2,633 (28.4%)
Beta-blockers: n (%)	788 (17.6%)	791 (17.7%)	619 (16.8%)	621 (16.9%)	1,620 (20.1%)	1,580 (19.6%)	1,989 (21.5%)	1,958 (21.1%)
Anticoagulants: n (%)	126 (2.8%)	120 (2.7%)	106 (2.9%)	116 (3.1%)	298 (3.7%)	311 (3.9%)	356 (3.8%)	359 (3.9%)
NSAIDs: n (%)	506 (11.3%)	501 (11.2%)	405 (11.0%)	404 (11.0%)	1,001 (12.4%)	990 (12.3%)	1,073 (11.6%)	1,049 (11.3%)
Coxibs: n (%)	102 (2.3%)	104 (2.3%)	87 (2.4%)	87 (2.4%)	283 (3.5%)	291 (3.6%)	319 (3.4%)	309 (3.3%)
Outpatient visits: mean (sd)	0.14 (0.40)	0.14 (0.48)	0.13 (0.38)	0.13 (0.42)	0.10 (0.33)	0.09 (0.34)	0.10 (0.33)	0.09 (0.35)
Inpatient hospitalization: mean (sd)	0.01 (0.13)	0.01 (0.11)	0.01 (0.10)	0.01 (0.09)	0.00 (0.00)	0.00 (0.00)	0.00 (0.00)	0.00 (0.00)
ED visit: mean (sd)	0.27 (0.93)	0.27 (0.96)	0.23 (0.75)	0.23 (0.89)	0.29 (0.82)	0.29 (0.85)	0.26 (0.77)	0.26 (0.77)
Number of distinct medication prescriptions: mean (sd)	4.15 (4.21)	4.11 (4.19)	3.96 (3.96)	3.95 (4.03)	4.95 (4.91)	4.94 (4.83)	4.93 (4.72)	4.91 (4.67)

Combined Comorbidity Score: mean (sd)	0.10 (1.44)	0.10 (1.43)	0.02 (1.32)	0.02 (1.33)	0.18 (1.35)	0.19 (1.35)	0.15 (1.31)	0.15 (1.29)

Table AG- Characteristics of patients included in the evaluation of comparative outcomes for *sertraline* between generic or authorized generics (AG) and brand initiators after 1:1 propensity score matching in each database

	Optum				Truven			
	Generic initiators	Brand initiators	AG initiators	Brand initiators	Generic initiators	Brand initiators	AG initiators	Brand initiators
**Cohort selection steps**								
Total number of patients filling prescriptions for the version of interest after 6-month continuous enrollment	263,912		259,228		821,509		796,518	
Patients meeting new drug use restriction	190,914		192,092		387,055		386,146	
Total eligible by exposure group	54,572	136,342	55,750	136,342	133,010	254,045	132,099	254,047
**1:1 PS matched**	54,493	54,493	55,674	55,674	132,995	132,995	132,067	132,067
**Baseline characteristics**								
Age: mean (sd)	40 (15.34)	39 (15.05)	39 (14.92)	38 (14.86)	43.82 (17.80)	43.84 (18.06)	42.45 (17.30)	42.44 (18.02)
Gender								
MALE: n (%)	17,110 (31.4%)	16,884 (31.0%)	17,472 (31.4%)	17,303 (31.1%)	41,631 (31.3%)	41,424 (31.1%)	41,592 (31.5%)	41,698 (31.6%)
FEMALE: n (%)	37,376 (68.6%)	37,599 (69.0%)	38,197 (68.6%)	38,368 (68.9%)	91,364 (68.7%)	91,571 (68.9%)	90,475 (68.5%)	90,369 (68.4%)
UNKNOWN: n (%)	7 (0.0%)	10 (0.0%)	5 (0.0%)	3 (0.0%)	0 (0.0%)	0 (0.0%)	0 (0.0%)	0 (0.0%)
Region								
Northeast: n (%)	5,794 (10.6%)	5,420 (9.9%)	3,472 (6.2%)	3,562 (6.4%)	14,514 (10.9%)	14,151 (10.6%)	9,338 (7.1%)	9,340 (7.1%)
Midwest: n (%)	15,302 (28.1%)	15,665 (28.7%)	18,040 (32.4%)	18,189 (32.7%)	38,523 (29.0%)	38,128 (28.7%)	44,556 (33.7%)	44,232 (33.5%)
South: n (%)	27,846 (51.1%)	27,769 (51.0%)	25,564 (45.9%)	25,530 (45.9%)	65,812 (49.5%)	65,876 (49.5%)	55,215 (41.8%)	55,831 (42.3%)
West: n (%)	5,522 (10.1%)	5,607 (10.3%)	8,576 (15.4%)	8,373 (15.0%)	13,579 (10.2%)	14,247 (10.7%)	22,393 (17.0%)	22,051 (16.7%)
Unknown/other: n (%)	29 (0.1%)	32 (0.1%)	22 (0.0%)	20 (0.0%)	567 (0.4%)	593 (0.4%)	565 (0.4%)	613 (0.5%)
Epilepsy: n (%)	645 (1.2%)	615 (1.1%)	720 (1.3%)	689 (1.2%)	1,540 (1.2%)	1,428 (1.1%)	1,465 (1.1%)	1,352 (1.0%)
Depression: n (%)	4,985 (9.1%)	4,685 (8.6%)	5,278 (9.5%)	4,887 (8.8%)	9,313 (7.0%)	8,555 (6.4%)	9,920 (7.5%)	9,122 (6.9%)
Anxiety: n (%)	4,025 (7.4%)	3,833 (7.0%)	4,323 (7.8%)	3,896 (7.0%)	6,102 (4.6%)	5,632 (4.2%)	6,662 (5.0%)	6,067 (4.6%)
Alcohol abuse or dependence: n	938 (1.7%)	846 (1.6%)	1,119 (2.0%)	1,041 (1.9%)	1,432 (1.1%)	1,300 (1.0%)	1,611 (1.2%)	1,438 (1.1%)

(%)								
Drug abuse or dependence: n (%)	896 (1.6%)	819 (1.5%)	1,032 (1.9%)	960 (1.7%)	1,263 (0.9%)	1,168 (0.9%)	1,568 (1.2%)	1,420 (1.1%)
Sleep disorder: n (%)	4,642 (8.5%)	4,436 (8.1%)	4,807 (8.6%)	4,523 (8.1%)	6,876 (5.2%)	6,348 (4.8%)	6,892 (5.2%)	6,420 (4.9%)
Psychotic disorder: n (%)	613 (1.1%)	589 (1.1%)	701 (1.3%)	664 (1.2%)	1,356 (1.0%)	1,260 (0.9%)	1,532 (1.2%)	1,440 (1.1%)
Personality disorder: n (%)	316 (0.6%)	278 (0.5%)	350 (0.6%)	343 (0.6%)	387 (0.3%)	387 (0.3%)	414 (0.3%)	374 (0.3%)
Adjustment reaction/post-traumatic stress disorder: n (%)	2,776 (5.1%)	2,524 (4.6%)	3,073 (5.5%)	2,820 (5.1%)	4,960 (3.7%)	4,532 (3.4%)	5,407 (4.1%)	5,021 (3.8%)
ADHD: n (%)	2,007 (3.7%)	1,825 (3.3%)	2,196 (3.9%)	2,011 (3.6%)	3,045 (2.3%)	2,781 (2.1%)	3,412 (2.6%)	3,149 (2.4%)
Delirium: n (%)	497 (0.9%)	456 (0.8%)	543 (1.0%)	517 (0.9%)	1,170 (0.9%)	1,067 (0.8%)	1,174 (0.9%)	1,085 (0.8%)
Bipolar disorder: n (%)	120 (0.2%)	104 (0.2%)	116 (0.2%)	102 (0.2%)	210 (0.2%)	192 (0.1%)	218 (0.2%)	215 (0.2%)
Other psychiatric disorder: n (%)	2,744 (5.0%)	2,641 (4.8%)	2,897 (5.2%)	2,698 (4.8%)	3,767 (2.8%)	3,475 (2.6%)	4,200 (3.2%)	3,904 (3.0%)
Psychiatric hospitalization: mean (sd)	0.03 (0.21)	0.03 (0.21)	0.03 (0.22)	0.03 (0.23)	0.02 (0.17)	0.02 (0.17)	0.02 (0.18)	0.02 (0.17)
Psychiatric office visit: mean (sd)	1.71 (5.51)	1.67 (5.44)	1.87 (6.19)	1.85 (6.21)	1.44 (4.68)	1.35 (4.10)	1.64 (5.11)	1.50 (4.40)
Anticonvulsants/antiepileptic drugs: n (%)	3,136 (5.8%)	2,932 (5.4%)	3,309 (5.9%)	3,024 (5.4%)	8,247 (6.2%)	7,619 (5.7%)	8,247 (6.2%)	7,662 (5.8%)
SSRIs (without sertraline): n (%)	8,860 (16.3%)	8,260 (15.2%)	9,223 (16.6%)	8,589 (15.4%)	19,990 (15.0%)	18,746 (14.1%)	19,786 (15.0%)	18,606 (14.1%)
SNRIs: n (%)	2,732 (5.0%)	2,574 (4.7%)	2,880 (5.2%)	2,697 (4.8%)	6,571 (4.9%)	6,201 (4.7%)	6,485 (4.9%)	6,043 (4.6%)
Tricyclic antidepressants: n (%)	1,278 (2.3%)	1,195 (2.2%)	1,385 (2.5%)	1,322 (2.4%)	3,415 (2.6%)	3,236 (2.4%)	3,425 (2.6%)	3,291 (2.5%)
Antipsychotics: n (%)	1,994 (3.7%)	1,887 (3.5%)	2,247 (4.0%)	2,098 (3.8%)	5,126 (3.9%)	4,762 (3.6%)	5,255 (4.0%)	4,948 (3.7%)
Benzodiazepines: n (%)	10,330 (19.0%)	9,730 (17.9%)	11,283 (20.3%)	10,541 (18.9%)	25,608 (19.3%)	24,494 (18.4%)	26,282 (19.9%)	24,932 (18.9%)
Non-BZD sedative hypnotics: n (%)	5,657 (10.4%)	5,225 (9.6%)	6,241 (11.2%)	5,713 (10.3%)	14,569 (11.0%)	13,899 (10.5%)	14,907 (11.3%)	13,932 (10.5%)
Outpatient visits: mean (sd)	0.21 (0.45)	0.21 (0.49)	0.20 (0.44)	0.20 (0.47)	0.17 (0.42)	0.16 (0.41)	0.17 (0.42)	0.16 (0.41)
Inpatient hospitalization: mean (sd)	0.01 (0.11)	0.01 (0.12)	0.01 (0.11)	0.01 (0.11)	0.00 (0.01)	0.00 (0.01)	0.00 (0.01)	0.00 (0.01)
ED visit: mean (sd)	0.27 (0.88)	0.25 (0.86)	0.30 (1.28)	0.28 (0.95)	0.35 (1.06)	0.31 (1.06)	0.35 (1.07)	0.32 (1.04)
Number of distinct medication prescriptions: mean (sd)	4.34 (4.18)	4.16 (4.10)	4.46 (4.28)	4.25 (4.16)	4.86 (4.75)	4.71 (4.58)	4.68 (4.70)	4.56 (4.52)
Combined Comorbidity Score: mean (sd)	0.29 (1.08)	0.27 (1.01)	0.32 (1.10)	0.29 (1.02)	0.30 (1.12)	0.28 (1.05)	0.32 (1.09)	0.30 (1.05)
