# Supplementary material for: Comparative effectiveness of generic and brand-name medication use: A database study of US health insurance claims
Source: PLoS Med. 2019 Mar 13;16(3):e1002763. doi: 10.1371/journal.pmed.1002763 (PMC6415809; doi:10.1371/journal.pmed.1002763)

S2 Figure: Hazard ratios (HR) and 95% confidence intervals (CI) comparing outcomes between authorized generic (AG) versus brand initiators and generic versus brand initiators before 1:1 propensity score matching in each database

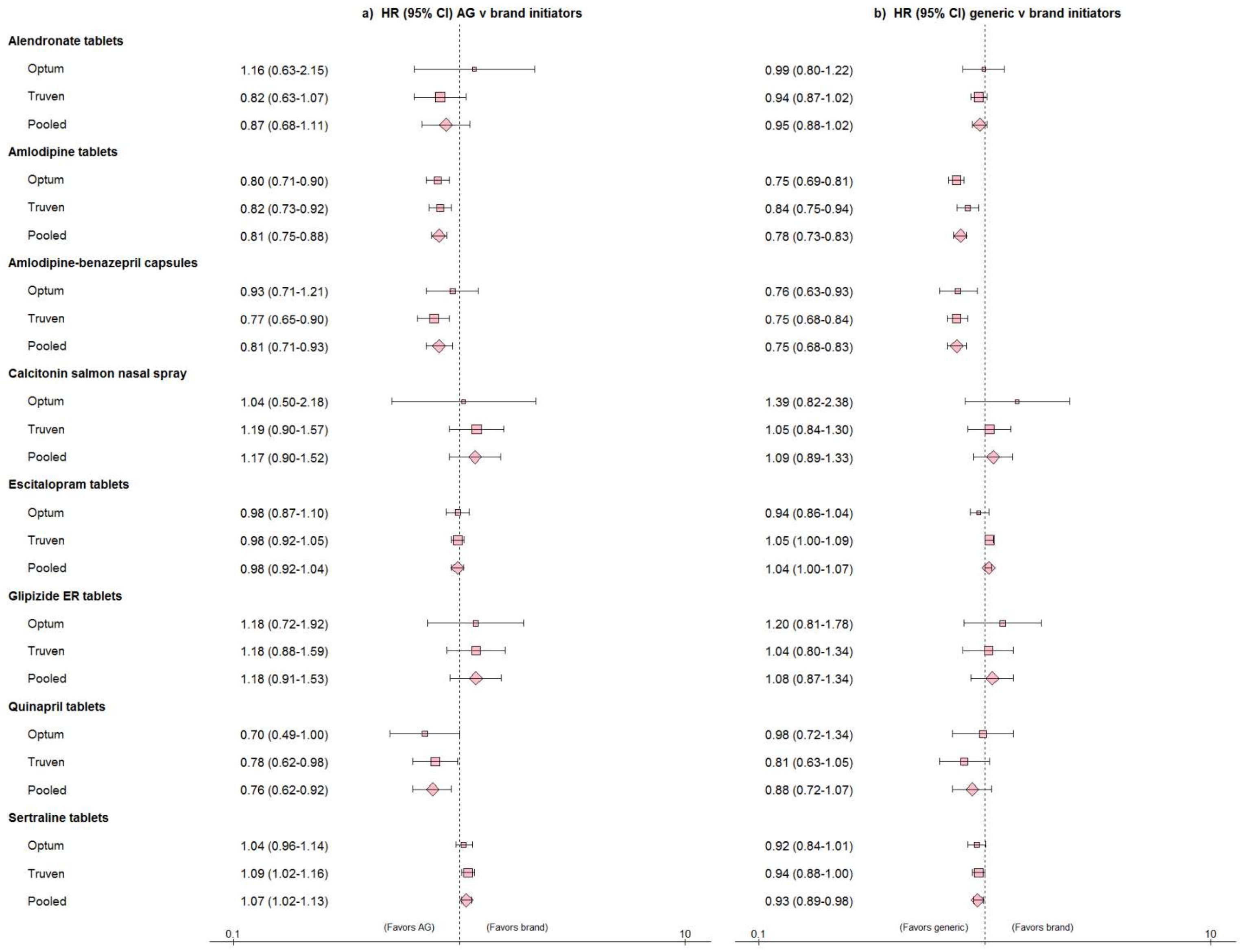

Supplement: S2 Fig — The outcome for amlodipine tablets, amlodipine-benazepril capsules, and quinapril tablets was a composite endpoint comprising hospitalization for myocardial infarction, ischemic stroke, or coronary revascularization procedures. The outcome for alendronate tablets and calcitonin salmon nasal spray was a composite non-vertebral fracture endpoint comprising humerus, wrist, hip, or pelvis fractures. The outcome for escitalopram tablets and sertraline tablets was hospitalization with a psychiatric condition as the principal discharge diagnosis code. The outcome for glipizide extended release (ER) tablets was initiation of insulin during the follow-up period. (PDF) [file pmed.1002763.s003.pdf]
