# Supplementary material for: Comparative effectiveness of generic and brand-name medication use: A database study of US health insurance claims
Source: PLoS Med. 2019 Mar 13;16(3):e1002763. doi: 10.1371/journal.pmed.1002763 (PMC6415809; doi:10.1371/journal.pmed.1002763)
Supplement: S1 STROBE checklist — (PDF) [file pmed.1002763.s004.pdf]

STROBE Statement—Checklist of items that should be included in reports of ***cohort studies***

	Item No	Recommendation	Page no
**Title and abstract**	1	(*a*) Indicate the study's design with a commonly used term in the title or the abstract	Abstract, Methods & Findings- First Sentence
		(*b*) Provide in the abstract an informative and balanced summary of what was done and what was found	Yes (Under the Heading Abstract)
**Introduction**			
Background/rationale	2	Explain the scientific background and rationale for the investigation being reported	Introduction, paragraphs 1-3
Objectives	3	State specific objectives, including any prespecified hypotheses	Introduction, paragraph 4
**Methods**			
Study design	4	Present key elements of study design early in the paper	Methods, Section-Study Design (page 11)
Setting	5	Describe the setting, locations, and relevant dates, including periods of recruitment, exposure, follow-up, and data collection	Methods, Sections-Data Source, Drug Products, Outcomes of Interest and Follow-up
Participants	6	(*a*) Give the eligibility criteria, and the sources and methods of selection of participants. Describe methods of follow-up	Methods, Section-Study Design (page 11)
		(*b*) For matched studies, give matching criteria and number of exposed and unexposed	Tables A-AG in S1 Appendix
Variables	7	Clearly define all outcomes, exposures, predictors, potential confounders, and effect modifiers. Give diagnostic criteria, if applicable	Methods, Sections-Outcomes of Interest and Follow-up, Covariates
Data sources/ measurement	8*	For each variable of interest, give sources of data and details of methods of assessment (measurement). Describe comparability of assessment methods if there is more than one group	Methods, Section- Data source
Bias	9	Describe any efforts to address potential sources of bias	Methods, Section-Study design (Use of authorized generics as design tool to address bias)
Study size	10	Explain how the study size was arrived at	NA (Secondary analysis of existing data)
Quantitative variables	11	Explain how quantitative variables were handled in the analyses. If applicable, describe which groupings were chosen and why	NA
Statistical methods	12	(*a*) Describe all statistical methods, including those used to control for confounding	Methods, Section-Statistical analysis
		(*b*) Describe any methods used to examine subgroups and interactions	NA
		(*c*) Explain how missing data were addressed	Methods, Section-Study design (Require continuous insurance eligibility to prevent

			missing data)
		(*d*) If applicable, explain how loss to follow-up was addressed	NA (Retrospective study)
		(*e*) Describe any sensitivity analyses	Methods, Section- Data source, Drug products (Analyses conducted for two databases and eight drug products selected to maximize generalizability)
**Results**			
Participants	13*	(a) Report numbers of individuals at each stage of study—eg numbers potentially eligible, examined for eligibility, confirmed eligible, included in the study, completing follow-up, and analysed	Tables A-AG in S1 Appendix
		(b) Give reasons for non-participation at each stage	Tables A-AG in S1 Appendix
		(c) Consider use of a flow diagram	Numbers provided in Tables
Descriptive data	14*	(a) Give characteristics of study participants (eg demographic, clinical, social) and information on exposures and potential confounders	Tables A-AG in S1 Appendix
		(b) Indicate number of participants with missing data for each variable of interest	NA
		(c) Summarise follow-up time (eg, average and total amount)	Tables 2 & 4
Outcome data	15*	Report numbers of outcome events or summary measures over time	Tables 2 & 4
Main results	16	(*a*) Give unadjusted estimates and, if applicable, confounder-adjusted estimates and their precision (eg, 95% confidence interval). Make clear which confounders were adjusted for and why they were included	S1 and S2 Figures
		(*b*) Report category boundaries when continuous variables were categorized	NA
		(*c*) If relevant, consider translating estimates of relative risk into absolute risk for a meaningful time period	Tables 2 & 4 (Incidence rates reported)
Other analyses	17	Report other analyses done—eg analyses of subgroups and interactions, and sensitivity analyses	NA
**Discussion**			
Key results	18	Summarise key results with reference to study objectives	Discussion, paragraph 1
Limitations	19	Discuss limitations of the study, taking into account sources of potential bias or imprecision. Discuss both direction and magnitude of any potential bias	Discussion, paragraph 5
Interpretation	20	Give a cautious overall interpretation of results considering objectives, limitations, multiplicity of analyses, results from similar studies, and other relevant evidence	Discussion, paragraph 2-5
Generalisability	21	Discuss the generalisability (external validity) of the study results	Discussion, paragraph 4
**Other information**			

Funding	22	Give the source of funding and the role of the funders for the present study and, if applicable, for the original study on which the present article is based	Provided under the Section: Additional Information

*Give information separately for exposed and unexposed groups.

Note: An Explanation and Elaboration article discusses each checklist item and gives methodological background and published examples of transparent reporting. The STROBE checklist is best used in conjunction with this article (freely available on the Web sites of PLoS Medicine at http://www.plosmedicine.org/, Annals of Internal Medicine at http://www.annals.org/, and Epidemiology at http://www.epidem.com/). Information on the STROBE Initiative is available at http://www.strobe-statement.org.
