# Supplementary material for: Comparative effectiveness of generic and brand-name medication use: A database study of US health insurance claims
Source: PLoS Med. 2019 Mar 13;16(3):e1002763. doi: 10.1371/journal.pmed.1002763 (PMC6415809; doi:10.1371/journal.pmed.1002763)
Supplement: S1 Text — Funding proposal submitted to the US Food and Drug Administration. (PDF) [file pmed.1002763.s005.pdf]

RESEARCH STRATEGY

Significance

Importance of generic drugs on health system and patient outcomes

More than 8 out of every 10 prescriptions dispensed in the US are for generic drugs. Generic medications contain the same amount of the same active ingredient as their brand-name counterparts and must demonstrate bioequivalence to approved brand-name products. Bioequivalence is usually established on the basis of the maximum serum concentration of the drug (C_{max}), the time until maximum concentration is reached, or the area under a curve defined by serum concentration as a function of time (AUC). For example, one of FDA's definitions of bioequivalence requires that the 90% confidence intervals for the ratio of brand-to-generic AUC and C_{max} fall within an acceptance interval of 0.80-1.25 (known as the "-20%/+25% rule").[1] In reality, the bioequivalence between brand-name and generic drugs is much narrower. A recent review of more than 2,000 clinical bioequivalence studies of orally administered generic products approved by FDA found that the average differences between generic and innovator products with respect to peak drug concentration and area under the curve were 4.4 and 3.6%, respectively.[2] In nearly 98% of the studies reviewed, the properties of generic products differed from those of the brand-name product by less than 10%.[2]

Due largely to the success of the Drug Price Competition and Patent Term Restoration Act of 1984 (the Hatch-Waxman Act), generic drugs are much less costly than their brand-name counterparts. While comprising 84% of all prescriptions dispensed in the US in 2012, generic drugs account for only about 20% of spending on prescription drugs in the US.[3] Generic drugs therefore reduce overall health system spending.[4,5] In several studies, we have also found that, by lowering out-of-pocket drug costs for patients, generic drugs promote better medication adherence.[6] In recently completed work, we found that patients who initiated a generic statin had better adherence to statin therapy than patients who initiated a brand-name version. We also found that, by improving adherence, initiating treatment with a generic statin resulted in better clinical outcomes. Those who initiated generic statins had an 8% lower rate of subsequent major adverse cardiovascular outcomes than those who initiated brand-name versions of the same drugs (Gagne JJ et al; *manuscript under review*).

Negative perceptions of generic drugs as a source of bias in observational studies

Despite potential financial savings and clinical benefits associated with generic drug use, negative perceptions of generics are prevalent[7,8] and patients do not always use generic drugs when they are available. In a survey of attitudes toward generic antiepileptic medications, 75% of physicians expressed concern about the efficacy of generic antiepileptics and 56% expressed concerns about the safety of these drugs.[9] Among patients, 65% reported concerns about generic antiepileptic efficacy and 58% expressed concern about safety. Case studies in the medical literature have described adverse outcomes related to generic switching,[10] leading editorialists to warn against substitution of generic drugs.[11,12] It is very likely that these negative perceptions cause patients to switchback from generic to brand-name versions, which would explain why a number of studies have found high switchback rates with generic drugs. However, the authors of these studies generally attribute switchbacks to poor patient experiences with generic drugs, rather than to preconceived negative perceptions. In recent work, we found that prior experience with generic drugs was a strong predictor of subsequent use of other generic drugs. Because the converse is also true – that patients who did not have prior generic use were

more likely to initiate subsequent treatment with a brand-name drug – this suggests that perceptions of generics are an important driver of usage.

Authorized generics as a tool to assess bias in generic drug studies

While claims data do not contain information on patient perceptions of generic drugs, authorized generics offer a clever opportunity to indirectly assess negative perceptions and their impact on observational studies of generic drugs.

Authorized generics are brand-name drugs that are marketed, sold, or distributed as generic medications, usually either by a subsidiary of the brand-name manufacturer or by another company licensed to do so. Since they are produced by the brand-name manufacturer, authorized generics are chemically-identical to the brand-name versions because they contain exactly the same active and inactive ingredients as the brand-name version, but they may differ in labeling, packaging, product code, labeler code, trade name, or trademark.

Authorized generics can serve as a tool to examine the extent to which negative perceptions of generic drugs affect acceptance of generics because they are essentially brand-name drugs that are perceived as generic drugs. We will use authorized generics to elucidate whether differences in substitution, switchbacks, medication adherence, medical utilization rates, and clinical outcomes are associated with negative perceptions of generics or to properties of generic products marketed by other companies. If generic drugs are truly less safe or effective than brand-name versions, then differences in these outcomes would be observed when comparing generics to authorized generic versions since authorized generics are chemically identical to the corresponding brand products. If other generics are not associated with differences in outcomes when compared to authorized generics, but both other generics and authorized generics are associated with worse outcomes when compared to the brand, then we would be able to conclude that this is due to negative perceptions of generics drugs since authorized generics are chemically identical to brand-name products and since patients generally do not know whether they receive authorized generics or other generics such that any negative perceptions would effect both equally.

Using 25 model authorized generics, this project will systematically and comprehensively assess whether and the extent to which negative perceptions of generic drugs affect clinical outcomes when comparing generic and brand-name drugs.

Approach

Compare substitution and switchback rates and clinical outcomes between authorized generic and other generic versions of model drug products and between other generic versions and brand versions of these drugs

We will use multiple large electronic healthcare claims databases to determine the extent to which negative perceptions of generic drugs bias observational studies of these drugs by conducting a series of analyses to: (1) compare substitution rates when an authorized generic is marketed versus when only other generics are marketed; (2) compare switchback rates from authorized generic to brand-name versions and from other generics to brand-name versions; and (2) compare clinical outcomes associated with authorized generics versus other generic products and other generics versus brand versions.

3.D.I.a. Hypotheses

We hypothesize that substitution rates, switchback rates, and clinical outcomes will not differ between authorized generics and other generics and that clinical outcomes will be equal or better with both authorized generics and other generics than with brand-name products.

Data set

The Optum© Clinformatics® Data Mart comprises a large, geographically diverse population of health insurance beneficiaries enrolled in commercial UnitedHealth Group-affiliated health plans. The database includes information relating to approximately 14 million patients on a yearly basis and 60 million patients in total. It comprises complete medical claims from healthcare providers and facilities, outpatient pharmacy dispensing records, and enrollment information that provides demographic data and dates of insurance eligibility for persons in the database.

Model drug products

The substitution, switchback, and outcomes studies will focus on 25 authorized generic products (**Table 1**). We have drawn these drugs from FDA's List of Authorized Generic Drugs: www.fda.gov/downloads/AboutFDA/CentersOffices/OfficeofMedicalProductsandTobacco/CDER/UCM183605.pdf. We selected the drugs based on whether relevant clinical outcomes can be accurately measured in administrative claims data. In Table 1 we include the clinical outcome that we will investigate for each drug. Generic versions of each drug were first approved within the 1996-2013 time period for which we have data.

Table 1. Proposed set of 25 model authorized generic drugs

Drug class	Drug and dosage form	Clinical outcome
Antiepileptic drugs	Carbamazepine extended release tablets	Seizure-related hospitalizations[14]
	Gabapentin capsules	
	Lamotrigine tablets	
	Oxcarbazepine tablets	
Antidepressants	Bupropion extended release tablets	Psychiatric hospitalizations[40]
	Citalopram tablets	
	Escitalopram tablets	
	Paroxetine tables	
	Sertraline tablets	
	Venlafaxine extended release tablets	
Cardiovascular drugs	Amlodipine tablets	Composite cardiovascular outcomes (hospitalization for acute coronary syndromes or stroke)[41]
	Atorvastatin tablets	
	Diltiazem extended release capsules	
	Lorsartan tablets	
	Metoprolol extended release tablets	
	Pravastatin tablets	
	Quinapril tablets	

	Simvastatin tablets	
	Verapamil extended release capsules	
Antidiabetes drugs	Glipizide extended release tablets	Insulin initiation as a proxy for treatment intensification[42]
	Glimepiride tablets	
	Glyburide tablets	
	Metformin tablets	
Osteoporosis drugs	Alendronate tablets	Composite fracture outcome (hip, humerus, pelvis, wrist)[43]
	Calcitonin salmon nasal spray	

Compare substitution rates when an authorized generic is marketed versus when only other generics are marketed

We will use healthcare claims data to compare substitution rates when an authorized generic is marketed (i.e., in the case of the 25 model drugs of interest) versus when only other generics are marketed. The control drugs for this analysis (i.e., drugs for which only other generics were marketed and no authorized generic was marketed) will be selected in collaboration with FDA and will ideally be in the same class as the models of interest. For example, lovastatin, which is not on FDA's list of authorized generic drugs, would be a reasonable control drug for atorvastatin, pravastatin, and simvastatin.

We will identify the first generic approval date for each of the 25 model drugs of interest and for each corresponding control drug. We will then identify all prescriptions filled in each database for each drug of interest using the National Drug Code associated with each prescription and use this to determine the manufacturer of the drug that the patient filled. We will then plot the proportion of prescription fills for brand versus generic versions following the generic introduction. We will compare the monthly proportions within each model and control drug pair to determine whether substitution rates are higher, lower, or the same when an authorized generic is and is not marketed. We hypothesize that substitution rates will be similar for authorized generics and for other generic products.

Compare rates of switchbacks from authorized generic to brand-name versions to switchback rates from other generics to brand-name versions

We will use healthcare claims data to compare switchback rates among patients who switched from brand to authorized generic versions of each of the 25 drugs of interest to those who switched from brand to other generic versions of the same drugs. For each drug of interest we will use a cohort design to determine the cumulative proportions of patients who switched from a brand to an authorized generic version and from a brand to another

Figure 2: Kaplan-Meier comparing acarbose vs. nateglinide switchbacks

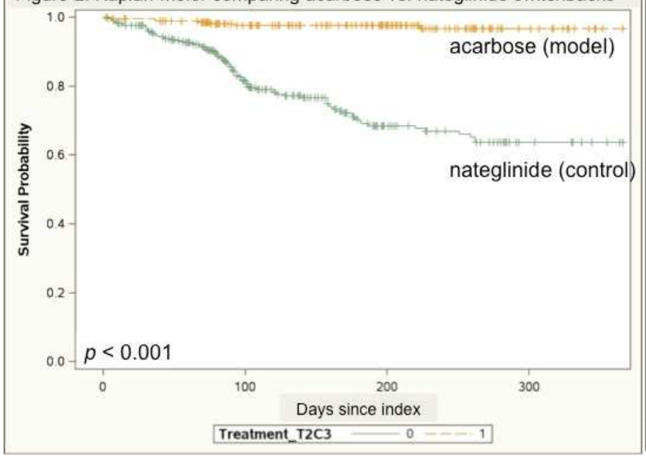

Figure 3: Kaplan-Meier comparing acarbose vs. glimepiride switchbacks

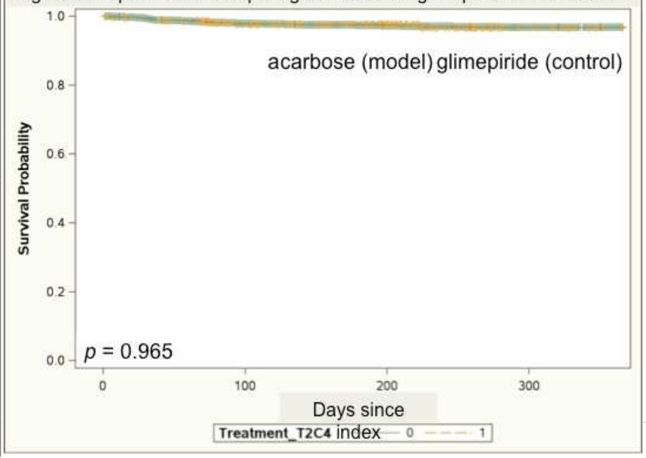

generic version and, secondly, the proportion of those patients who switched back from each generic type. Brand-to-generic switching will be defined – as we have previously done[14] – by identifying patients who received a prescription dispensing for a brand version of the drug of interest and subsequently refilled the prescription with a generic version of the same drug, holding constant the dose and dosage form. We will define switchback as a subsequent prescription for the brand version, following the initial switch, again holding constant the dose and dosage form.

We will use Kaplan-Meier plots to calculate cumulative switch and switchback proportions while accounting for loss-to-follow-up, which could be due to discontinuation of the drug product, death, or disenrollment from the insurance plan. We will use log-rank tests to compare the curves. **Figure 2** shows an example of such a Kaplan-Meier plot from our ongoing work that compares switchback rates with six model generic drugs approved by innovative pathways to switchback rates with control drugs that were approved through conventional pathways. The "Survival Probability" on the y-axis represents the probability of remaining on the generic (i.e., the probability of not switching back). In this example, the orange line on top (i.e., Treatment_T2C3 = 1) represents the model generic drug acarbose and the green line on the bottom (i.e., Treatment_T2C3 = 0) represents the control drug nateglinide. In this example, we see that switchback rates are much higher with nateglinide than with acarbose ($p < 0.001$), suggesting that the innovative approval pathway did not increase patients' likelihood of switching back to the brand version of acarbose as compared to the switchback rate for nateglinide.

We hypothesize that switchback rates for authorized generic versions will be very similar to switchback rates for other generic versions. **Figure 3** shows an example from our ongoing work in which switchback rates with acarbose are virtually identical to those with glimepiride, a control drug ($p = 0.9651$). We hypothesize that our analyses of authorized generics versus other generics will yield results similar to this example.

The primary switchback analyses will be among those with a current prescription at the time of generic entry. For these patients, the time scale will begin at the generic entry date; that is, the generic entry date will be t_0. We will also conduct stratified analyses in which we stratify patients based on their duration of brand product use preceding the generic entry date. Secondary analyses will focus on patients who initiate a brand product after the generic entry date. For these patients, the time scale will begin at the brand product initiation date. We will follow patients for brand-to-generic switches for 180 days following t_0. Among those patients who switch, we will follow them for switchbacks over the 180 days following the switch date. Because generic drug utilization can be negatively impacted by differences in appearance between brand-name and generic versions, which might occur less frequently with authorized generics, we will repeat these analyses restricted to other generics with similar appearance to the brand-name version.

Because we have already conducted the switch and switchback analyses for the six model drugs of interest and their control drugs in our ongoing project, we have programming code that can be readily reused to conduct the analyses of the 25 drugs of interest quickly and efficiently.

Compare clinical outcomes associated with use of authorized generics versus other generic products

We will compare clinical outcomes among initiators of brand-name, authorized generic, and other generic versions of the 25 drugs of interest.

We will use a propensity-score matched, parallel, active-comparator new user cohort design. Cohort approaches are particularly useful when comparing outcomes between patients exposed to different medical products, such as between authorized generic versus other generic versions of the same drugs or between generic users and brand users of the same drugs. Focusing on new users ensures accurate assessment of temporality among exposures, outcomes, and other study variables, and ensures that outcomes that occur shortly after initiation are captured. Propensity scores are a powerful tool used to minimize confounding by balancing a potentially large number of possible confounders between exposure groups.

We will identify new users of the product(s) of interest and new users of the comparator product (or products). New use is defined by no prior use of the product (or potentially of other pre-specified products) in a pre-specified period preceding each patient's product initiation (i.e., index) date. Outcomes are identified over a pre-specified risk window following product initiation.

Potential confounders are identified in a baseline period of pre-specified length preceding each patient's index date. Importantly, all confounders are measured before exposure to the medical product. Pre-defined potential confounders are entered into a database-specific propensity score model. A separate propensity score is estimated in each database and patients are matched by propensity score within each database.

We have selected clinical outcomes for each drug that can be defined in claims data with high specificity. In addition to comparing brand and generic versions, we will also compare authorized generics to other generic versions and then separately compare authorized generics to brand-name versions and other generics to brand-name versions for each outcomes of interest.

For each of the 25 model drugs, we will develop NDC lists for brand versions, authorized generic versions, and other generic versions. Much of the work to develop these lists will be completed as part of the analyses of substitution and switchback rates. We will also develop a list of risk factors for each of the outcomes of interest that will serve as the pre-defined covariates for analyses involving those outcomes. We have developed a preliminary list of risk factors for the composite cardiovascular outcome in **Table 2**. Similar lists will be developed for each outcome.

For each of the 25 drugs, we will assess clinical outcomes in the following comparisons: (1) brand versus generic initiators; (2) authorized generic versus other generic initiators; (3) authorized generis versus brand-name initiators; and (4) other generic versus brand-name initiators.

**Table 2. Potential risk factors for cardiovascular events**
**Socio-demographics**
Age
Sex
Race
**Health services utilization**
Number of physician visits
Number of days in hospital
Number of drugs dispensed
**Clinical characteristics**
Comorbidity score
Prior gastrointestinal bleed
Prior hemorrhagic stroke
Prior ischemic stroke
Prior myocardial infarction
Prior transient ischemic attach
Atrial fibrillation
Prior venous thromboembolism
Acute coronary syndrome prior to index hospitalization
Valve replacement
Diabetes
Hypertension
Hyperlipidemia
Peripheral artery disease
**Drug utilization**
Angiotensin-converting enzyme inhibitors
Antidiabetic drugs
Beta-blockers
H2-blockers
Non-steroidal anti-inflammatory drugs
Proton pump inhibitors
Statins
Warfarin
Use of other antidepressants
Use of other potentially interacting drugs

Limitations

There are many limitations to administrative data and observational research methods. Administrative data often lack information on important confounders (e.g. smoking status, body mass index, etc.) and on health outcomes that do not result in medical care (e.g., a minor bleeding). Pharmacy claims data provide accurate information about drugs dispensed to patients, but do not provide insight into whether patients actually consume the medications. These limitations can result in misclassification of study variables, including exposures, outcomes, and potential confounders. If non-differential, misclassification of exposures and outcomes will generally result bias toward the null. Misclassification of confounders will result in less complete adjustment than if the variables were accurately measured. Several measures will be taken to examine the robustness of study findings, including the use of control drugs and propensity scores to address confounding.
